# TiO_2_-Catalyzed Direct Diazenylation of Active Methylene Compounds with Diazonium Salts

**DOI:** 10.1021/acs.joc.4c02266

**Published:** 2024-12-18

**Authors:** Edson Evangelista, Yuri P. V. de Carvalho, Iva S. de Jesus, Maria Eduarda S. Rodrigues, Emelli P. Hayashi, Karine N. de Andrade, Rodolfo G. Fiorot, Luana da S. M. Forezi

**Affiliations:** † Department of Organic Chemistry, Institute of Chemistry, Federal Fluminense University—UFF, Niteroi, Rio de Janeiro 24020-141, Brazil; ‡ Department of Pharmaceutical Technology, Federal Fluminense University—UFF, Niteroi, Rio de Janeiro 24241-000, Brazil

## Abstract

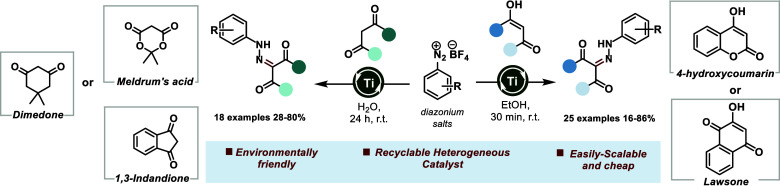

Herein, we report a direct diazenylation of active methylene compounds that is environmentally friendly, cost-effective, and scalable, utilizing a heterogeneous TiO_2_ catalyst that is both accessible and recyclable. The scope of this transformation shows excellent compatibility with both electron-rich and electron-poor diazonium salts, yielding the desired products in very good yields at room temperature.

## Introduction

**Figure 1:**
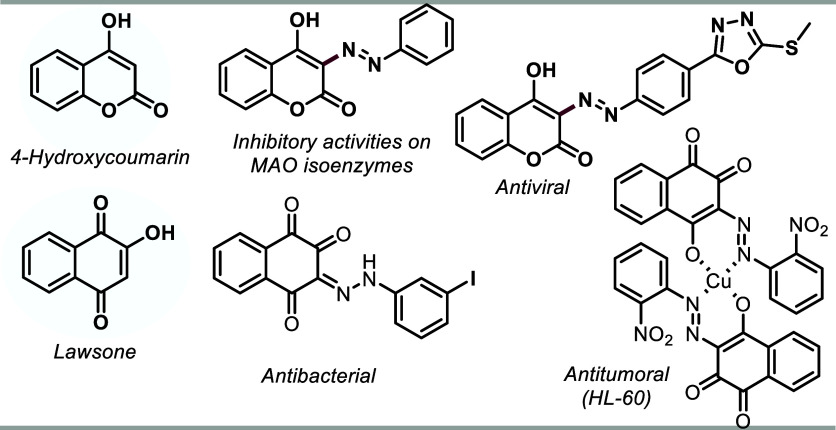
Bioactive compounds containing the 4-hydroxycoumarin and lawsone moieties.

Azo compounds have garnered significant interest due to their notable properties and diverse applications.^1−3^ They are widely explored in fields such as dyes, advanced materials, and therapeutic agents. The versatility of these compounds is further enhanced by their potential for structural modifications to meet various needs. In particular, 4-hydroxycoumarin and lawsone azo-derivatives exhibit promising bioactivities, including antitumoral,^4,5^ antibacterial,^5^ antimicrobial,^6^ and antioxidant effects.^4^ Moreover, they are important intermediates for the synthesis of promising bioactive compounds (Figure 1).^5^

**Scheme 1:**
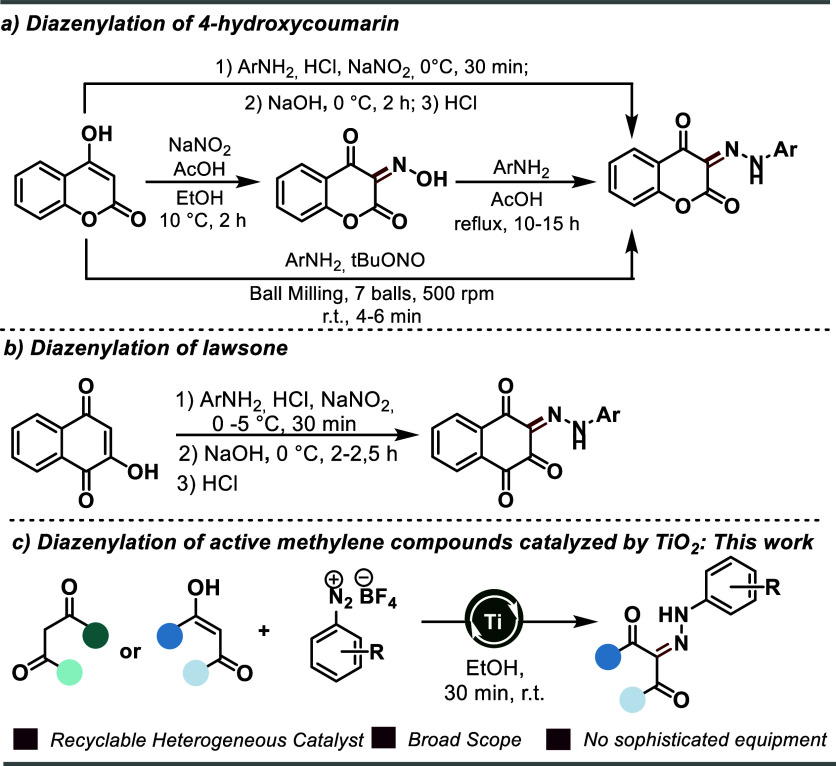
(a) Diazenylation of 4-Hydroxycoumarins Using Basic Catalysts; (b) Azo-Coumarins in 2 Steps through Nitrosation of 4-Hydroxycoumarin; (c) C-3 Dehydrogenative Aza-Coupling of 4-Hydroxycoumarins via Mechanochemistry; (d) Diazenylation of Lawsone Using Basic Catalysis; (e) Our Proposal: TiO_2_-Catalyst Direct Diazenylation of Active Methylene Compounds

The most straightforward synthetic pathway for synthesizing 3-azo-4-hydroxycoumarins involves a direct diazenylation reaction at the C3 position. In these protocols, a stoichiometric amount of base promotes the deprotonation of hydroxyl group, making the C3 carbon an excellent nucleophilic center, giving rise to an addition product (Scheme 1a).^7^ Alternatively, the nitrosation of 4-hydroxycoumarin followed by condensation with different anilines represents a secondary route (Scheme 1a).^8^ Brahmachari and co-workers described the insertion of the azo group into 4-hydroxycoumarin via mechanochemistry using *t*BUONO in the presence of different anilines (Scheme 1a). However, using a highly toxic agent represents a drawback and a limitation of the protocol.^9^ Diazenylation of lawsone typically occurs through the prior formation of the diazonium salt from anilines, followed by the addition to lawsone facilitated by a base (Scheme 1b).^5^ Therefore, identifying alternative catalytic systems that are inexpensive, readily available, and do not require additives or specialized equipment is highly desirable.

In recent years, heterogeneous catalysis has emerged as a valuable technology for performing transformations under mild reaction conditions.^10^ Among the advantages of using a heterogeneously catalyzed approach are process simplification, easy separation, and catalyst recycling.^11,12^ In this context, using TiO_2_ as a heterogeneous catalyst provides an environmentally friendly alternative for promoting chemical reactions. Due to its low cost, nontoxicity, and high chemical stability, TiO_2_ is one of the most popular heterogeneous catalysts.^13−15^

Considering these aspects, we developed a direct diazenylation of active methylene compounds using TiO_2_ as a heterogeneous catalyst for incorporating the azo group into the structure of cyclic 1,3-dicarbonyl compounds (or in its enol form). This catalyst is easily accessible, recyclable, and applicable to a broad spectrum of substrates, yielding a series of azo-compounds under mild and environmentally friendly reaction conditions (Scheme 1c).

## Results and Discussion

We started our study with a preliminary investigation of the reaction between 4-hydroxycoumarin **1** and aryldiazonium salt **2a** to evaluate the effect of various experimental parameters on the reaction outcome. After a set of preliminary experiments, we identified that using 1.0 equiv of **1**, 1.0 equiv of **2a**, and 0.25 equiv of TiO_2_ in EtOH afforded the desired product **3a** in 81% yield, after 30 min of reaction at room temperature (Table 1, entry 1). Entries 2–11 summarize some deviations from the optimized conditions. Studies on the solvent have shown that ethanol is the most efficient for promoting the transformation (Table 1, entries 2–4). Subsequently, the influence of different metal oxides was investigated. A slight reduction in the reaction efficiency was further observed when Al_2_O_3_ and Cu_2_O were used as heterogeneous catalysts (entries 5 and 6, 54 and 40% yields, respectively). When the reaction was conducted with 1.0 equiv of TiO_2_ for 30 min, a slight increase in the yield of **3a** was observed (Table 1, entry 7). In contrast, after 1 h of reaction, **3a** was obtained in 70% yield (Table 1, entry 8). We assume that this fact may be associated with the low stability of **2a** at room temperature.

**Table 1:**
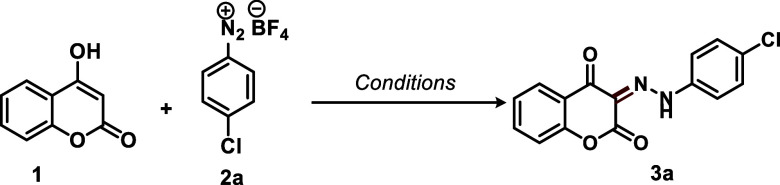
Optimization of the Reaction Conditions

entry	catalyst	solvent	time (h)	temperature	light irradiation	yield (%)
1	TiO_2_ (0.25 equiv)	Et0H	0.5	rt		81
2	TiO_2_ (0.25 equiv)	H_2_O	0.5	rt		52
3	TiO_2_ (0.25 equiv)	Me0H	0.5	rt		57
4	TiO_2_ (0.25 equiv) CH_3_CN	CH_3_CN	0.5	rt		67
5	Al_2_O_3_ (0.25 equiv)	Et0H	0.5	rt		54
6	Cu_2_O (0.25 equiv)	Et0H	0.5	rt		40
7	TiO_2_ (1.0 equiv)	Et0H	0.5	rt		88
8	TiO_2_ (1.0 equiv)	Et0H	1	rt		70
9	absence	Et0H	48	rt		60
10	TiO_2_ (1.0 equiv)	Et0H	1	rt	blue LED	61
11	TiO_2_ (0.25 equiv)	EtOH	1	rt		

Considering environmental aspects, the condition using 0.25 equiv of heterogeneous catalyst was maintained. A control experiment in the absence of TiO_2_ was shown to be detrimental to the reaction outcome leading to the formation of the product in 60% yields after 48 h (Table 1, entry 9). Inspired by the results obtained by Zoller and co-workers,^16^ which described the dual role of titanium dioxide in the visible light heterogeneous catalyzed C–H arylation of heteroarenes with aryldiazonium salts, the direct diazenylation using TiO_2_ light-mediated was also investigated. However, we observed a decrease in the yield of **3a** (entry 10). Lastly, the control experiment revealed that light no is essential to the reaction outcome (entry 11). Titanium oxide behaves as a Lewis acid, facilitating enolization through the hydroxyl group, followed by the diazenylation reaction.

**Scheme 2:**
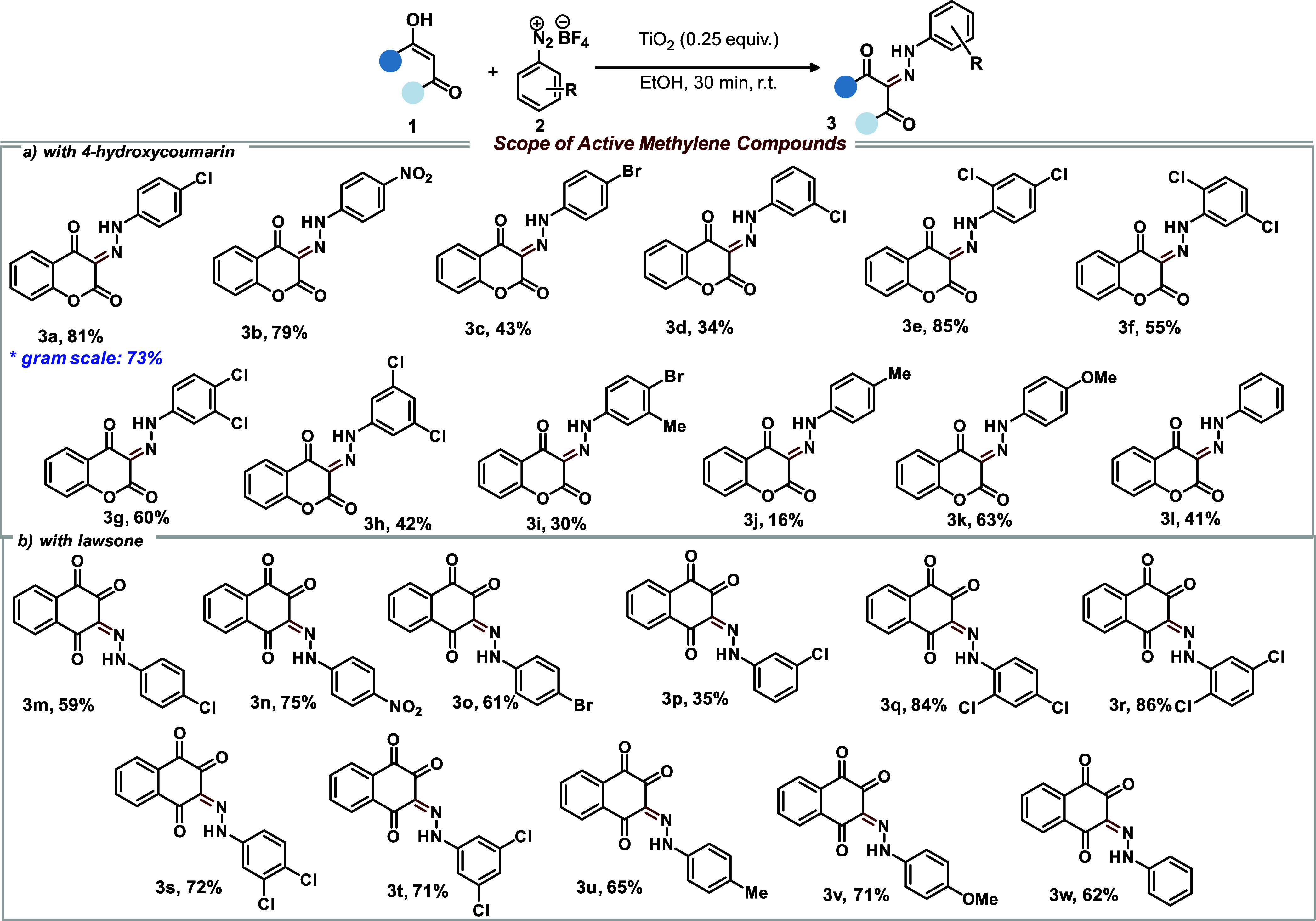
Substrate Scope Investigation Reaction conditions: 0.5 mmol of **1** (4-hydroxycoumarin for [a] or Lawsone for [b]), 0.5 mmol (1.0 equiv) of **2**, and 0.25 equiv of TiO_2_ in 10 mL of EtOH. *Gram scale condition: 5.0 mmol of 4-hydroxycoumarin, 5.0 mmol (1.0 equiv) of aryldiazonium salt and 0.25 equiv of TiO_2_ in 20 mL of EtOH. The yield refers to an isolated compound.

With the optimal conditions in hands, we explored the scope and limitations of this titanium dioxide-mediated direct diazenylation among a wide variety of diazonium salts (Scheme 2a). Accordingly, unsubstituted and aryldiazonium salts containing electron-withdrawing and electron-donating substituents underwent the desired transformation, giving products with satisfactory yields in short reaction time. Importantly, *ortho*, *meta*, *para*, and multiple substitutions were tolerated well under our developed conditions. Additionally, both *p*-Cl and *p*-Br substituted aryldiazonium salts afforded the desired products in good yields (**3a** and **3c**, respectively).

To examine the efficiency of the heterogeneous catalyst, the reaction scale was increased to 5.0 mmol (gram scale). The reaction proceeded satisfactorily and yielded **3a** an almost similar yield (73%), highlighting the potential of the developed protocol as an environmentally friendly methodology. Encouraged by the results of the diazenylation of 4-hydroxycoumarin, we then decided to evaluate the compatibility of this protocol with another similar system, lawsone. As an extension of the substrate scope, we applied similar direct diazenylation conditions to the reaction between lawsone and various substituted aryldiazonium salts (Scheme 2b). The reaction was carried out using 0.25 equiv of TiO_2_ in ethanol at room temperature for 30 min, yielding products in good to excellent yields (35–86%). The use of aryldiazonium salts with substituents at *ortho*, *meta*, and *para* positions, as well as those with multiple substituted, proved to be effective under the developed conditions.

**Scheme 3:**
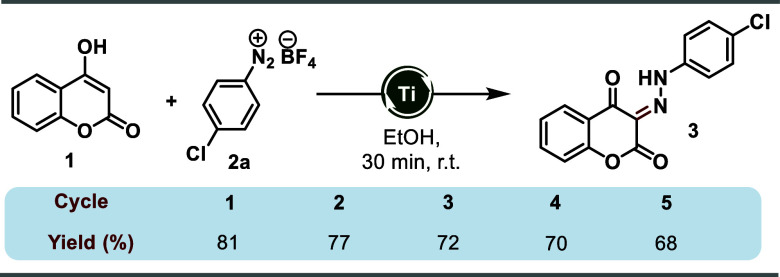
Evaluation of Catalyst Recycling

To further demonstrate the viability of this method, TiO_2_ was recycled after the reaction and reused. The catalyst was easily recovered through centrifugation and then successfully applied in multiple reaction cycles without any loss of reactivity or selectivity (Scheme 3).

**Scheme 4:**
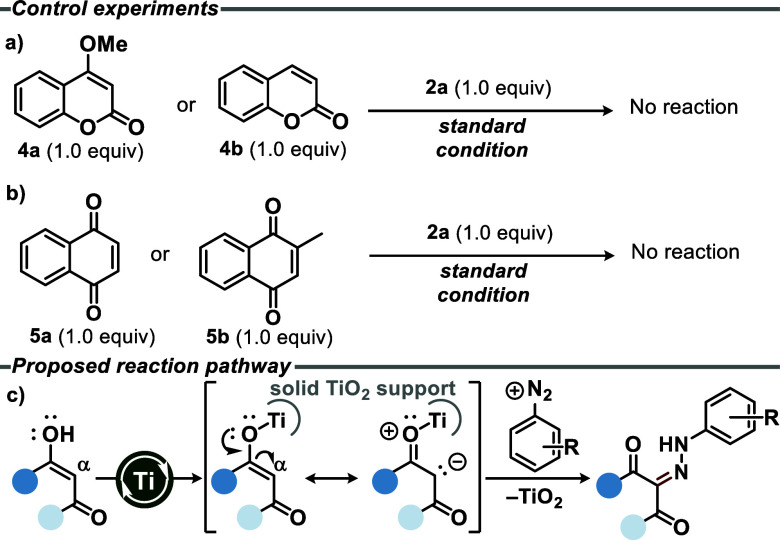
(a,b) Control Experiments; (c) Proposed Reaction Pathway

**Figure 2:**
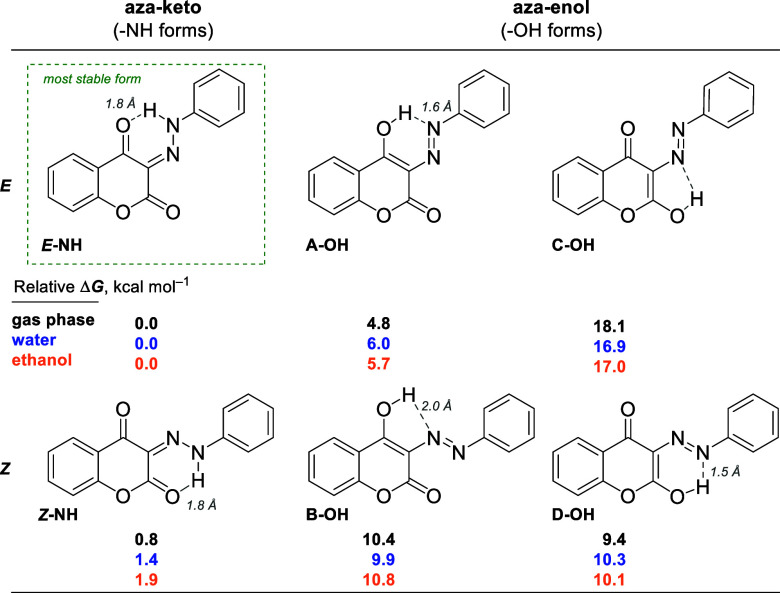
*E*/*Z*-keto-hydrazone (−NH) and azo-enol (A–D)–OH possible structures formed in the diazenylation reactions. The relative energies (in kcal mol^–1^) of the evaluated systems obtained using M06-2X/def2-TZVP@SMD (water or ethanol), were calculated relative to the most stable form (*E*-NH).

Some control experiments were carried out to gain insight into the reaction mechanism (Scheme 4). The role of hydroxy groups in direct diazenylation was examined using 4-methoxy-2*H*-chromen-2-one (**4a**) and 2*H*-chromen-2-one (**4b**) as starting materials under the optimized condition. In both cases, the diazenylation at the C-3 position did not occur and the starting materials were completely recovered (Scheme 4a). 1,4-Naphthoquinone (**5a**) and menadione (**5b**) were also tested as controls, and similarly, no reaction was observed (Scheme 4b). This investigation suggests that the enol moiety is important for the reaction proceeding. It is proposed that the enolic hydroxyl groups act as directing groups, and that the formation of a titanium enolate intermediate is required for the progression of the reaction. These hydroxyl groups function as electron-donating groups (EDG), increasing the electron density on the α-carbon and thereby enhancing its reactivity. In addition, titanium enolate are feasible reaction intermediates with improved nucleophilicity. This rationale explains why the reaction does not proceed with compounds **4a**–**b** and **5a**–**b**. Although the methoxyl group in **4a** has a similar electronic effect, no diazenylation product was observed. During the reaction course (as proposed in Scheme 4c), the enolic hydroxyl group is converted into a carbonyl group, as depicted in the subsequent tautomeric/conformational analysis (Figure 2). In the case of **4a**, the presence of the OCH_3_ bond prevents the enolate formation, and thus, no carbonyl group is generated.

To confirm the success of the diazenylation reactions, we employed ^1^H and ^13^C Nuclear Magnetic Resonance (NMR) spectroscopy, including 1D and 2D techniques, as well as high-resolution mass spectrometry (HRMS). The data are provided in the experimental section.

The aza-keto moiety formed in the proposed structure of the products might undergo a tautomerization that yields the azo-enol product, as reported by other works.^1,7,8,17^ In addition, while the C=N double bond in the aza-keto (defined as –NH, Figure 2) structure might present an *E*- or *Z*-stereochemistry, the azo-enol (defined as –OH, Figure 2) forms might have different conformations (A–D), engaging different intramolecular hydrogen bonds that could stabilize the structures. All these possibilities caught our attention in establishing the most stable and preferential structure for the diazenylation products. Therefore, to correctly point out which structure is the most likely to be isolated, we performed quantum-chemical calculations using Density Functional Theory (DFT) methods to investigate each structure’s relative stability.

The computational protocol employed in these simulations, along with the matrices of the optimized structures, is organized in the Supporting Information file. Figure 2 presents the relative Gibbs free energy for each assessed structure at the M06-2X/def2-TZVP level computed in the gas phase and with implicit solvation (SMD model) in water and ethanol. The computed energy values are relative to the most stable computed form, *E*-NH. Our outcomes reveal that the azo-keto tautomers, *E*/*Z*-NH, are more stable than any of the azo-enol conformations, (A–D)–OH. The higher stability of the azo-keto forms can be attributed to the difference in the overall bond strengths in these structures: the sum of the C=O, C=N, and N–H bond strengths present in the azo-keto form are higher than the C=C, N=N, and O–H bonds in the azo-enol form.^18,19^ Between the azo-keto, the *E* isomer is more stable than *Z*, approximately 2.0 kcal mol^–1^ in ethanol, and this preference remains unaltered despite the solvent effect (gas phase, water, or ethanol) and the DFT level.

We also evaluated the influence of the density functional, performing the same calculations with ωB97X-D, whose results are displayed in the Supporting Information. Overall, the trends are the same, with the *E*-NH being the preferred structure, suggesting this to be the most abundant form. This consistent preference across the investigated solvent environments agrees with the theoretical-experimental identification of the azo form’s preference in solvent (DMSO) in aryldiazenyl compounds, with a preference energy (Δ*E*) of 12.5 kcal mol^–1^ (DFT-B3LYP/6-31G**) for the –NH form. For such systems, the azo form was identified only in the solid state.^20^

Regarding the azo-compounds derived from lawsone, Mangrish and co-workers demonstrated that the azo-keto forms are more stable than the azo-enol forms, regardless of the substituent on the phenylene ring.^5^

**Scheme 5:**
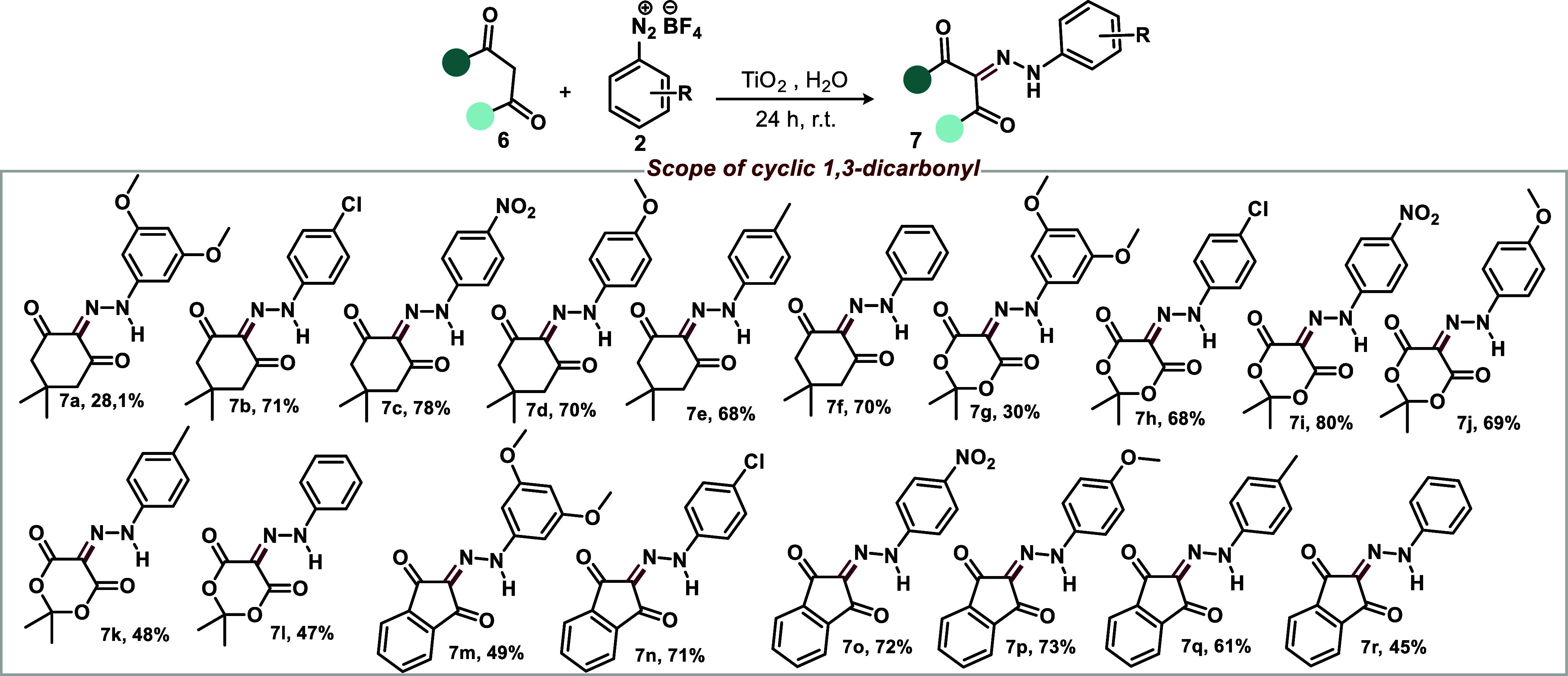
TiO_2_-Catalyzed Direct Diazenylation of Cyclic 1,3-Dicarbonyl Compounds Reaction conditions: 0.5 mmol of **6**, 0.5 mmol (1.0 equiv) of **2**, and 0.25 equiv of TiO_2_ in 10 mL of H_2_O. The yield refers to an isolated compound.

The methodology was also applied to different cyclic 1,3-dicarbonyl compounds, demonstrating the efficiency of the methodology concerning different ring sizes and compositions. It was still possible to optimize the reaction, making it more ecologically viable by replacing the ethanol solvent with water. As a result of replacing the solvent, an increase in yields was observed compared to the previous method. The same study evaluating withdrawal and donor groups in different positions of the diazonium salt was applied, demonstrating that the products, even in different systems, still present good to excellent yields for the investigated cases (Scheme 5).

## Conclusion

In summary, we present a smooth and operationally simple direct diazenylation of active methylene compounds using an accessible, inexpensive, and recyclable heterogeneous catalyst. The structures of the products and the higher stability of the E-azo-keto forms were assessed by DFT calculations. The TiO_2_ catalyst is robust and can be repeatedly used for the diazenylation of cyclic 1,3-dicarbonyl compounds (or in its enol form) with both electron-rich and electron-poor aryldiazonium salts, affording moderate to excellent yields. Importantly, the reaction is carried out under eco-friendly conditions, using mild solvents at room temperature. This efficient synthetic approach for diazenylation of 1,3-dicarbonyl compounds opens up new avenues for the development of novel chemical libraries.

## Experimental Section

### General Experimental Information

Commercially available chemicals and solvents were used without further purification unless otherwise noted. ^1^H NMR and ^13^C NMR spectra were recorded on Bruker 500 and 600 MHz NMR spectrometers. Chemical shifts (δ) are reported in parts per million relative to the residual solvent signals, and coupling constants (*J*) are reported in hertz. The multiplicities are described as brs = broad signal, s = singlet, d = doublet, t = triplet, q = quartet, dd = doublet of doublets, dt = doublet of triplets, dq = doublet of quartets, and m = multiplet. High-resolution mass spectrometry (HRMS) data were obtained using an LC–MS Bruker Daltonics MicroTOF (time-of-flight) analyzer. Reagents and materials were of the highest commercially available grade and used without further purification. Flash column chromatography was carried out using silica gel 60 (230–400 mesh), and analytical thin layer chromatography (TLC) was performed using silica gel aluminum sheets. Visualization of the compounds on TLC was achieved by UV or using a suitable TLC stain.

### Starting Materials

The compound **1** (4-hydroxycoumarin or lawsone) was obtained commercially. The experimental procedure for the synthesis of **2** followed the methodology reported in the literature and is described in the Supporting Information.^21,22^

### General Procedure for the Synthesis of **3a–w**

Aryldiazonium salts **2** (0.5 mmol) and TiO_2_ (0.125 mmol) were added to a round-bottom flask containing 4-hydroxycoumarin or lawsone **1** (0.5 mmol) and 10 mL of ethanol. The reaction mixture was stirred at room temperature for 30 min. After the reaction time, the mixture was filtered and the solids were extracted with dichloromethane (3 × 20 mL). The organic extract was washed with water (3 × 20 mL) to remove residuals of TiO_2_ and then dried with Na_2_SO_4_. The solids were obtained after the solvent was removed under reduced pressure. The compounds **3a**–**y** were purified by column chromatography using a hexane/ethyl acetate gradient.

### General Procedure for the Synthesis of **7a–r**

Diazonium salts **2** (1 mmol) and TiO_2_ (0.5 mmol) were added to a round-bottom flask containing cyclic 1,3-dicarbonyl compound (**6**, 1 mmol) and 5 mL of H_2_O. The reaction mixture was stirred at room temperature for 24 h. After the reaction time, the mixture was filtered, and the solids were extracted with dichloromethane (3 × 20 mL). The organic extract was washed with water (3 × 20 mL) to remove residual TiO_2_ and then dried with Na_2_SO_4_. The solids were obtained after the solvent was removed under reduced pressure. The compounds were purified by column chromatography using a hexane/ethyl acetate gradient.

### (*E*)-3-(2-(4-Chlorophenyl)hydrazono)chromane-2,4-dione (**3a**).^1^

(121.5 mg, 81% yield), yellow solid. Melting point: 97–98 °C. IR υ_max_ (ATR) cm^–1^: 3358, 1729, 1615, 1590, 830. ^1^H NMR (500 MHz, CDCl_3_, δ) 8.09 (d, *J* = 7.8 Hz, 1H), 7.68 (t, *J* = 7.8 Hz, 1H), 7.63 (d, *J* = 8.7 Hz, 2H), 7.45 (d, *J* = 8.7 Hz, 2H), 7.35–7.28 (m, 2H). ^13^C{^1^H} NMR (125 MHz, CDCl_3_, δ) 178.8, 159.0, 154.6, 139.2, 136.6, 134.2, 130.2, 127.0, 124.9, 122.7, 120.3, 119.3, 117.8.

### (*E*)-3-(2-(4-Nitrophenyl)hydrazono)chromane-2,4-dione (**3b**).^1^

(123.5 mg, 79% yield), yellow solid. Melting point: 110–112 °C. IR υ_max_ (ATR) cm^–1^: 3118, 1725, 1629, 1538, 1318. ^1^H NMR (500 MHz, CDCl_3_, δ) 8.36 (d, *J* = 9.1 Hz, 2H), 8.11 (dd, *J* = 7.9, 1.6 Hz, 1H), 7.79 (d, *J* = 9.1 Hz, 2H), 7.75–7.69 (m, 1H), 7.39–7.30 (m, 2H). ^13^C{^1^H} NMR (125 MHz, CDCl_3_, δ) 179.4, 158.2, 154.8, 146.4, 145.4, 137.4, 136.5, 127.4, 125.9, 125.2, 124.5, 120.1, 118.0.

### (*E*)-3-(2-(4-Bromophenyl)hydrazono)chromane-2,4-dione (**3c**).^1^

(74.5 mg, 43% yield), yellow solid. Melting point: 200–201 °C. IR υ_max_ (ATR) cm^–1^: 3052, 1727, 1624, 818, 735. ^1^H NMR (500 MHz, CDCl_3_, δ) 8.08 (d, *J* = 7.6 Hz, 1H), 7.68 (t, *J* = 7.3 Hz, 1H), 7.61 (d, *J* = 8.8 Hz, 2H), 7.56 (d, *J* = 8.8 Hz, 2H), 7.38–7.28 (m, 2H). ^13^C{^1^H} NMR (125 MHz, CDCl_3_, δ) 178.8, 158.9, 154.6, 139.7, 136.6, 133.1, 127.0, 124.8, 122.7, 122.0, 120.2, 119.5, 117.8.

### (*E*)-3-(2-(3-Chlorophenyl)hydrazono)chromane-2,4-dione (**3d**)

(50.4 mg, 34% yield), yellow solid. Melting point: 111–112 °C (dec.). IR υ_max_ (ATR) cm^–1^: 3102, 1739, 1622, 882, 791. ^1^H NMR (500 MHz, CDCl_3_, δ) 8.09 (dd, *J* = 7.9, 1.4 Hz, 1H), 7.75 (t, *J* = 2.0 Hz, 1H), 7.69 (ddd, *J* = 8.3, 7.3, 1.7 Hz, 1H), 7.51 (ddd, *J* = 8.1, 2.1, 0.9 Hz, 1H), 7.41 (t, *J* = 8.0 Hz, 1H), 7.36–7.29 (m, 3H). ^13^C{^1^H} NMR (125 MHz, CDCl_3_, δ) 179.0, 158.8, 154.7, 141.8, 136.7, 136.2, 131.0, 128.4, 127.1, 124.9, 120.3, 118.0, 117.8, 116.4. HRMS (ESI): for C_15_H_9_ClN_2_NaO_3_ [M + Na]^+^ 323.0199; found, 323.0203.

### (*E*)-3-(2-(2,4-Dichlorophenyl)hydrazono)chromane-2,4-dione (**3e**).^1^

(142.8 mg, 85% yield), yellow solid. Melting point: 199–200 °C. IR υ_max_ (ATR) cm^–1^: 3072, 1740, 1584, 899, 829. ^1^H NMR (500 MHz, CDCl_3_, δ) 8.15–8.07 (m, 2H), 7.69 (ddd, *J* = 8.7, 7.3, 1.7 Hz, 1H), 7.49 (d, *J* = 2.2 Hz, 1H), 7.39 (dd, *J* = 8.9, 2.2 Hz, 1H), 7.36–7.28 (m, 2H). ^13^C{^1^H} NMR (125 MHz, CDCl_3_, δ) 178.7, 158.7, 154.6, 136.9, 136.5, 133.7, 129.8, 129.1, 127.3, 125.0, 124.2, 124.0, 120.2, 119.2, 117.8.

### (*E*)-3-(2-(2,5-Dichlorophenyl)hydrazono)chromane-2,4-dione (**3f**).^9^

(92.1 mg, 55% yield), yellow solid. Melting point: 201–202 °C. IR υ_max_ (ATR) cm^–1^: 3092, 1742, 1623, 892, 817. ^1^H NMR (500 MHz, CDCl_3_, δ) 8.14 (d, *J* = 2.4 Hz, 1H), 8.13 (dd, *J* = 7.9, 1.7 Hz, 1H), 7.70 (ddd, *J* = 8.8, 7.3, 1.7 Hz, 1H), 7.41 (d, *J* = 8.6 Hz, 1H), 7.37–7.29 (m, 2H), 7.23 (dd, *J* = 8.6, 2.4 Hz, 1H). ^13^C{^1^H} NMR (125 MHz, CDCl_3_, δ) 178.9, 158.5, 154.6, 138.5, 137.0, 135.0, 130.9, 128.4, 127.3, 125.0, 124.3, 121.7, 120.2, 118.2, 117.9.

### (*E*)-3-(2-(3,4-Dichlorophenyl)hydrazono)chromane-2,4-dione (**3g**)

(99.8 mg, 60% yield), yellow solid. Melting point: 197–198 °C. IR υ_max_ (ATR) cm^–1^: 3069, 1726, 1623, 897, 824. ^1^H NMR (500 MHz, CDCl_3_, δ) 8.09 (dd, *J* = 7.9, 1.7 Hz, 1H), 7.83 (d, *J* = 2.5 Hz, 1H), 7.74–7.67 (m, 1H), 7.55 (d, *J* = 8.7 Hz, 1H), 7.49 (dd, *J* = 8.7, 2.5 Hz, 1H), 7.37–7.28 (m, 2H). ^13^C{^1^H} NMR (125 MHz, CDCl_3_, δ) 179.1, 158.6, 154.7, 140.0, 136.9, 134.5, 132.1, 131.6, 127.1, 125.0, 123.2, 120.1, 119.5, 117.9, 117.2. HRMS (ESI): for C_15_H_8_Cl_2_N_2_NaO_3_ [M + Na]^+^ 356.9810; found, 356.9813.

### (*E*)-3-(2-(3,5-Dichlorophenyl)hydrazono)chromane-2,4-dione (**3h**)

(70.6 mg, 42% yield), yellow solid. Melting point: 181–182 °C. IR υ_max_ (ATR) cm^–1^: 3071, 1736, 1664, 1583, 893. ^1^H NMR (500 MHz, DMSO-*d*_6_, δ): 8.00 (d, *J* = 7.5 Hz, 1H), 7.87–7.76 (m, 3H), 7.53 (s, 1H), 7.43–7.35 (m, 2H). ^13^C{^1^H} NMR (125 MHz, CDCl_3_, δ) 179.2, 158.4, 154.7, 142.4, 137.1, 136.7, 127.9, 127.2, 125.0, 120.1, 117.9, 116.4. HRMS (ESI): for C_15_H_8_Cl_2_N_2_NaO_3_ [M + Na]^+^ 356.9810; found, 356.9810.

### (*E*)-3-(2-(4-Bromo-3-methylphenyl)hydrazono)chromane-2,4-dione (**3i**).^23^

(54.6 mg, 30% yield), pale orange solid. Melting point: 167–168 °C. IR υ_max_ (ATR) cm^–1^: 2951, 1731, 1626, 897, 812. ^1^H NMR (500 MHz, CDCl_3_, δ) 8.06 (dd, *J* = 7.9, 1.6 Hz, 1H), 7.72–7.63 (m, 1H), 7.58 (dd, *J* = 16.5, 5.5 Hz, 2H), 7.38–7.21 (m, 3H), 2.45 (s, 3H). ^13^C{^1^H} NMR (125 MHz, CDCl_3_, δ) 178.7, 159.0, 154.5, 140.2, 139.7, 136.5, 133.7, 126.9, 124.8, 124.7, 122.6, 120.3, 119.82, 119.80, 117.7, 117.0, 23.1.

### (*E*)-3-(2-(*p*-Tolyl)hydrazono)chromane-2,4-dione (**3j**).^1^

(22.7 mg, 16% yield), yellow solid. Melting point: 148–150 °C. IR υ_max_ (ATR) cm^–1^: 2922, 1734, 1605, 1578, 815. ^1^H NMR (500 MHz, CDCl_3_, δ) 8.00 (d, *J* = 7.8 Hz, 1H), 7.58 (t, *J* = 7.7 Hz, 1H), 7.51 (d, *J* = 8.2 Hz, 2H), 7.32–7.18 (m, 4H), 2.33 (s, 3H). ^13^C{^1^H} NMR (125 MHz, CDCl_3_, δ) 178.3, 159.3, 154.4, 139.2, 138.3, 136.1, 130.5, 126.7, 124.6, 121.9, 120.3, 118.2, 117.6, 21.3.

### (*E*)-3-(2-(4-Methoxyphenyl)hydrazono)chromane-2,4-dione (**3k**).^1^

(92.6 mg, 63% yield), orange solid. Melting point: 144–145 °C. IR υ_max_ (ATR) cm^–1^: 2941, 1731, 1255, 1075, 830. ^1^H NMR (500 MHz, CDCl_3_, δ) 8.00 (dd, *J* = 7.8, 1.7 Hz, 1H), 7.64–7.52 (m, 3H), 7.26–7.18 (m, 2H), 6.92 (d, *J* = 9.0 Hz, 2H), 3.79 (s, 3H). ^13^C{^1^H} NMR (125 MHz, CDCl_3_, δ) 178.1, 160.3, 154.4, 135.9, 135.4, 134.1, 126.6, 124.6, 120.4, 119.9, 117.6, 115.3, 55.8.

### (*E*)-3-(2-Phenylhydrazono)chromane-2,4-dione (**3l**).^1^

(54.8 mg, 41% yield), yellow solid. Melting point: 151–152 °C. IR υ_max_ (ATR) cm^–1^: 2918, 1736, 1615, 756, 732. ^1^H NMR (500 MHz, CDCl_3_, δ) 8.08 (dd, *J* = 7.8, 1.7 Hz, 1H), 7.70–7.65 (m, 3H), 7.48 (dd, *J* = 8.5, 7.4 Hz, 2H), 7.37–7.27 (m, 3H). ^13^C{^1^H} NMR (125 MHz, CDCl_3_, δ) 178.6, 159.2, 154.5, 140.6, 136.3, 130.0, 128.6, 126.9, 124.7, 122.4, 120.3, 118.2, 117.7.

### (*E*)-3-(2-(4-Chlorophenyl)hydrazono)naphthalene-1,2,4(3*H*)-trione (**3m**).^24^

(98.2 mg, 59% yield), red solid. Melting point: 178–180 °C (dec.). IR υ_max_ (ATR) cm^–1^: 3096, 1674, 1586, 1085, 839. ^1^H NMR (500 MHz, DMSO-*d*_6_, δ): 8.16 (d, *J* = 54.5 Hz, 2H), 7.92 (d, *J* = 27.9 Hz, 2H), 7.80 (s, 2H), 7.58 (d, *J* = 5.7 Hz, 2H). ^13^C{^1^H} NMR (125 MHz, CDCl_3_, δ) 181.8, 180.3, 179.7, 135.7, 134.5, 134.1, 130.0, 129.9, 129.2, 128.8, 128.6, 128.1, 128.0, 127.7, 118.7, 118.3.

### (*E*)-3-(2-(4-Nitrophenyl)hydrazono)naphthalene-1,2,4(3*H*)-trione (**3n**).^25^

(121.0 mg, 75% yield), orange solid. Melting point: 148–149 °C (dec.). IR υ_max_ (ATR) cm^–1^: 3107, 1698, 1666, 1529, 1313. ^1^H NMR (500 MHz, DMSO, δ): 8.36 (d, *J* = 9.1 Hz, 1H), 8.24 (d, *J* = 7.6 Hz, 1H), 8.14 (dd, *J* = 7.5, 1.5 Hz, 1H), 7.99–7.88 (m, 4H). ^13^C{^1^H} NMR (125 MHz, CDCl_3_, δ) 179.4, 136.3, 136.2, 135.5, 134.9, 129.4, 128.7, 128.6, 128.3, 125.9, 125.9, 118.6, 118.2.

### (*E*)-3-(2-(4-Bromophenyl)hydrazono)naphthalene-1,2,4(3*H*)-trione (**3o**).^26^

(108.6 mg, 61% yield), orange solid. Melting point: 178–180 °C. IR υ_max_ (ATR) cm^–1^: 3104, 1691, 1662, 1575, 835. ^1^H NMR (500 MHz, CDCl_3_, δ) 8.43 (dd, *J* = 7.9, 1.3 Hz, 1H), 8.33–8.24 (m, 1H), 7.90 (tdd, *J* = 7.6, 2.5, 1.4 Hz, 1H), 7.82 (tdd, *J* = 7.5, 3.2, 1.3 Hz, 1H), 7.66–7.56 (m, 4H). ^13^C{^1^H} NMR (125 MHz, CDCl_3_, δ) 212.8, 192.0, 191.7, 185.8, 183.1, 178.4, 177.7, 177.4, 172.5, 170.1, 169.8, 168.8, 164.6, 157.2, 151.7.

### (*E*)-3-(2-(3-Chlorophenyl)hydrazono)naphthalene-1,2,4(3*H*)-trione (**3p**)

(54.1 mg, 35% yield), red solid. Melting point: 163–164 °C. IR υ_max_ (ATR) cm^–1^: 1691, 1668, 881, 793, 691. ^1^H NMR (500 MHz, DMSO-*d*_6_, δ): 8.22 (dd, *J* = 9.2, 8.7 Hz, 1H), 8.12 (d, *J* = 7.5 Hz, 1H), 8.02–7.82 (m, 3H), 7.74 (dd, *J* = 11.3, 9.0 Hz, 1H), 7.53 (t, *J* = 8.0 Hz, 1H), 7.36 (d, *J* = 7.7 Hz, 1H). ^13^C{^1^H} NMR (125 MHz, CDCl_3_, δ) 182.2, 180.0, 178.8, 136.1, 136.0, 135.0, 134.5, 132.6, 131.1, 129.0, 128.4, 128.1, 118.5, 118.3, 117.0, 116.5. HRMS (ESI): for C_16_H_9_ClN_2_NaO_3_ [M + Na]^+^ 335.0200; found, 335.0186.

### (*E*)-3-(2-(2,4-Dichlorophenyl)hydrazono)naphthalene-1,2,4(3*H*)-trione (**3q**)

(146.8 mg, 84% yield), red solid. Melting point: 191–192 °C. IR υ_max_ (ATR) cm^–1^: 3097, 1706, 1682, 876, 822. ^1^H NMR (500 MHz, DMSO-*d*_6_, δ): 8.24 (t, *J* = 6.2 Hz, 1H), 8.17–8.07 (m, 1H), 7.99–7.80 (m, 4H), 7.64 (td, *J* = 8.7, 2.1 Hz, 1H). ^13^C{^1^H} NMR (125 MHz, CDCl_3_, δ) 179.8, 178.8, 175.5, 136.1, 136.1, 135.1, 134.6, 130.0, 129.8, 129.2, 129.1, 128.5, 128.4, 128.2, 119.8, 119.5. HRMS (ESI): for C_16_H_9_Cl_2_N_2_NaO_3_ [M + Na]^+^ 368.9800; found, 368.9812.

### (*E*)-3-(2-(2,5-Dichlorophenyl)hydrazono)naphthalene-1,2,4(3*H*)-trione (**3r**)

(150.0 mg, 86% yield), red solid. Melting point: 178–179 °C. IR υ_max_ (ATR) cm^–1^: 3095, 1698, 1678, 870, 803. ^1^H NMR (500 MHz, DMSO, δ): 8.25 (t, *J* = 6.6 Hz, 1H), 8.17–8.12 (m, 1H), 7.99–7.85 (m, 4H), 7.64 (t, *J* = 8.3 Hz, 1H). ^13^C{^1^H} NMR (125 MHz, CDCl_3_, δ) 184.9, 181.9, 156.3, 135.9, 135.3, 133.1, 130.9, 130.8, 129.1, 128.8, 128.4, 126.7, 126.5, 118.6, 118.3, 110.7. HRMS (ESI): for C_16_H_9_Cl_2_N_2_NaO_3_ [M + Na]^+^ 368.9800; found, 368.9786.

### (*E*)-3-(2-(3,4-Dichlorophenyl)hydrazono)naphthalene-1,2,4(3*H*)-trione (**3s**)

(124.5 mg, 72% yield), red solid. Melting point: 134–135 °C. IR υ_max_ (ATR) cm^–1^: 3105, 1689, 1670, 867, 833. ^1^H NMR (500 MHz, CDCl_3_, δ) 8.31–8.21 (m, 1H), 7.99–7.89 (m, 2H), 7.84 (tdd, *J* = 7.5, 3.6, 1.3 Hz, 2H), 7.64–7.50 (m, 3H). ^13^C{^1^H} NMR (125 MHz, CDCl_3_, δ) 184.8, 181.8, 156.1, 135.1, 134.8, 134.3, 133.0, 132.8, 131.5, 129.3, 128.9, 128.2, 127.9, 126.6, 126.4, 110.5. HRMS (ESI): for C_16_H_9_Cl_2_N_2_NaO_3_ [M + Na]^+^ 368.9800; found, 368.9802.

### (*E*)-3-(2-(3,5-Dichlorophenyl)hydrazono)naphthalene-1,2,4(3*H*)-trione (**3t**)

(122.9 mg, 71% yield), red solid. Melting point: 237–238 °C. IR υ_max_ (ATR) cm^–1^: 3057, 1692, 1659, 1574, 874. ^1^H NMR (500 MHz, DMSO, δ): 8.21 (t, *J* = 6.8 Hz, 1H), 8.12 (dd, *J* = 7.6, 1.4 Hz, 1H), 8.03 (d, *J* = 12.5 Hz, 1H), 7.94 (d, *J* = 7.4 Hz, 1H), 7.89 (t, *J* = 7.4 Hz, 1H), 7.81–7.72 (m, 2H). ^13^C{^1^H} NMR (125 MHz, CDCl_3_, δ) 181.6, 175.5, 173.0, 144.3, 134.9, 134.5, 129.6, 127.7, 127.2, 125.3, 116.0. HRMS (ESI): for C_16_H_9_Cl_2_N_2_NaO_3_ [M + Na]^+^ 368.9800; found, 368.9792.

### (*E*)-3-(2-(*p*-Tolyl)hydrazono)naphthalene-1,2,4(3*H*)-trione (**3u**)

(95.1 mg, 65% yield), red solid. Melting point: 205–206 °C. IR υ_max_ (ATR) cm^–1^: 3064, 1674, 1634, 1578, 805. ^1^H NMR (500 MHz, CDCl_3_, δ) 8.51–8.29 (m, 1H), 8.18–8.10 (m, 1H), 7.89–7.76 (m, 1H), 7.71 (t, *J* = 7.5 Hz, 1H), 7.53 (dd, *J* = 18.5, 8.4 Hz, 2H), 7.30–7.13 (m, 2H), 2.32 (d, *J* = 6.7 Hz, 3H). ^13^C{^1^H} NMR (125 MHz, CDCl_3_, δ) 185.0, 182.0, 174.6, 156.5, 140.2, 135.4, 134.1, 133.2, 130.8, 130.6, 128.1, 126.8, 126.6, 118.9, 118.5, 110.8, 21.5. HRMS (ESI): for C_17_H_12_N_2_NaO_3_ [M + Na]^+^ 315.0700; found, 315.0724.

### (*E*)-3-(2-(4-Methoxyphenyl)hydrazono)naphthalene-1,2,4(3*H*)-trione (**3v**).^25^

(109.3 mg, 71% yield), brown solid. Melting point: 138–139 °C (dec.). IR υ_max_ (ATR) cm^–1^: 3139, 1674, 1635, 1257, 1023. ^1^H NMR (500 MHz, DMSO, δ): 8.22 (t, *J* = 9.0 Hz, 1H), 8.10 (d, *J* = 7.6 Hz, 1H), 7.94 (q, *J* = 7.4 Hz, 1H), 7.87 (td, *J* = 7.5, 1.3 Hz, 1H), 7.76 (dd, *J* = 13.1, 8.7 Hz, 2H), 7.12 (dd, *J* = 8.9, 4.1 Hz, 2H), 3.83 (s, 3H). ^13^C{^1^H} NMR (125 MHz, CDCl_3_, δ) 185.1, 182.0, 156.4, 135.7, 135.4, 133.2, 133.0, 129.5, 126.8, 126.6, 120.7, 120.2, 115.5, 115.4, 110.8, 55.9.

### (*E*)-3-(2-Phenylhydrazono)naphthalene-1,2,4(3*H*)-trione (**3w**).^25^

(86.3 mg, 62% yield), orange solid. Melting point: 187–188 °C. IR υ_max_ (ATR) cm^–1^: 3058, 1698, 1667, 732, 690. ^1^H NMR (500 MHz, CDCl_3_, δ) 8.43 (dd, *J* = 7.8, 1.3 Hz, 1H), 8.33–8.24 (m, 1H), 7.89 (tt, *J* = 7.6, 1.6 Hz, 1H), 7.81 (tt, *J* = 7.5, 1.5 Hz, 1H), 7.76–7.69 (m, 2H), 7.51 (td, *J* = 9.0, 7.3 Hz, 2H), 7.43–7.35 (m, 1H). ^13^C{^1^H} NMR (125 MHz, CDCl_3_, δ) 181.8, 180.3, 174.9, 135.8, 135.7, 134.5, 134.1, 130.0, 129.9, 129.2, 128.9, 128.7, 128.2, 128.1, 127.8, 118.7, 118.4.

### S6 Characterization of Compounds **7a–r**

#### 2-(2-(3,5-Dimethoxyphenyl)hydrazono)-5,5-dimethylcyclohexane-1,3-dione (**7a**)

(85.2 mg, 28% yield), yellow solid, Melting point: 122–124 °C. IR υ_max_ (ATR) cm^–1^: 2957, 2918, 2849, 1677, 1485. ^1^H NMR (500 MHz, CDCl_3_, δ) 15.28 (s, 1H), 6.68 (d, *J* = 1.3 Hz, 2H), 6.32 (s, 2H), 3.80 (s, 6H), 2.58 (d, *J* = 3.0 Hz, 4H), 1.11 (s, 6H), 7.43–7.34 (m, 2H). ^13^C{^1^H} NMR (126 MHz, CDCl_3_, δ) 197.1, 193.4, 161.7, 142.9, 130.1, 99.9, 95.7, 55.6, 52.6, 52.5, 29.7, 28.5. HRMS (ESI): for C_16_H_20_N_2_NaO_4_ [M + Na]^+^ 327.1321; found, 327.1310.

#### 2-(2-(4-Chlorophenyl)hydrazono)-5,5-dimethylcyclohexane-1,3-dione (**7b**).^27^

(197.9 mg, 71% yield), yellow solid. Melting point: 215–217 °C. IR υ_max_ (ATR) cm^–1^: 3053, 2951, 1671, 1584, 854. ^1^H NMR (500 MHz, CDCl_3_, δ) 15.33 (s, 1H), 7.47 (d, *J* = 8.8 Hz, 2H), 7.36 (d, *J* = 8.9 Hz, 2H), 2.59 (d, *J* = 1.7 Hz, 3H), 1.11 (s, 6H). ^13^C{^1^H} NMR (126 MHz, CDCl_3_, δ): 197.4, 193.3, 139.8, 132.5, 130.5, 129.9, 118.6, 52.7, 52.6, 30.8, 28.6.

#### 5,5-Dimethyl-2-(2-(4-nitrophenyl)hydrazono)cyclohexane-1,3-dione (**7c**).^27^

(225.6 mg, 78% yield), yellow solid. Melting point: 210–212 °C. IR υ_max_ (ATR) cm^–1^: 3114, 2959, 1697, 1497, 824. ^1^H NMR (500 MHz, CDCl_3_, δ) 8.29 (d, *J* = 9.1 Hz, 2H), 7.64 (d, *J* = 9.2 Hz, 2H), 2.66 (d, *J* = 3.5 Hz, 4H), 1.15 (s, 6H). ^13^C{^1^H} NMR (126 MHz, CDCl_3_, δ) 197.1, 192.0, 145.2, 144.3, 130.7, 124.6, 116.1, 51.8, 51.7, 29.6, 27.5.

#### 2-(2-(4-Methoxyphenyl)hydrazono)-5,5-dimethylcyclohexane-1,3-dione (7**d**).^27^

(192.0 mg, 70% yield), yellow solid. Melting point: 129–131 °C. IR υ_max_ (ATR) cm^–1^: 2929, 2837, 1669, 1588, 814. ^1^H NMR (500 MHz, CDCl_3_, δ) 15.66 (s, 1H), 7.48 (d, *J* = 6.7 Hz, 2H), 6.90 (d, *J* = 6.8 Hz, 2H), 3.79 (d, *J* = 2.1 Hz, 3H), 2.55 (s, 4H), 1.09 (d, *J* = 2.3 Hz, 6H). ^13^C{^1^H} NMR (126 MHz, CDCl_3_, δ) 196.6, 193.4, 159.1, 134.5, 129.8, 119.1, 114.9, 55.6, 52.3, 30.8, 28.5.

#### 5,5-Dimethyl-2-(2-(*p*-tolyl)hydrazono)cyclohexane-1,3-dione (7**e**).^27^

(175.6 mg, 68% yield), yellow solid. Melting point: 153–156 °C. IR υ_max_ (ATR) cm^–1^: 3033, 2947, 1669, 1588, 808. ^1^H NMR (500 MHz, CDCl_3_, δ) 15.50 (s, 1H), 7.43 (d, *J* = 8.5 Hz, 2H), 7.19 (d, *J* = 8.2 Hz, 2H), 2.57 (s, 4H), 2.34 (s, 3H), 1.11 (s, 6H). ^13^C{^1^H} NMR (126 MHz, CDCl_3_, δ) 196.8, 193.4, 138.7, 130.2, 130.0, 117.5, 52.5, 52.4, 30.8, 28.5, 21.1.

#### 5,5-Dimethyl-2-(2-phenylhydrazono)cyclohexane-1,3-dione (7**f**).^27^

(180.8 mg, 70% yield), yellow solid. Melting point: 134–137 °C. IR υ_max_ (ATR) cm^–1^: 3052, 1671, 1589, 747, 688. ^1^H NMR (500 MHz, CDCl_3_, δ) 15.42 (s, 1H), 7.56 (d, *J* = 7.7 Hz, 2H), 7.42 (dd, *J* = 8.5, 7.3 Hz, 2H), 7.29–7.23 (m, 1H), 2.62 (s, 4H), 1.14 (s, 6H). ^13^C{^1^H} NMR (126 MHz, CDCl_3_, δ) 197.0, 193.4, 141.0, 130.3, 129.6, 127.2, 117.5, 52.6, 52.5, 30.7, 28.5.

#### 2-(2-(3,5-Dimethoxyphenyl)hydrazono)-5,5-dimethylcyclohexane-1,3-dione (**7g**)

(92.4 mg, 30% yield), yellow solid. Melting point: 129–132 °C. IR υ_max_ (ATR) cm^–1^: 2986, 2946, 1750, 1597, 891. ^1^H NMR (500 MHz, CDCl_3_, δ) 13.55 (s, 1H), 6.66 (d, *J* = 2.2 Hz, 2H), 6.35 (t, *J* = 2.2 Hz, 1H), 3.81 (s, 6H), 1.79 (s, 6H). ^13^C{^1^H} NMR (126 MHz, CDCl_3_, δ) 161.8, 161.0, 159.1, 142.2, 112.3, 105.8, 100.0, 95.5, 55.7, 27.5. HRMS (ESI): for C_14_H_16_N_2_NaO_6_ [M + Na]^+^ 331.0906; found, 331.0892.

#### 5-(2-(4-Chlorophenyl)hydrazono)-2,2-dimethyl-1,3-dioxane-4,6-dione (**7h**).^27^

(191.7 mg, 68% yield), yellow solid. Melting point: 126–128 °C. IR υ_max_ (ATR) cm^–1^: 3097, 2990, 1747, 1591, 802. ^1^H NMR (500 MHz, CDCl_3_, δ) 13.63 (s, 1H), 7.48 (d, *J* = 8.8 Hz, 2H), 7.41 (d, *J* = 8.8 Hz, 2H), 1.81 (s, 6H). ^13^C{^1^H} NMR (126 MHz, CDCl_3_, δ) 160.9, 159.0, 139.0, 132.9, 130.0, 118.3, 112.8, 106.0, 27.5.

#### 2,2-Dimethyl-5-(2-(4-nitrophenyl)hydrazono)-1,3-dioxane-4,6-dione (**7i**).^27^

(234.5 mg, 80% yield), yellow solid. Melting point: 192–195 °C. IR υ_max_ (ATR) cm^–1^: 3163, 1754, 1525, 1333, 800. ^1^H NMR (500 MHz, CDCl_3_, δ) 13.58 (s, 1H), 8.33 (d, *J* = 9.1 Hz, 2H), 7.66 (d, *J* = 9.1 Hz, 2H), 1.84 (s, 6H). ^13^C{^1^H} NMR (126 MHz, CDCl_3_, δ) 160.5, 158.2, 145.8, 145.1, 125.8, 117.1, 115.3, 106.5, 27.7.

#### 5-(2-(4-Methoxyphenyl)hydrazono)-2,2-dimethyl-1,3-dioxane-4,6-dione (**7j**).^27^

(192.0 mg, 69% yield), yellow solid. Melting point: 127–129 °C. IR υ_max_ (ATR) cm^–1^: 3128, 1744, 1275, 1024, 804. ^1^H NMR (500 MHz, CDCl_3_, δ) 13.77 (s, 1H), 7.47 (d, *J* = 9.1 Hz, 2H), 6.93 (d, *J* = 9.2 Hz, 2H), 3.82 (s, 3H), 1.78 (s, 6H). ^13^C{^1^H} NMR (126 MHz, CDCl_3_, δ) 161.3, 159.6, 159.3, 134.0, 118.7, 115.0, 111.2, 105.6, 55.6, 27.4.

#### 2,2-Dimethyl-5-(2-(*p*-tolyl)hydrazono)-1,3-dioxane-4,6-dione (**7k**).^27^

(125.8 mg, 48% yield), red solid. Melting point: 148–150 °C. IR υ_max_ (ATR) cm^–1^: 3126, 2992, 1738, 1596, 821. ^1^H NMR (500 MHz, CDCl_3_, δ) 13.68 (s, 1H), 7.41 (d, *J* = 1.2 Hz, 2H), 7.22 (d, *J* = 8.5 Hz, 2H), 2.36 (s, 3H), 1.78 (s, 6H). ^13^C{^1^H} NMR (126 MHz, CDCl_3_, δ) 161.1, 159.4, 138.2, 137.9, 130.3, 117.2, 111.7, 105.7, 27.4, 21.1.

#### 2,2-Dimethyl-5-(2-phenylhydrazono)-1,3-dioxane-4,6-dione (**7l**).^27^

(123.2 mg, 47% yield), yellow solid. Melting point: 170–172 °C. IR υ_max_ (ATR) cm^–1^: 3141, 2993, 1741, 1597, 890. ^1^H NMR (500 MHz, CDCl_3_, δ) 13.67 (s, 1H), 7.54 (dd, *J* = 8.7, 1.2 Hz, 2H), 7.47–7.42 (m, 2H), 7.32–7.27 (m, 1H), 1.81 (s, 6H). ^13^C{^1^H} NMR (126 MHz, CDCl_3_, δ) 161.0, 159.2, 140.4, 129.8, 127.5, 117.2, 112.3, 105.8, 27.5.

#### 2-(2-(3,5-Dimethoxyphenyl)hydrazono)-1*H*-indene-1,3(2*H*)-dione (**7m**)

(152.0 mg, 49% yield), brown solid. Melting point: 208–210 °C. IR υ_max_ (ATR) cm^–1^: 3177, 2919, 1715, 1589, 886. ^1^H NMR (500 MHz, CDCl_3_, δ) 13.40 (s, 1H), 7.97–7.92 (m, 1H), 7.90–7.86 (m, 1H), 7.81–7.73 (m, 2H), 6.67 (d, *J* = 2.2 Hz, 2H), 6.32 (t, *J* = 2.2 Hz, 1H), 3.84 (s, 6H). ^13^C{^1^H} NMR (126 MHz, CDCl_3_, δ) 188.8, 186.2, 161.8, 142.8, 140.6, 138.8, 135.3, 135.0, 130.7, 123.2, 122.9, 99.0, 94.6, 55.7. HRMS (ESI): for C_17_H_14_N_2_NaO_4_ [M + Na]^+^ 333,0851; found, 333.0855.

#### 2-(2-(4-Chlorophenyl)hydrazono)-1*H*-indene-1,3(2*H*)-dione (**7n**).^28^

(202.1 mg, 71% yield), yellow solid. Melting point: 196–198 °C. IR υ_max_ (ATR) cm^–1^: 3157, 1714, 1589, 825, 809. ^1^H NMR (500 MHz, CDCl_3_, δ) 13.41 (s, 1H), 7.95 (dd, *J* = 6.4, 2.4 Hz, 1H), 7.89 (dd, *J* = 6.7, 1.4 Hz, 1H), 7.78 (ddd, *J* = 6.4, 4.3, 1.5 Hz, 2H), 7.46 (d, *J* = 8.9 Hz, 2H), 7.38 (d, *J* = 8.9 Hz, 2H). ^13^C{^1^H} NMR (126 MHz, CDCl_3_, δ) 188.7, 185.9, 140.7, 139.7, 138.8, 135.5, 135.1, 131.4, 131.2, 129.8, 123.3, 123.0, 117.4.

#### 2-(2-(4-Nitrophenyl)hydrazono)-1*H*-indene-1,3(2*H*)-dione (**7o**).^29^

(212.5 mg, 72% yield), yellow solid. Melting point 297–298 °C(dec). IR υ_max_ (ATR) cm^–1^: 3154, 3096, 1719, 1540, 1333. ^1^H NMR (500 MHz, CDCl_3_, δ) 13.37 (s, 1H), 8.31 (d, *J* = 9.3 Hz, 2H), 8.03–8.01 (m, 1H), 7.98–7.95 (m, 1H), 7.89–7.82 (m, 2H), 7.62 (d, *J* = 9.1 Hz, 2H). ^13^C{^1^H} NMR (126 MHz, CDCl_3_, δ) 188.5, 185.4, 146.2, 144.8, 141.2, 139.0, 136.2, 135.7, 125.8, 123.7, 123.5, 115.8.

#### 2-(2-(4-Methoxyphenyl)hydrazono)-1*H*-indene-1,3(2*H*)-dione (**7p**).^30^

(204.6 mg, 73% yield), orange solid. Melting point: 200–202 °C. IR υ_max_ (ATR) cm^–1^: 3152, 1708, 1215, 1021, 809. ^1^H NMR (500 MHz, DMSO, δ): 8.28 (d, *J* = 1.7 Hz, 4H), 8.01 (d, *J* = 9.0 Hz, 2H), 7.46 (d, *J* = 9.0 Hz, 2H), 4.21 (s, 3H). ^13^C{^1^H} NMR (126 MHz, DMSO, δ): 186.7, 185.8, 157.7, 139.8, 138.5, 135.3, 135.3, 135.1, 129.6, 122.5, 118.0, 114.9, 55.5.

#### 2-(2-(*p*-Tolyl)hydrazono)-1*H*-indene-1,3(2*H*)-dione (**7q**)

(161.2 mg, 61% yield), yellow solid. Melting point: 196–199 °C. IR υ_max_ (ATR) cm^–1^: 3155, 1713, 1666, 1590, 807. ^1^H NMR (500 MHz, CDCl_3_, δ) 13.48 (s, 1H), 7.93–7.88 (m, 1H), 7.85–7.80 (m, 1H), 7.73 (td, *J* = 4.2, 1.9 Hz, 2H), 7.40 (d, *J* = 8.2 Hz, 2H), 7.19 (d, *J* = 8.1 Hz, 2H), 2.34 (s, 3H). ^13^C{^1^H} NMR (126 MHz, CDCl_3_, δ) 188.7, 186.3, 140.5, 138.7, 138.7, 136.5, 135.1, 134.8, 130.2, 123.1, 122.7, 116.3, 21.1.

#### 2-(2-Phenylhydrazono)-1*H*-indene-1,3(2*H*)-dione (**7r**)

(132.8 mg, 45% yield), yellow solid. Melting point: 186–188 °C. IR υ_max_ (ATR) cm^–1^: 3047, 1716, 1591, 739, 701. ^1^H NMR (500 MHz, CDCl_3_, δ) 13.43 (s, 1H), 7.95–7.90 (m, 1H), 7.86–7.82 (m, 1H), 7.74 (tt, *J* = 7.3, 5.8 Hz, 2H), 7.50 (d, *J* = 7.9 Hz, 2H), 7.40 (t, *J* = 7.9 Hz, 2H), 7.20 (t, *J* = 7.4 Hz, 1H). ^13^C{^1^H} NMR (126 MHz, CDCl_3_, δ) 188.7, 186.1, 141.0, 140.6, 138.7, 135.3, 134.9, 130.7, 129.6, 126.3, 123.2, 122.8, 116.3.

## Data Availability

The data underlying this study are available in the published article and its Supporting Information.

## Supporting Information Available

Details of the complete experimental procedures, computational details, characterization data, nuclear magnetic resonance spectra, and high-resolution mass spectrometry. The Supporting Information is available free of charge at https://pubs.acs.org/doi/10.1021/acs.joc.4c02266.
