# Supplementary material for: TiO2-Catalyzed Direct Diazenylation of Active Methylene Compounds with Diazonium Salts
Source: J Org Chem. 2024 Dec 18;90(1):300–8. doi: 10.1021/acs.joc.4c02266 (PMC11731305; doi:10.1021/acs.joc.4c02266)
Supplement: Supplementary file 1 — jo4c02266_si_001.pdf [file jo4c02266_si_001.pdf]

TiO_2-Catalyzed Direct Diazenylation of Active Methylene Compounds with Diazonium Salts

Edson Evangelista,[1] Yuri P. V. de Carvalho,[1] Iva S. de Jesus,[2] Maria Eduarda S. Rodrigues,[1] Emelli P. Hayashi,[1] Karine N. de Andrade,[1] Rodolfo G. Fiorot,[1] and Luana da S. M. Forezi[1,*]

[1] Department of Organic Chemistry, Institute of Chemistry, Federal Fluminense University – UFF, Niteroi, Rio de Janeiro, 24020-141, Brazil.

[2] Department of Pharmaceutical Technology, Federal Fluminense University – UFF, Niteroi, Rio de Janeiro, 24241-000, Brazil

Corresponding author: luanaforezi@id.uff.br

Contents

**S1.**	**General Procedure for the synthesis of aryldiazonium salts**	**S2**
**S2.**	**Scale-up Experiment for 3-aryl-4-Hydroxycoumarins**	**S2**
**S3.**	**Recycling of the catalyst experiment**	**S2**
**S4.**	**NMR spectral data and hight resolution mass spectral (HRMS) of the compounds 3 and 7**	**S3**
**S5.**	**Computational details**	**S97**

S1. General Procedure for the synthesis of aryldiazonium salts

In a 125 mL round bottom flask, 25 mmol of the aniline corresponding to the desired salt was added to 13 mL of 48% tetrafluoroboric acid w/w (HBF_4), in an ice bath and under magnetic stirring. After 10 minutes of stirring, a solution containing 25 mmol of $NaNO_2$ in 3 mL of previously, cooled water was slowly added into the reaction medium, with the help of an addition funnel. At this stage, a brown gas was released. As the reaction occurs, the desired salt precipitates in the reaction medium. At the end of the reaction time, this solid was filtered under vacuum and washed with 20 mL of ice-cold HBF_4 48% w/w, 20 mL of EtOH 96% w/w, and 40 mL of ice-cold ethyl ether.

S2. Scale-up Experiment for 3-aryl-4-Hydroxycoumarines

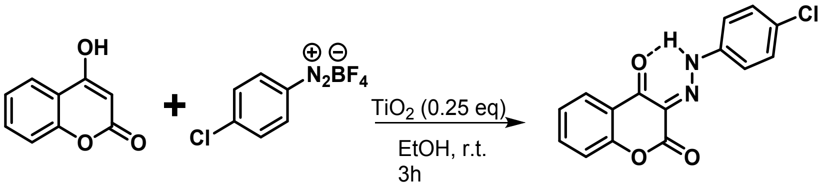

In a 50 mL round bottom flask, were added 0.8063 g (5 mmol) of 4-hydroxy-2H-chromen-2-one, 1.1657 g (5 mmol) of 1-(4-chlorophenyl)-2-(tetrafluoro-15-boranyl)diazene, 0.1067 g (1.34 mmol) of titanium(IV) oxide and 20 mL of EtOH. The round bottom flask was kept under agitation with the aid of a magnetic bar for 3 hours, at room temperature. The reaction was monitored by TLC (Hexane/Ethyl acetate 7:3) and after 3 hours, the mixture was filtered under vacuum. The yellow solid was dissolved in Dichloromethane (200 mL) and then filtered one more time to remove titanium (IV) oxide. The solution was dried over a vacuum to afford 1.113 g (74% yield) of (Z)-3-(2-(4-chlorophenyl)hydrazono)chromane-2,4-dione (**3a**).

S3. Recycling of the catalyst experiment

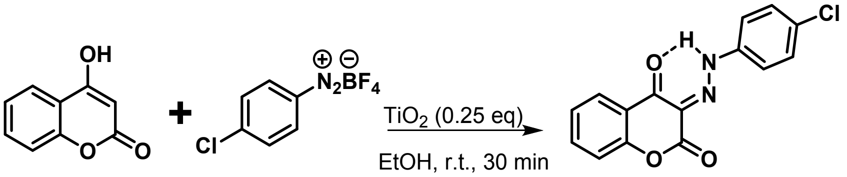

In a 20 ml centrifuge tube, were added 163.10 mg (1 mmol) of 4-hydroxy-2H-chromen-2-one, 230.03 mg (1 mmol) of 1-(4-chlorophenyl)-2-(tetrafluoro-15-boranyl)diazene, 20 mg (0.25 mmol) of titanium(IV) oxide and 10 ml of EtOH. The tube was sealed and kept under strong agitation with the aid of a magnetic bar. After the reaction time, the reaction was centrifuged and the solution was removed to a 50 ml flask, the titanium(IV) oxide present in the tube was washed with 10 ml of methanol and centrifuged again, the process was repeated 3X, and the solutions obtained from washing were added to the first fraction. The solvent was rotavaporated and the solid obtained was again solubilized in 20 ml of dichloromethane, the solution was subjected to extraction with 40 ml of water (2x) and the organic layer was dried with Na_2SO_4 and the solvent was removed under vacuum to obtain a yellow solid with 243.62 mg (81%) yield.

The procedure was repeated 4 times using the same tube and titanium oxide from the first reaction. After the isolation and purification steps, products with 231.54 mg (77%), 216.02 mg (72%), 210.49 mg (70%) and 204.48 mg (68%) were obtained.

S4. NMR spectral data and hight resolution mass spectral (HRMS) of the compounds 3 and 7

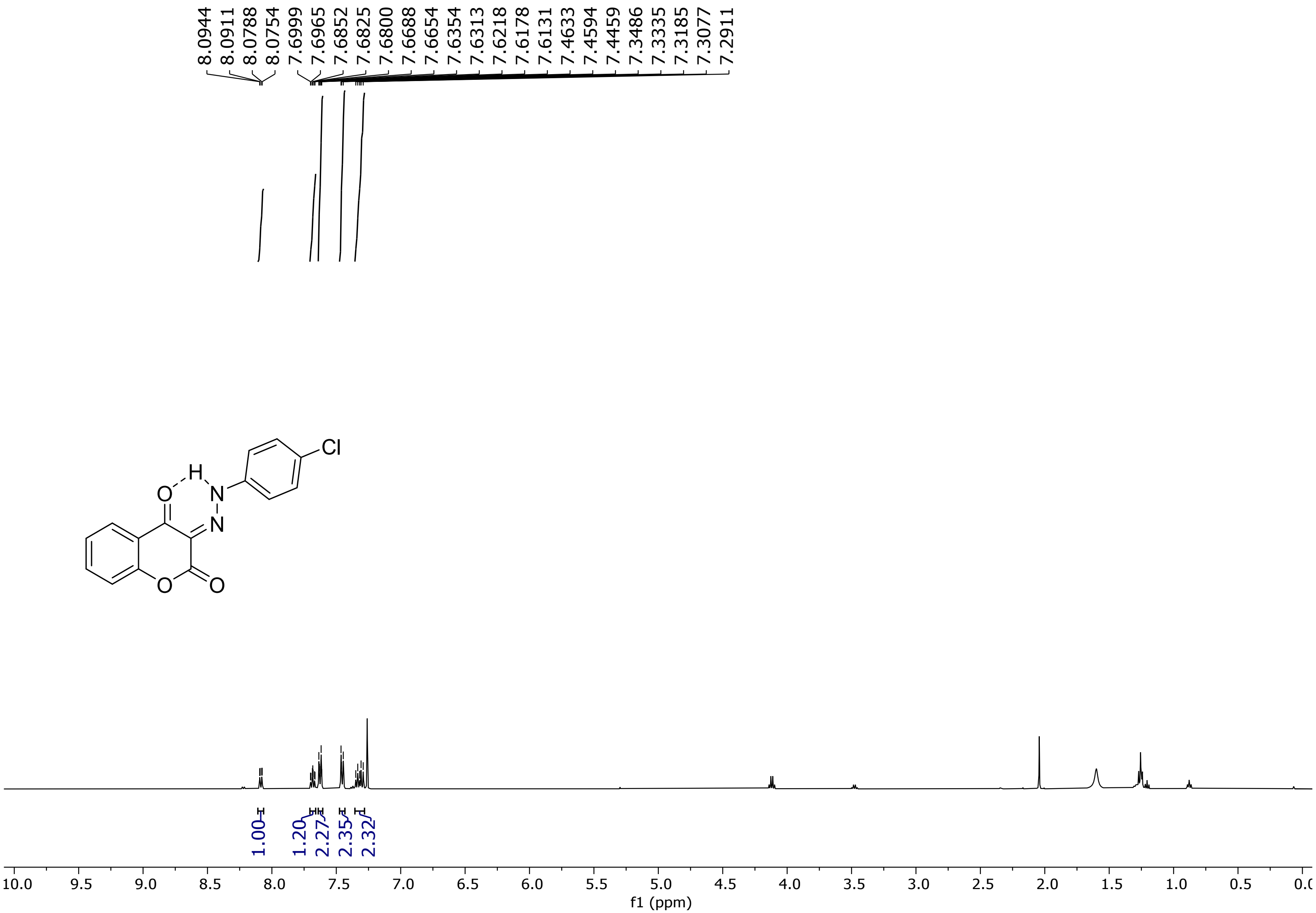

Figure S1. ^{1}H NMR spectrum of **3a** (500 MHz, $CDCl_3$)

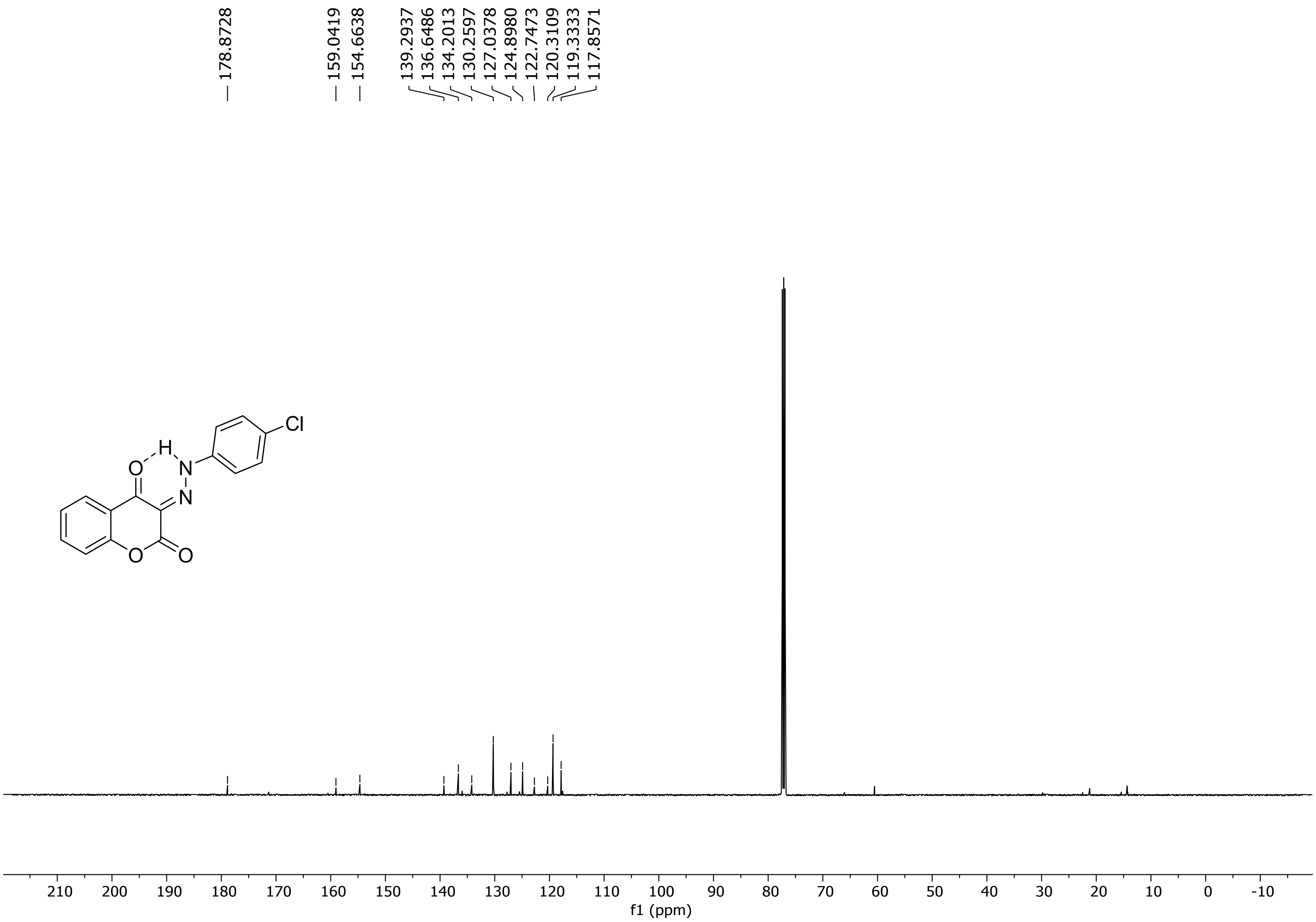

Figure S2. ^{13}C NMR spectrum of **3a** (125 MHz, $CDCl_3$)

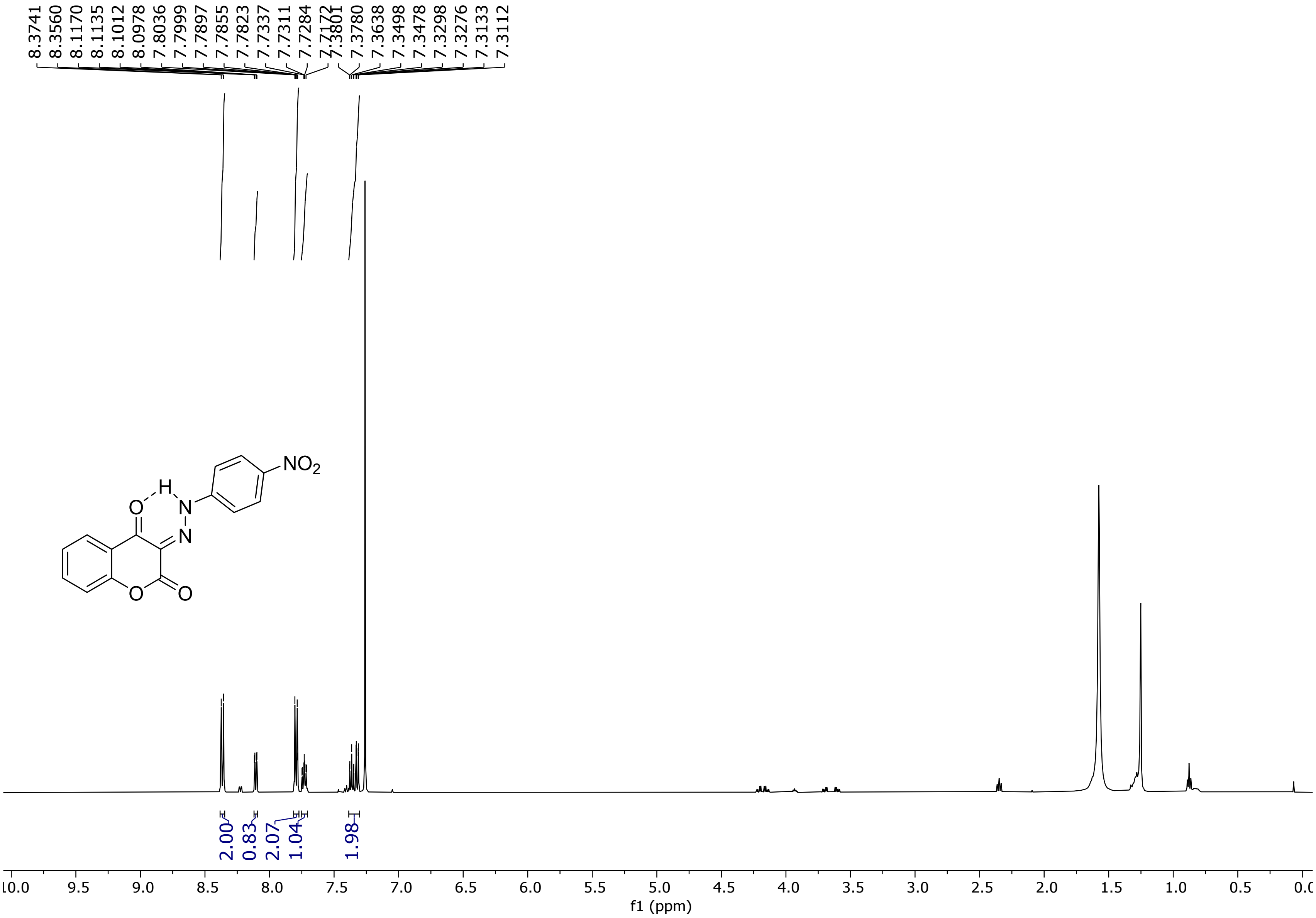

Figure S3. ^{1}H NMR spectrum of **3b** (500 MHz, $CDCl_3$)

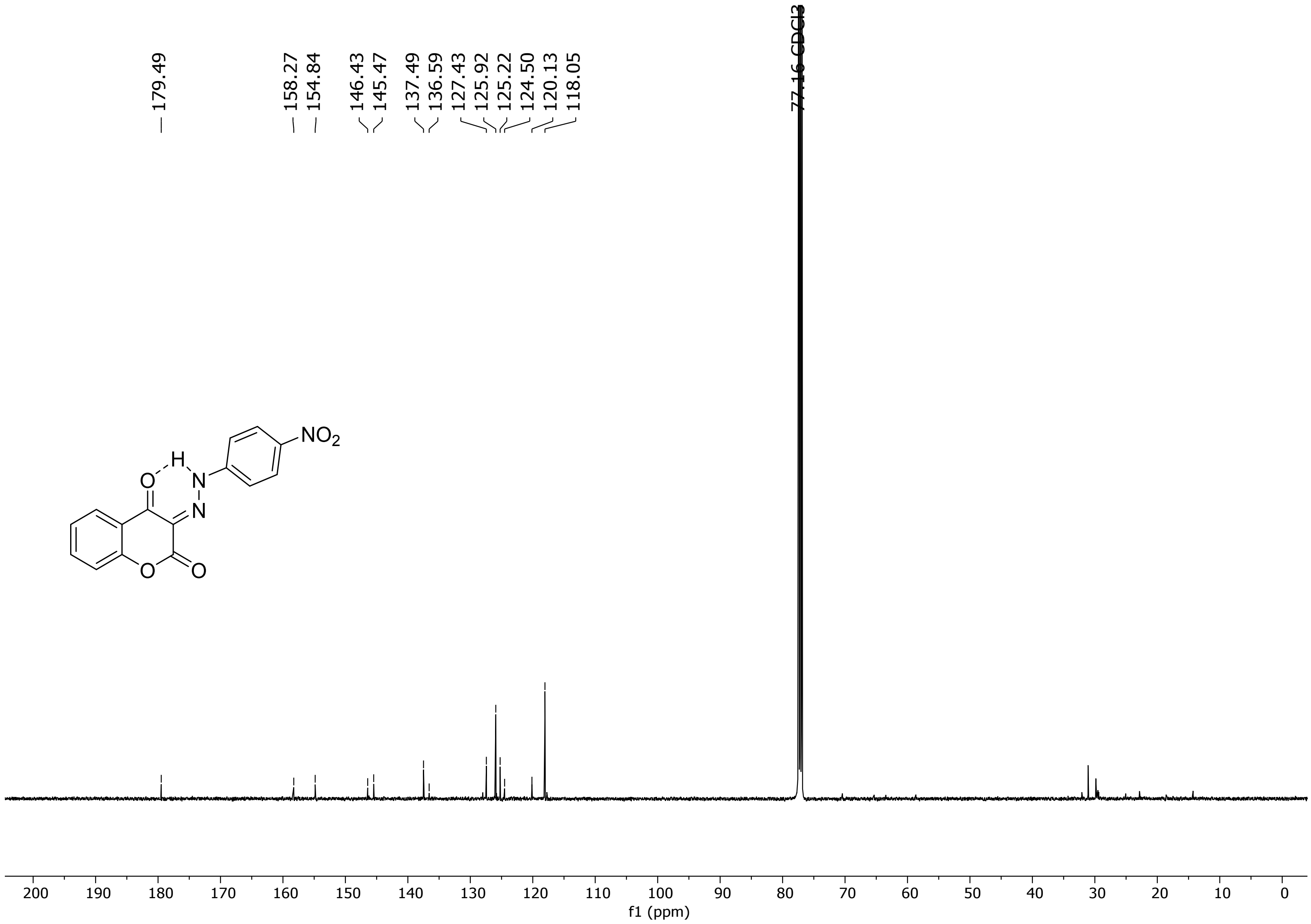

Figure S4. ^{13}C NMR spectrum of **3b** (125 MHz, $CDCl_3$)

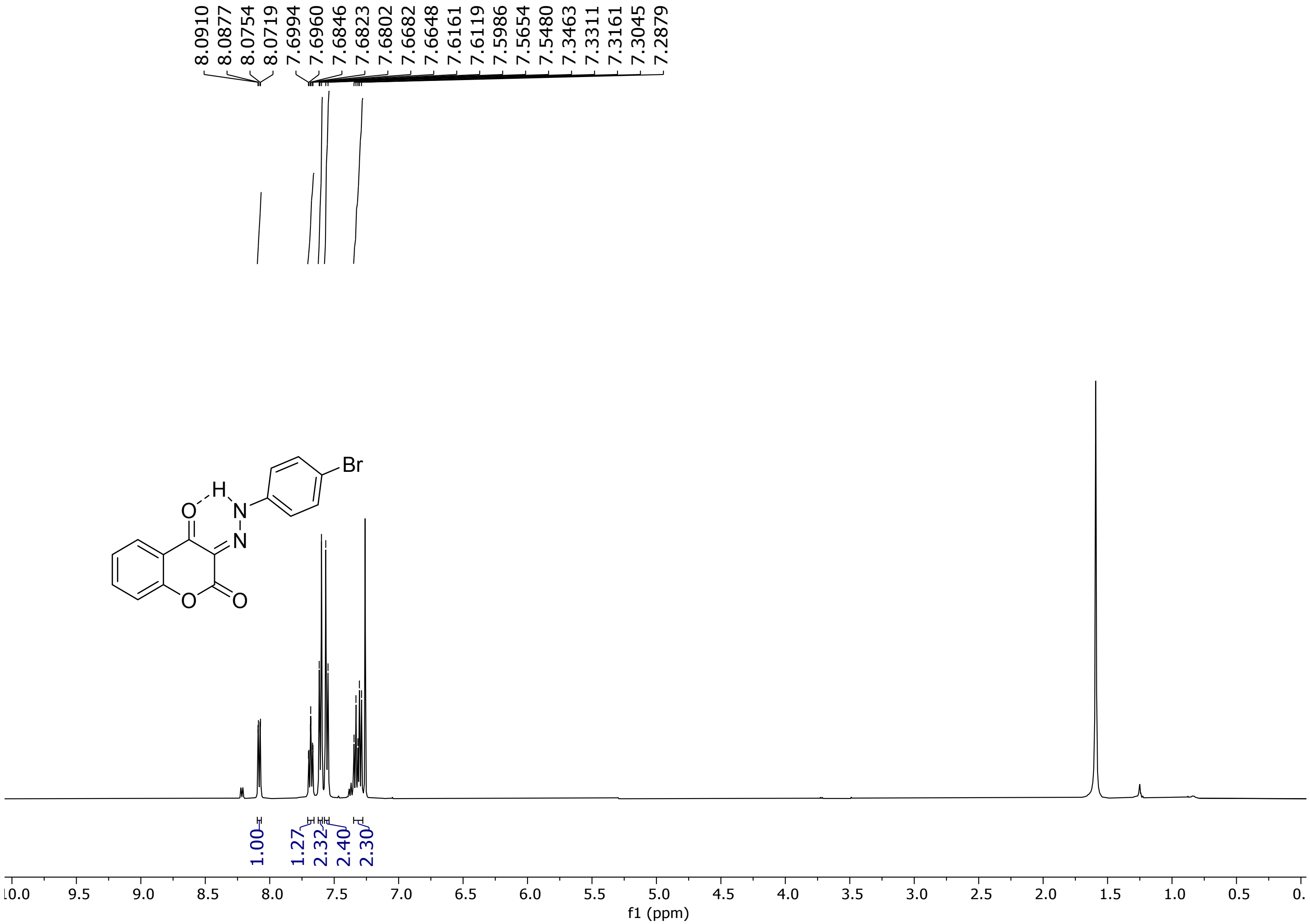

Figure S5.^{1}H NMR spectrum of **3c** (500 MHz, $CDCl_3$)

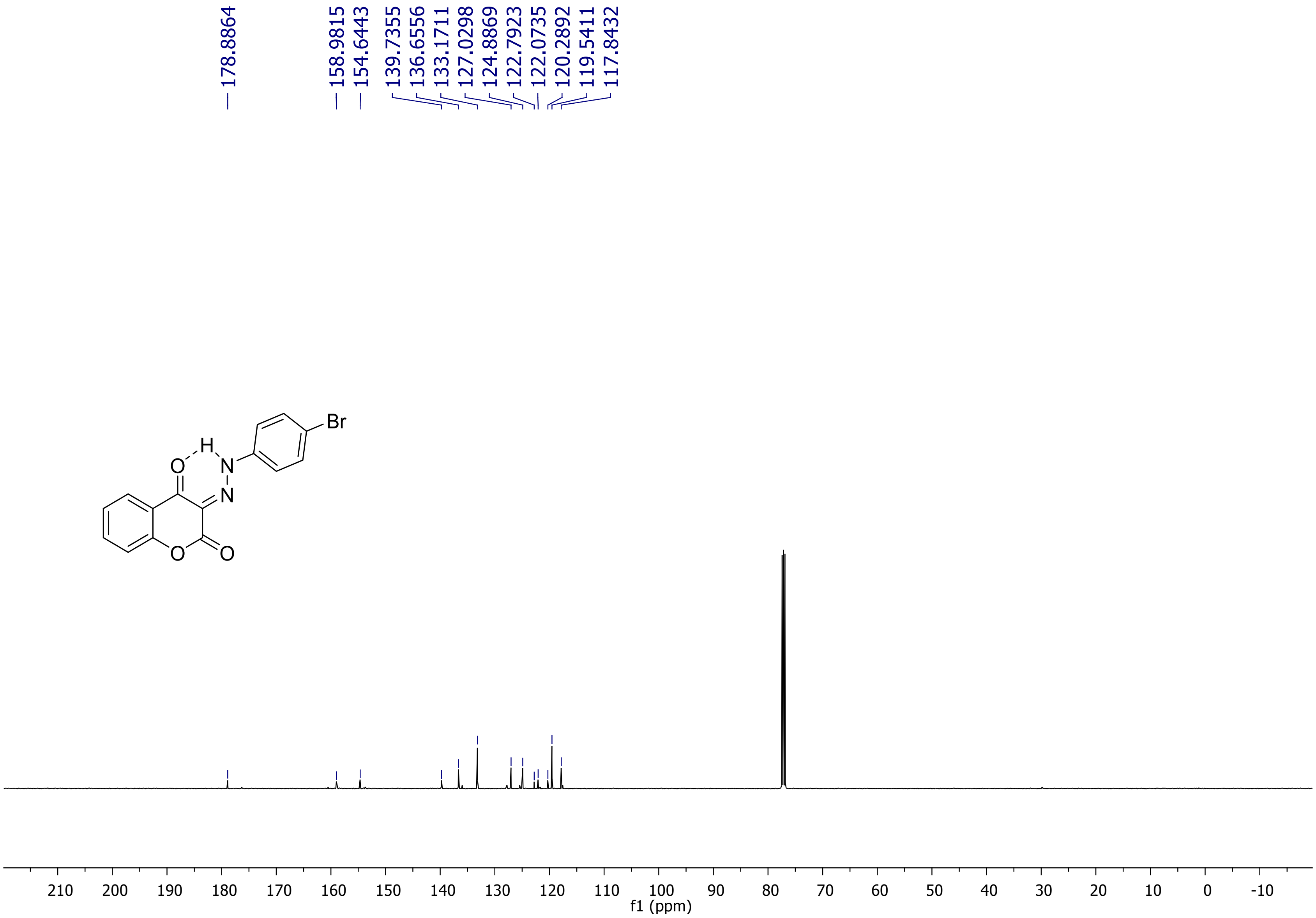

Figure S6. ^{13}C NMR spectrum of **3c** (125 MHz, $CDCl_3$)

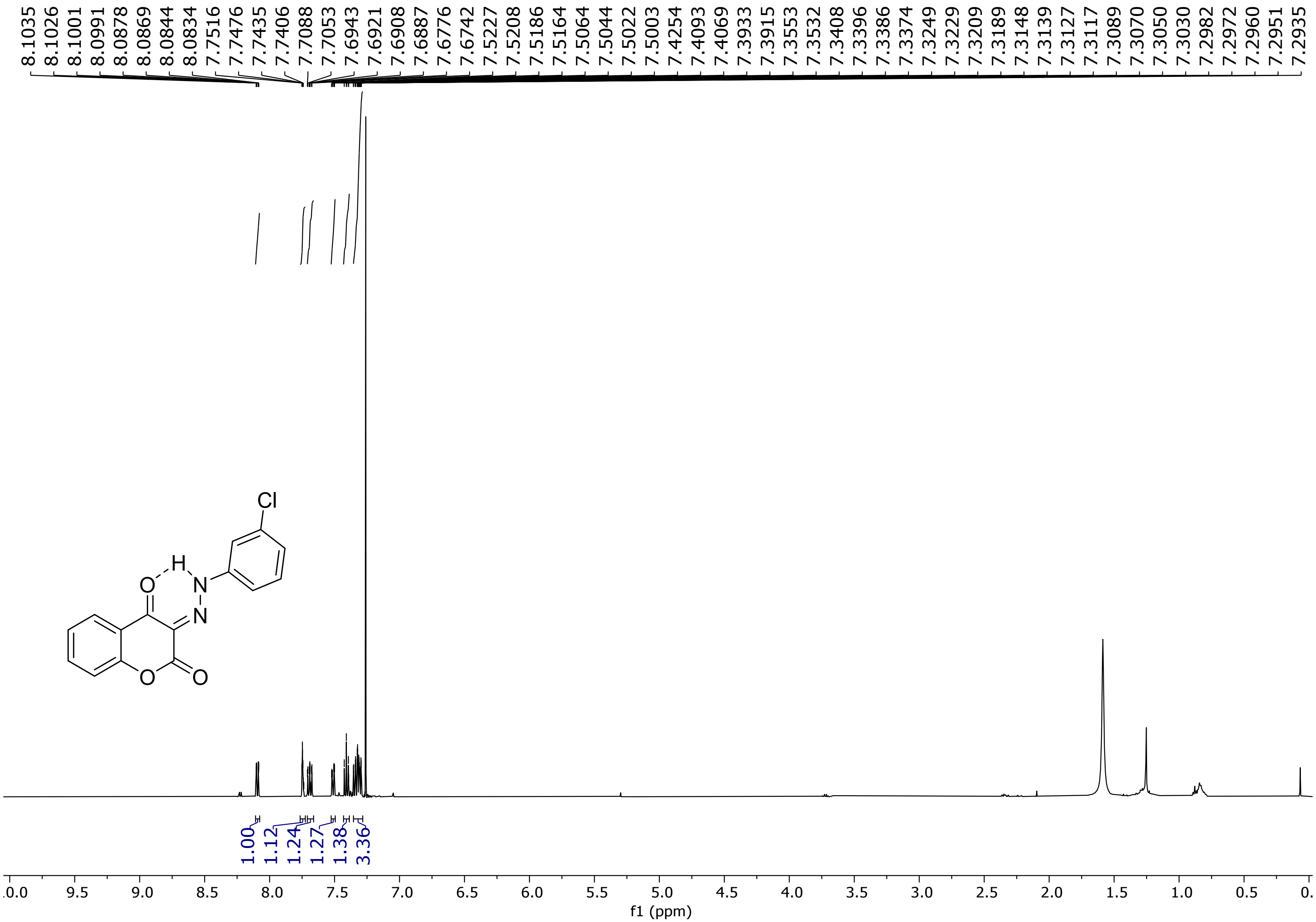

Figure S7. ^{1}H NMR spectrum of **3d** (500 MHz, $CDCl_3$)

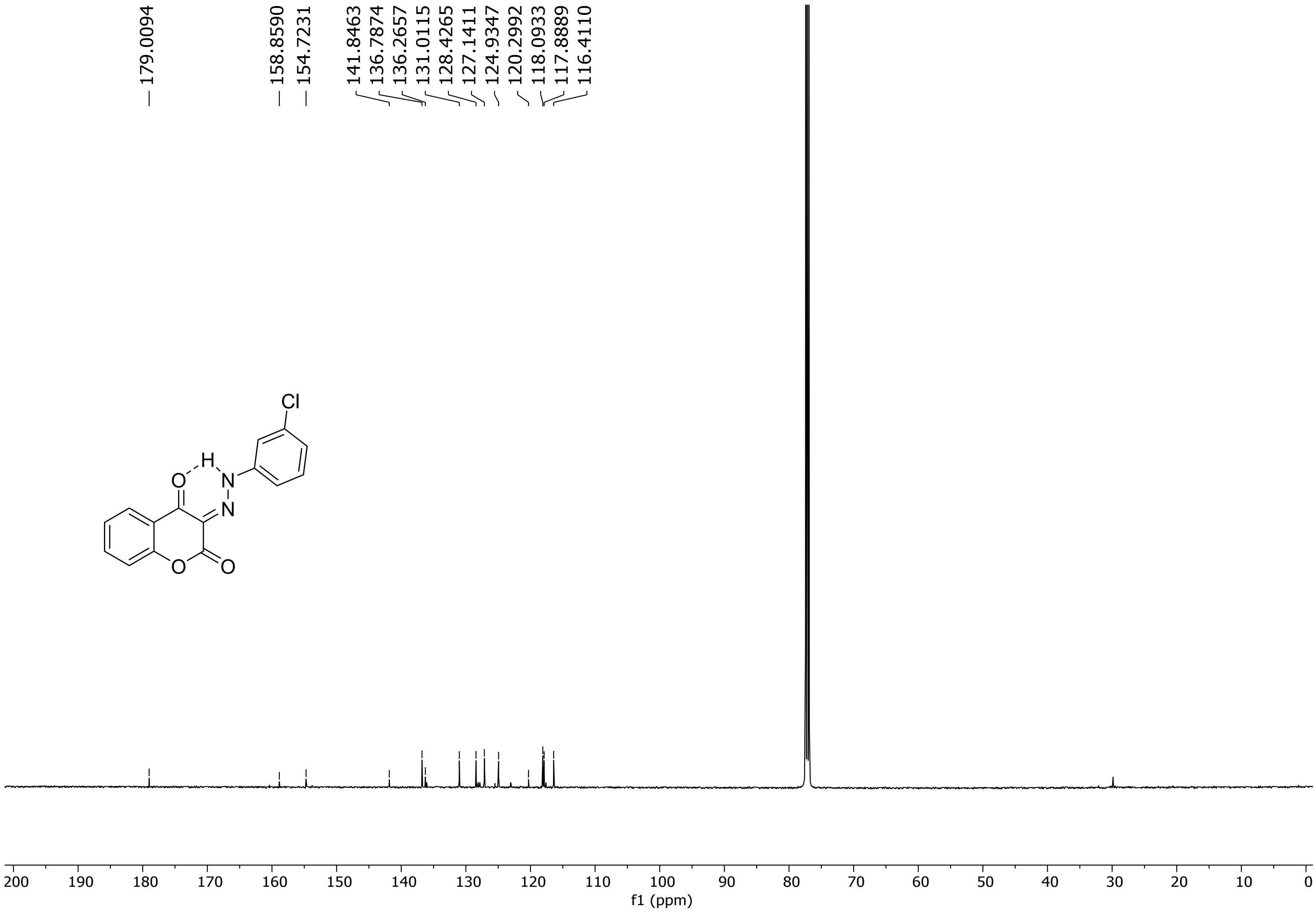

Figure S8. ^{13}C NMR spectrum of **3d** (125 MHz, $CDCl_3$)

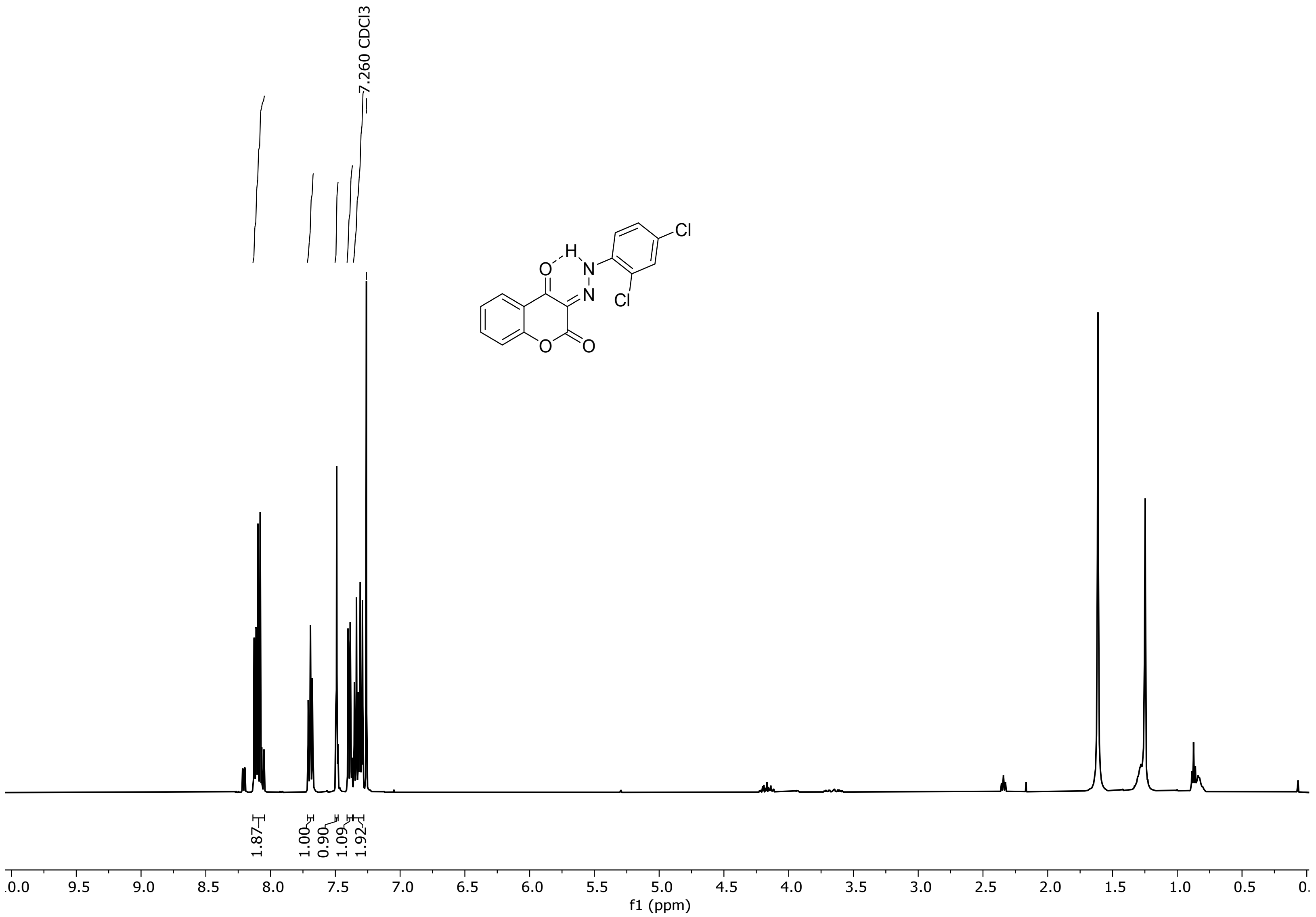

Figure S9. ^{1}H NMR spectrum of **3e** (500 MHz, $CDCl_3$)

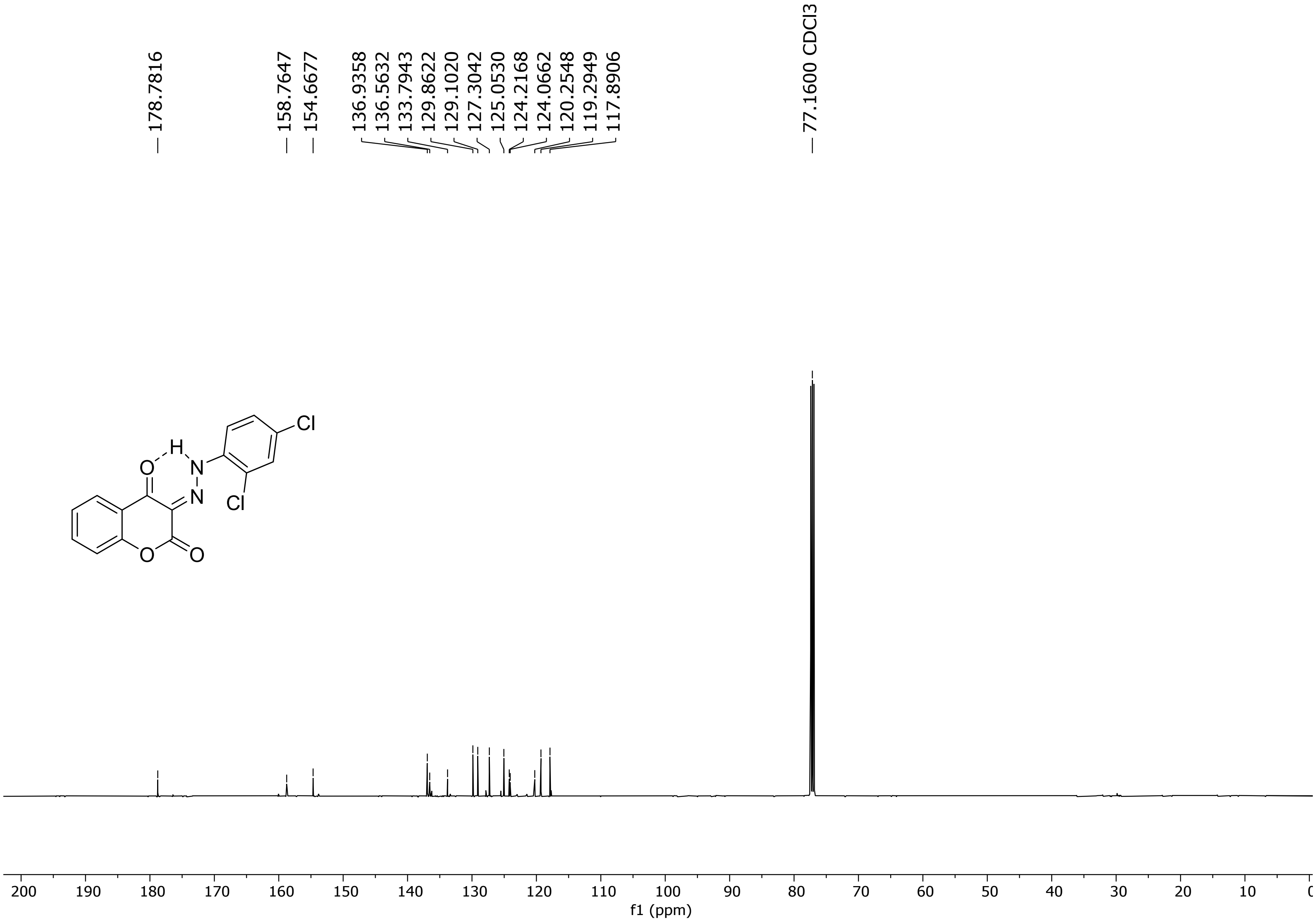

Figure S10. ^{13}C NMR spectrum of **3e** (125 MHz, $CDCl_3$)

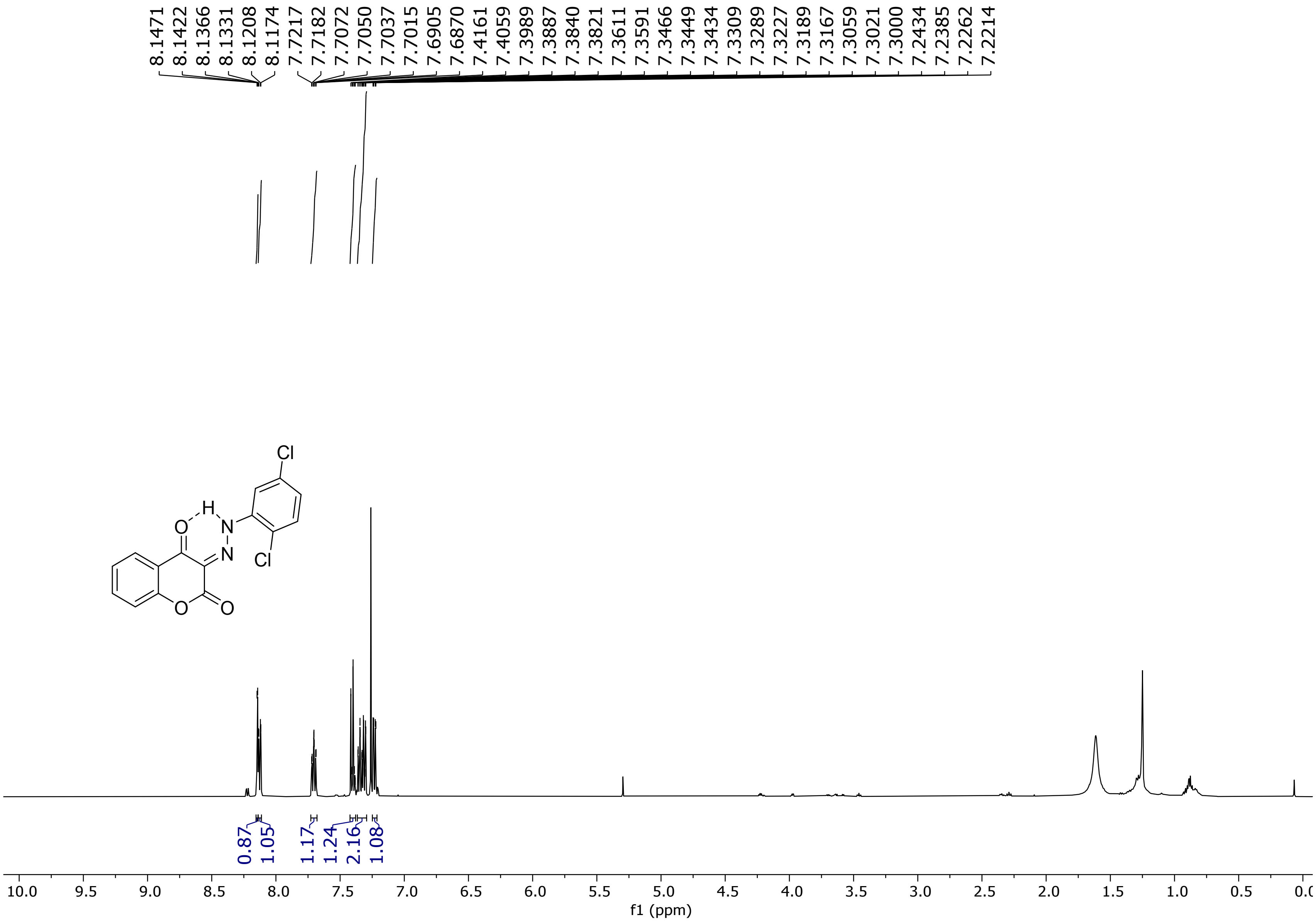

Figure S11. ^{1}H NMR spectrum of **3f** (500 MHz, $CDCl_3$)

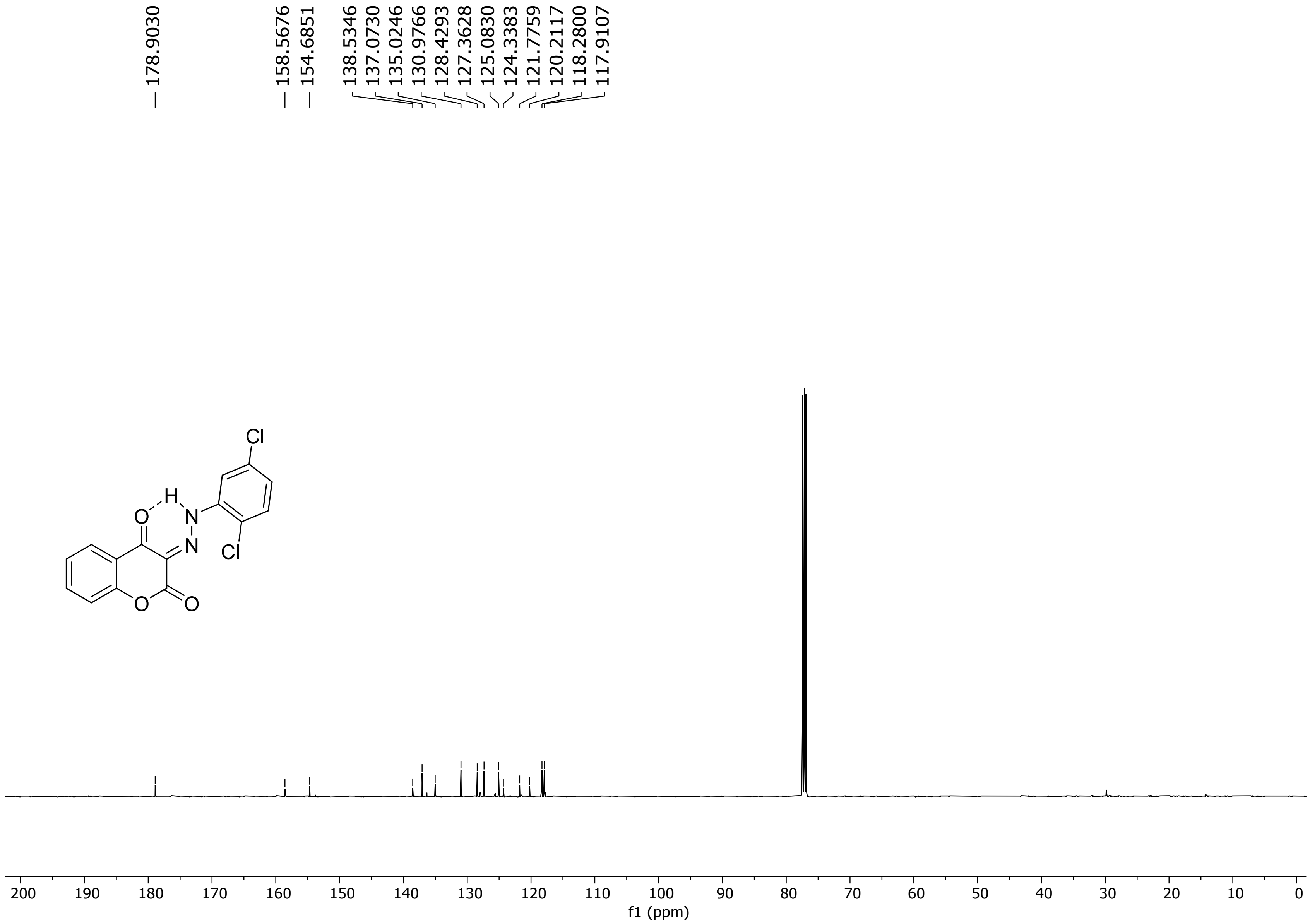

Figure S12. ^{13}C NMR spectrum of **3f** (125 MHz, $CDCl_3$)

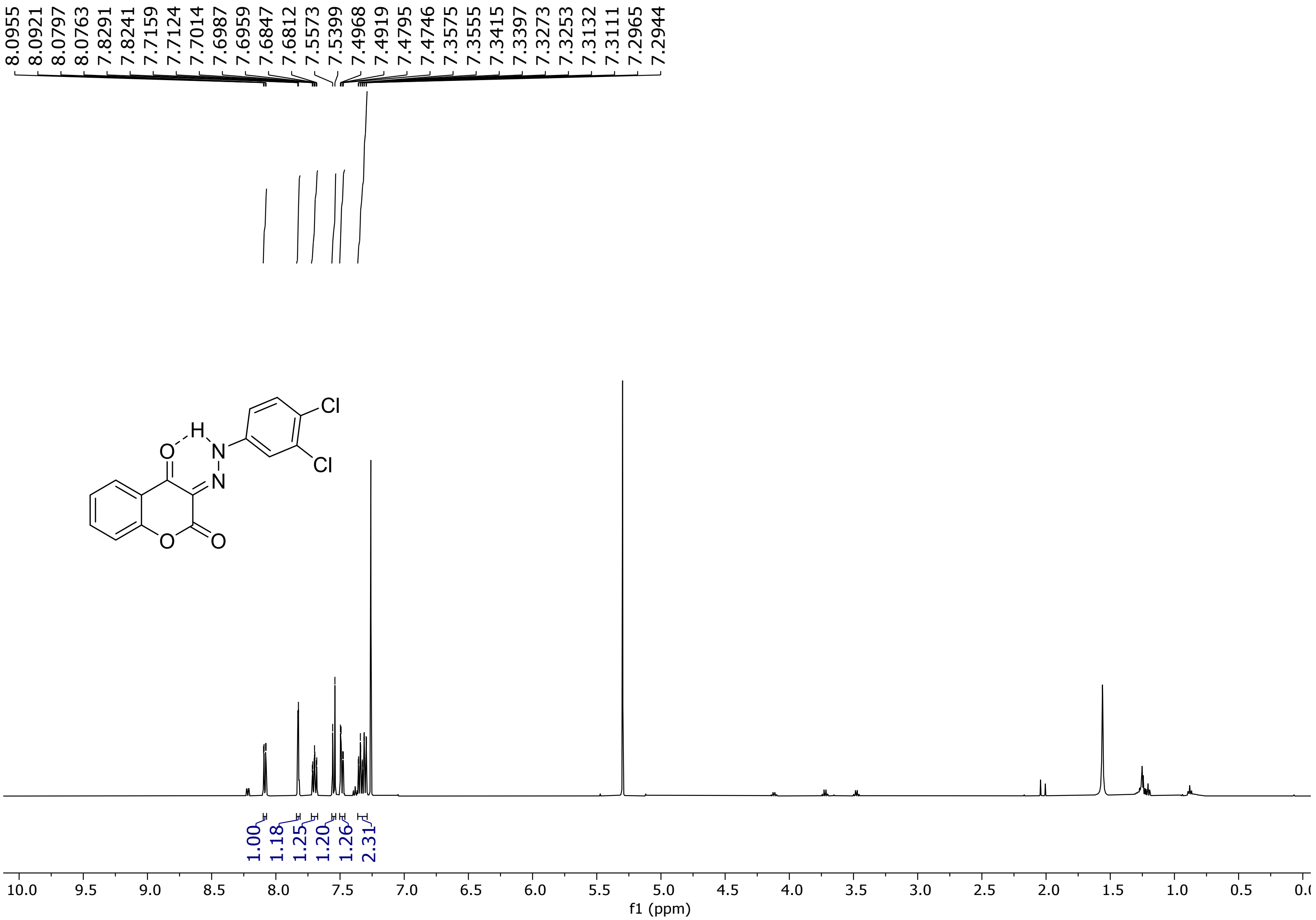

Figure S13. ^{1}H NMR spectrum of **3g** (500 MHz, $CDCl_3$)

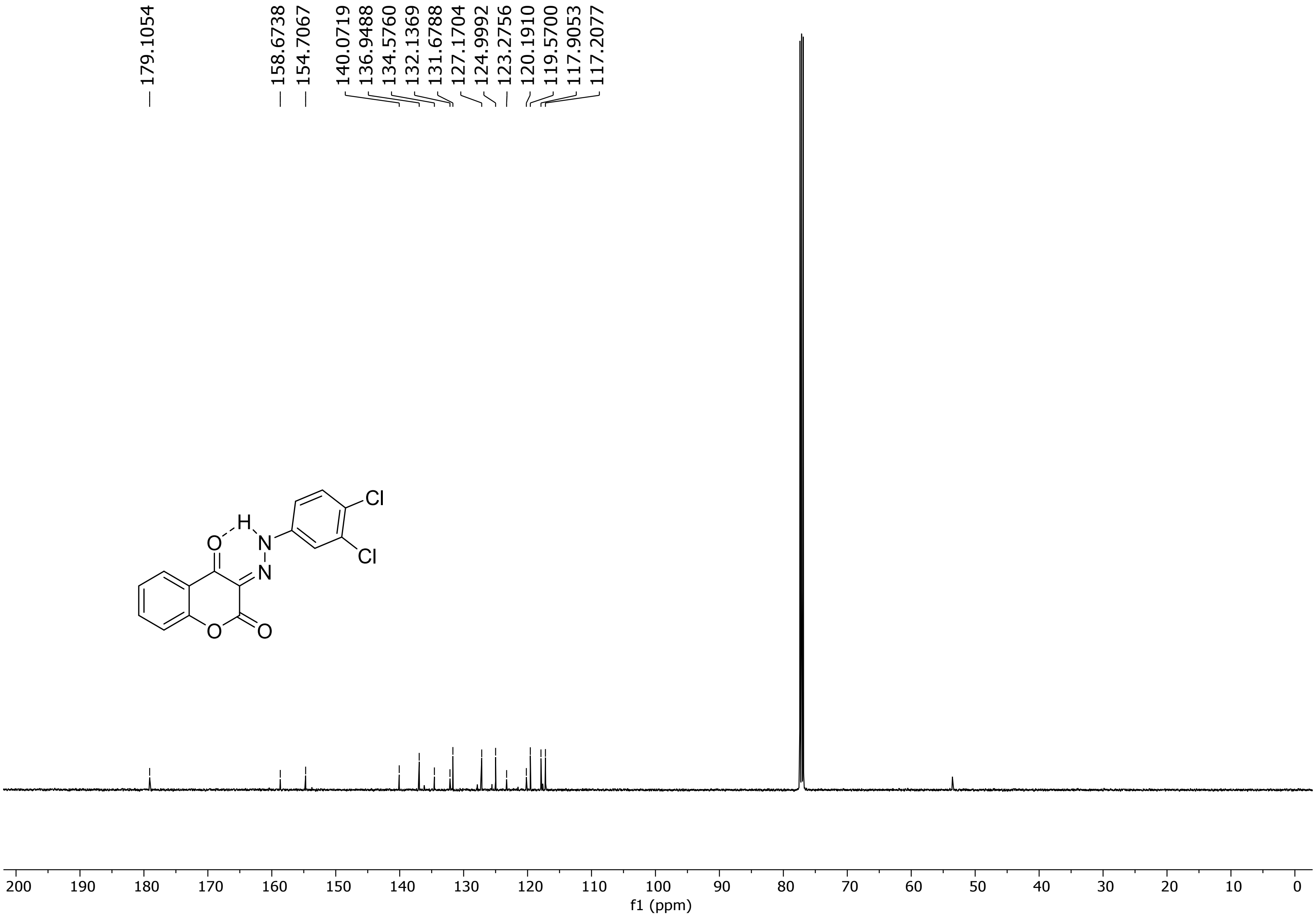

Figure S14. ^{13}C NMR spectrum of **3g** (125 MHz, $CDCl_3$)

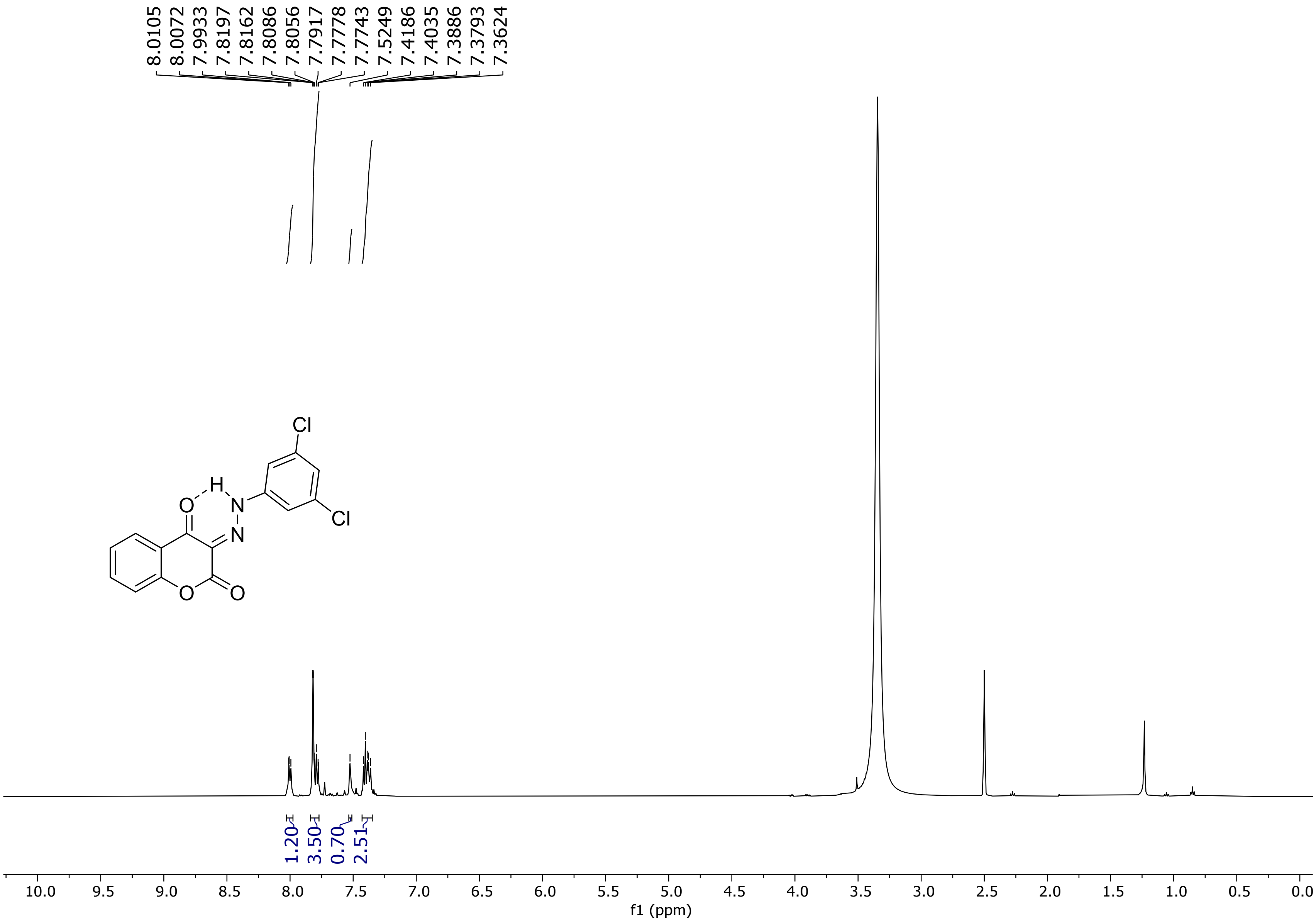

Figure S15. ^{1}H NMR spectrum of **3h** (500 MHz, DMSO-d6)

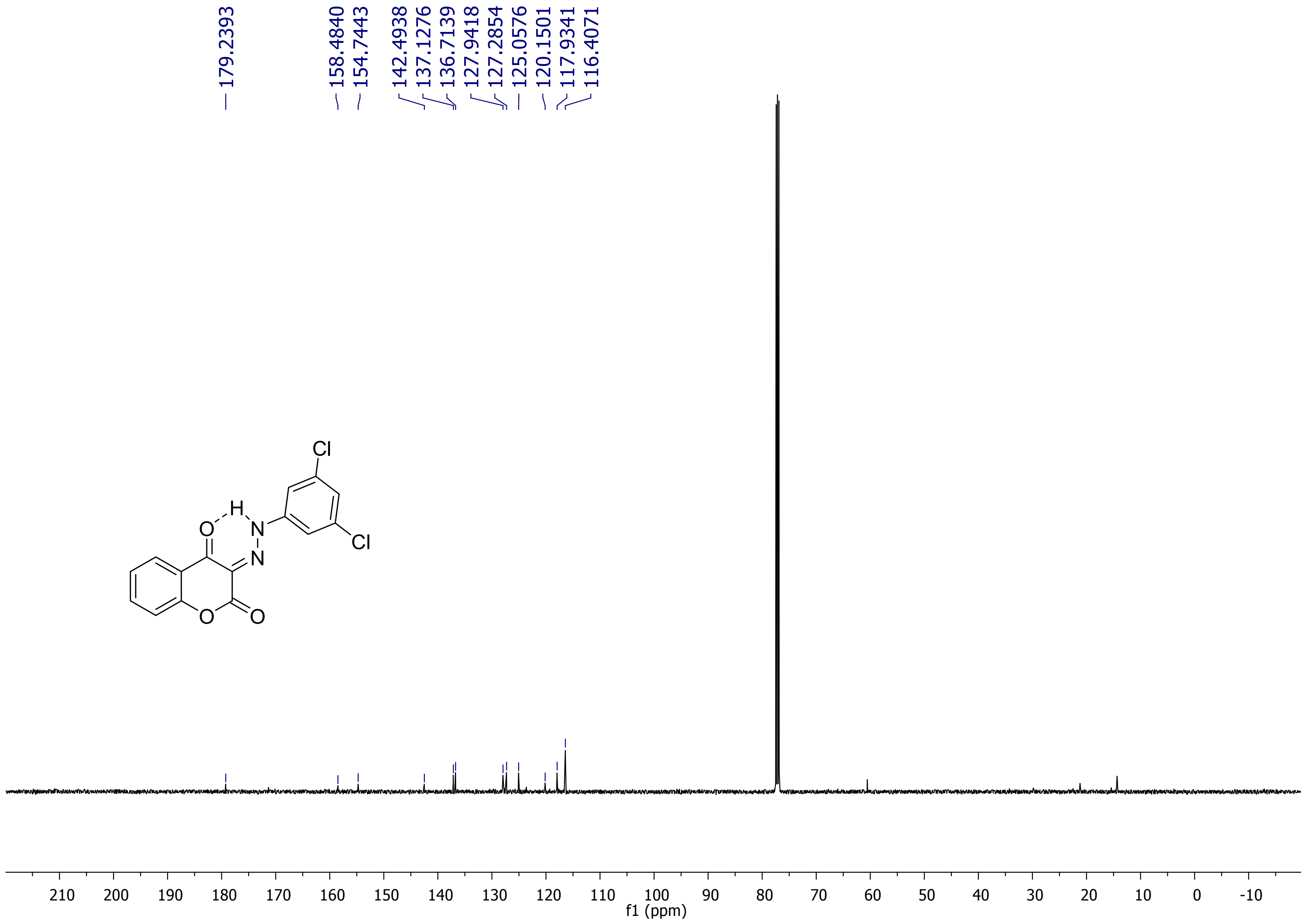

Figure S16. ^{13}C NMR spectrum of **3h** (125 MHz, $CDCl_3$)

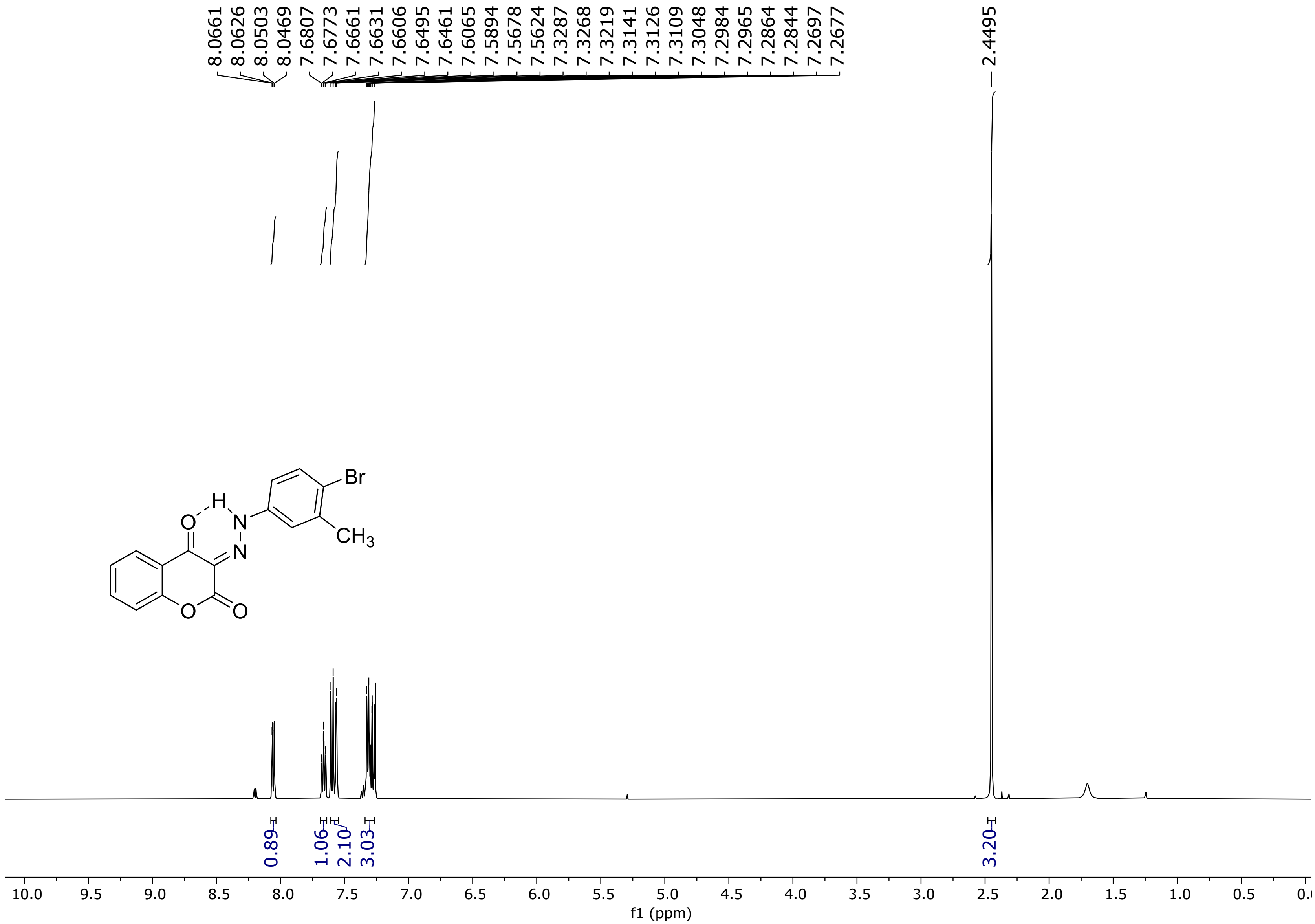

Figure S17. ^{1}H NMR spectrum of **3i** (500 MHz, $CDCl_3$)

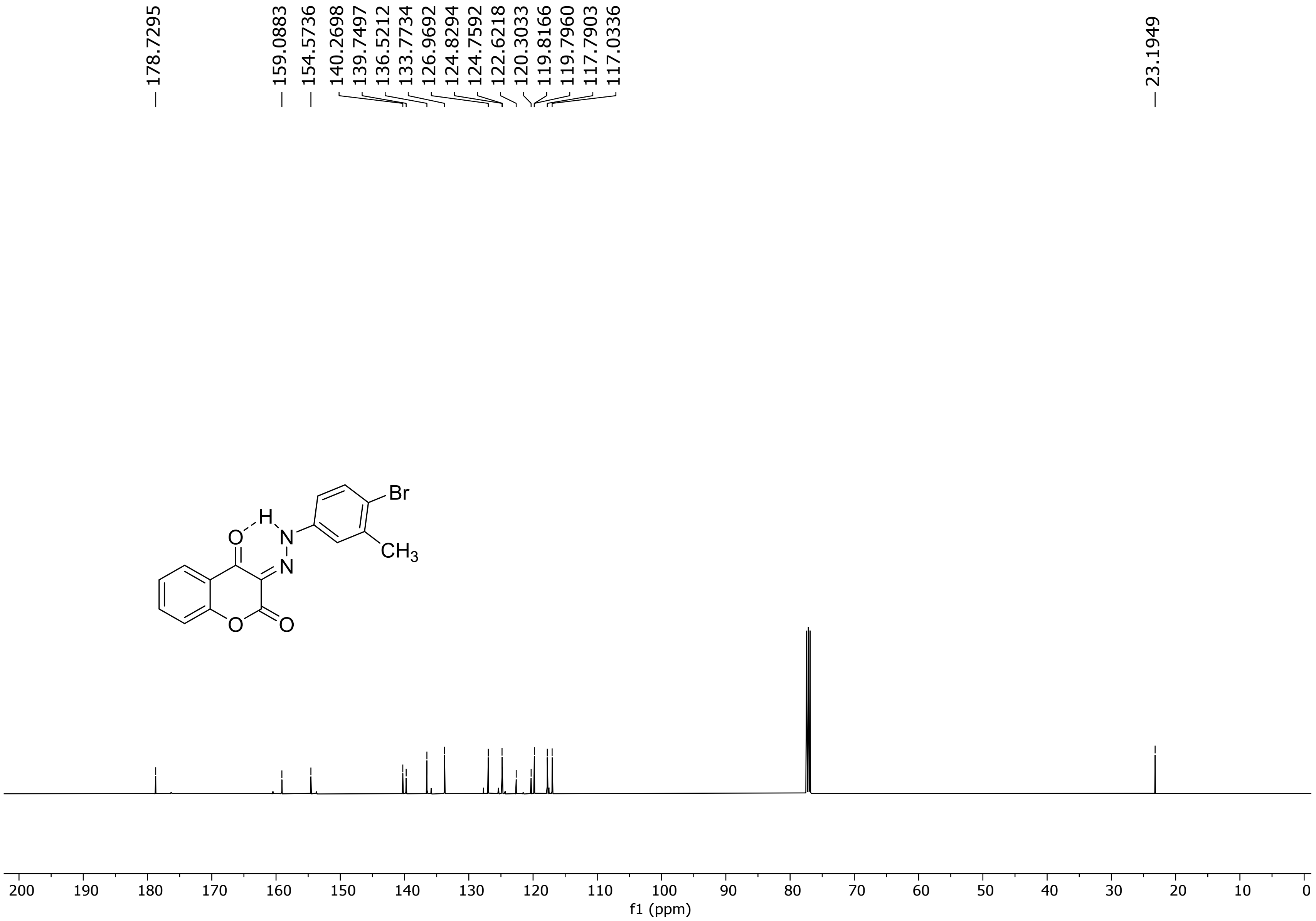

Figure S18. ^{13}C NMR spectrum of **3i** (125 MHz, $CDCl_3$)

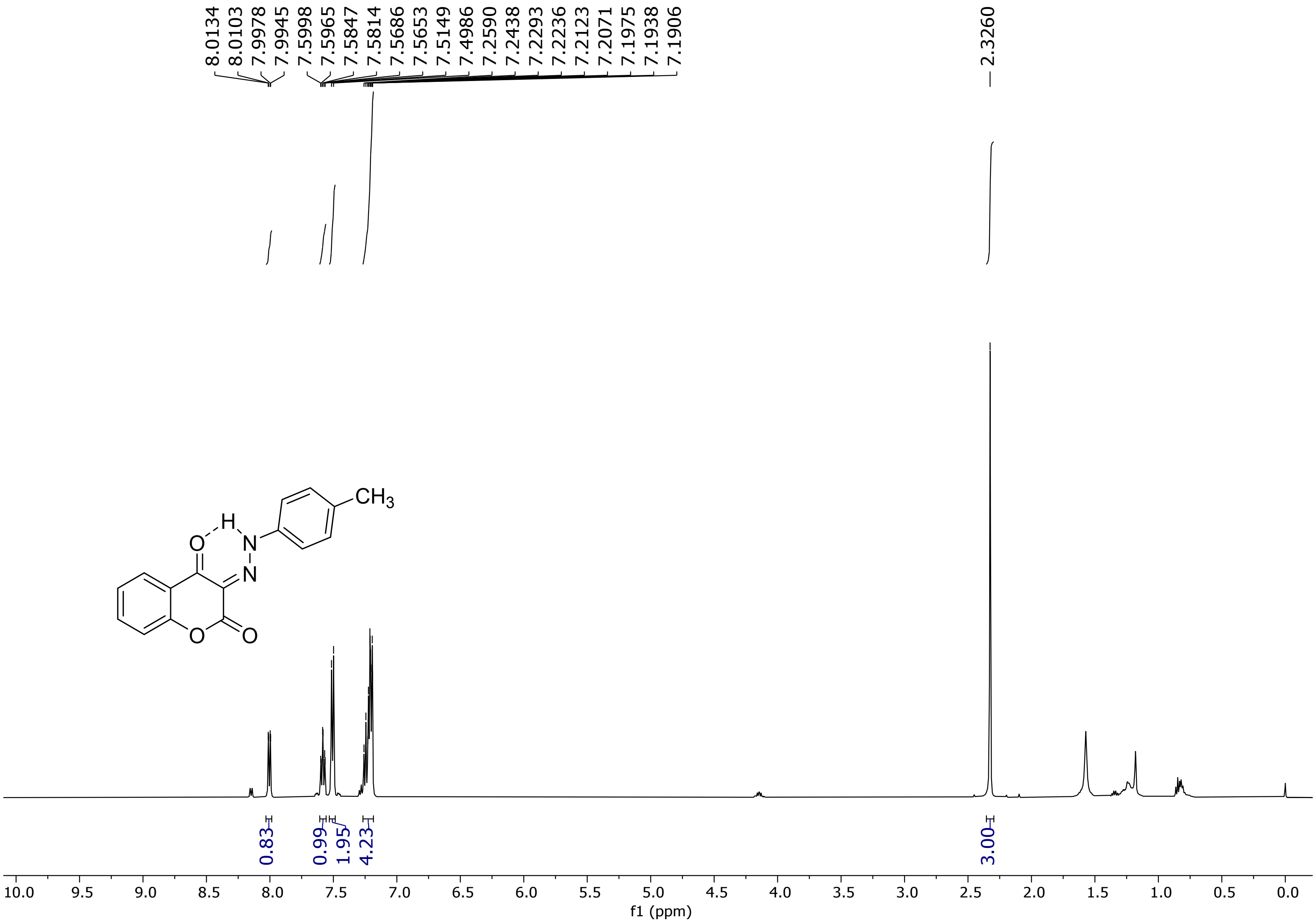

Figure S19. ^{1}H NMR spectrum of **3j** (500 MHz, $CDCl_3$)

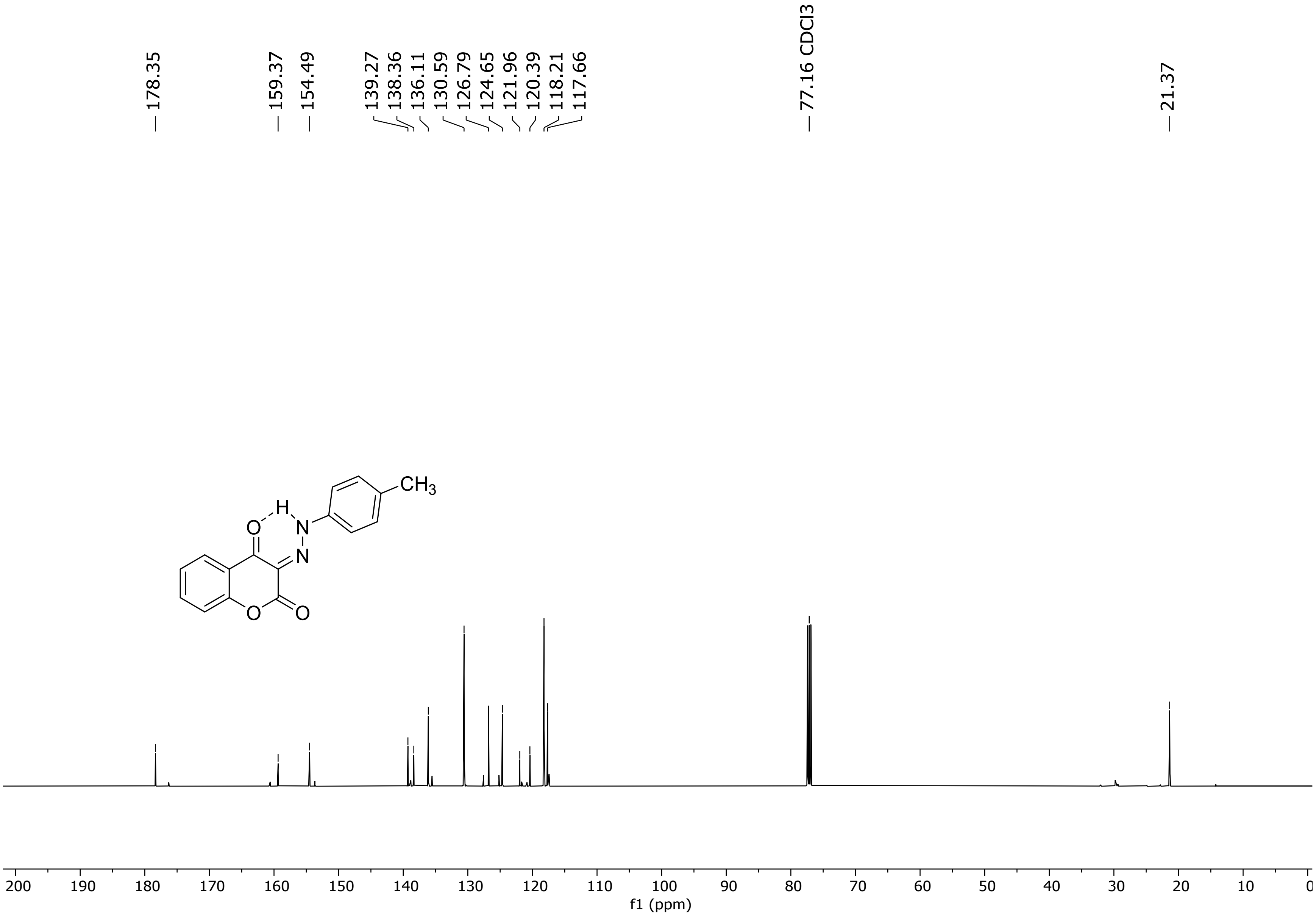

Figure S20. ${}^{13}C$ NMR spectrum of **3j** (125 MHz, $CDCl_3$)

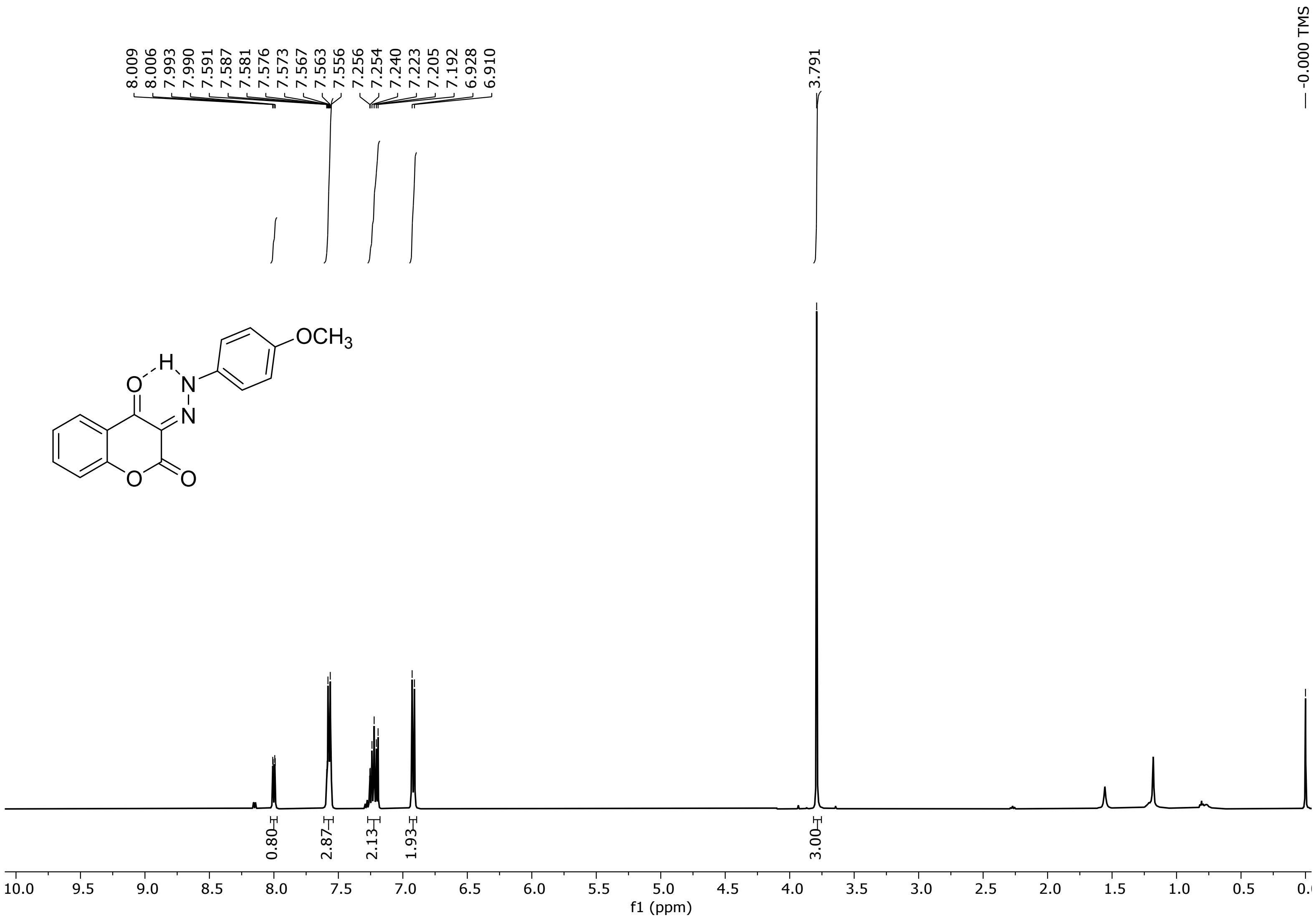

Figure S21. ^{1}H NMR spectrum of **3k** (500 MHz, $CDCl_3$)

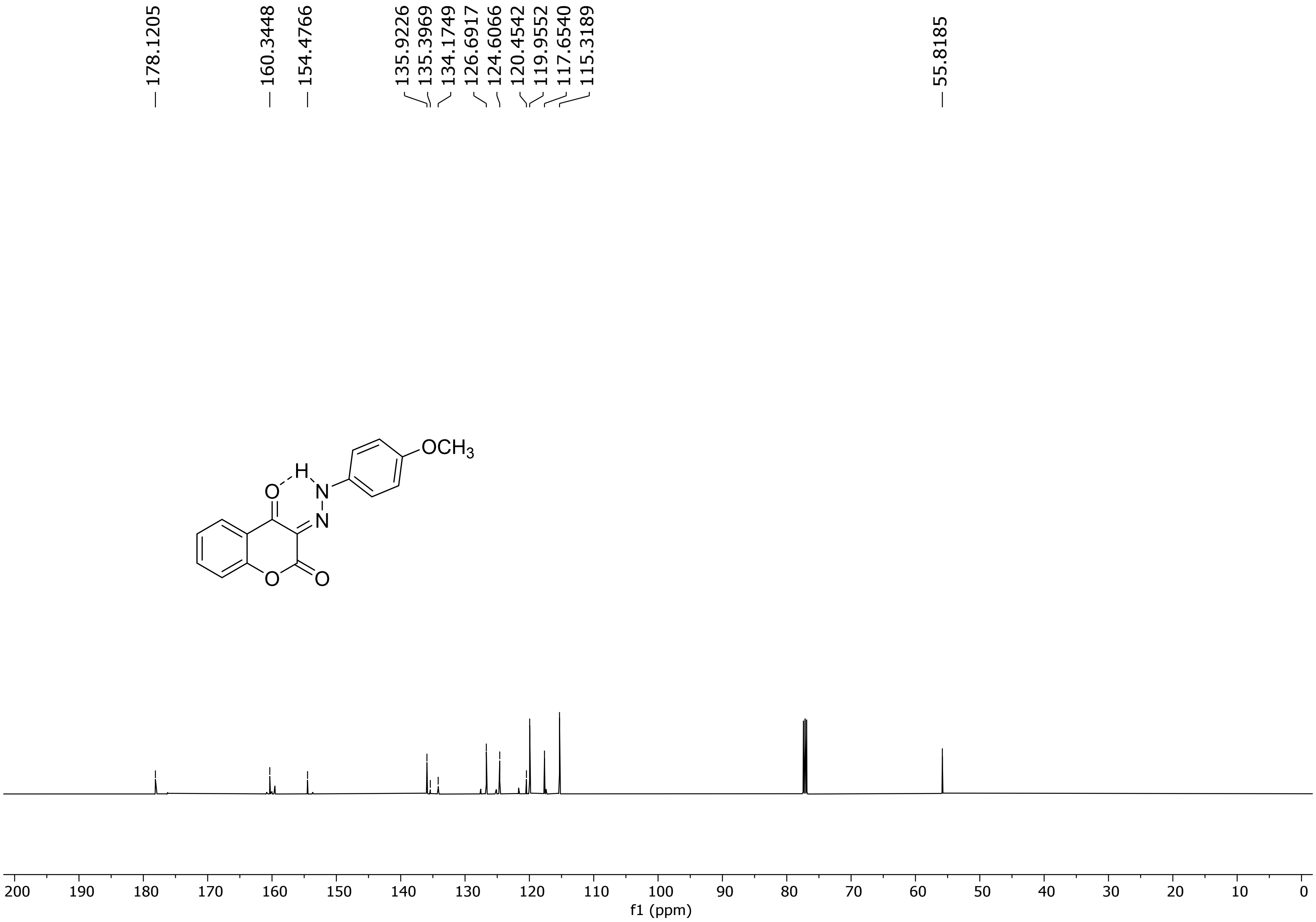

Figure S22. ^{13}C NMR spectrum of **3k** (125 MHz, $CDCl_3$)

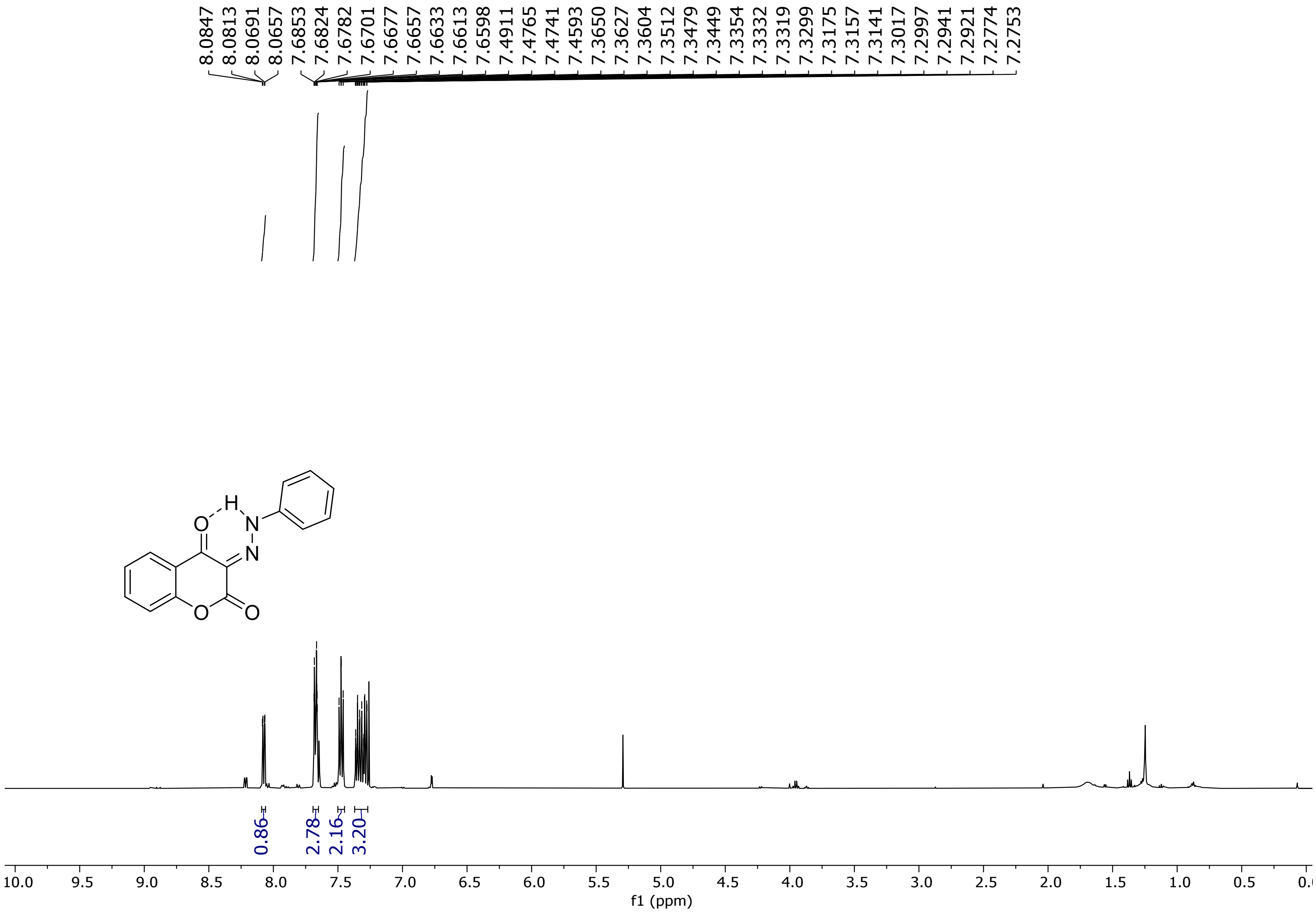

Figure S23. ^{1}H NMR spectrum of **3l** (500 MHz, $CDCl_3$)

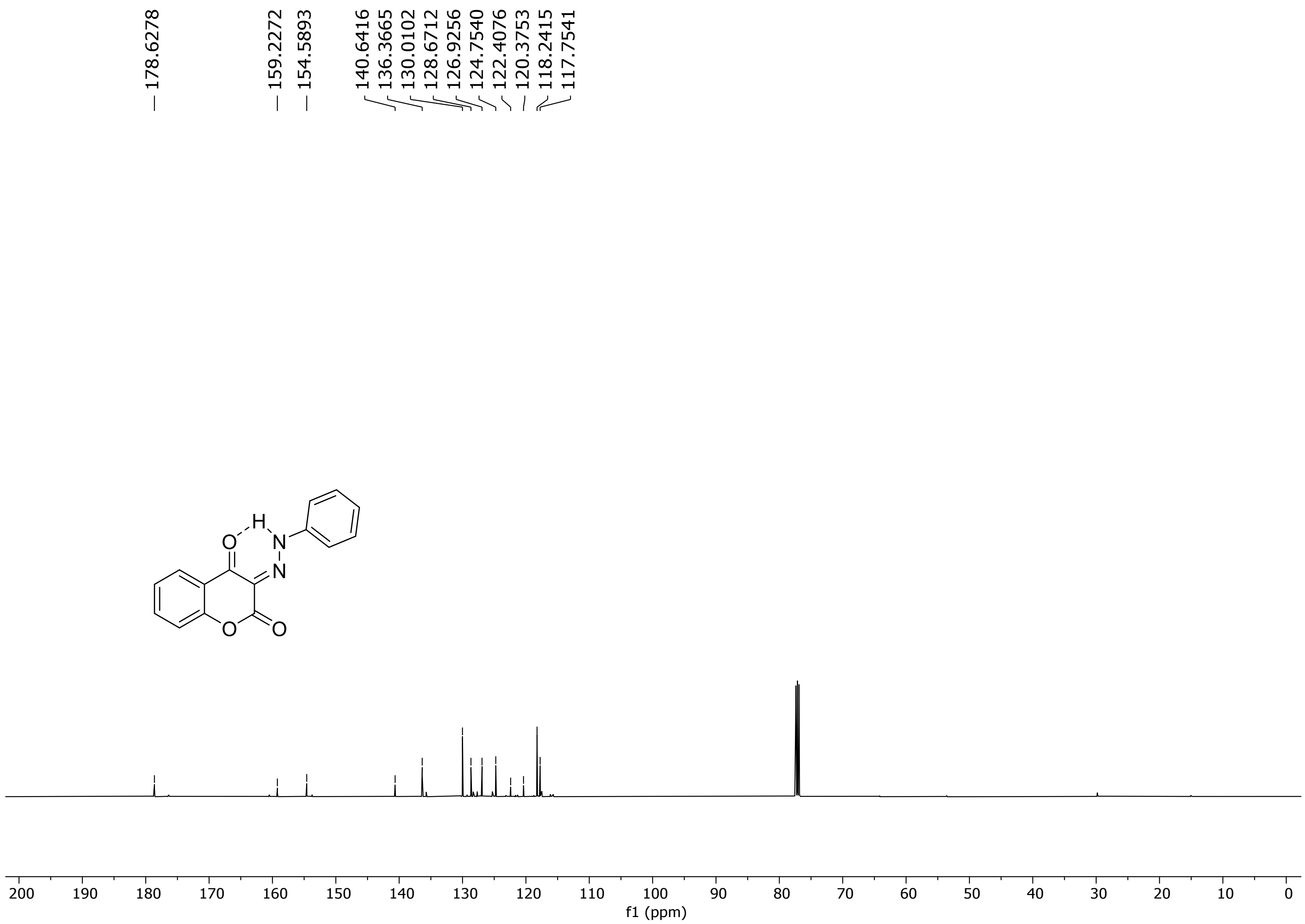

Figure S24. ^{13}C NMR spectrum of **3l** (125 MHz, $CDCl_3$)

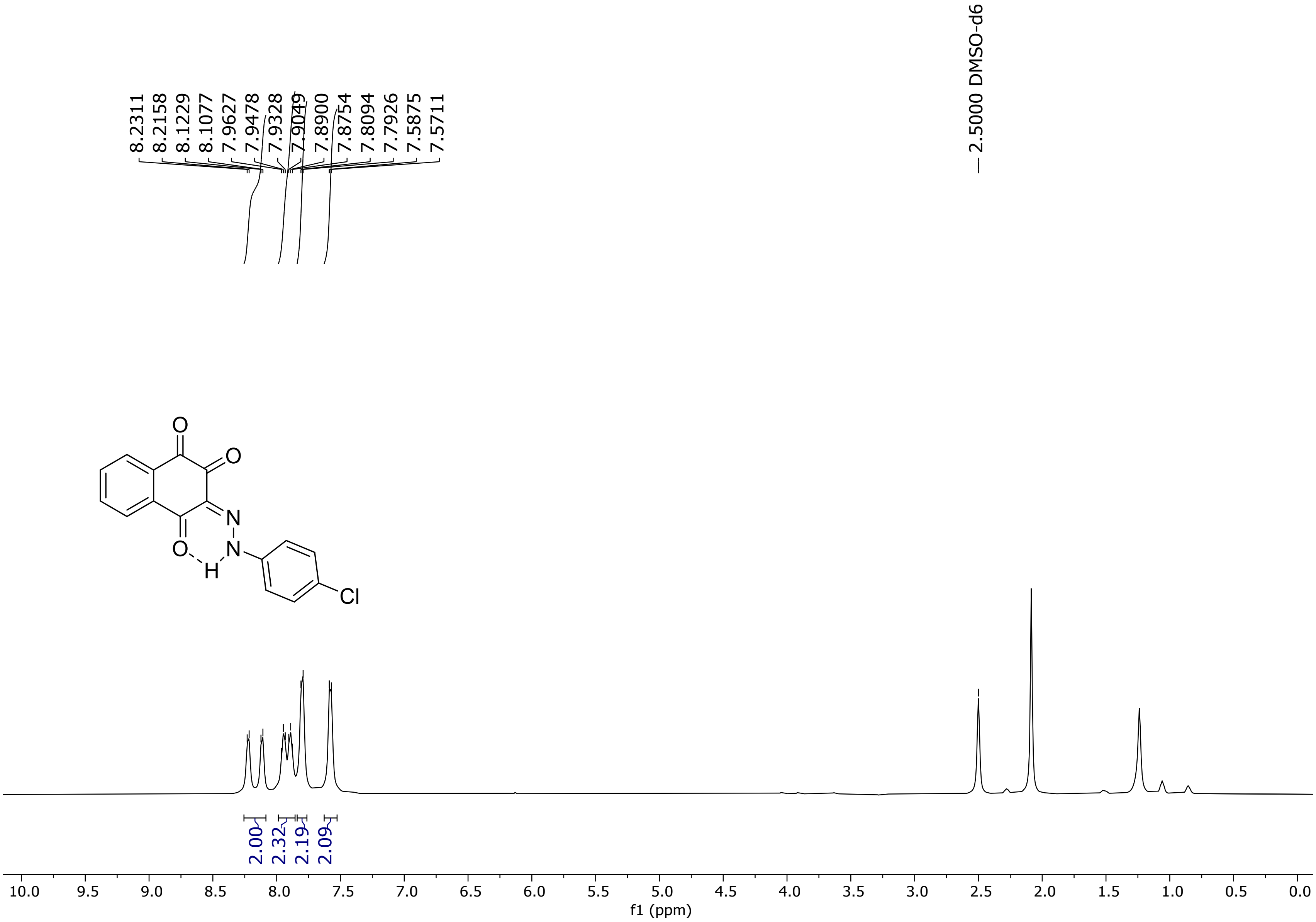

Figure S25. ^{1}H NMR spectrum of **3m** (500 MHz, DMSO-d6)

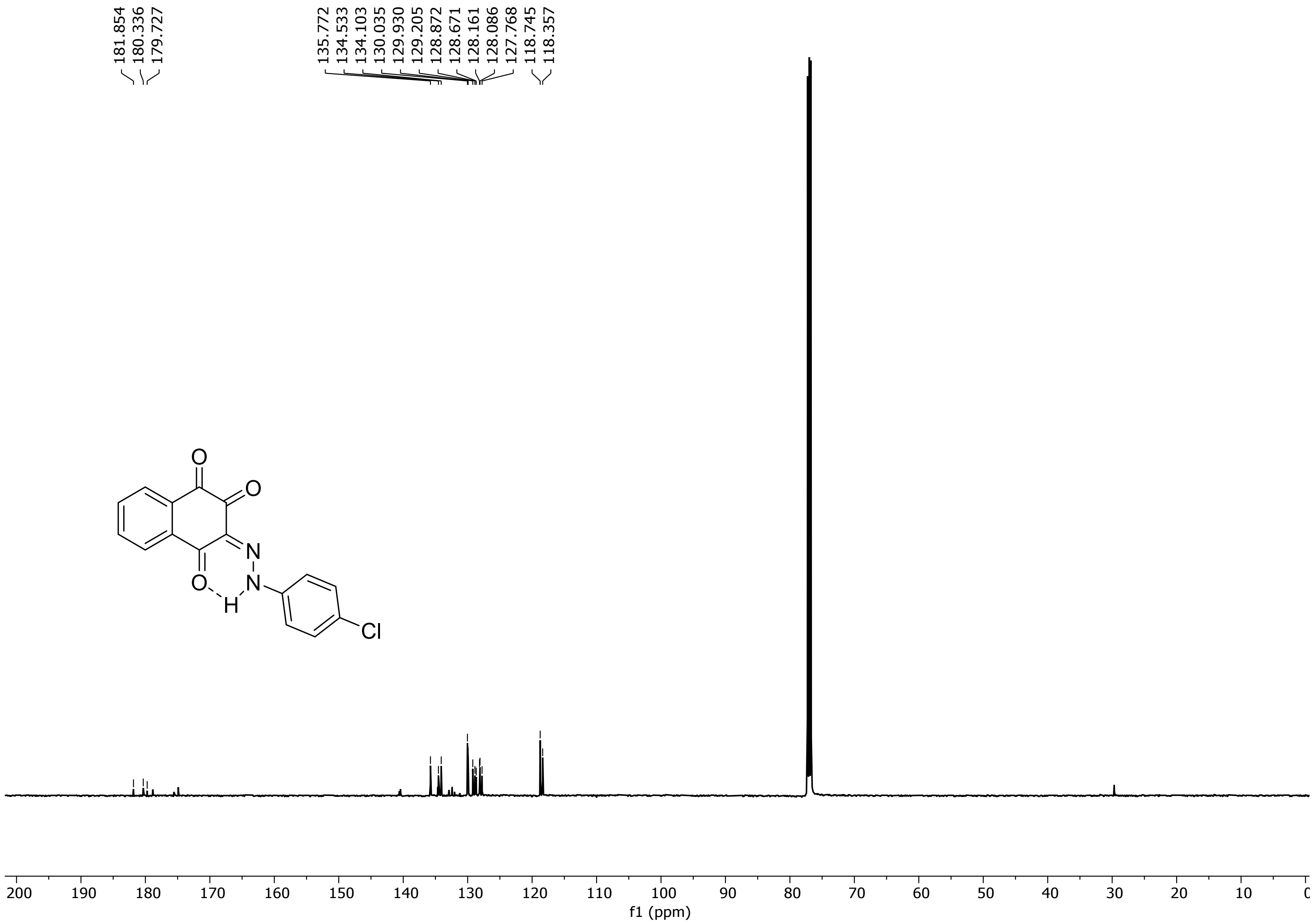

Figure S26. ^{13}C NMR spectrum of **3m** (125 MHz, $CDCl_3$)

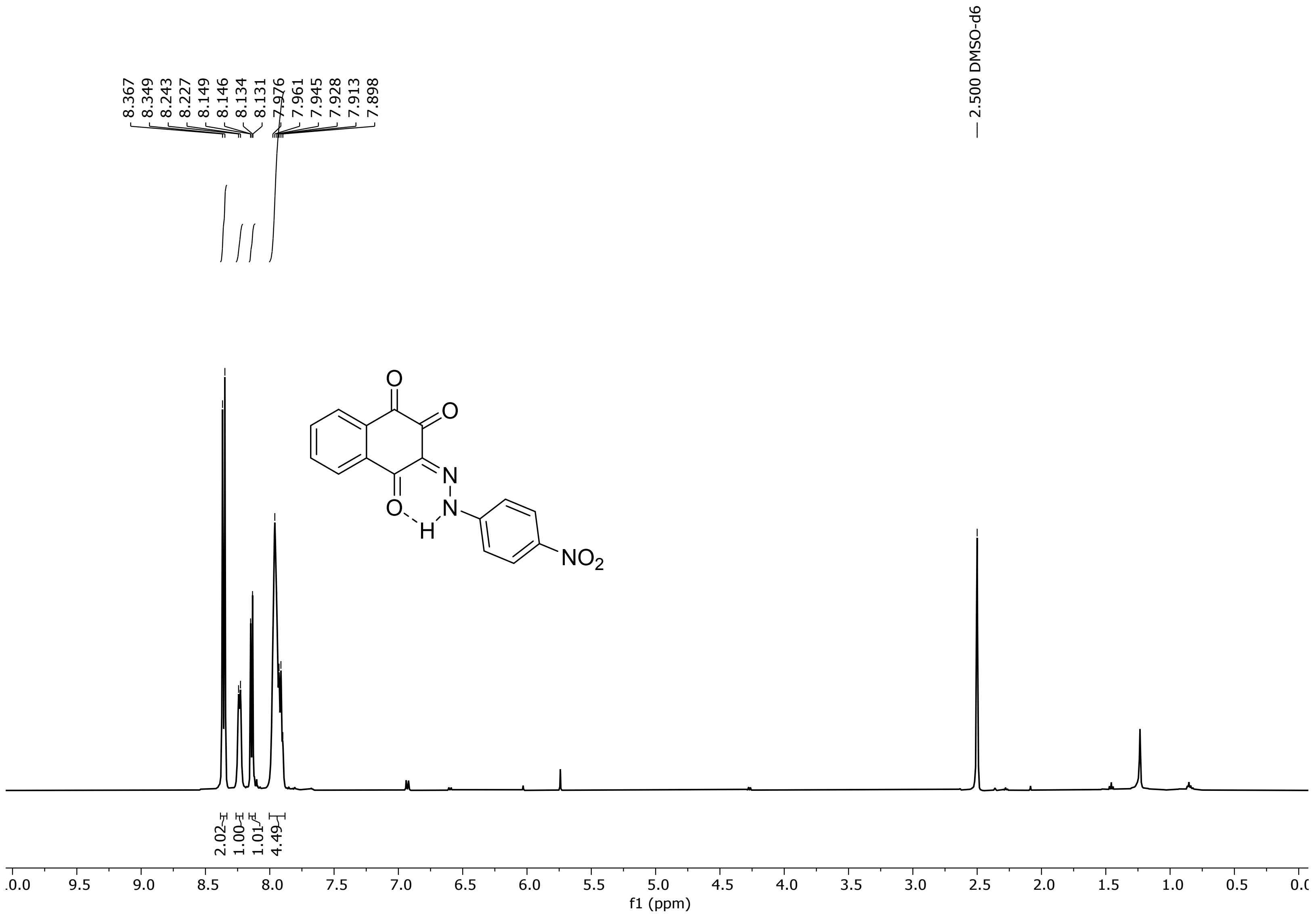

Figure S27. ^{1}H NMR spectrum of **3n** (500 MHz, DMSO-d6)

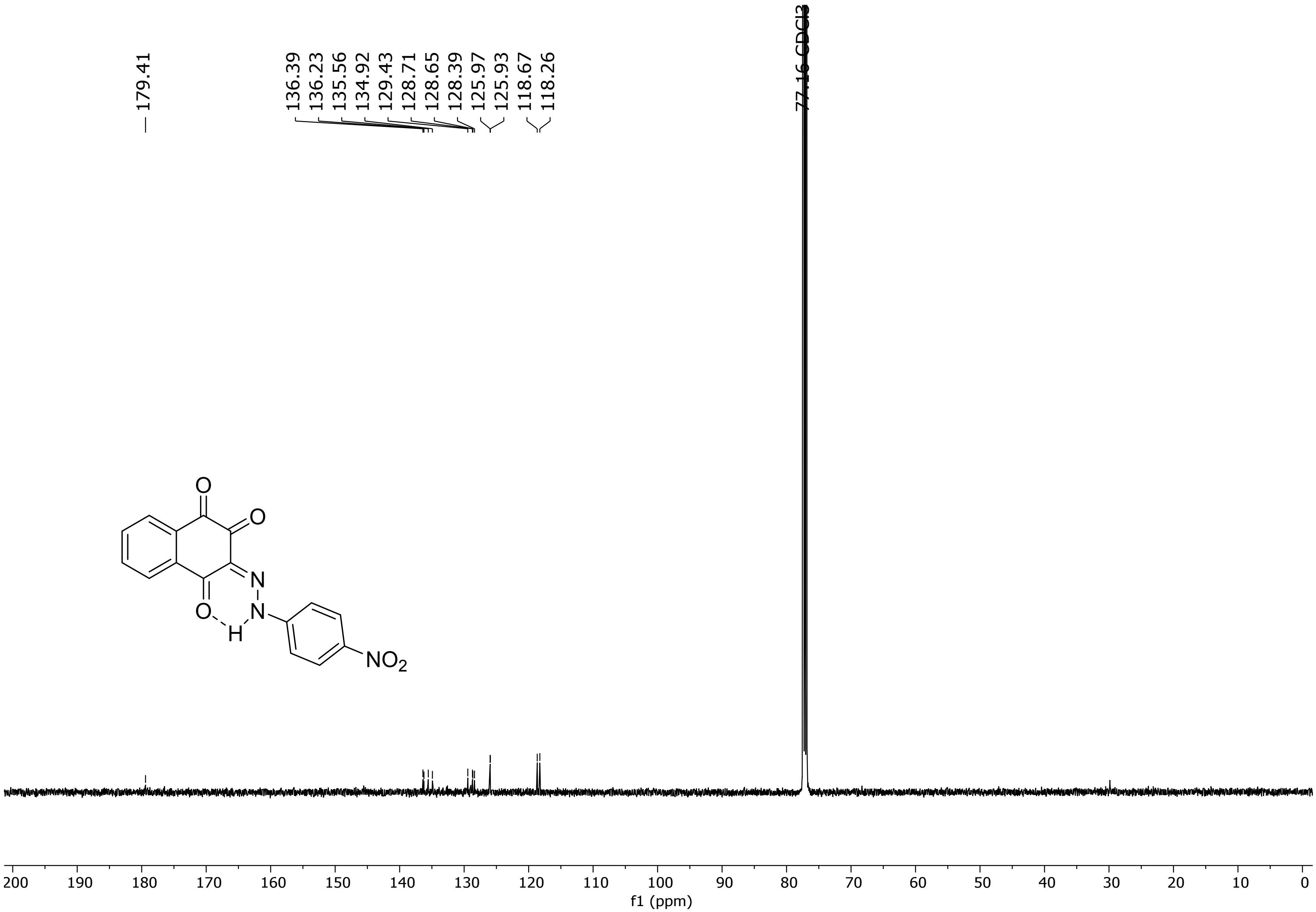

Figure S28. ^{13}C NMR spectrum of **3n** (125 MHz, $CDCl_3$)

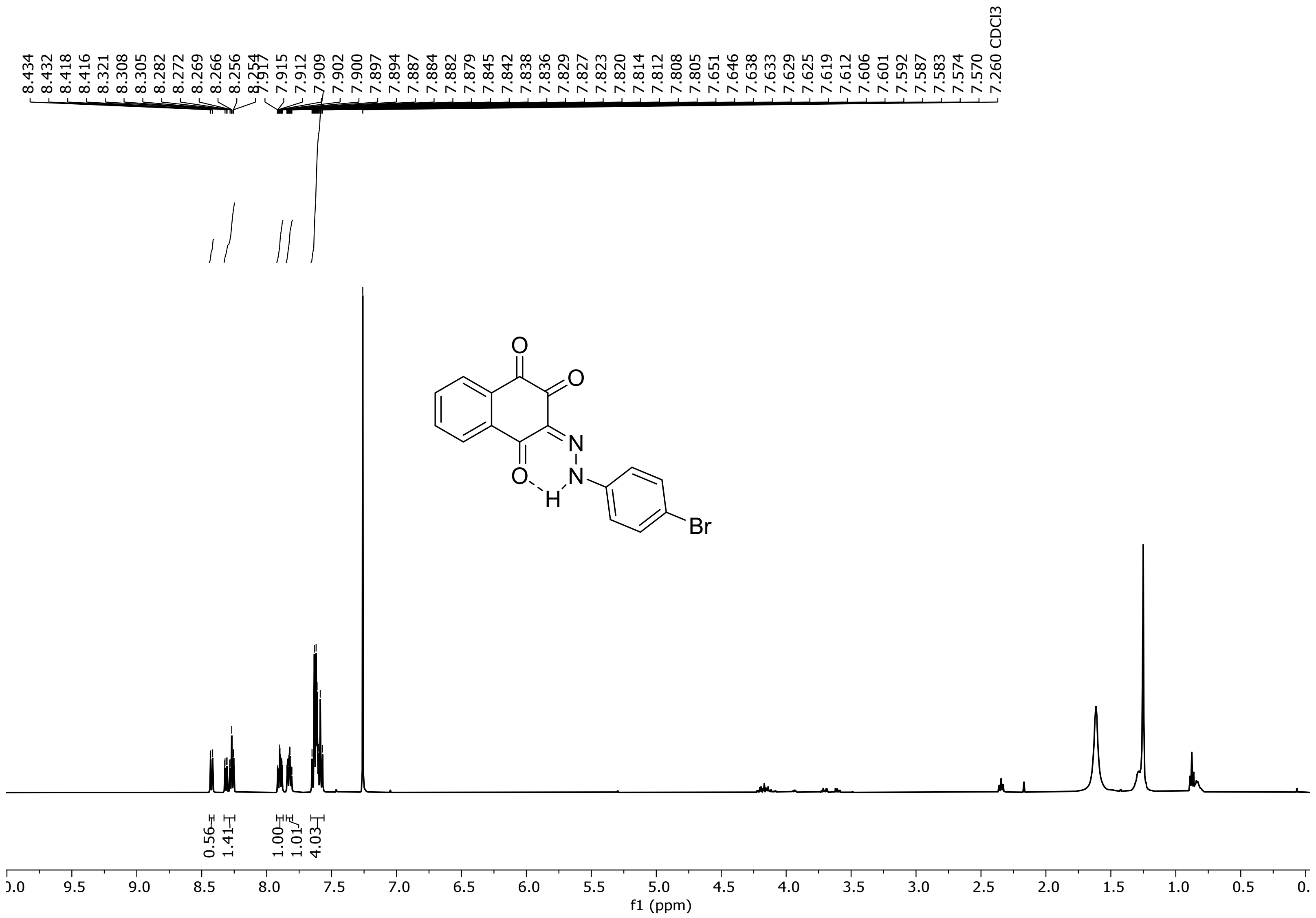

Figure S29. ^{1}H NMR spectrum of **3o** (500 MHz, $CDCl_3$)

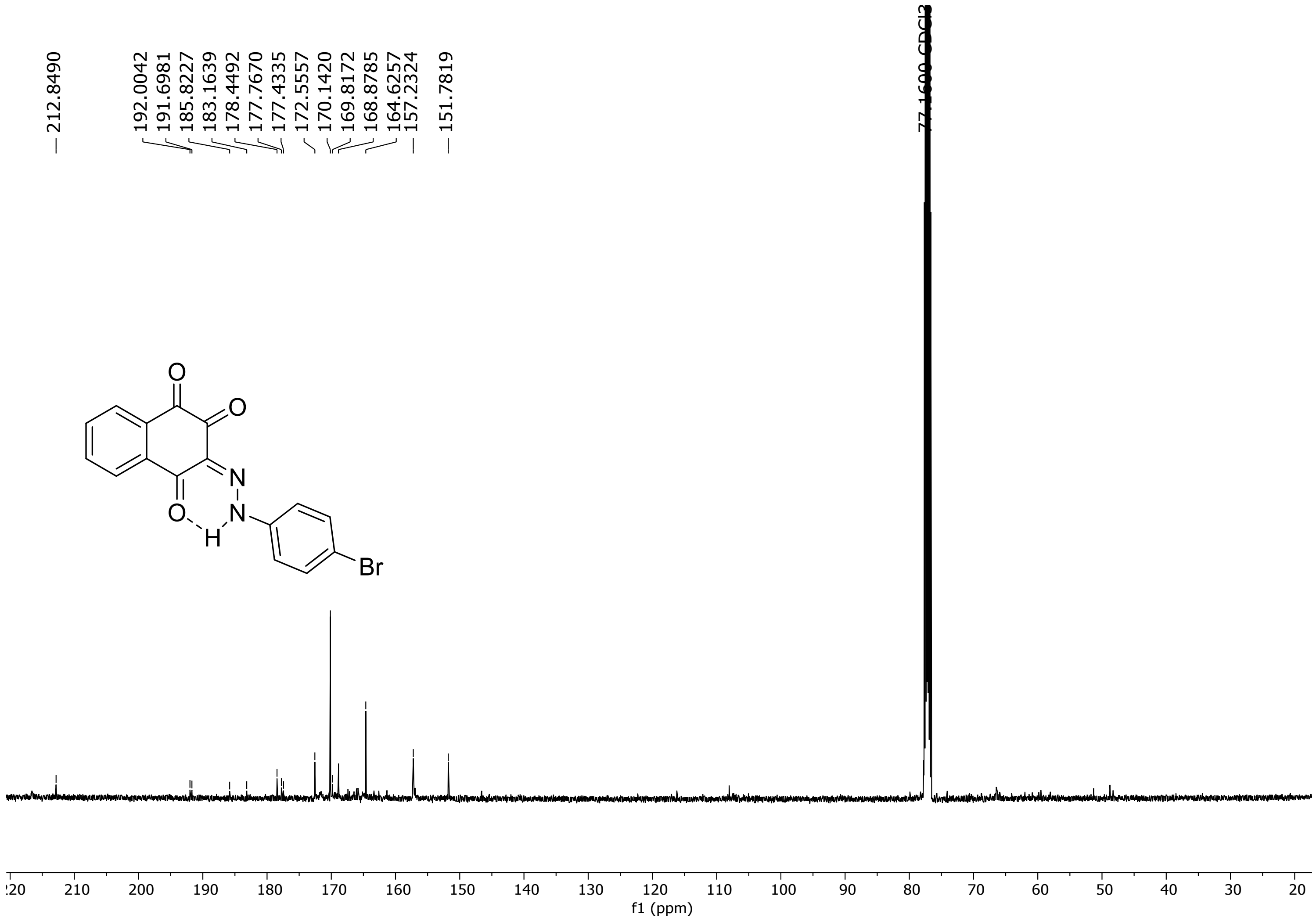

Figure S30. ^{13}C NMR spectrum of **3o** (126 MHz, $CDCl_3$)

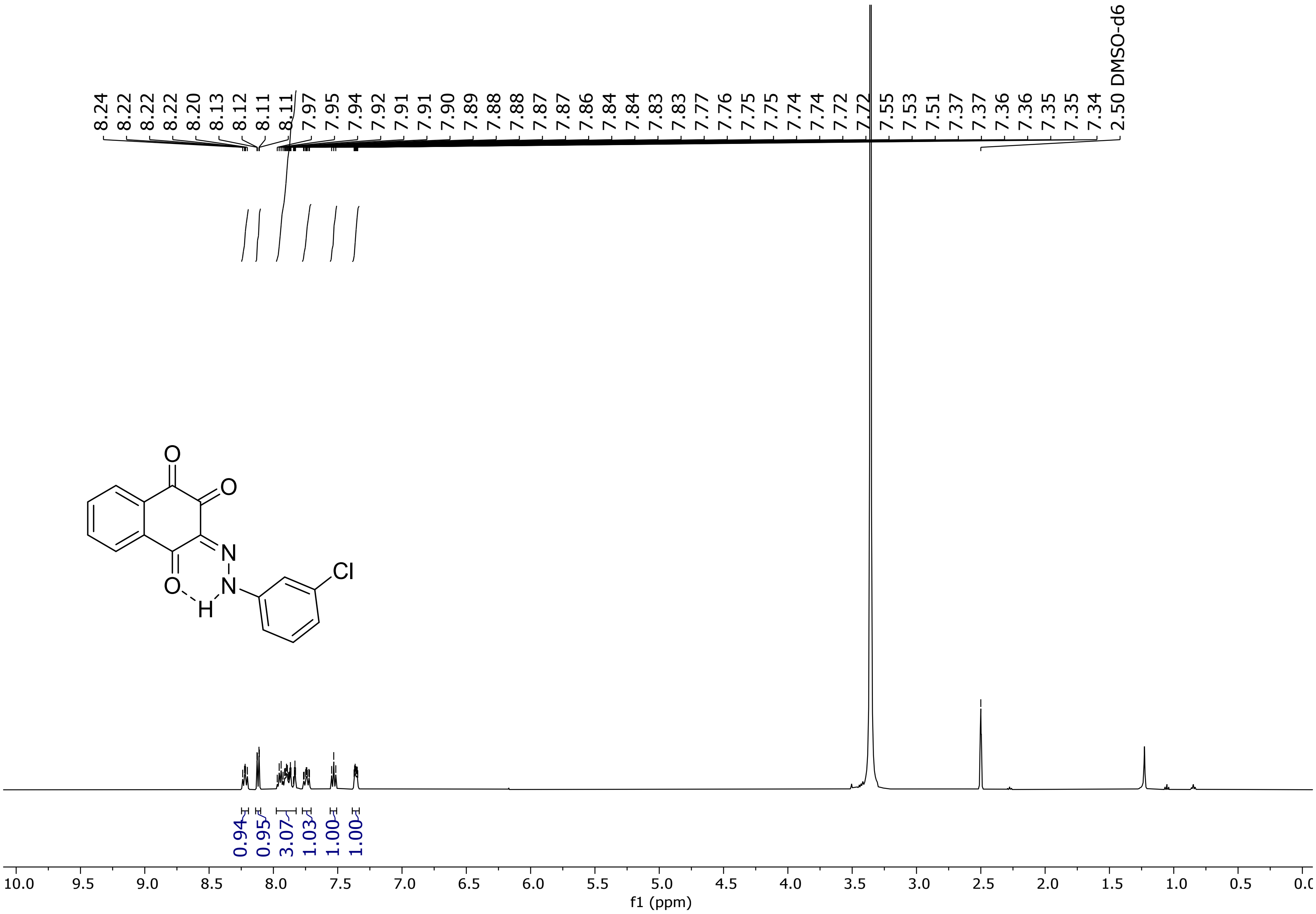

Figure S31. ^{1}H NMR spectrum of **3p** (500 MHz, DMSO-d6)

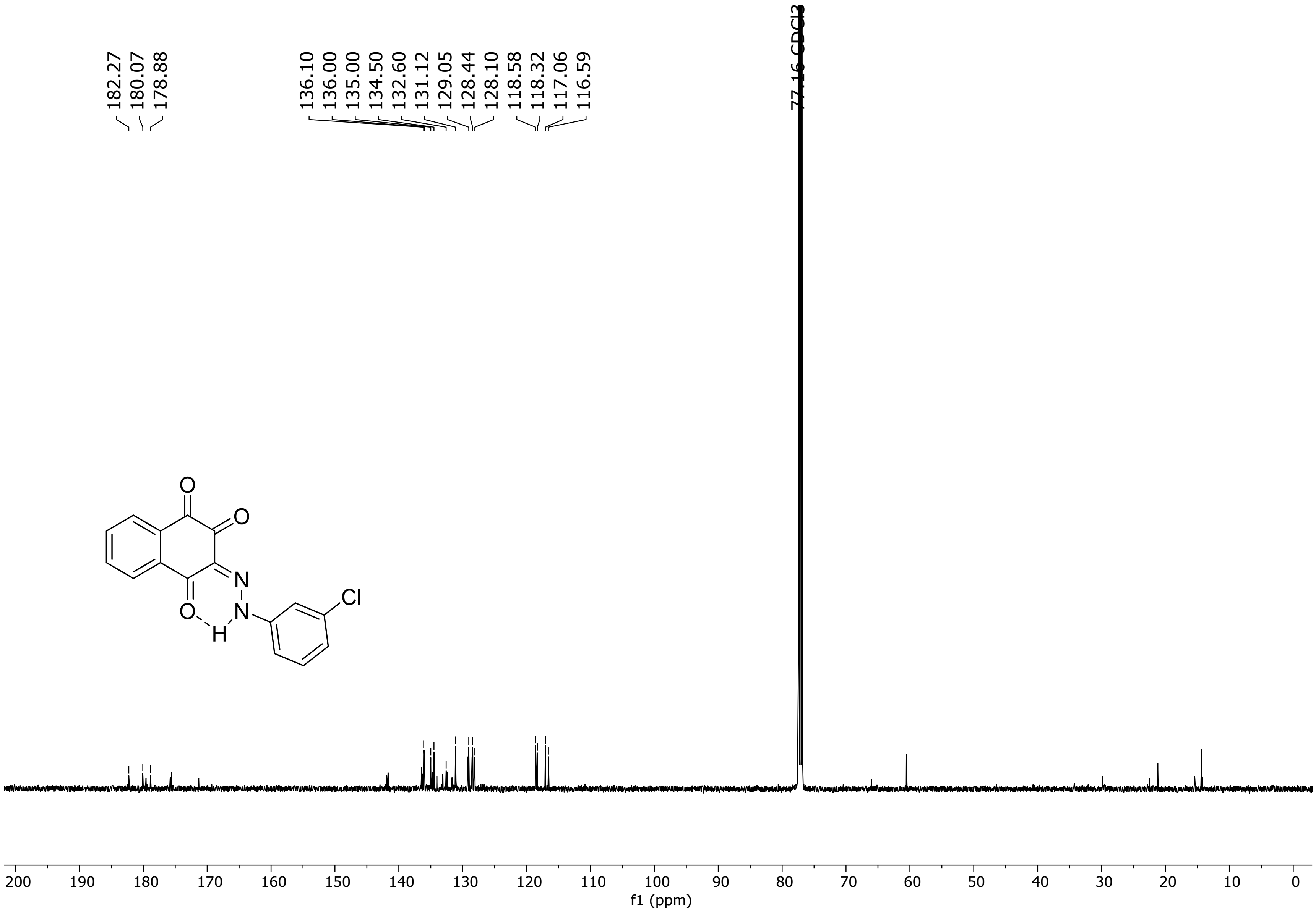

Figure S32. ^{13}C NMR spectrum of **3p** (125 MHz, $CDCl_3$)

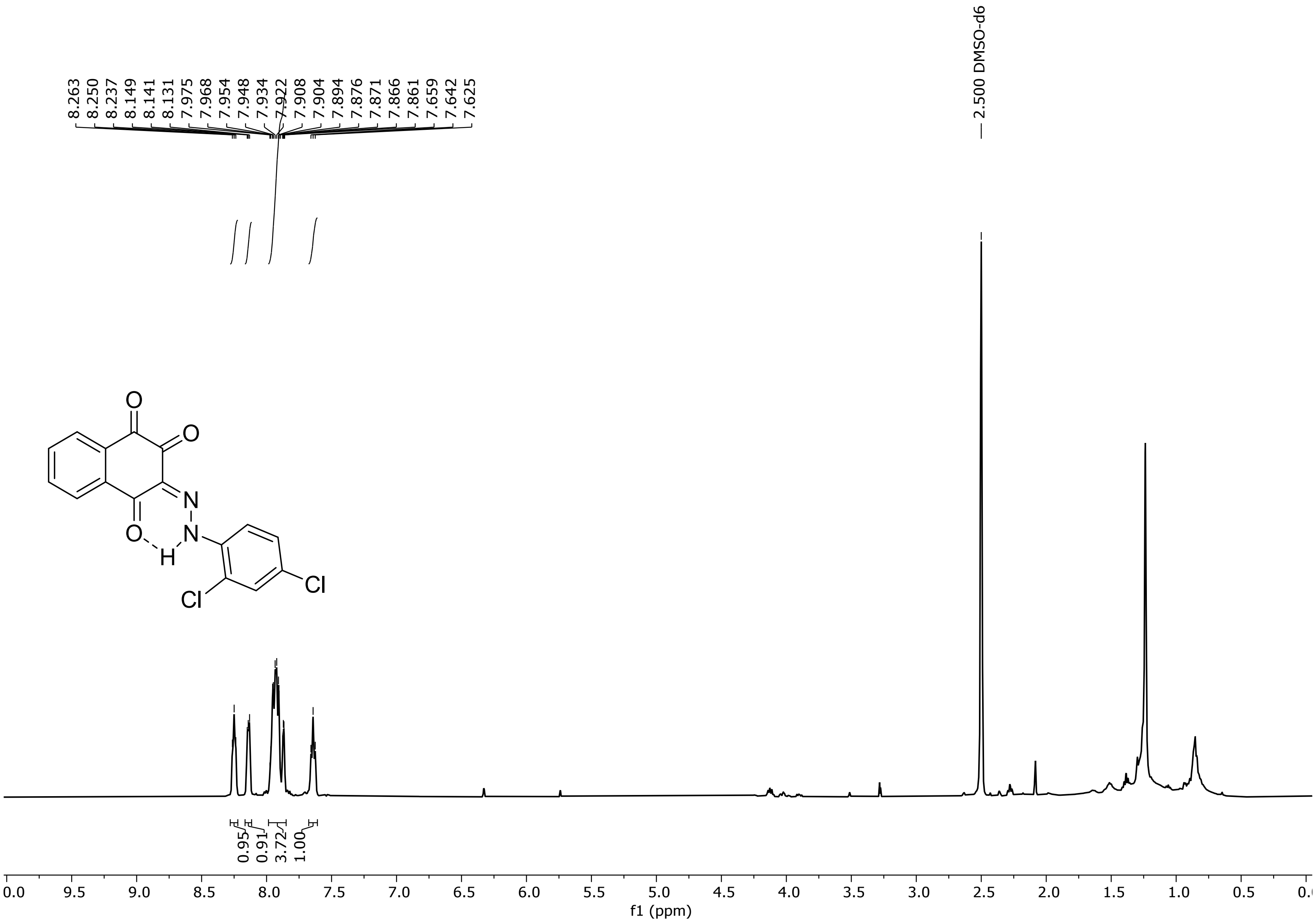

Figure S33. ^{1}H NMR spectrum of **3q** (500 MHz, DMSOd6)

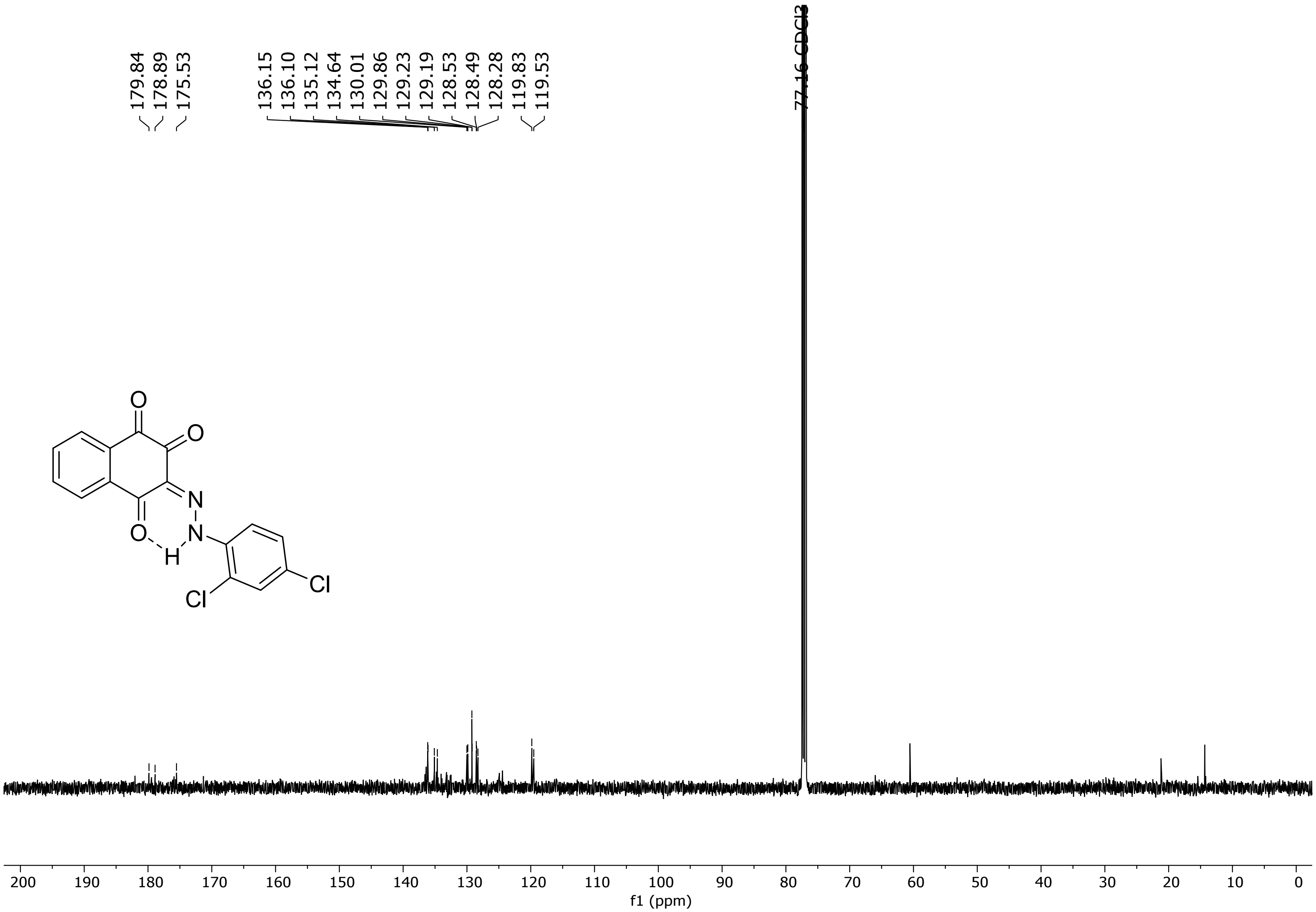

Figure S34. ^{13}C NMR spectrum of **3q** (125 MHz, $CDCl_3$)

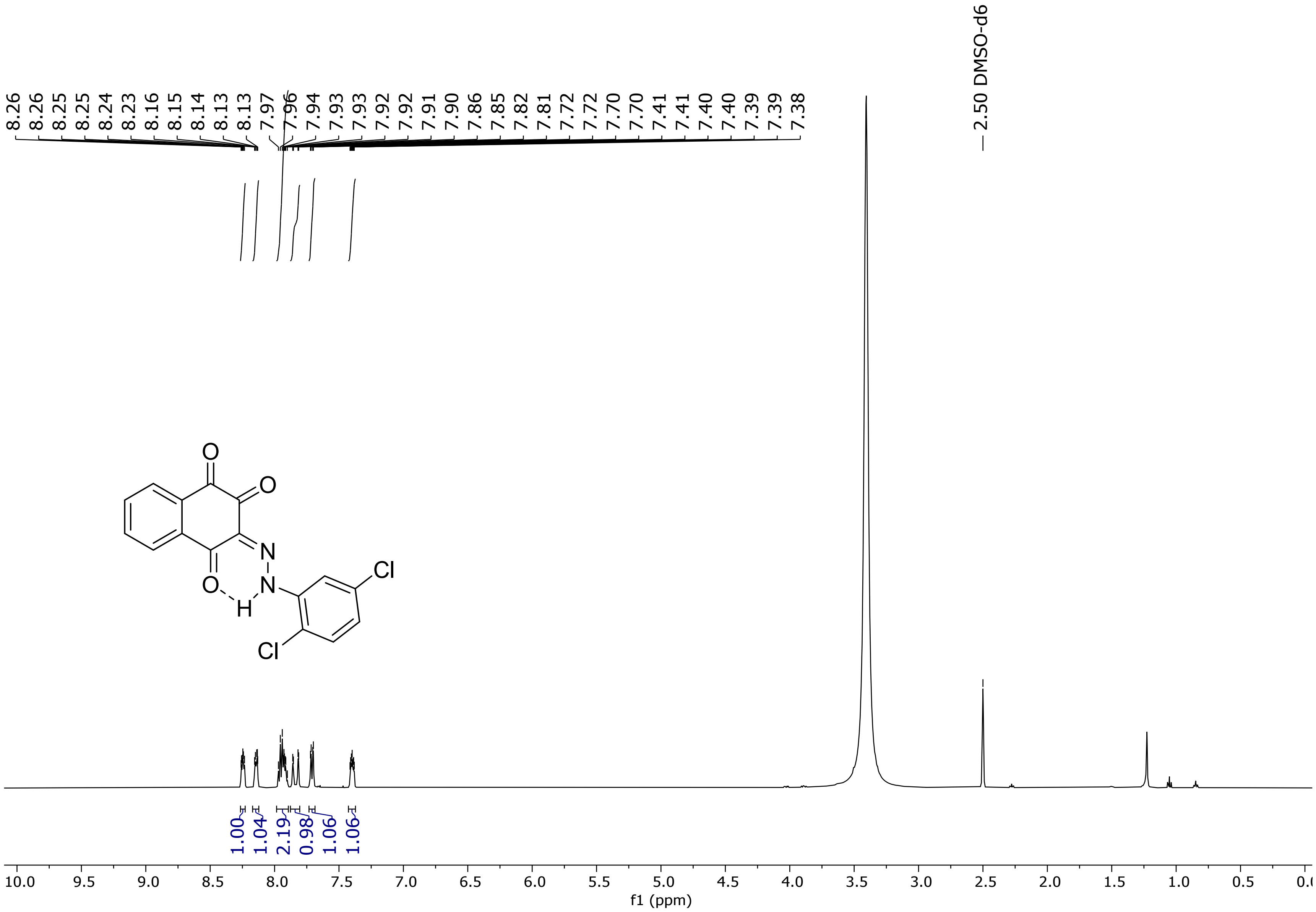

Figure S35. ^{1}H NMR spectrum of **3r** (500 MHz, $CDCl_3$)

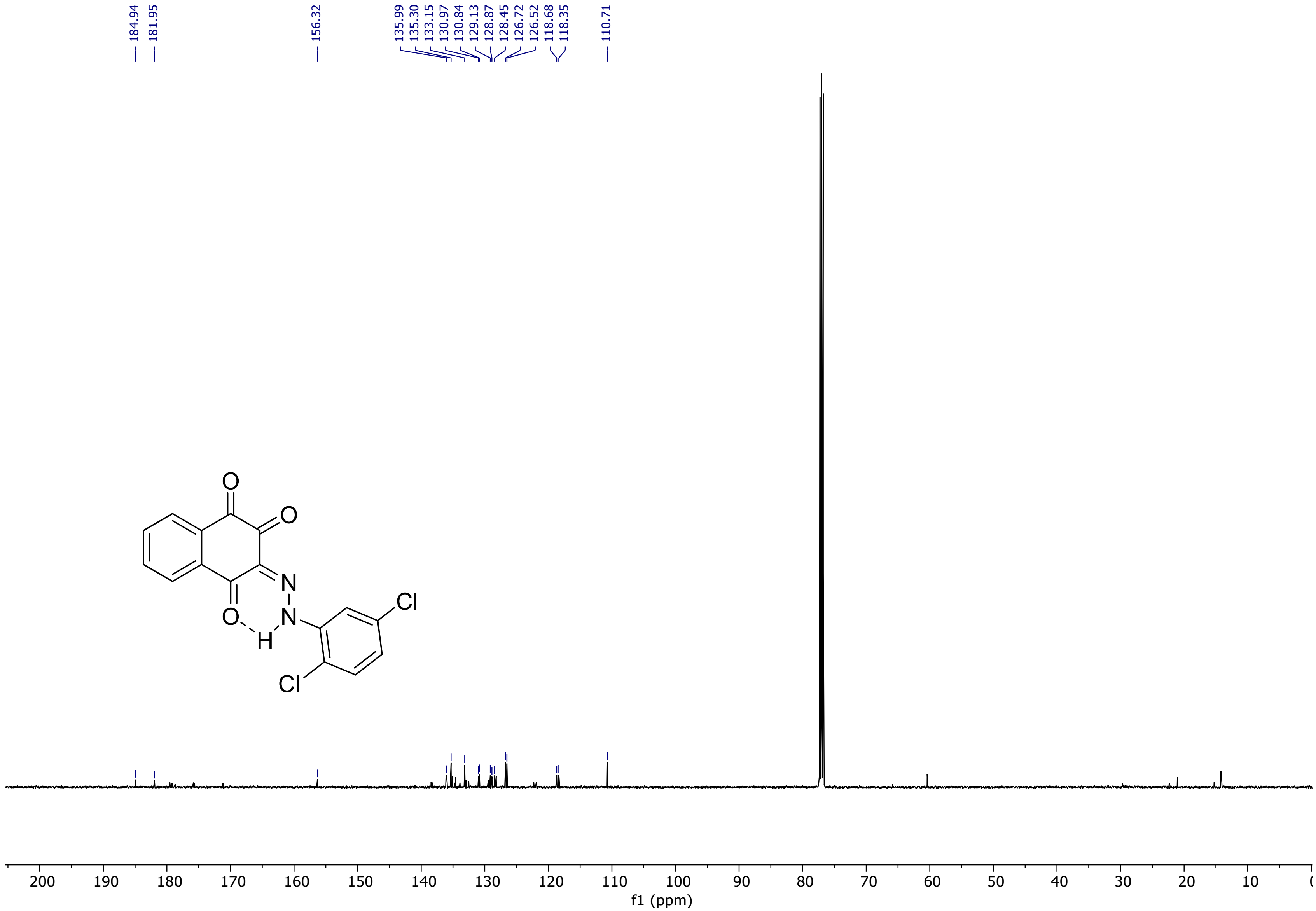

Figure S36. ^{13}C NMR spectrum of **3r** (125 MHz, $CDCl_3$)

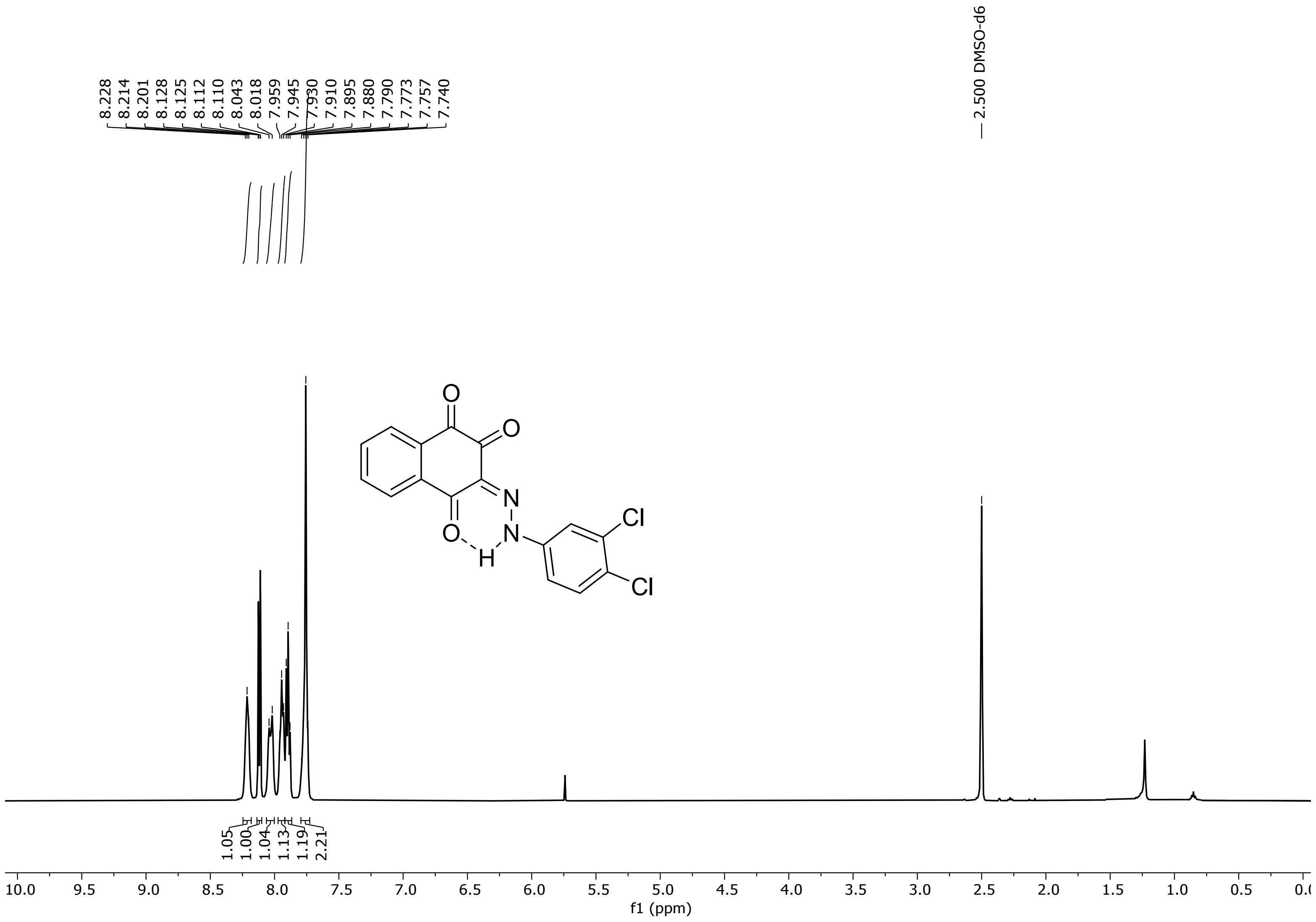

Figure S37. ^{1}H NMR spectrum of **3s** (500 MHz, DMSOd6)

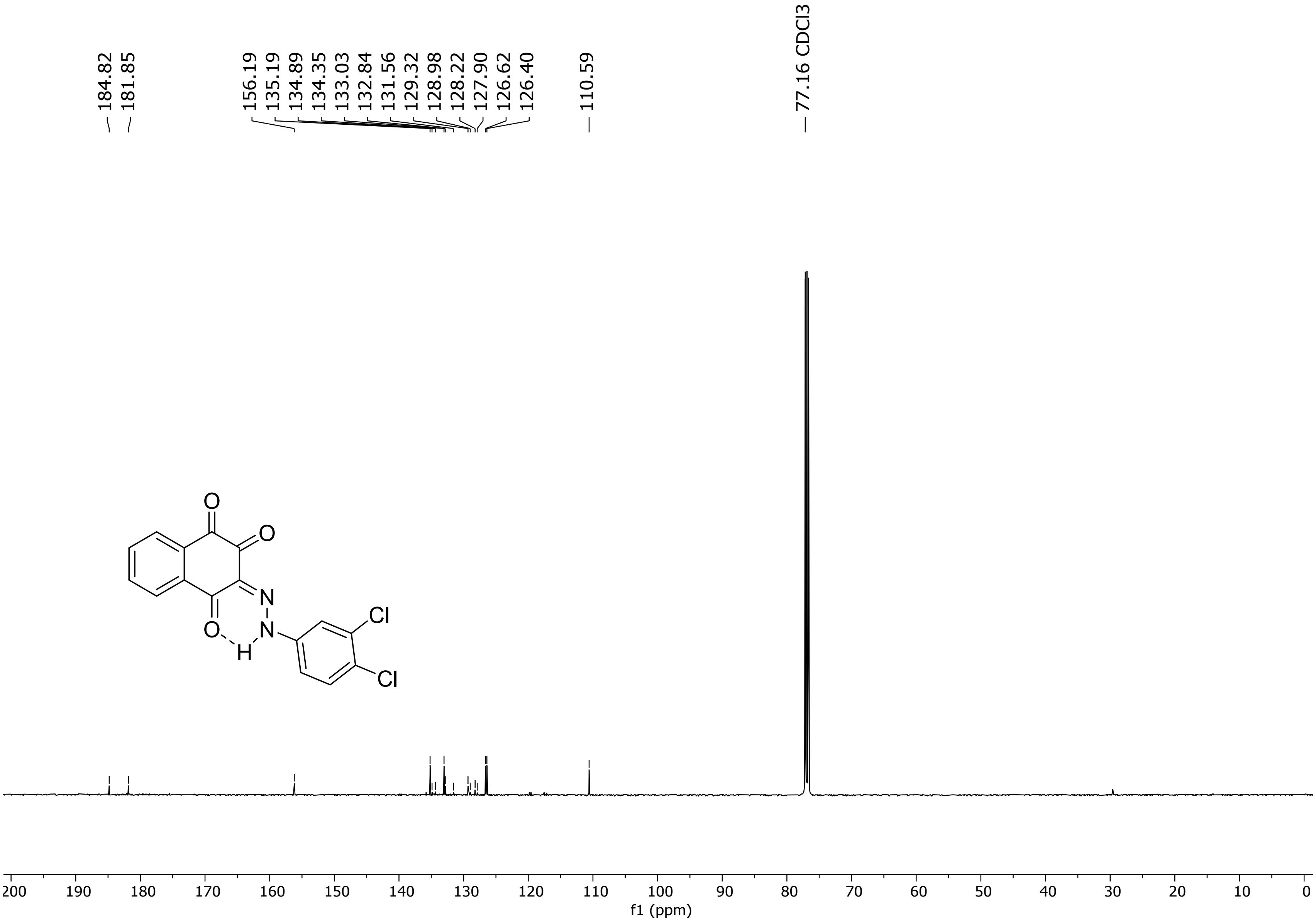

Figure S38. ^{13}C NMR spectrum of **3s** (125 MHz, $CDCl_3$)

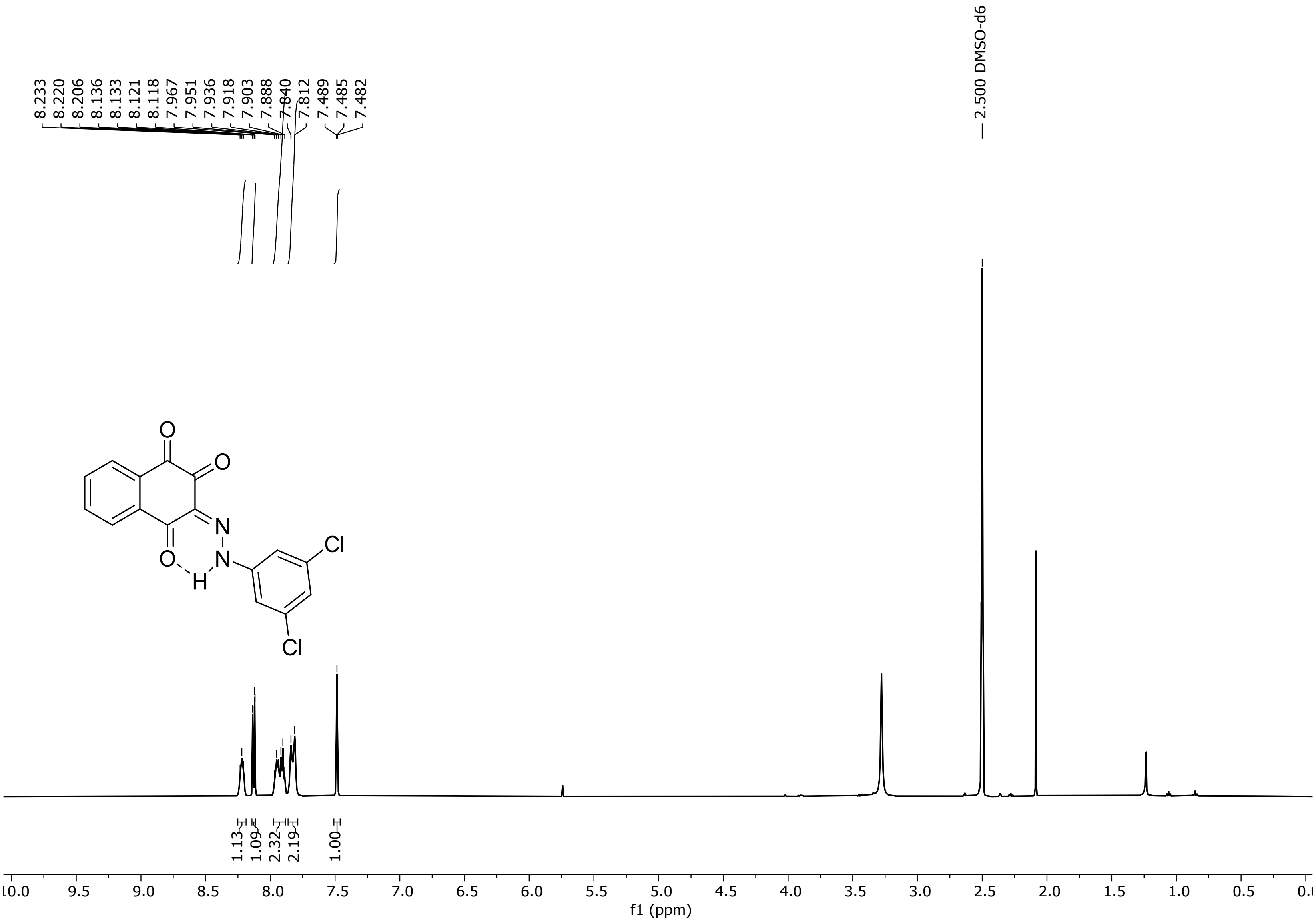

Figure S39. ^{1}H NMR spectrum of **3t** (500 MHz, DMSOd6)

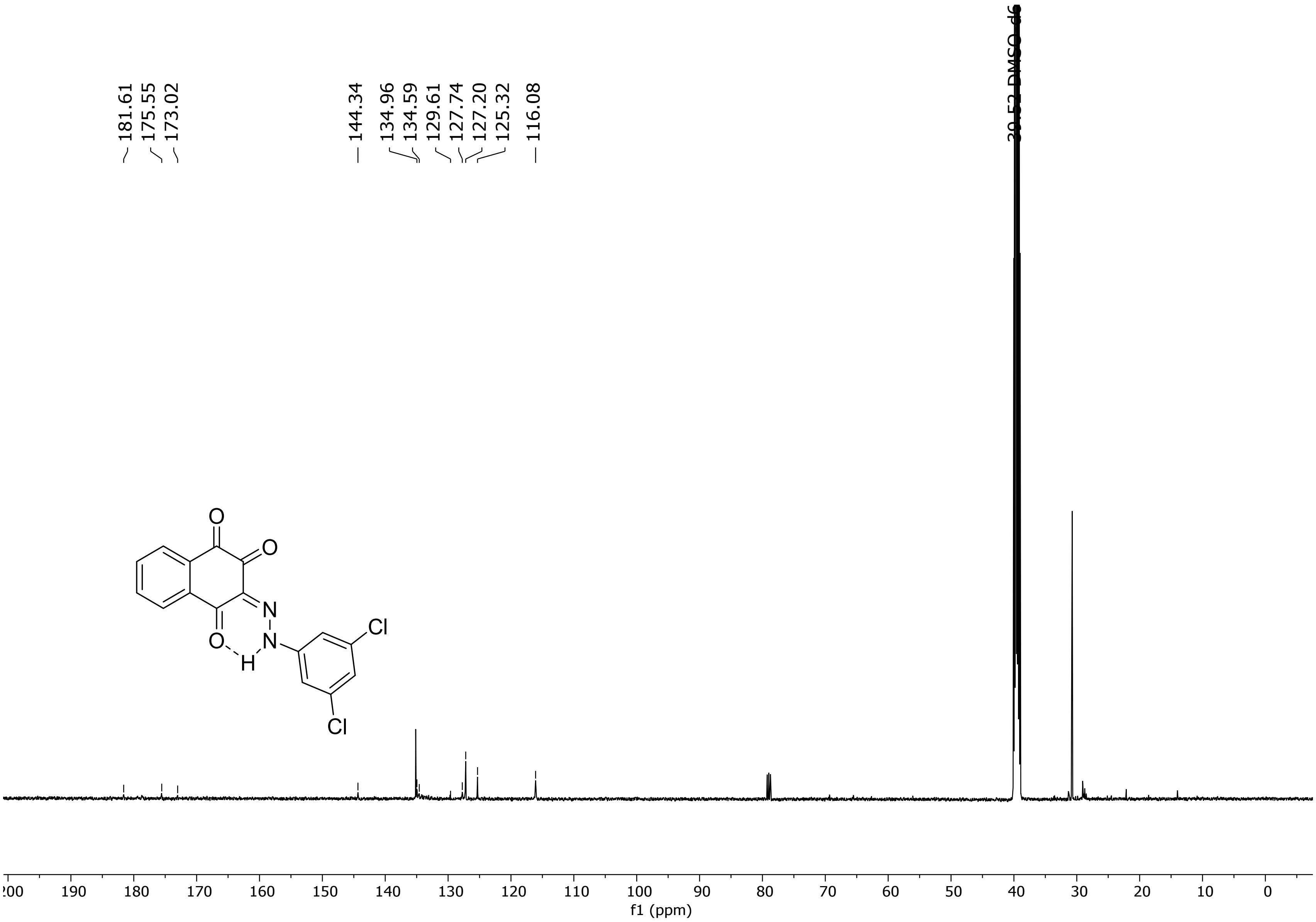

Figure S40. ^{13}C NMR spectrum of **3t** (125 MHz, DMSOd6)

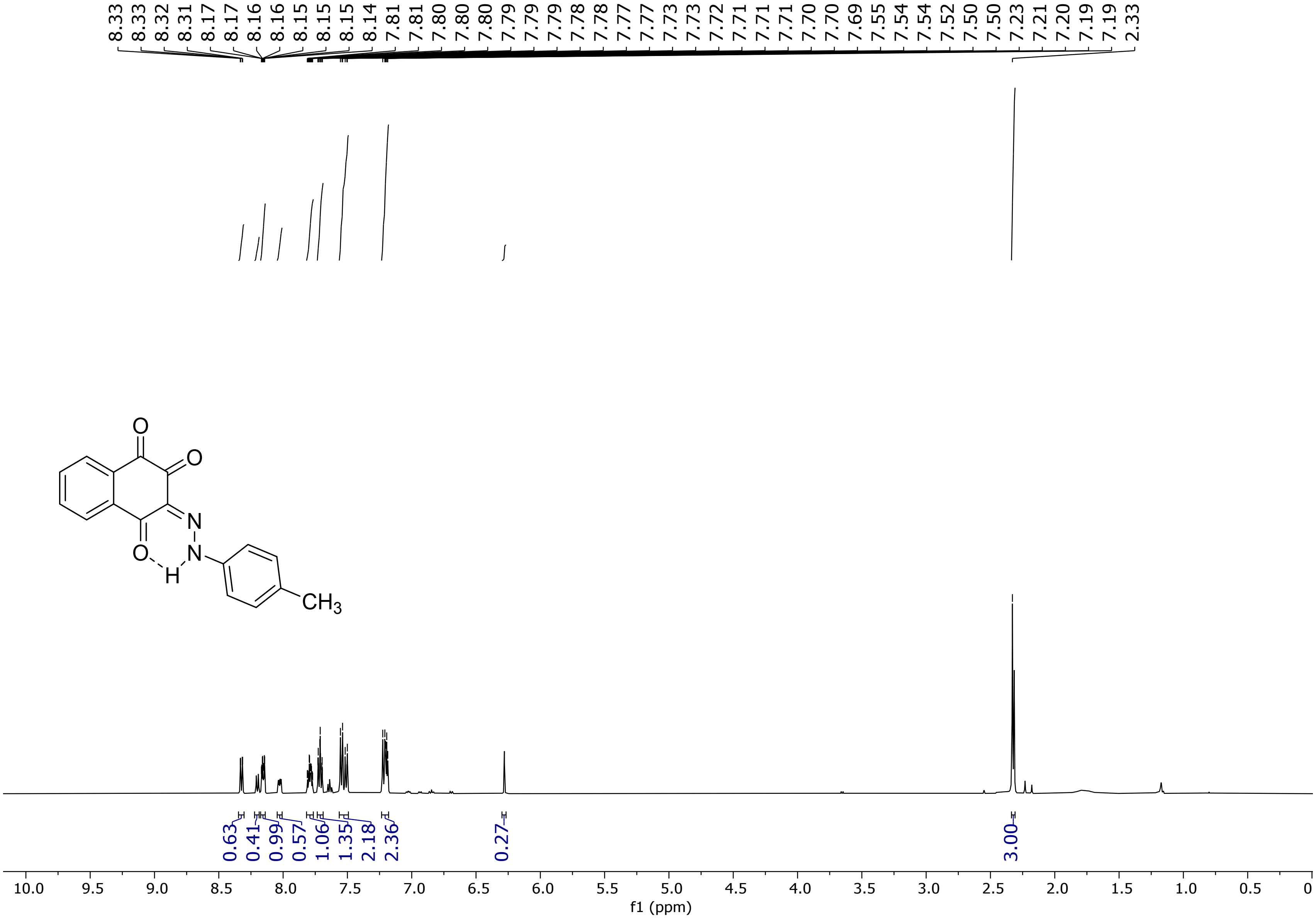

Figure S41. ^{1}H NMR spectrum of **3u** (500 MHz, $CDCl_3$)

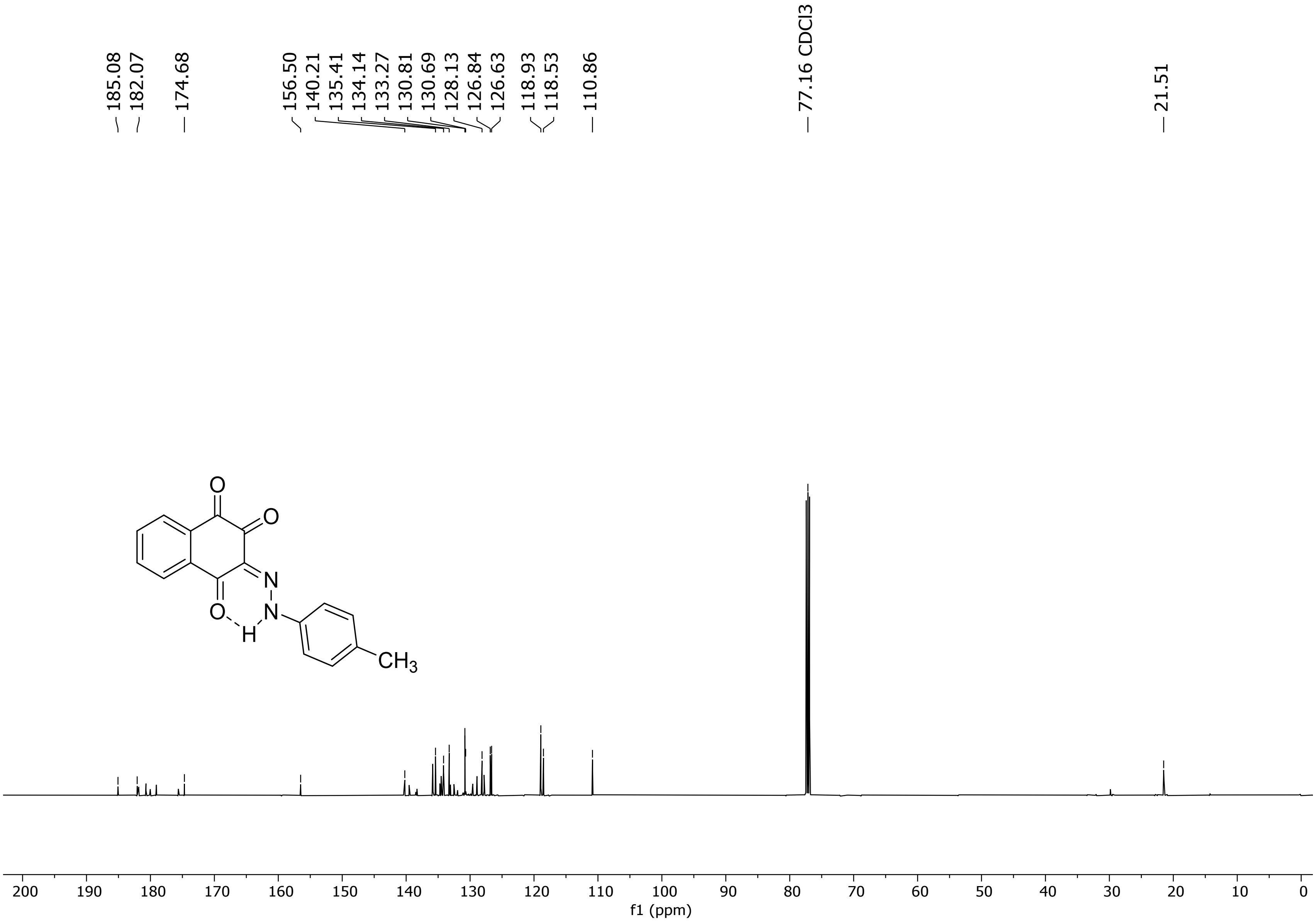

Figure S42. ^{13}C NMR spectrum of **3u** (125 MHz, $CDCl_3$)

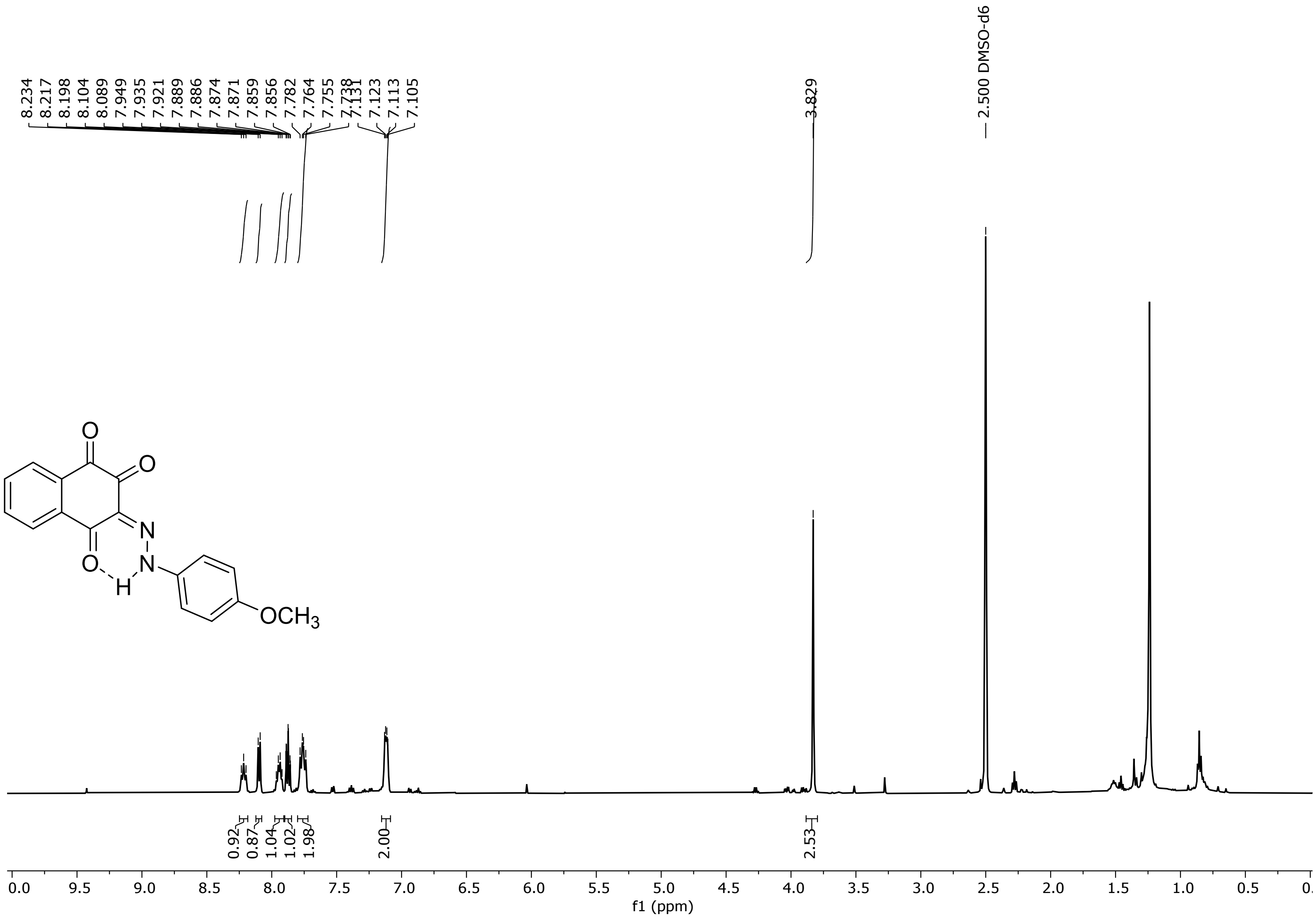

Figure S43. ¹H NMR spectrum of **3v** (500 MHz, DMSOd6)

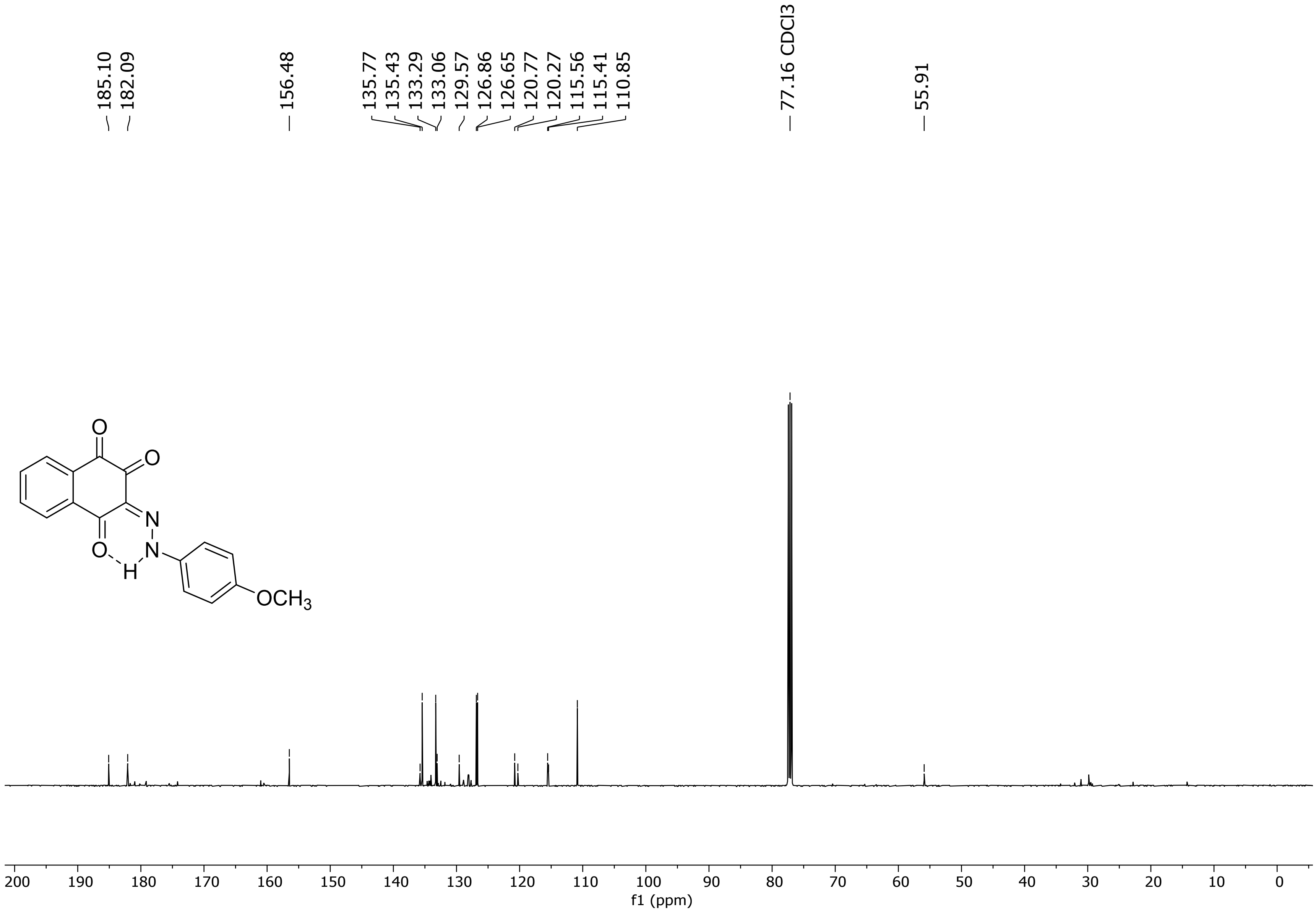

Figure S44. ^{13}C NMR spectrum of **3v** (125 MHz, $CDCl_3$)

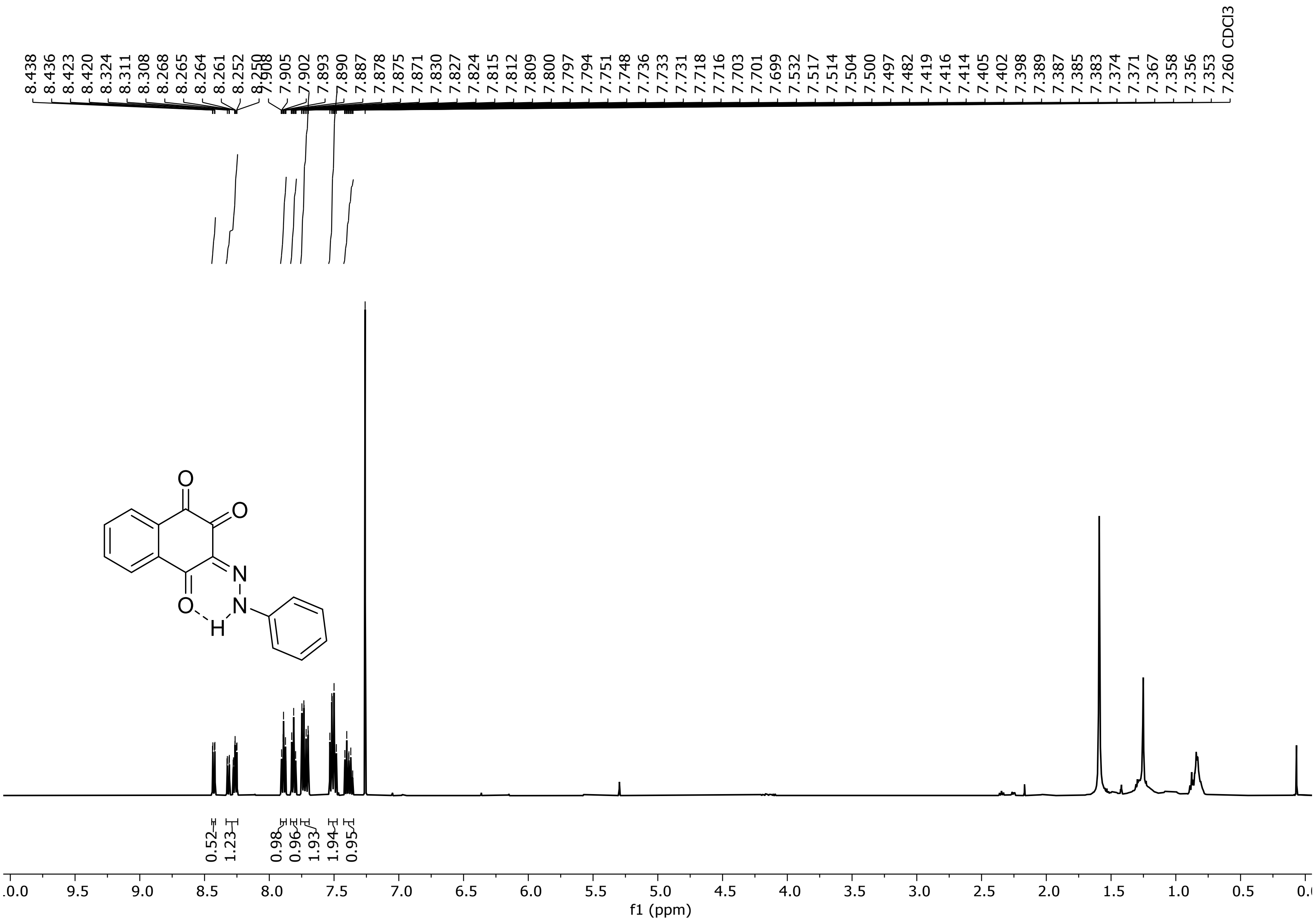

Figure S45. ^{1}H NMR spectrum of **3w** (500 MHz, $CDCl_3$)

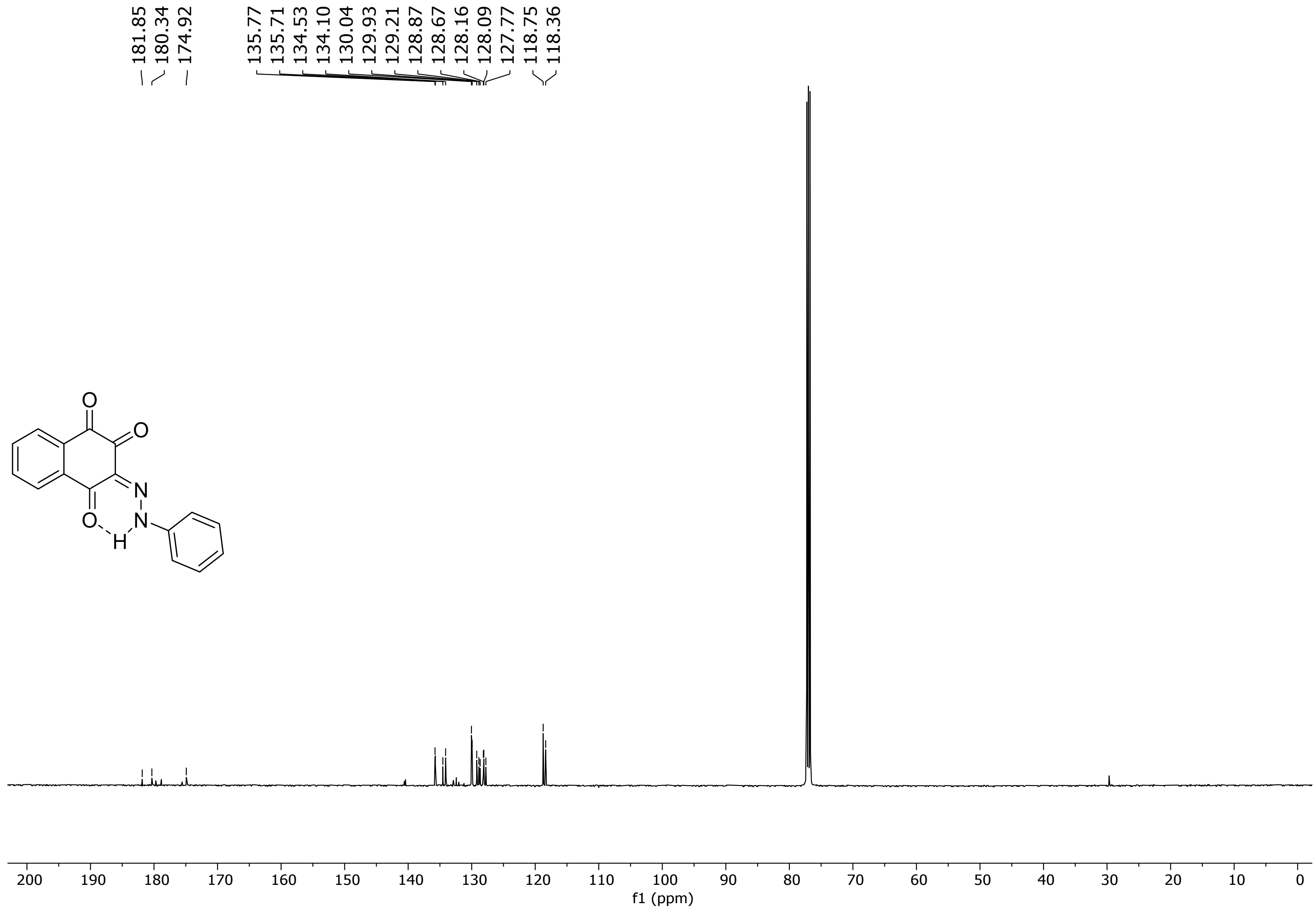

Figure S46. ^{13}C NMR spectrum of **3w** (125 MHz, $CDCl_3$)

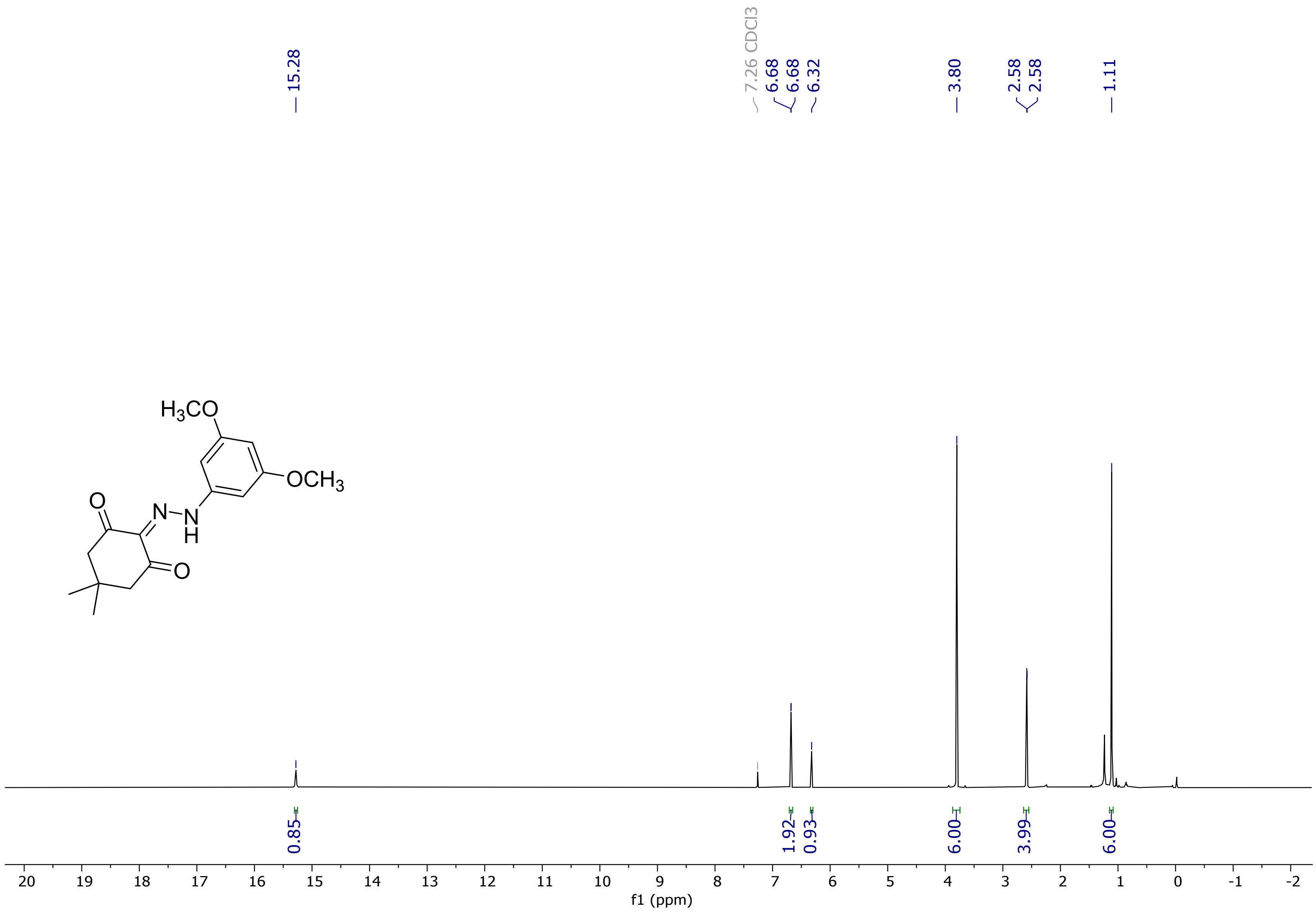

Figure 47. ^{1}H NMR spectrum of 7a (500 MHz, $CDCl_3$)

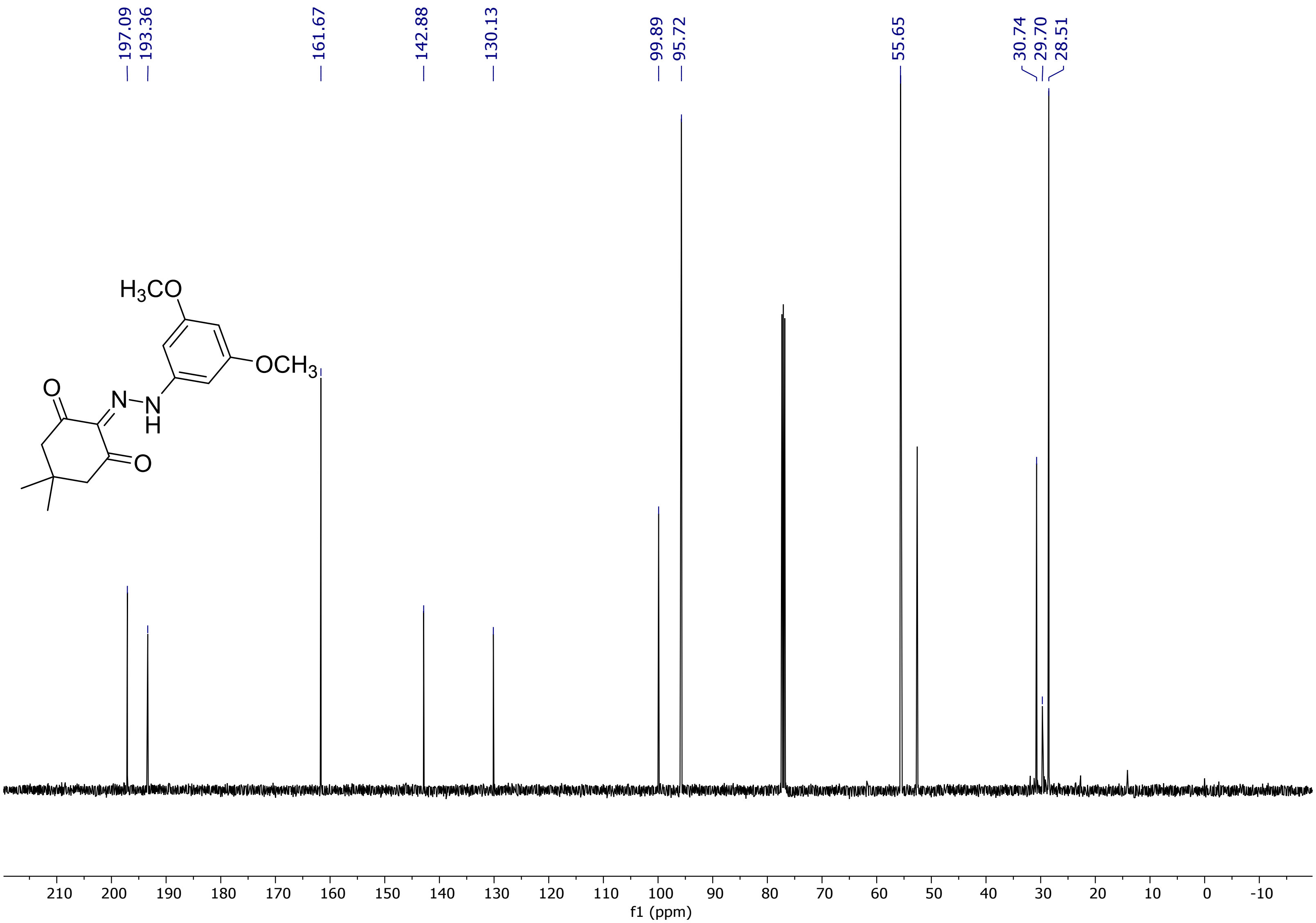

Figure S48. ^{13}C NMR spectrum of **7a** (125 MHz, $CDCl_3$)

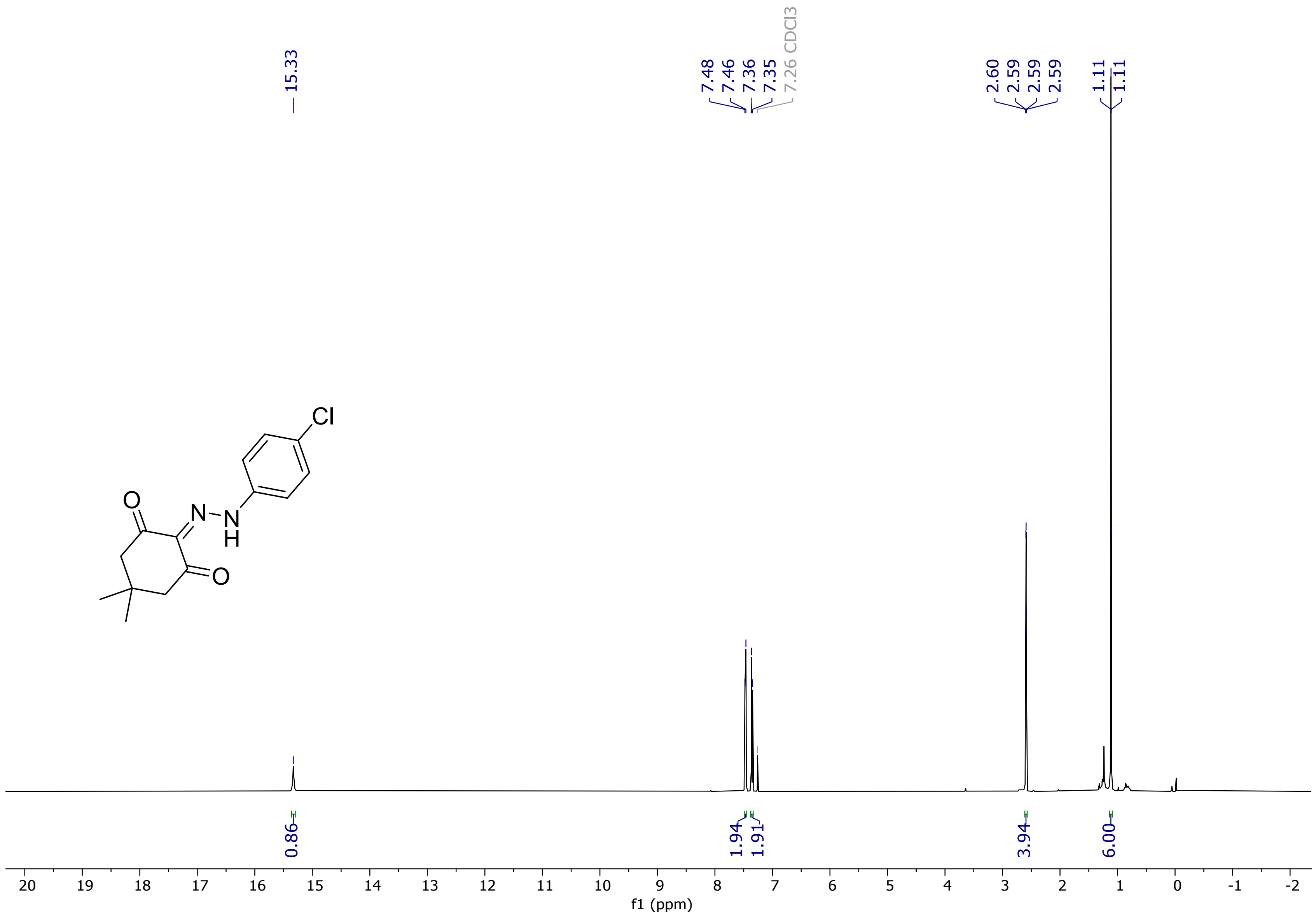

Figure S49. ^{1}H NMR spectrum of **7b** (500 MHz, $CDCl_3$)

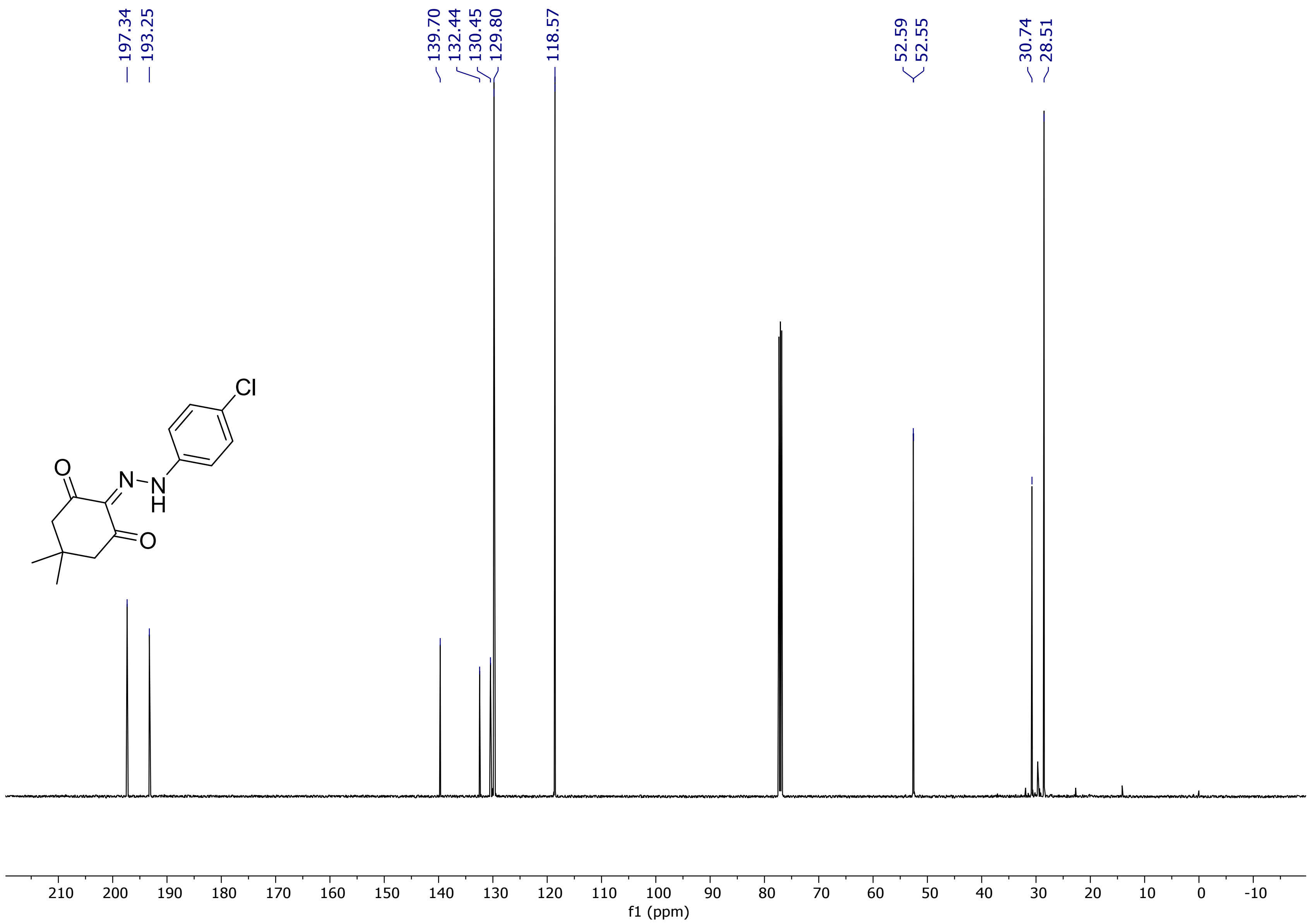

Figure S50. ^{13}C NMR spectrum of **7b** (125 MHz, $CDCl_3$)

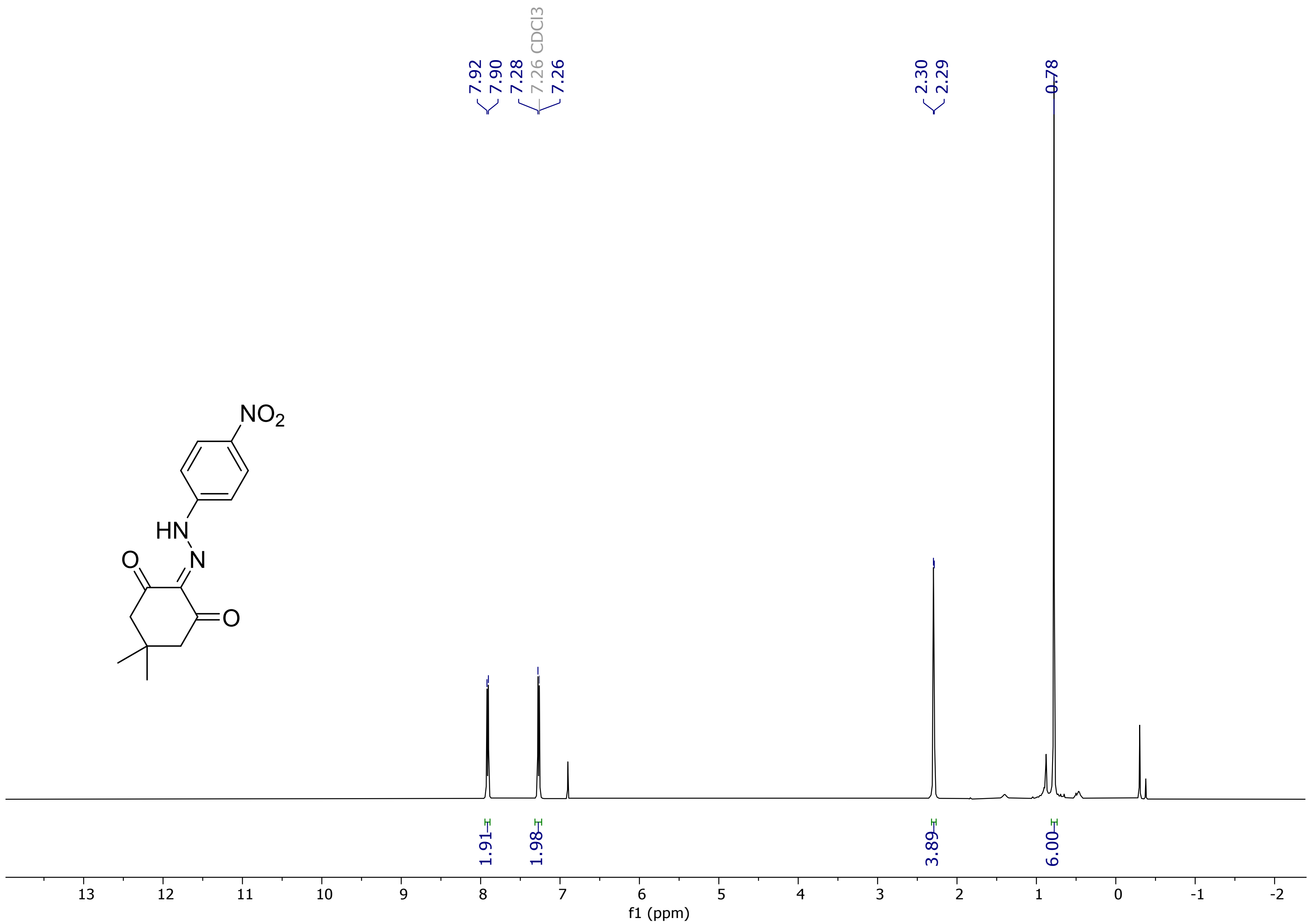

Figure S51. ^{1}H NMR spectrum of **7c** (500 MHz, $CDCl_3$)

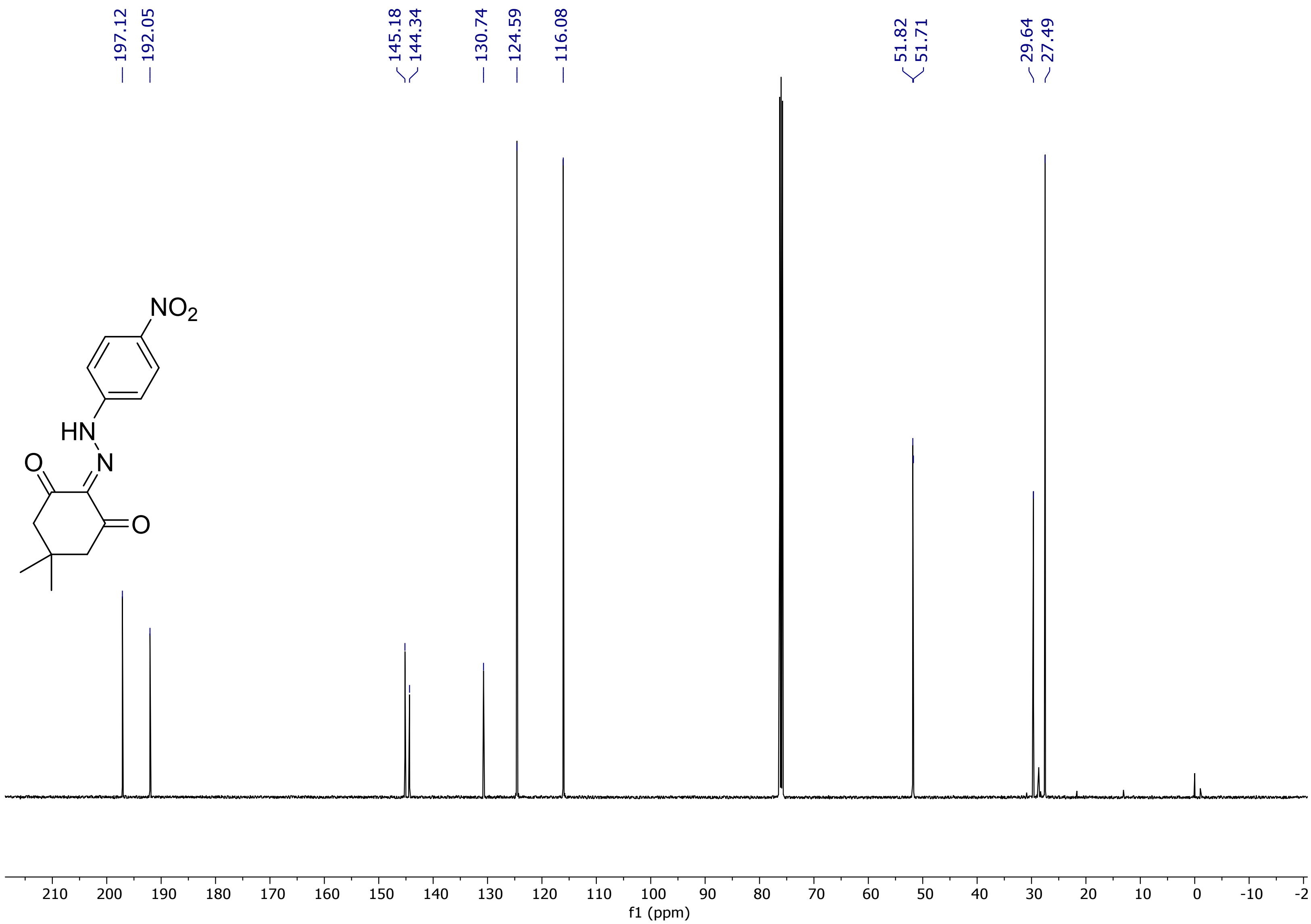

Figure S52. ^{13}C NMR spectrum of **7c** (125 MHz, $CDCl_3$)

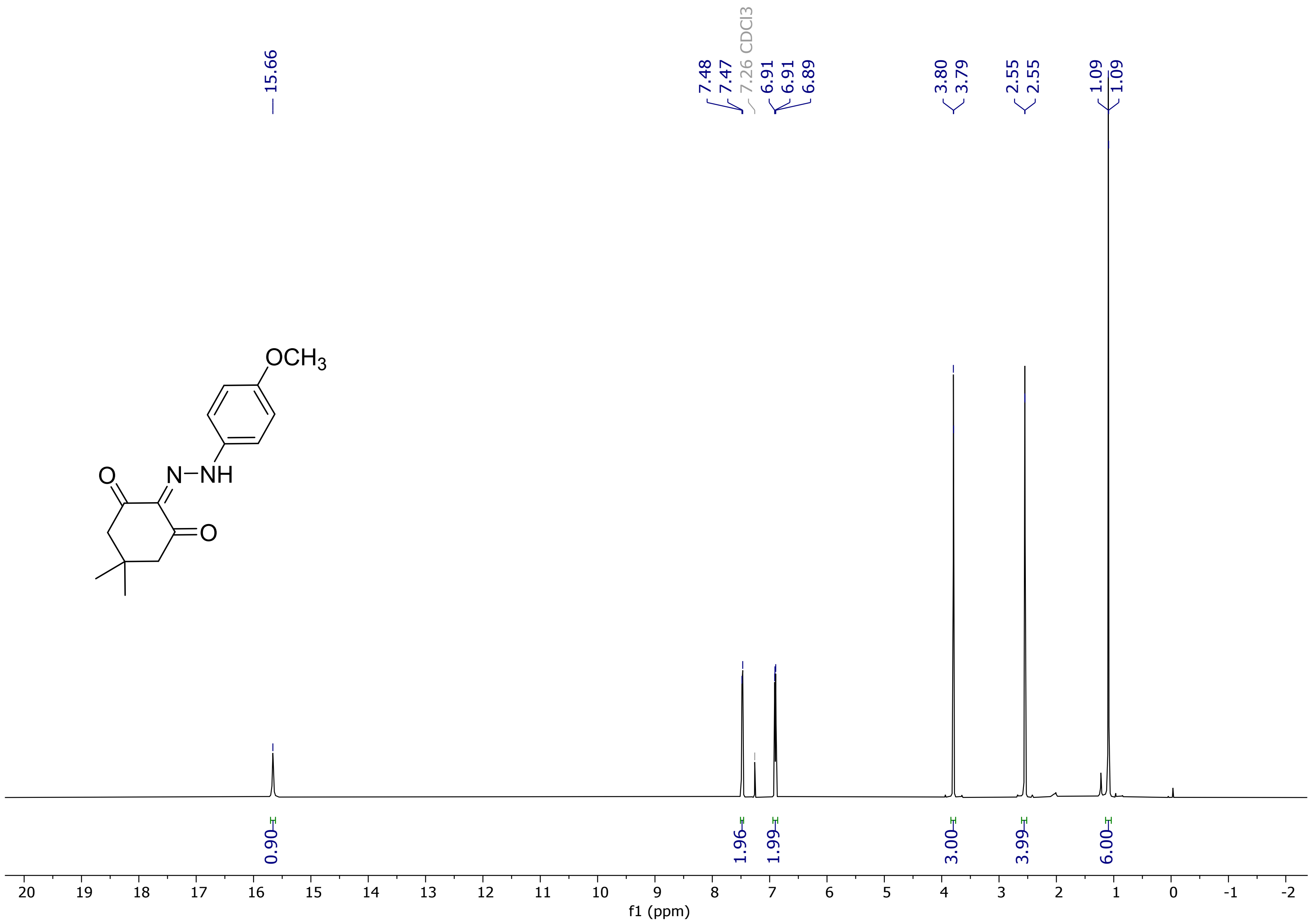

Figure S53. ^{1}H NMR spectrum of **7d** (500 MHz, $CDCl_3$)

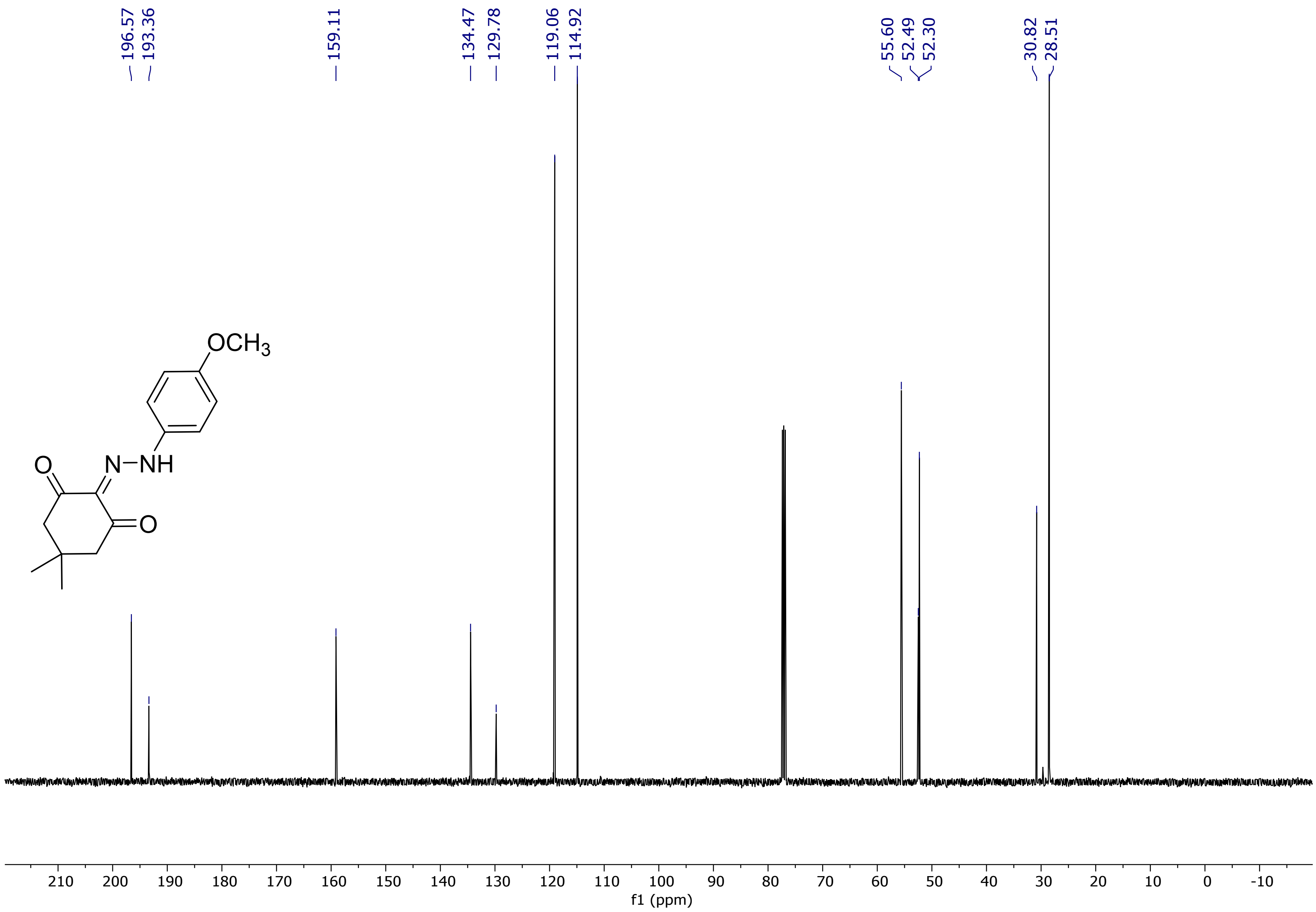

Figure S54. ^{13}C NMR spectrum of **7d** (125 MHz, $CDCl_3$)

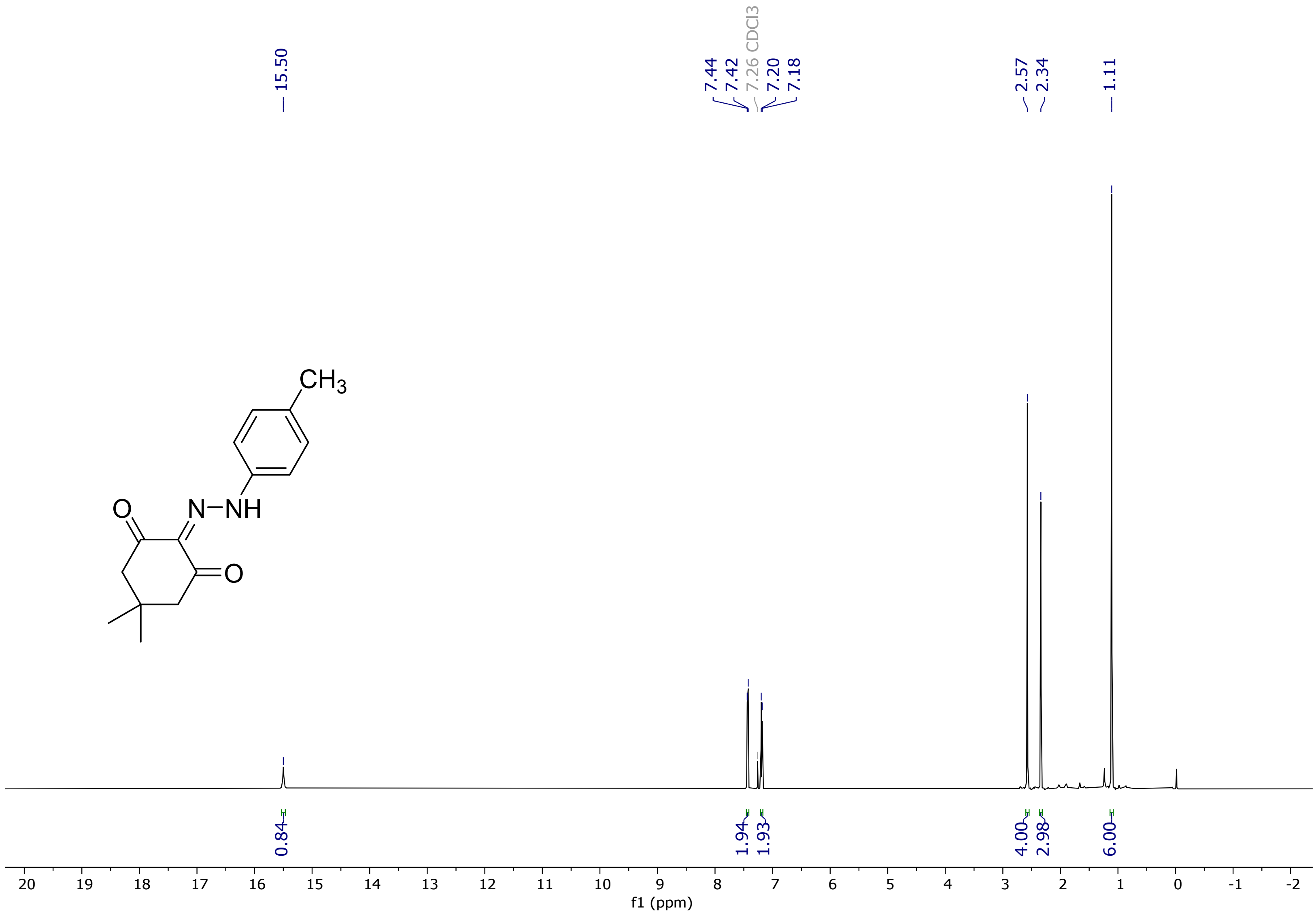

Figure S55. ^{1}H NMR spectrum of **7e** (500 MHz, $CDCl_3$)

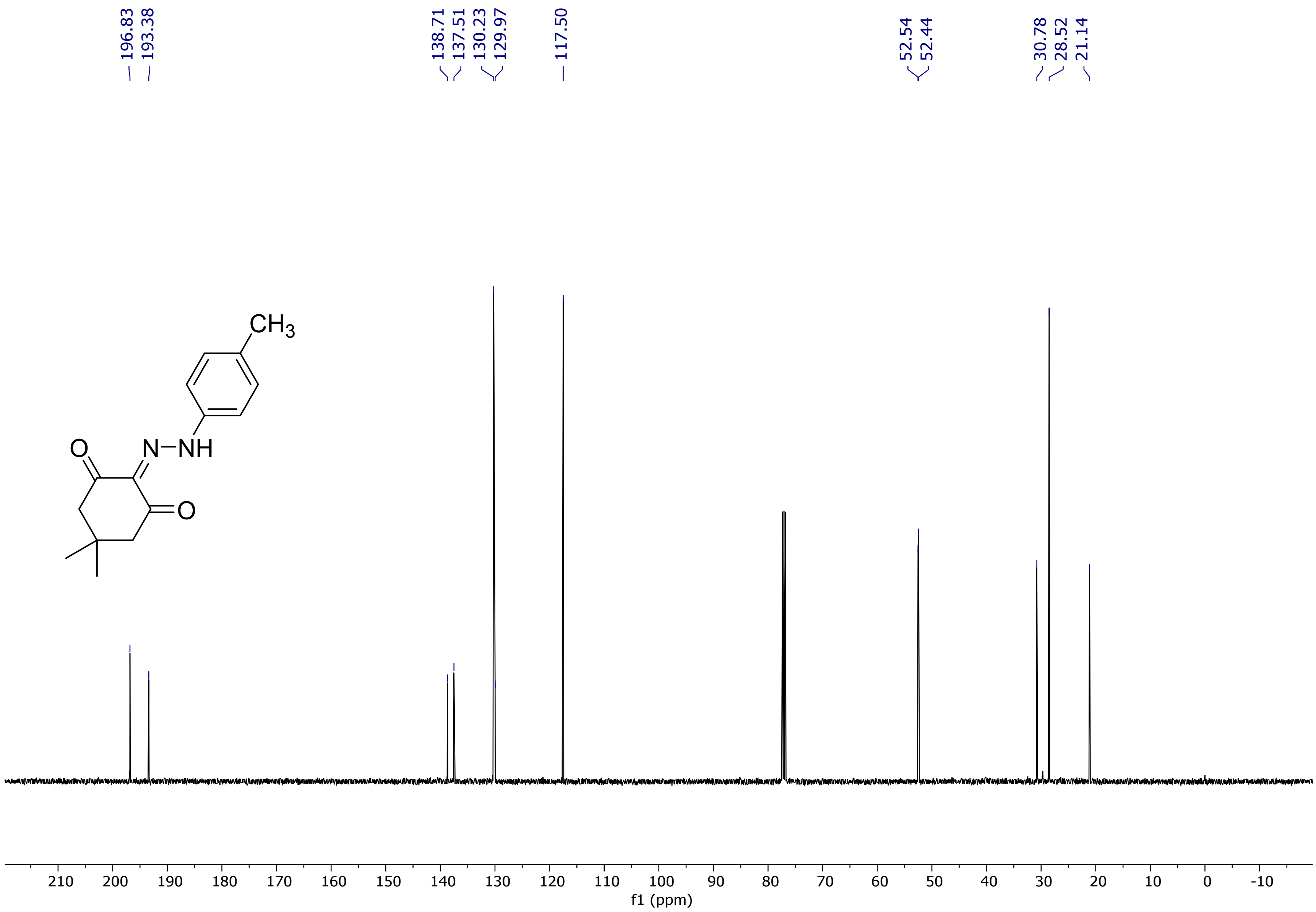

Figure S56. ^{13}C NMR spectrum of **7e** (125 MHz, $CDCl_3$)

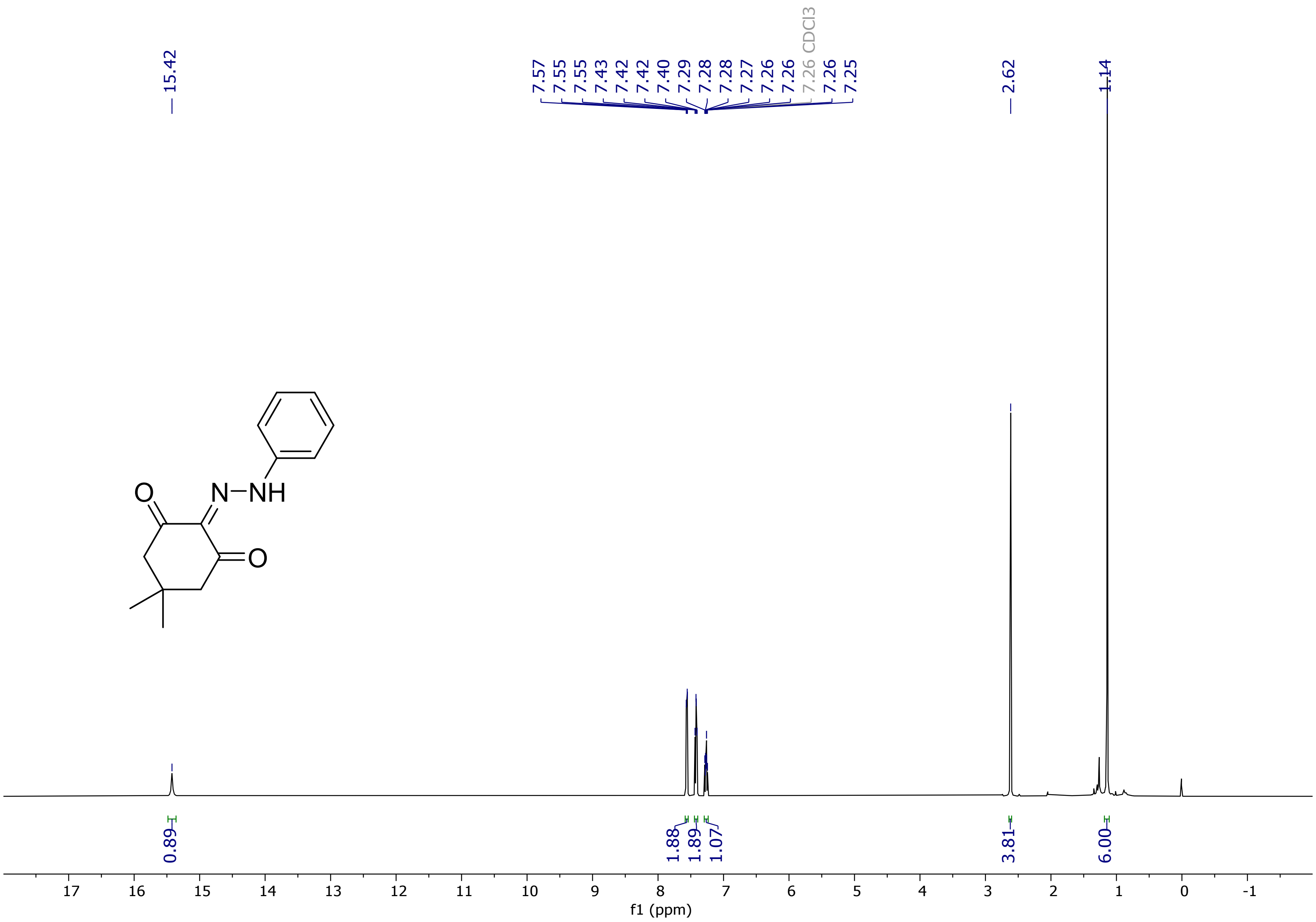

Figure S57. ^{1}H NMR spectrum of **7f** (500 MHz, $CDCl_3$)

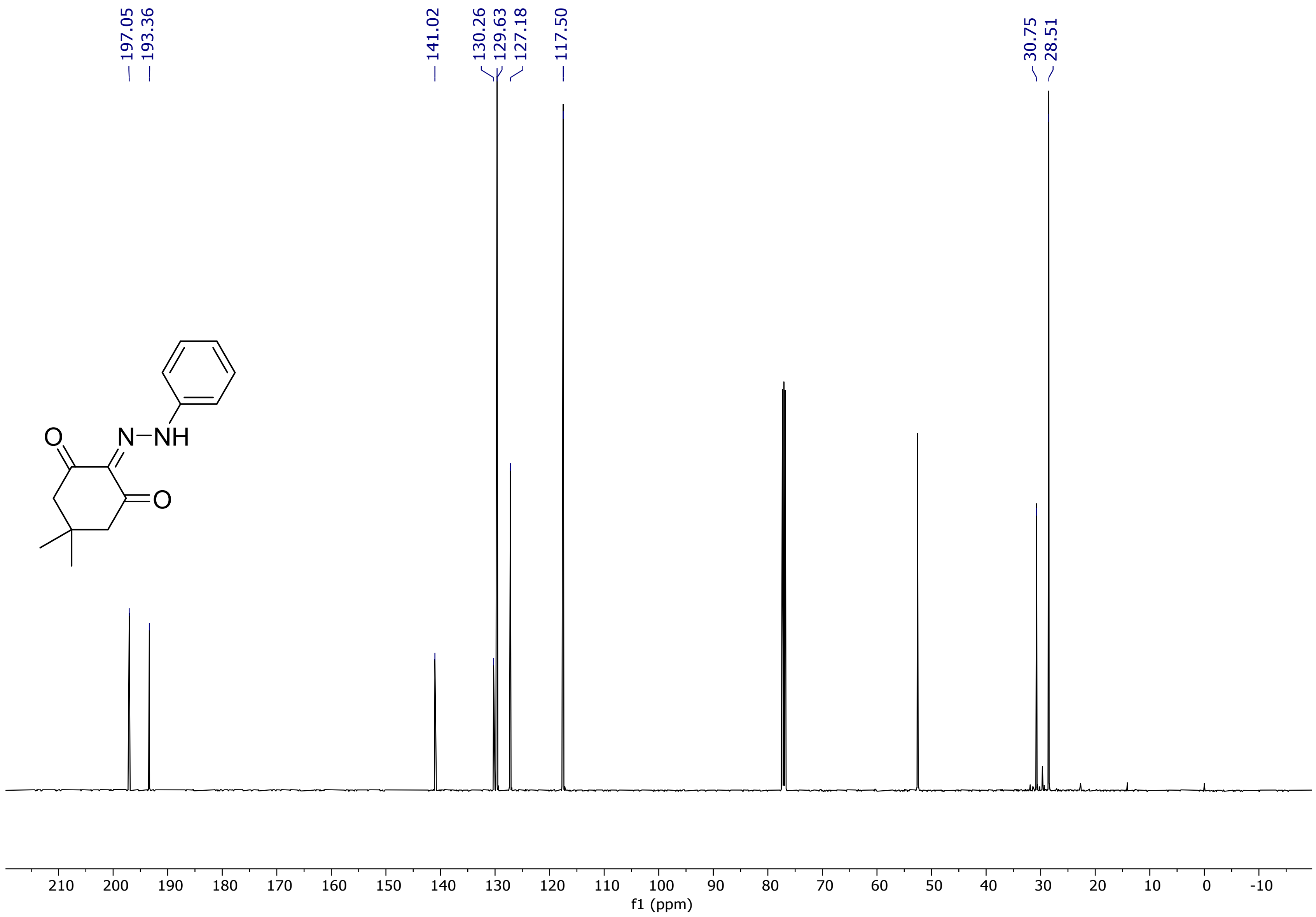

Figure S58. ^{13}C NMR spectrum of **7f** (125 MHz, $CDCl_3$)

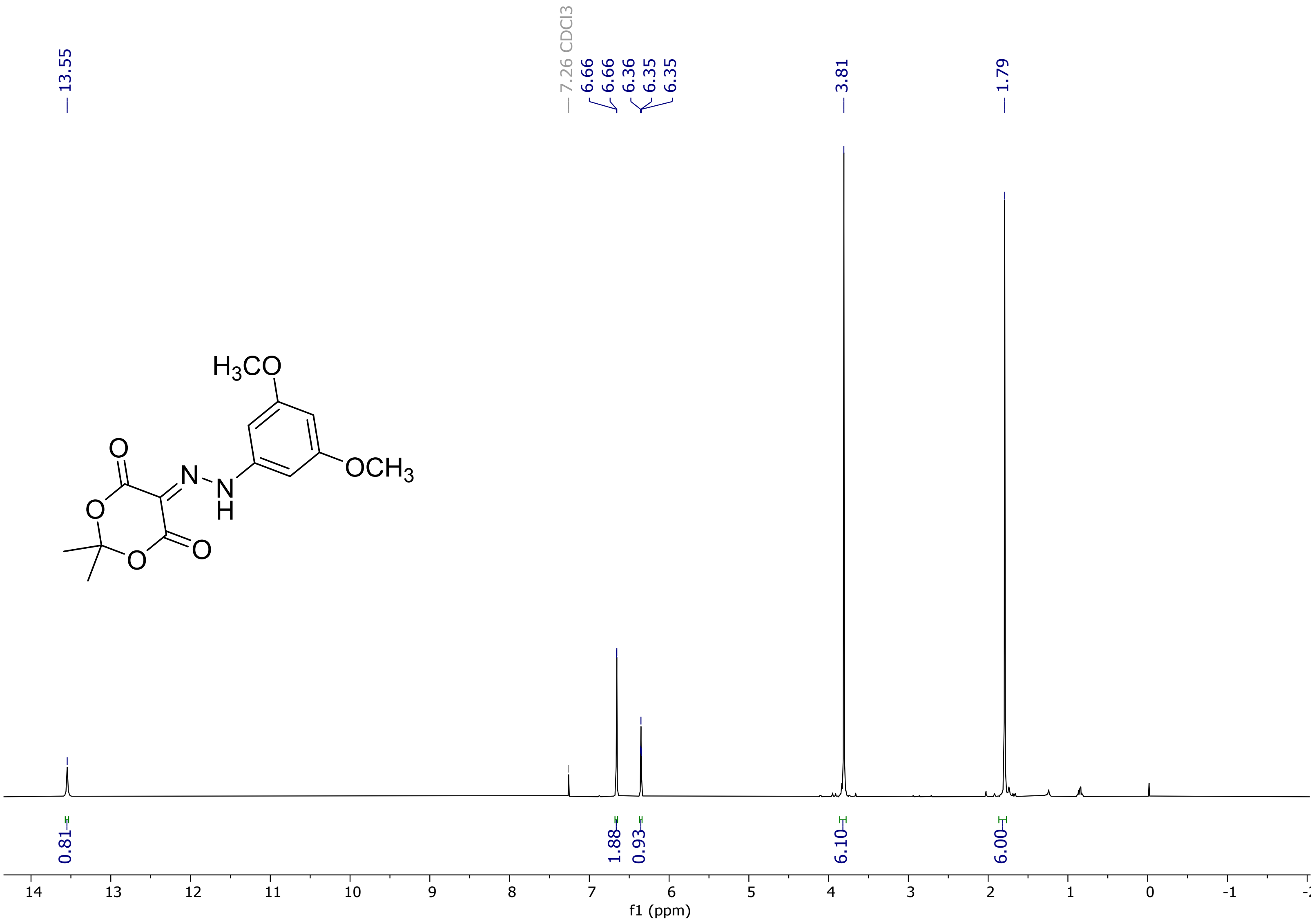

Figure S59. ^{1}H NMR spectrum of **7g** (500 MHz, $CDCl_3$)

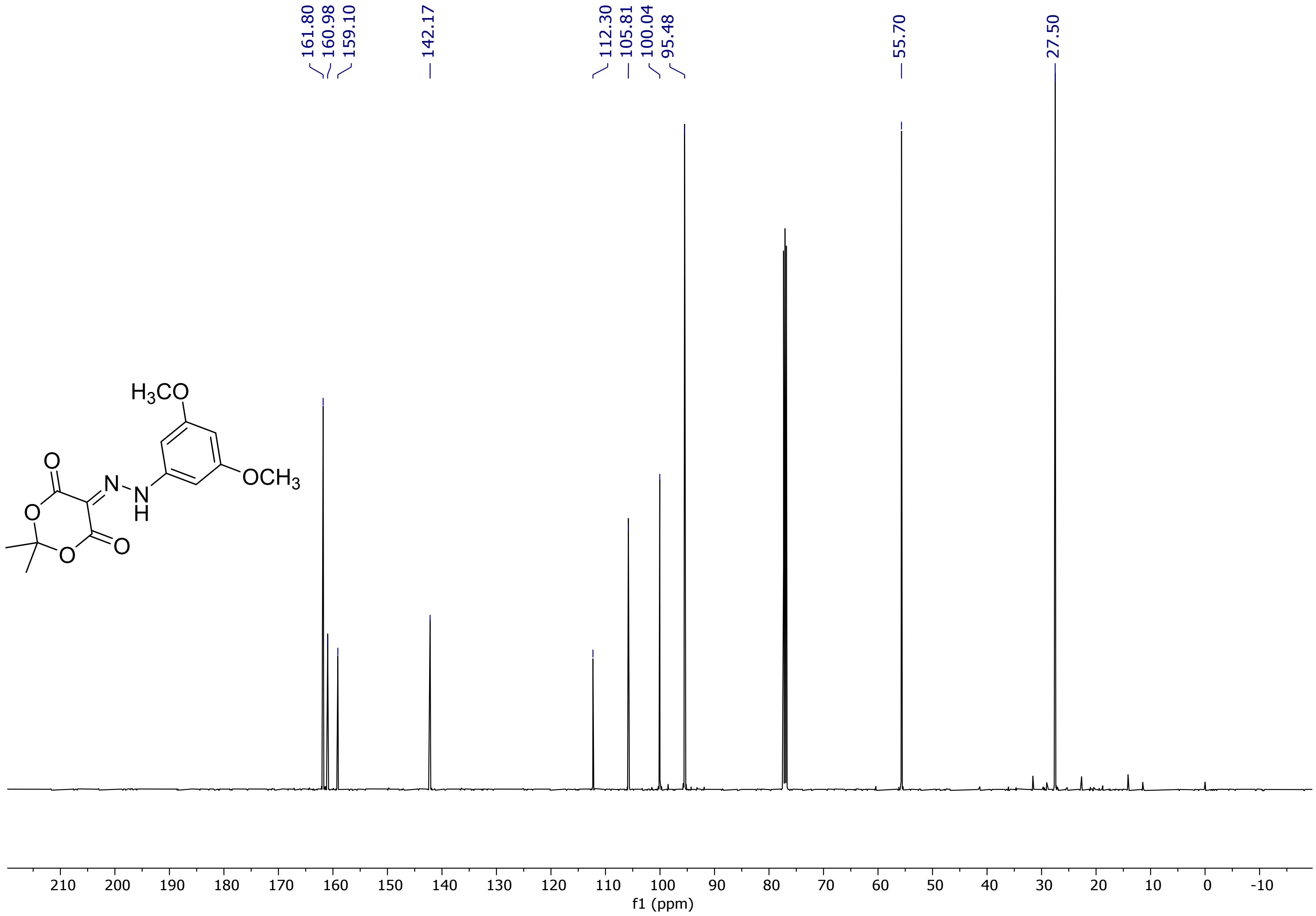

Figure S60. ^{13}C NMR spectrum of **7g** (125 MHz, $CDCl_3$)

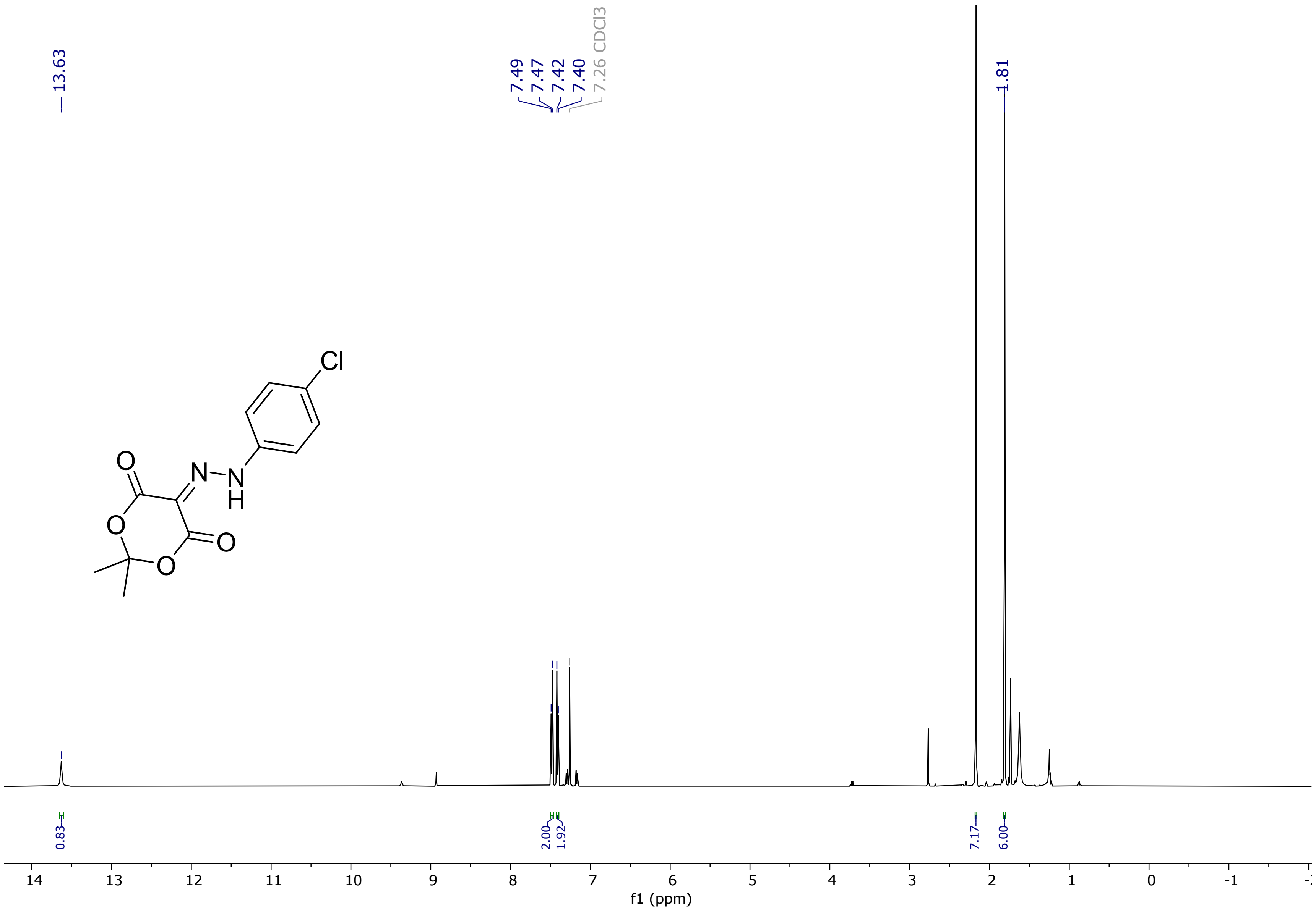

Figure S61. ^{1}H NMR spectrum of **7h** (500 MHz, $CDCl_3$)

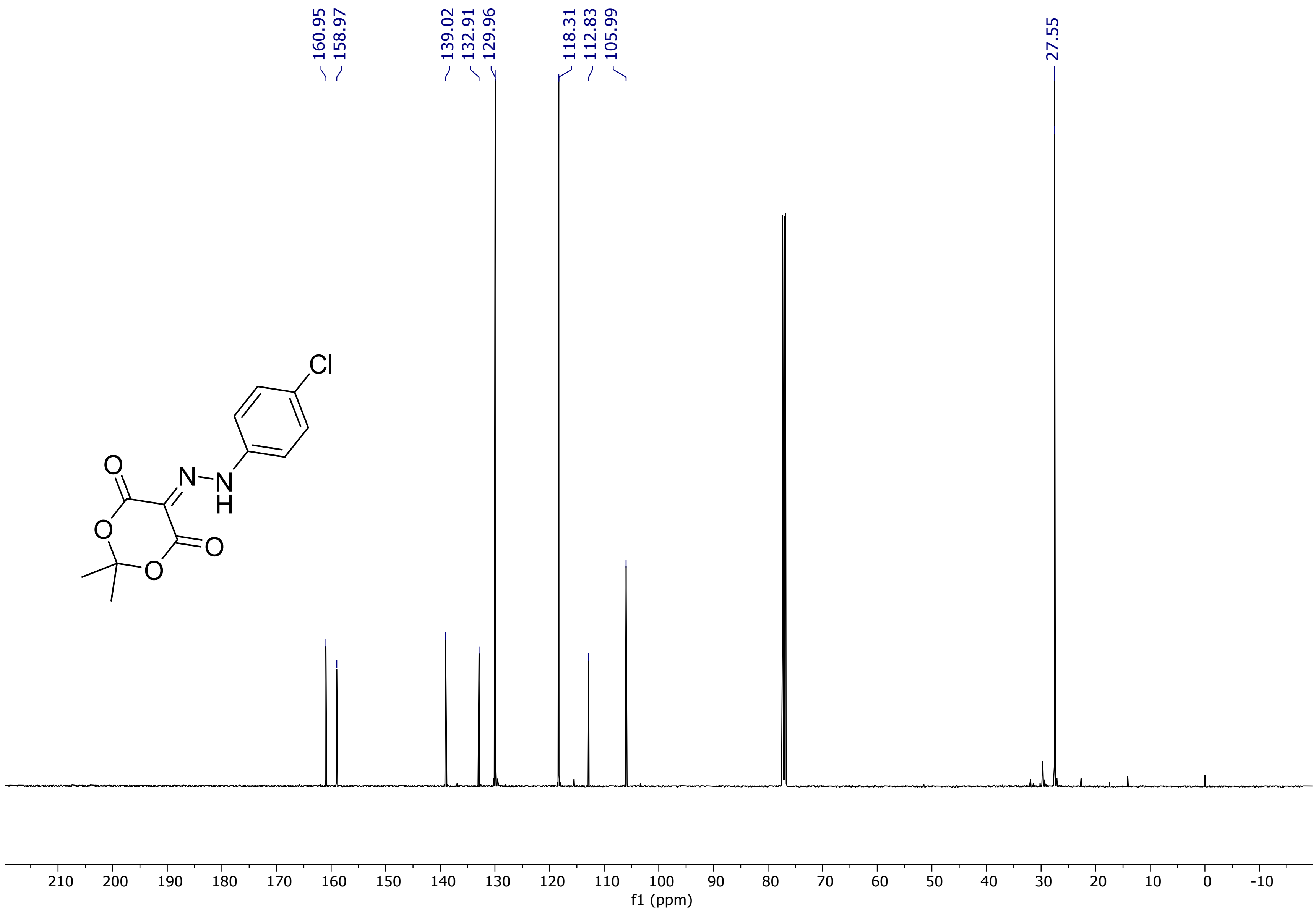

Figure S62. ^{13}C NMR spectrum of **7h** (125 MHz, $CDCl_3$)

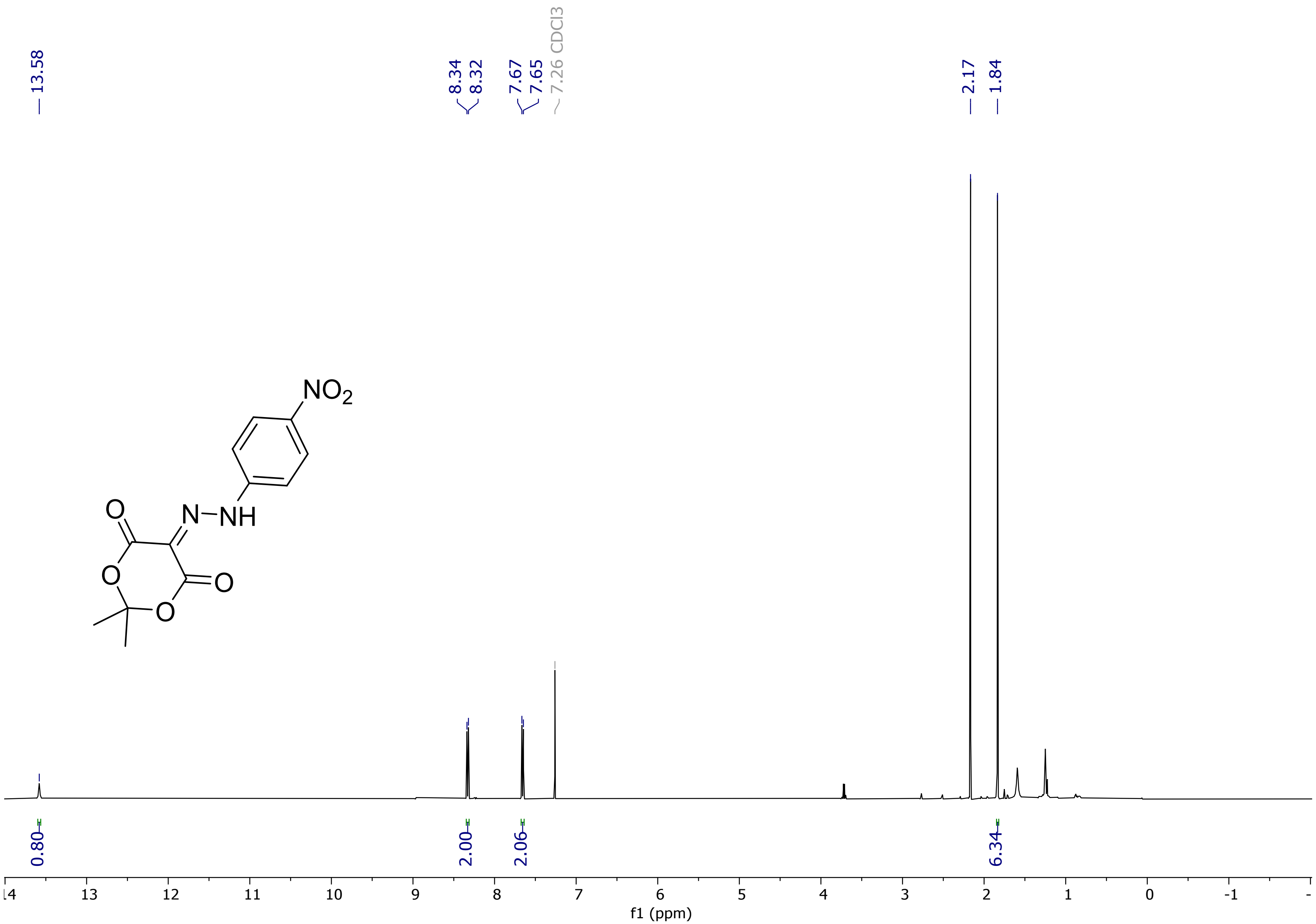

Figure S63. ^{1}H NMR spectrum of **7i** (500 MHz, $CDCl_3$)

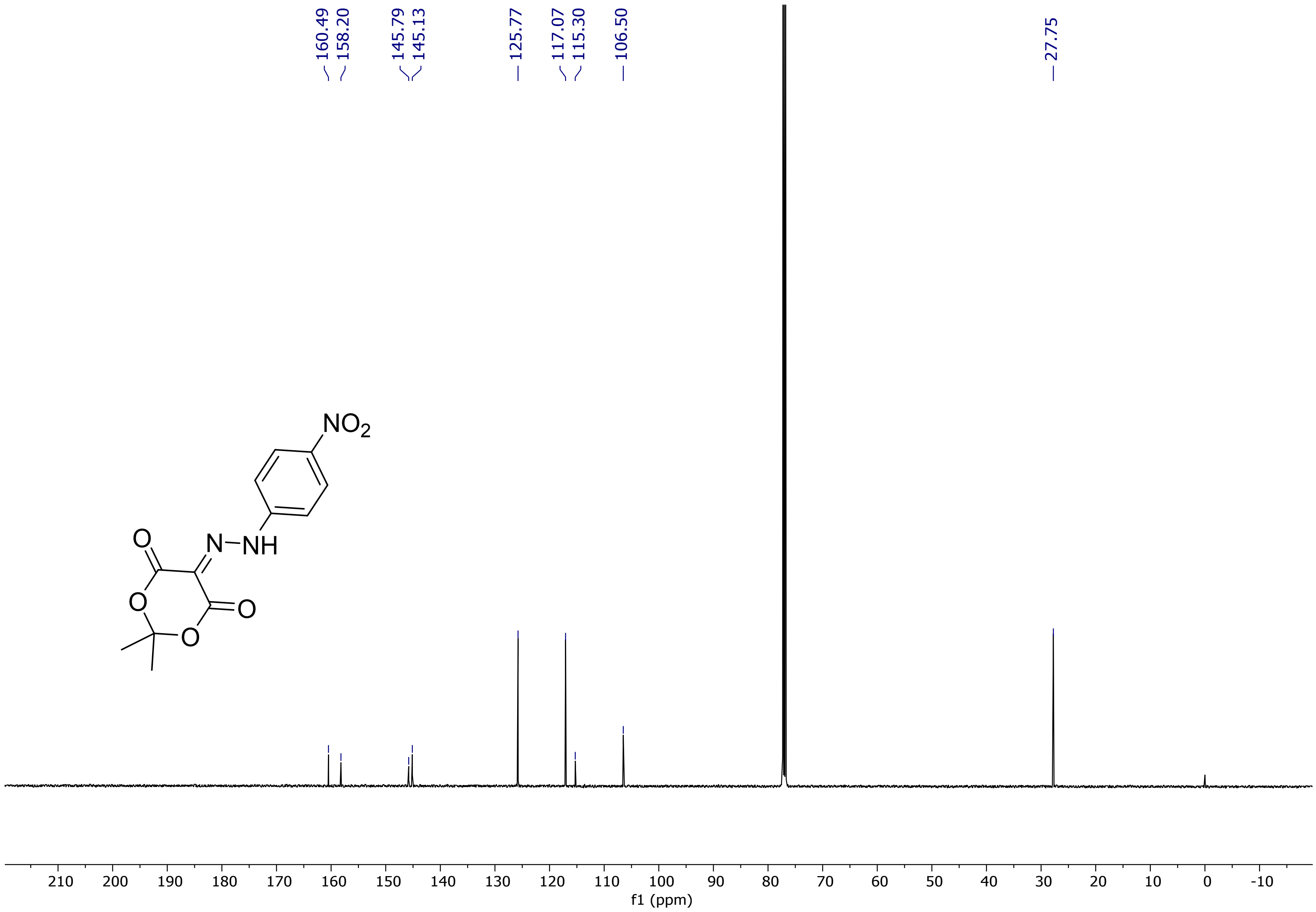

Figure S64. ^{13}C NMR spectrum of **7i** (125 MHz, $CDCl_3$)

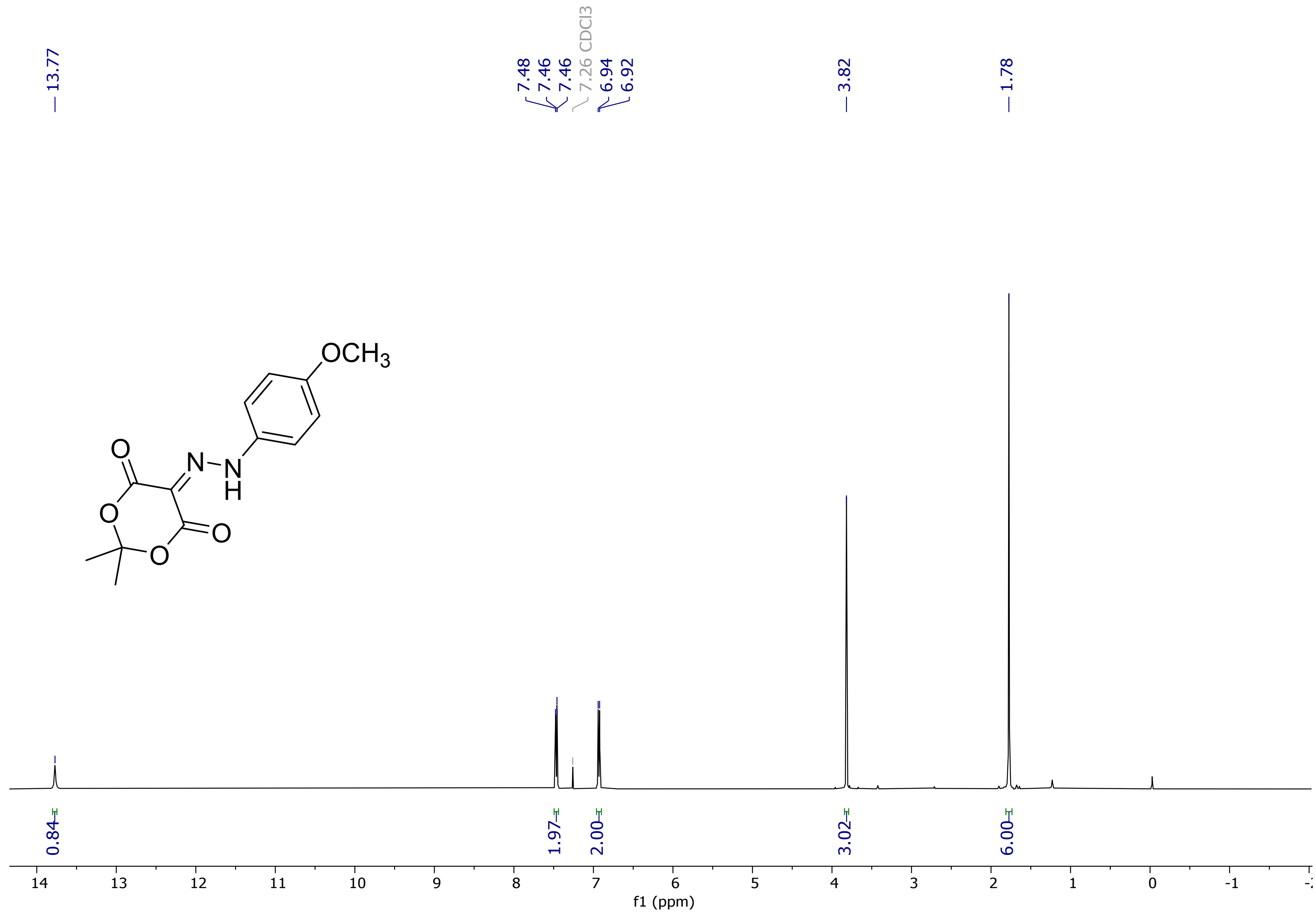

Figure S65. ^{1}H NMR spectrum of **7j** (500 MHz, $CDCl_3$)

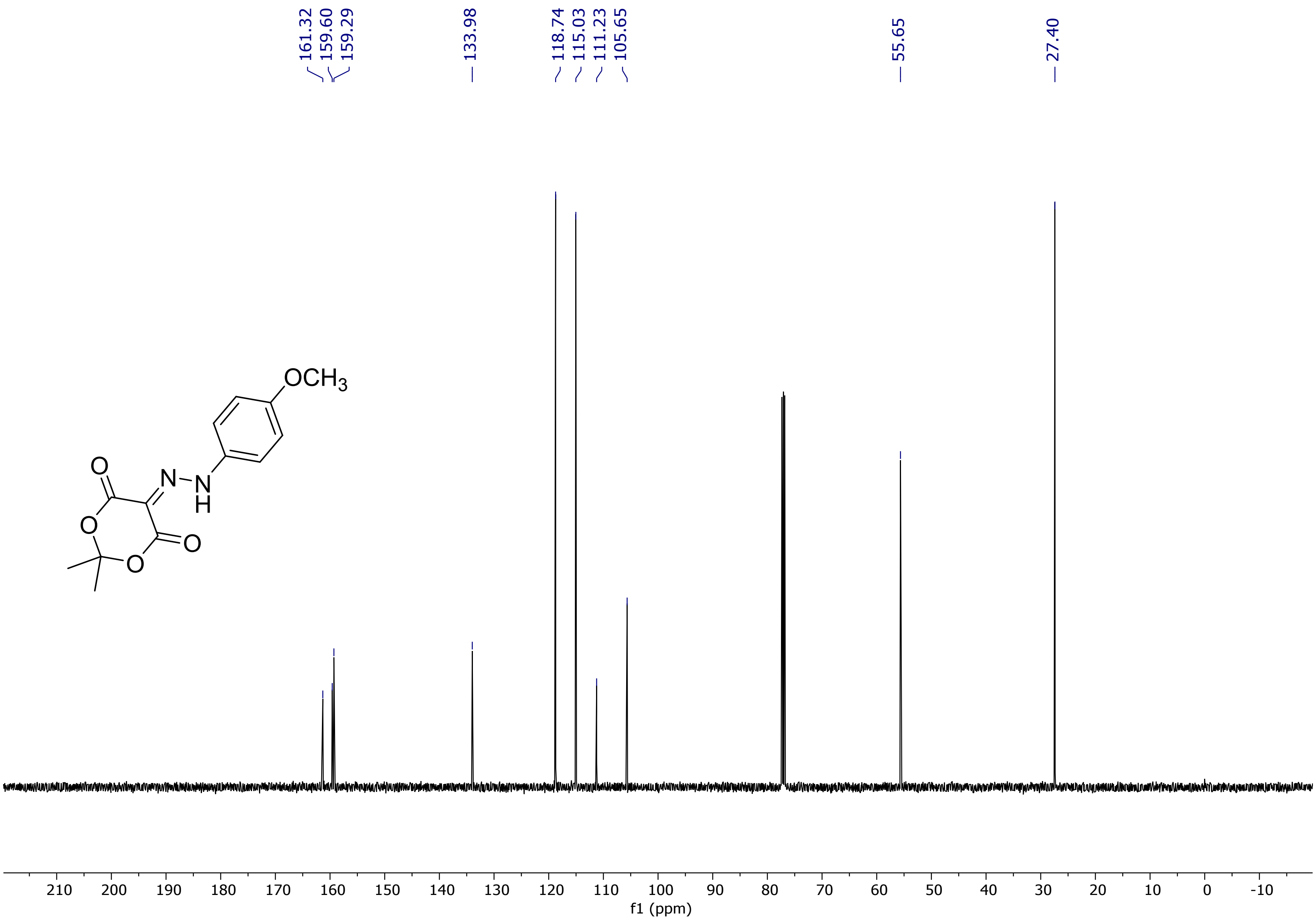

Figure S66. ^{13}C NMR spectrum of **7j** (125 MHz, $CDCl_3$)

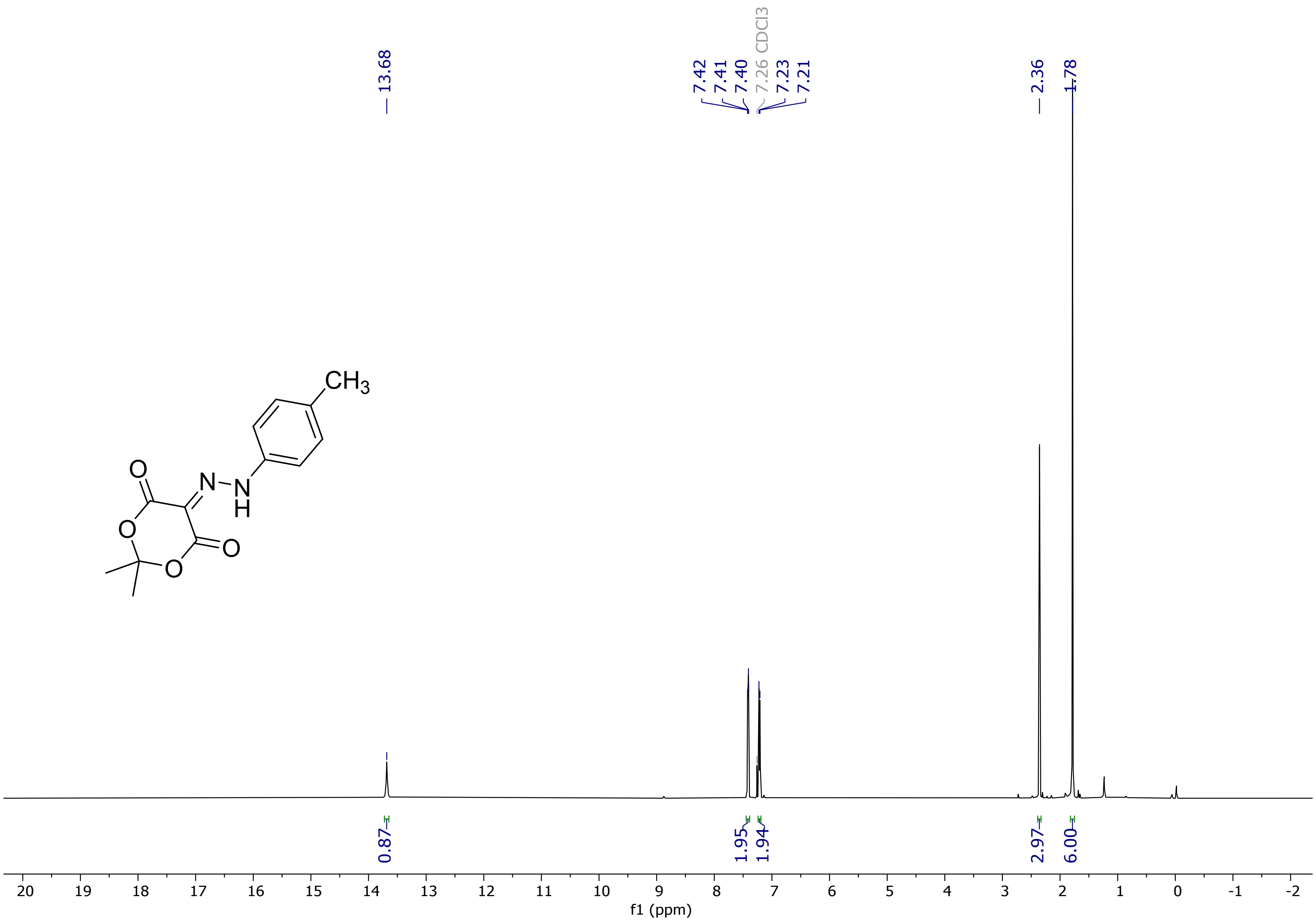

Figure S67. ^{1}H NMR spectrum of **7k** (500 MHz, $CDCl_3$)

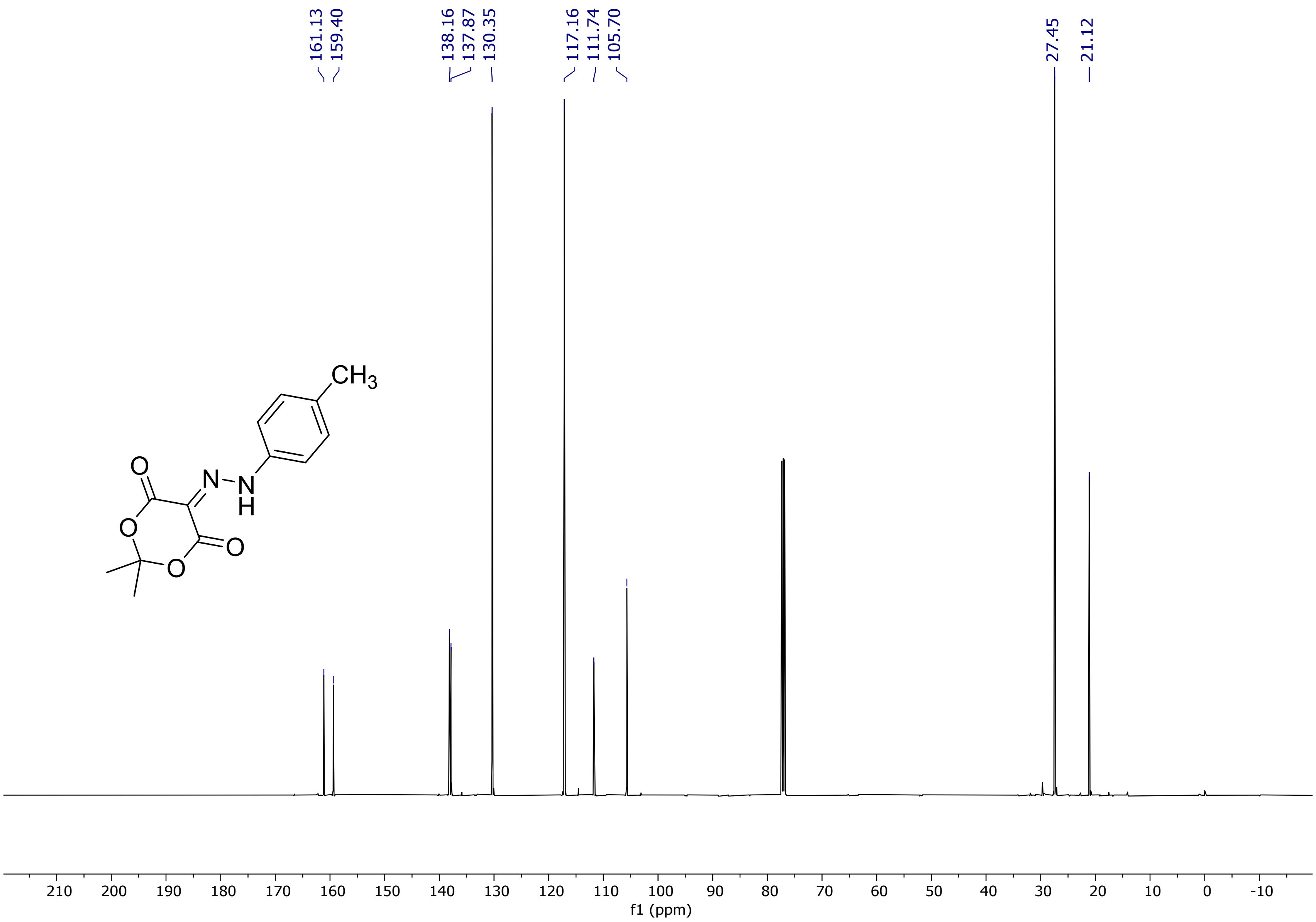

Figure S68. ^{13}C NMR spectrum of **7k** (125 MHz, $CDCl_3$)

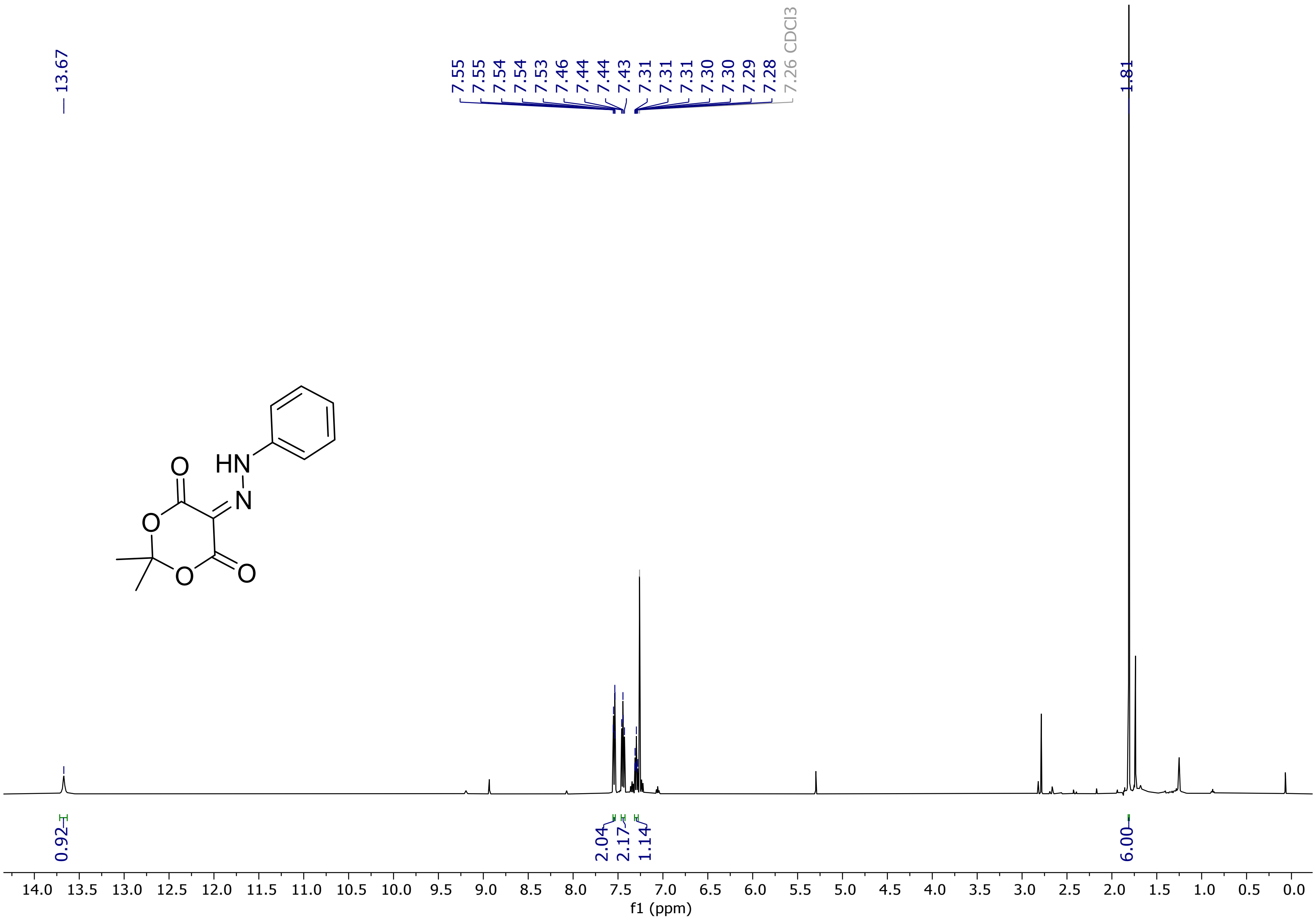

Figure S69. ^{1}H NMR spectrum of **7l** (500 MHz, $CDCl_3$)

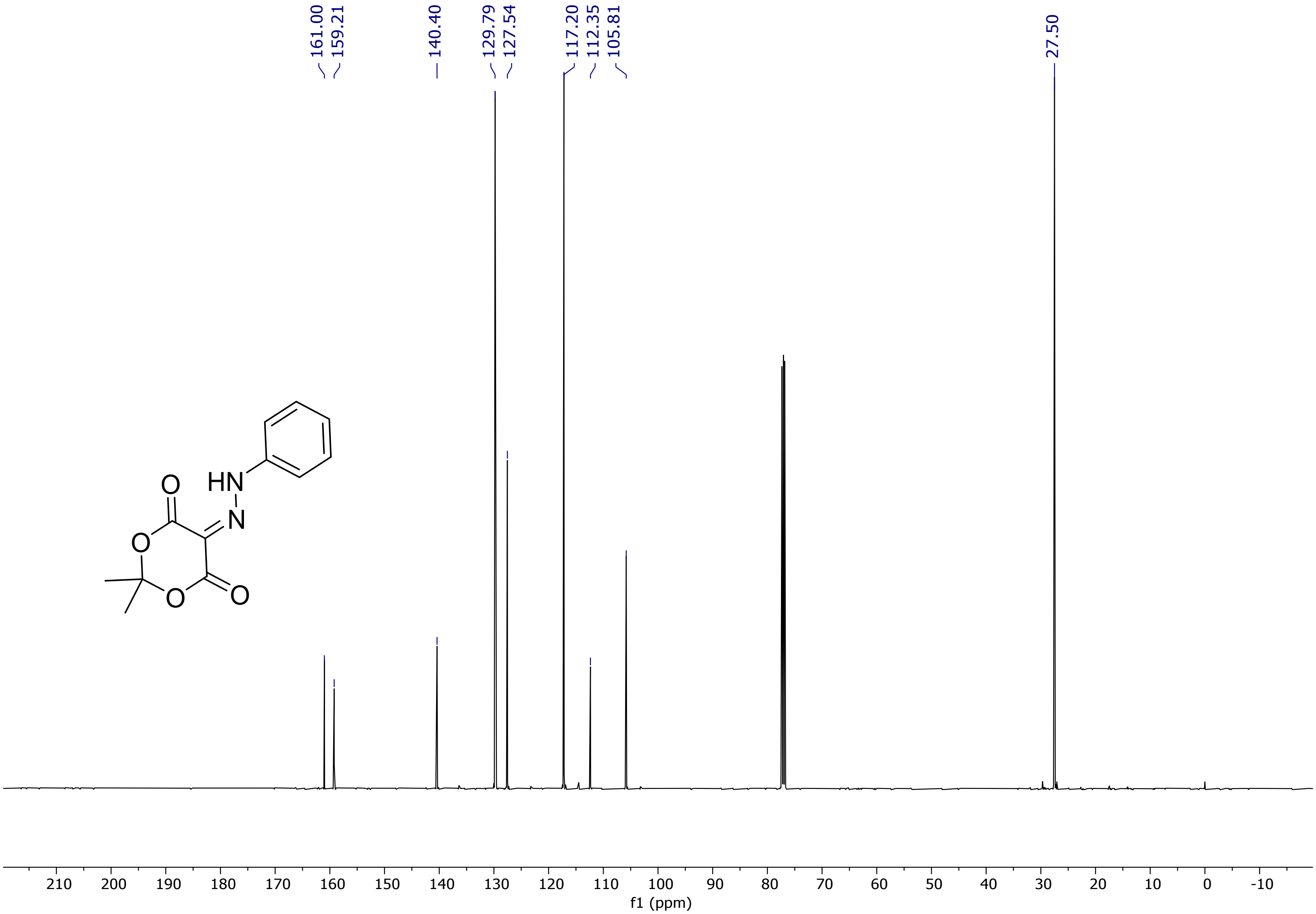

Figure S70. ^{13}C NMR spectrum of **7l** (125 MHz, $CDCl_3$)

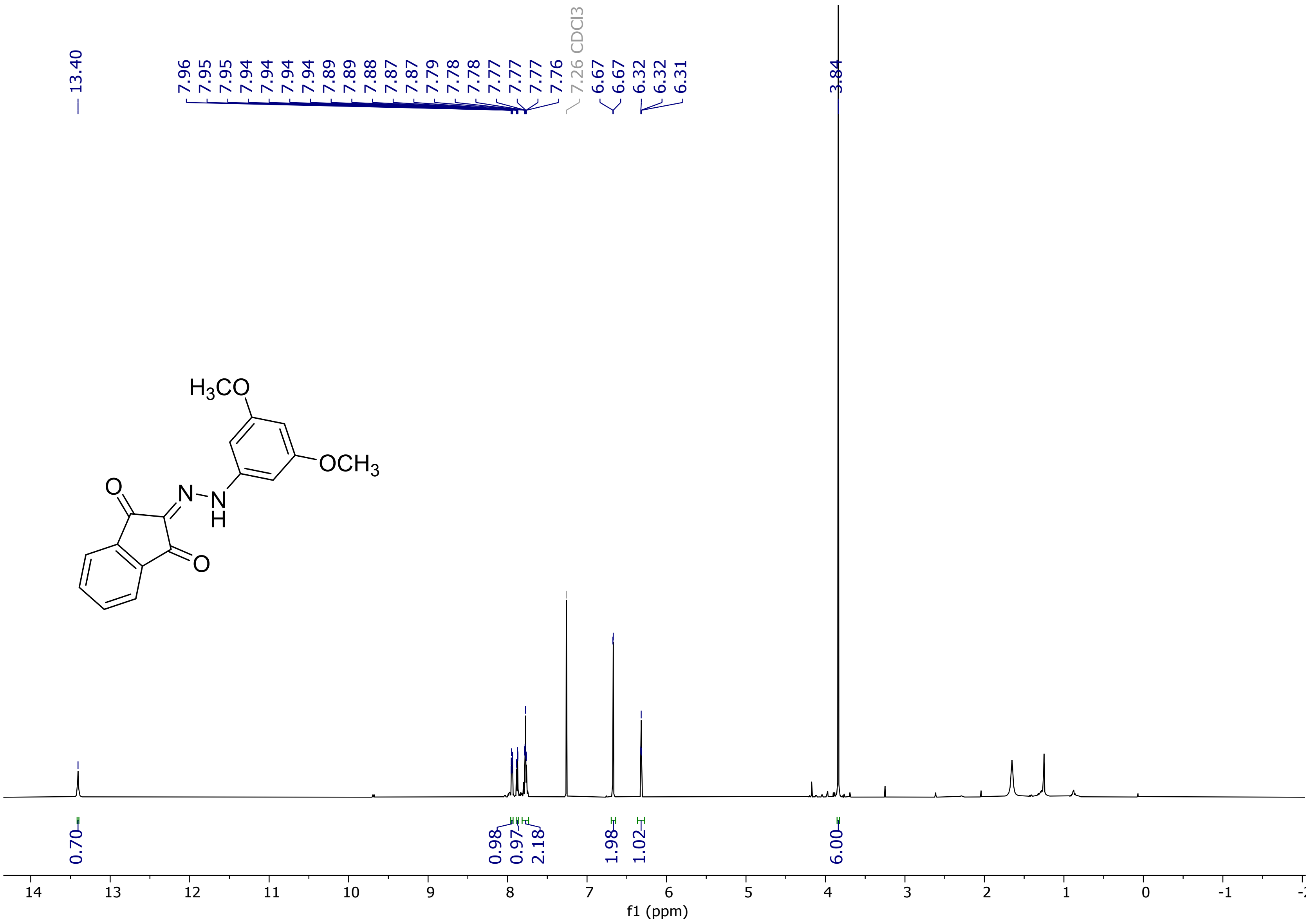

Figure S71. ^{1}H NMR spectrum of **7m** (500 MHz, $CDCl_3$)

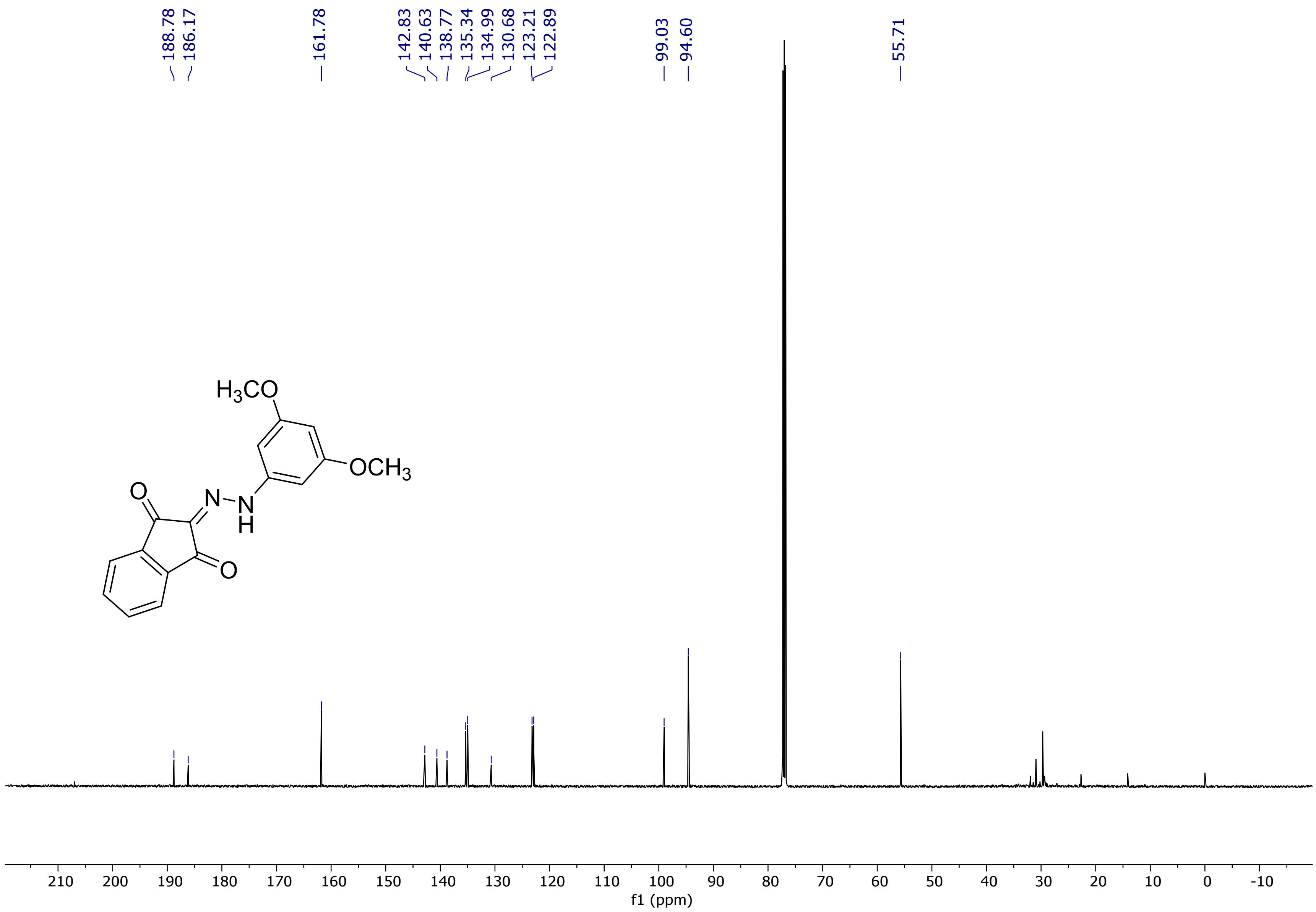

Figure S72. ^{13}C NMR spectrum of **7m** (125 MHz, $CDCl_3$)

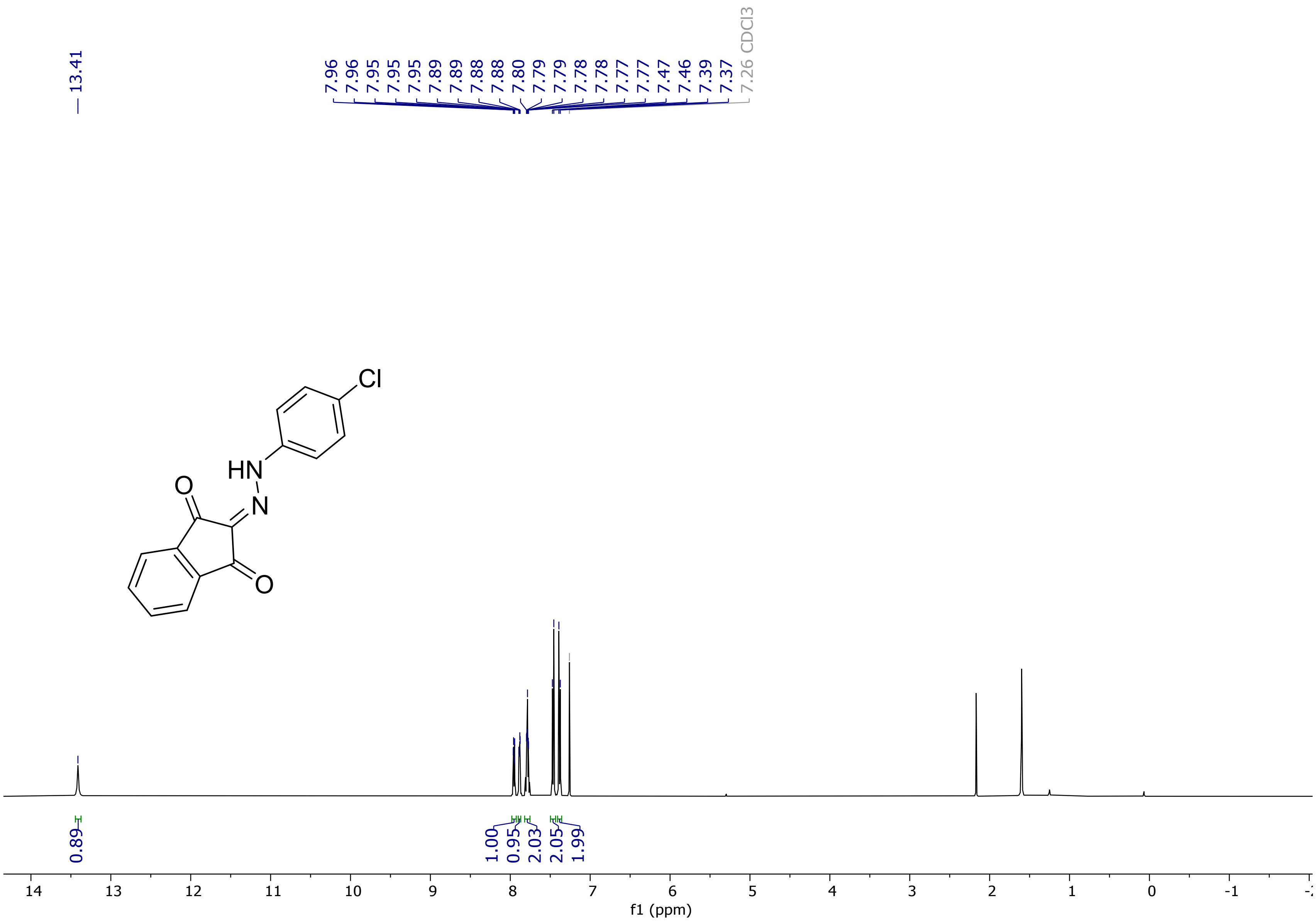

Figure S73. ^{1}H NMR spectrum of **7n** (500 MHz, $CDCl_3$)

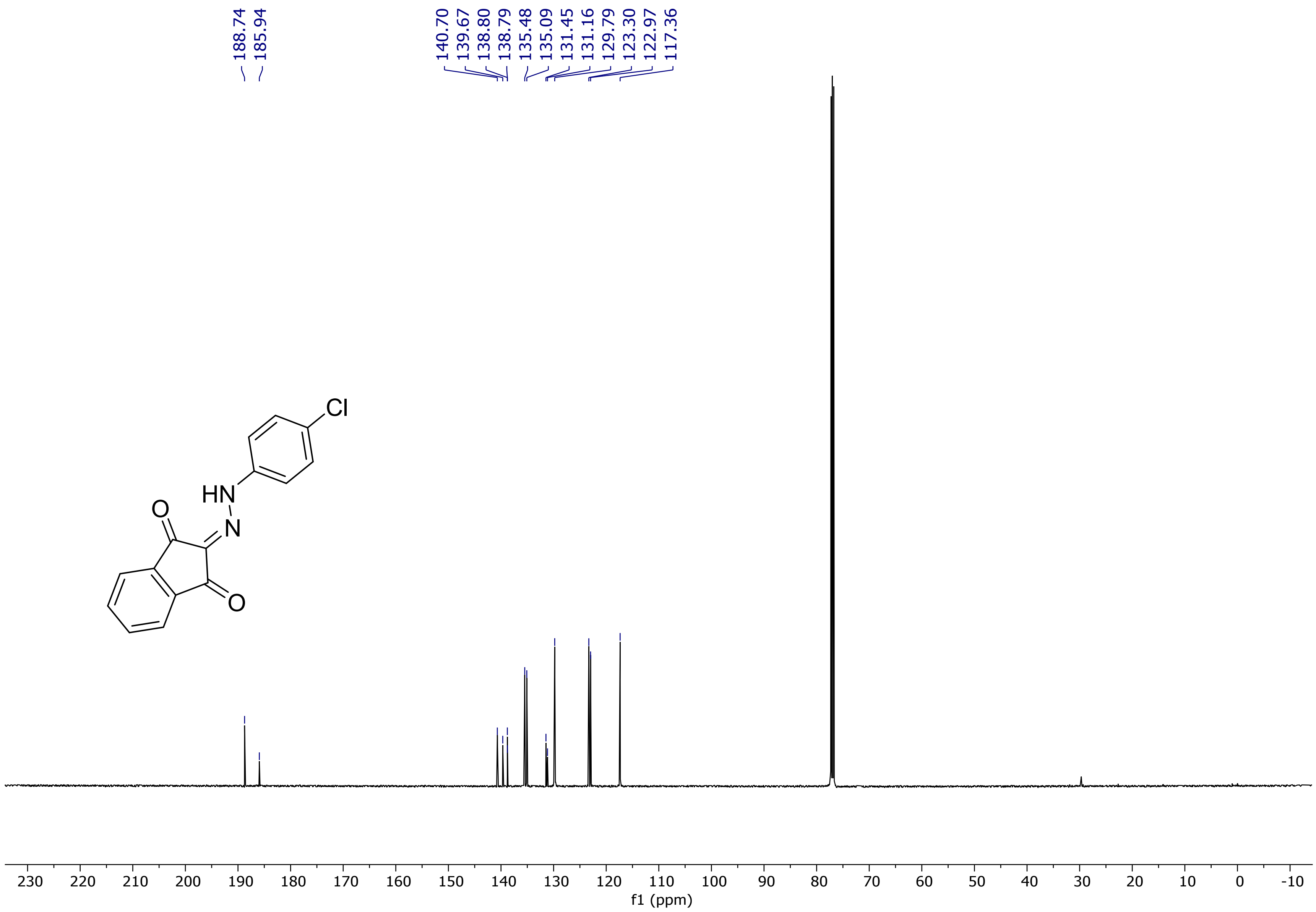

Figure S74. ^{13}C NMR spectrum of **7n** (125 MHz, $CDCl_3$)

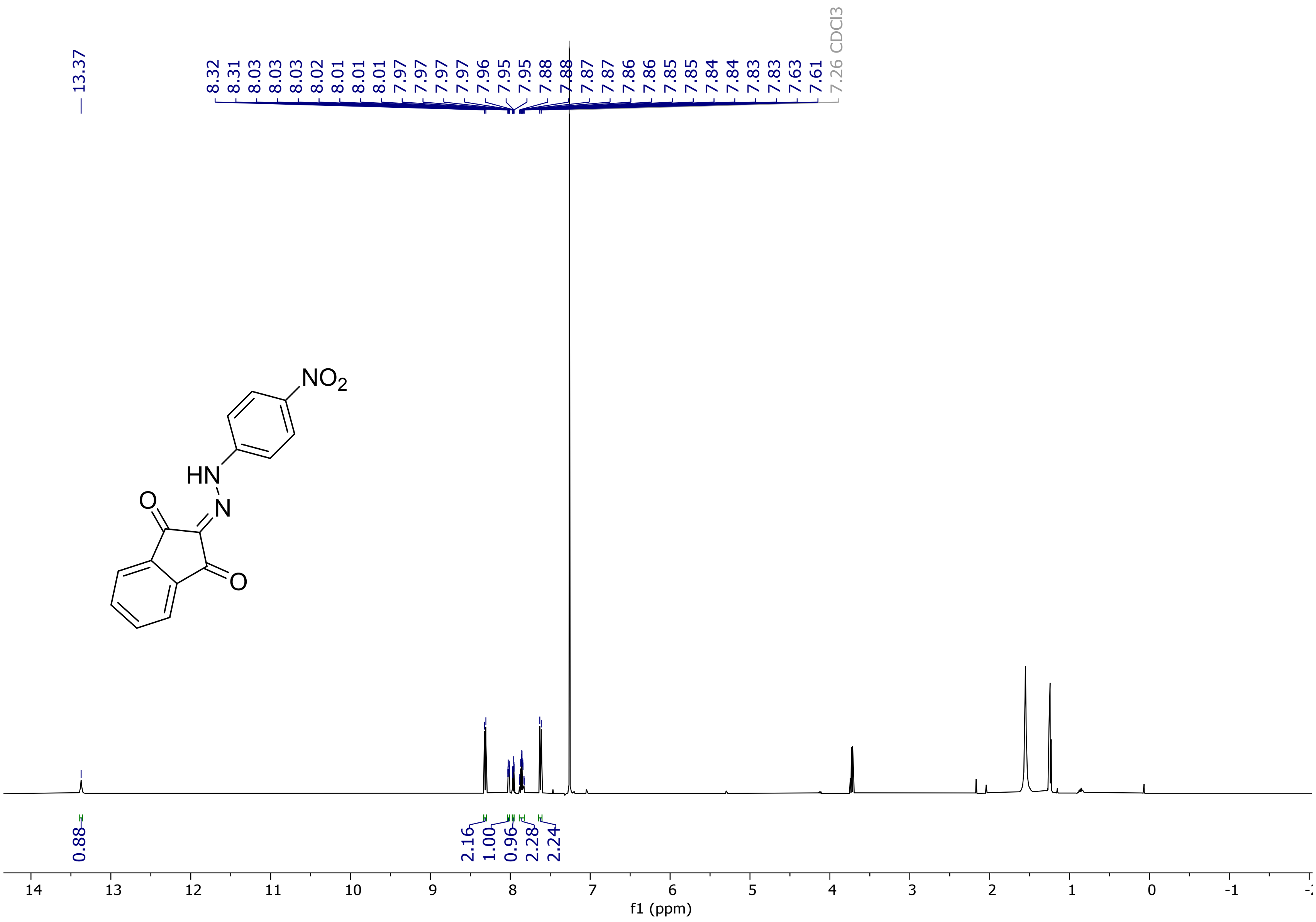

Figure S75. ^{1}H NMR spectrum of **7o** (500 MHz, $CDCl_3$)

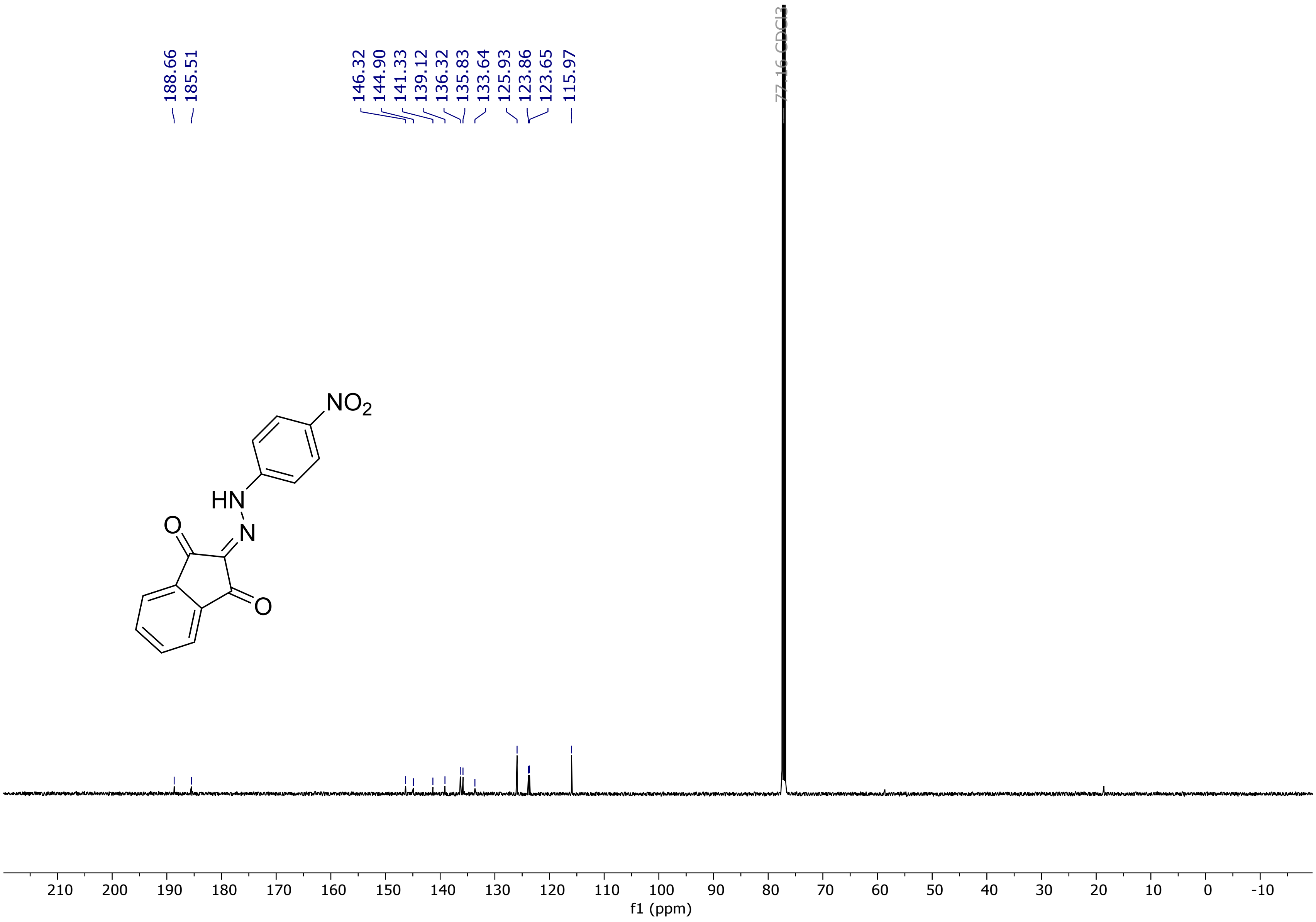

Figure S76. ^{13}C NMR spectrum of **7o** (125 MHz, $CDCl_3$)

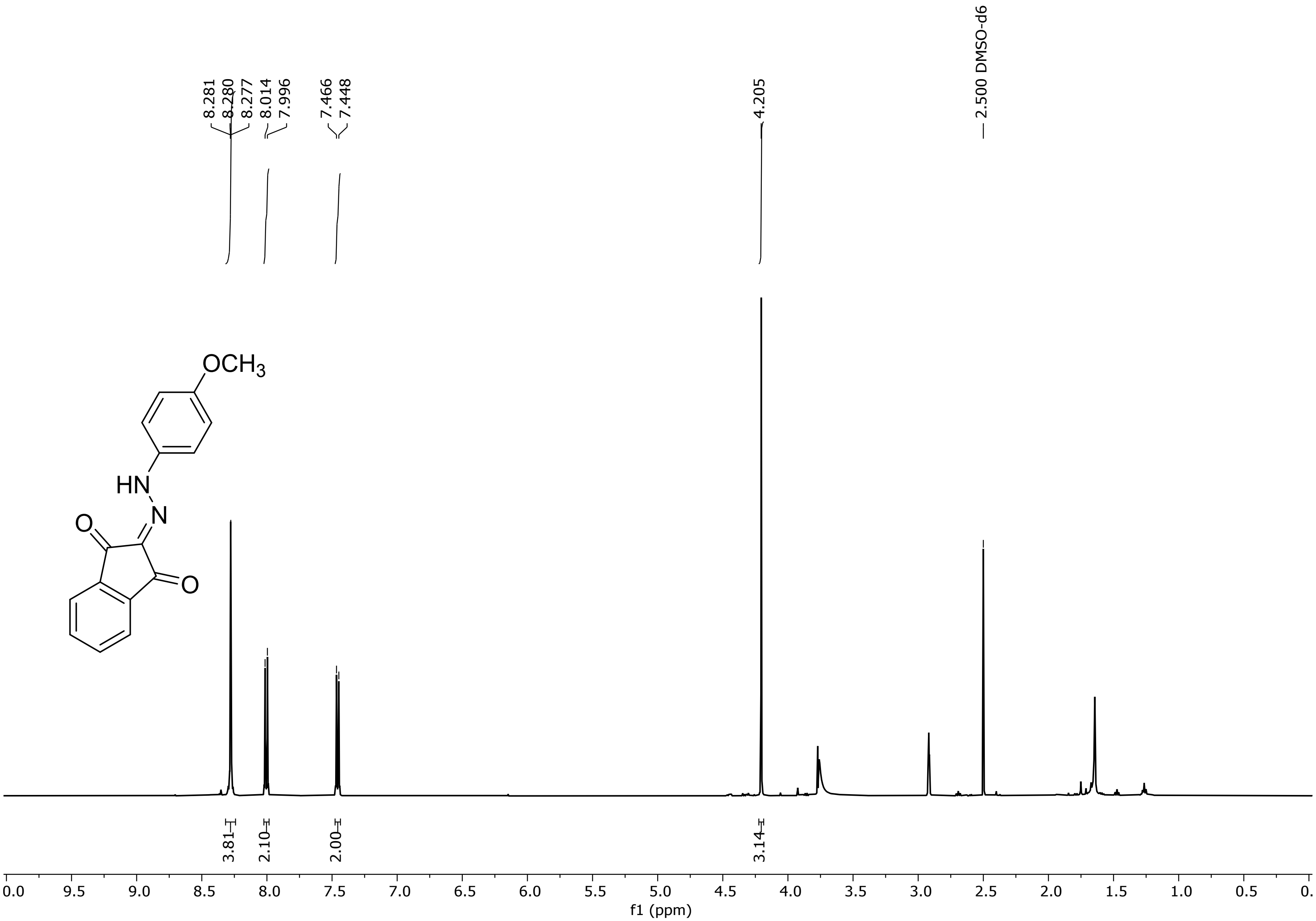

Figure S77. ^{1}H NMR spectrum of **7p** (500 MHz, DMSO-d6)

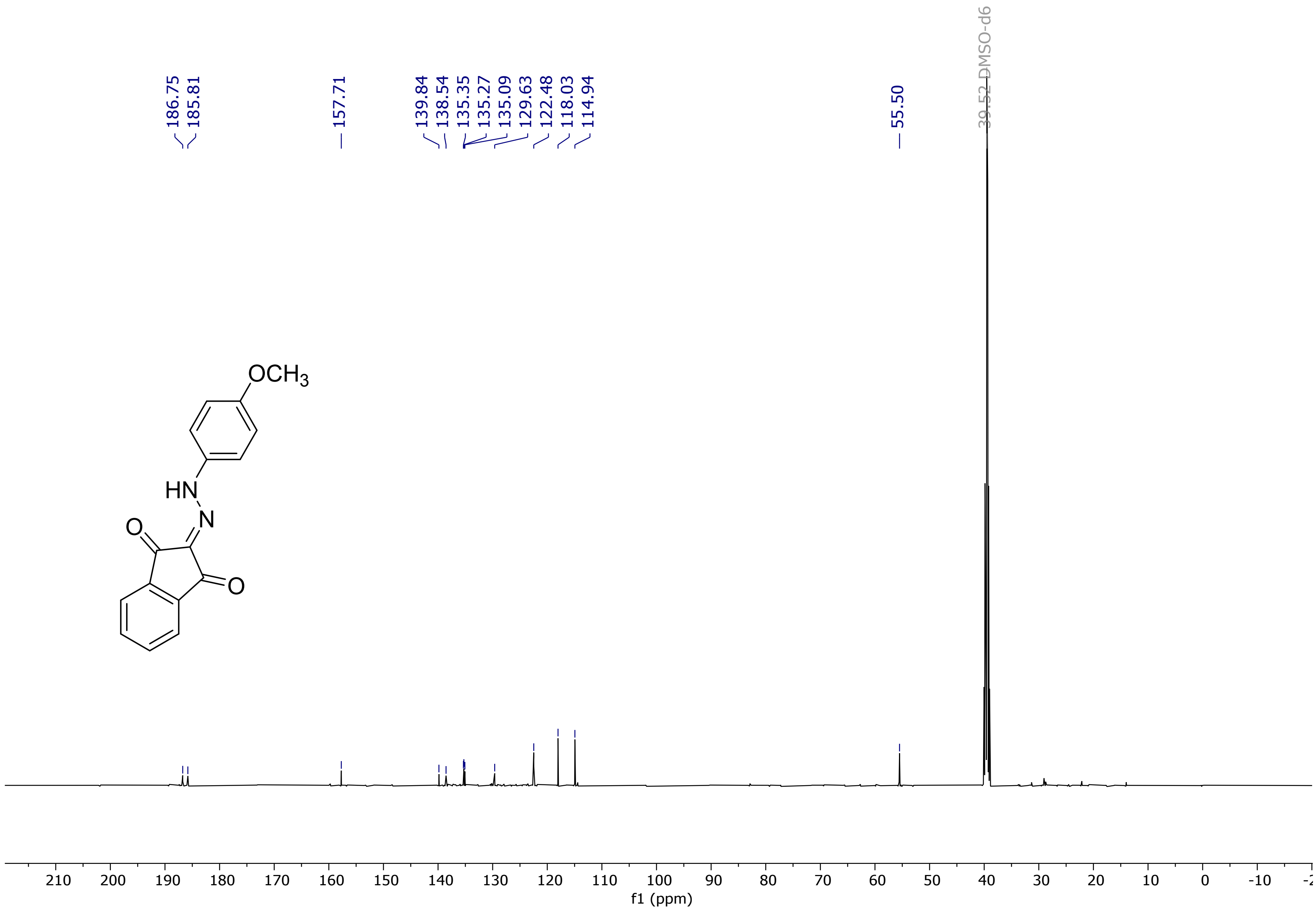

Figure S78. ^{13}C NMR spectrum of **7p** (125 MHz, DMSO-d6)

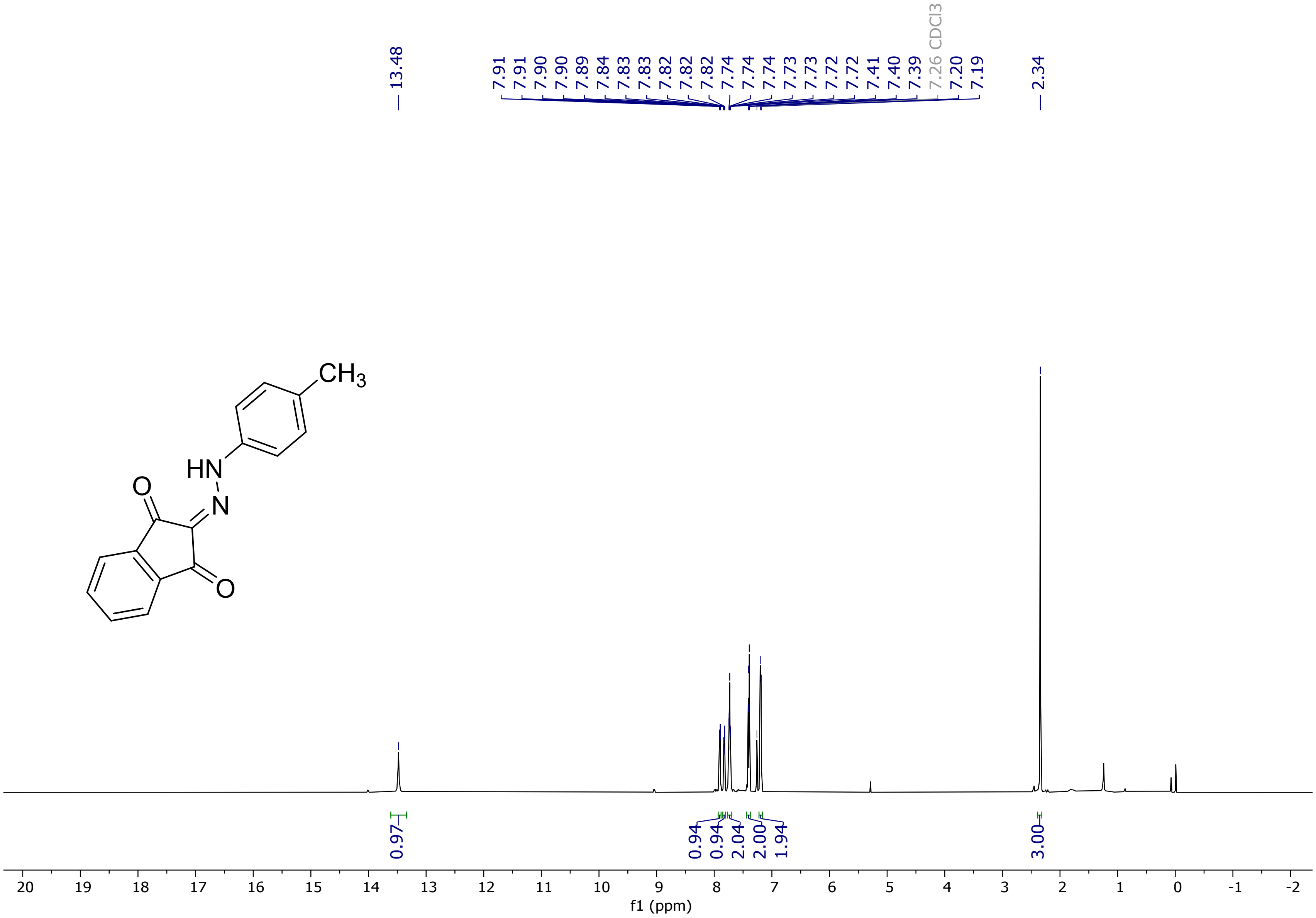

Figure S79. ^{1}H NMR spectrum of **7q** (500 MHz, $CDCl_3$)

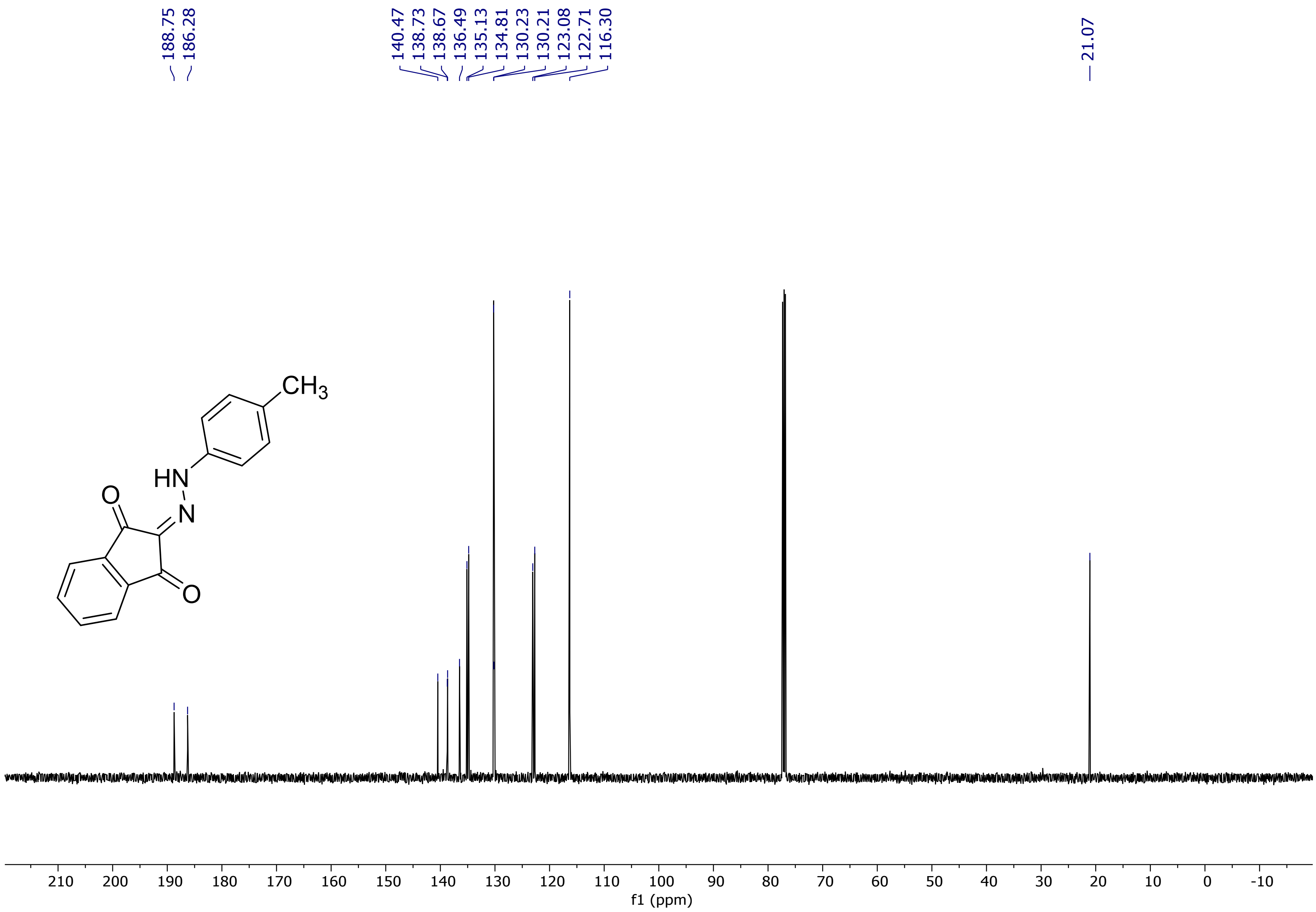

Figure S80. ^{13}C NMR spectrum of **7q** (125 MHz, D)

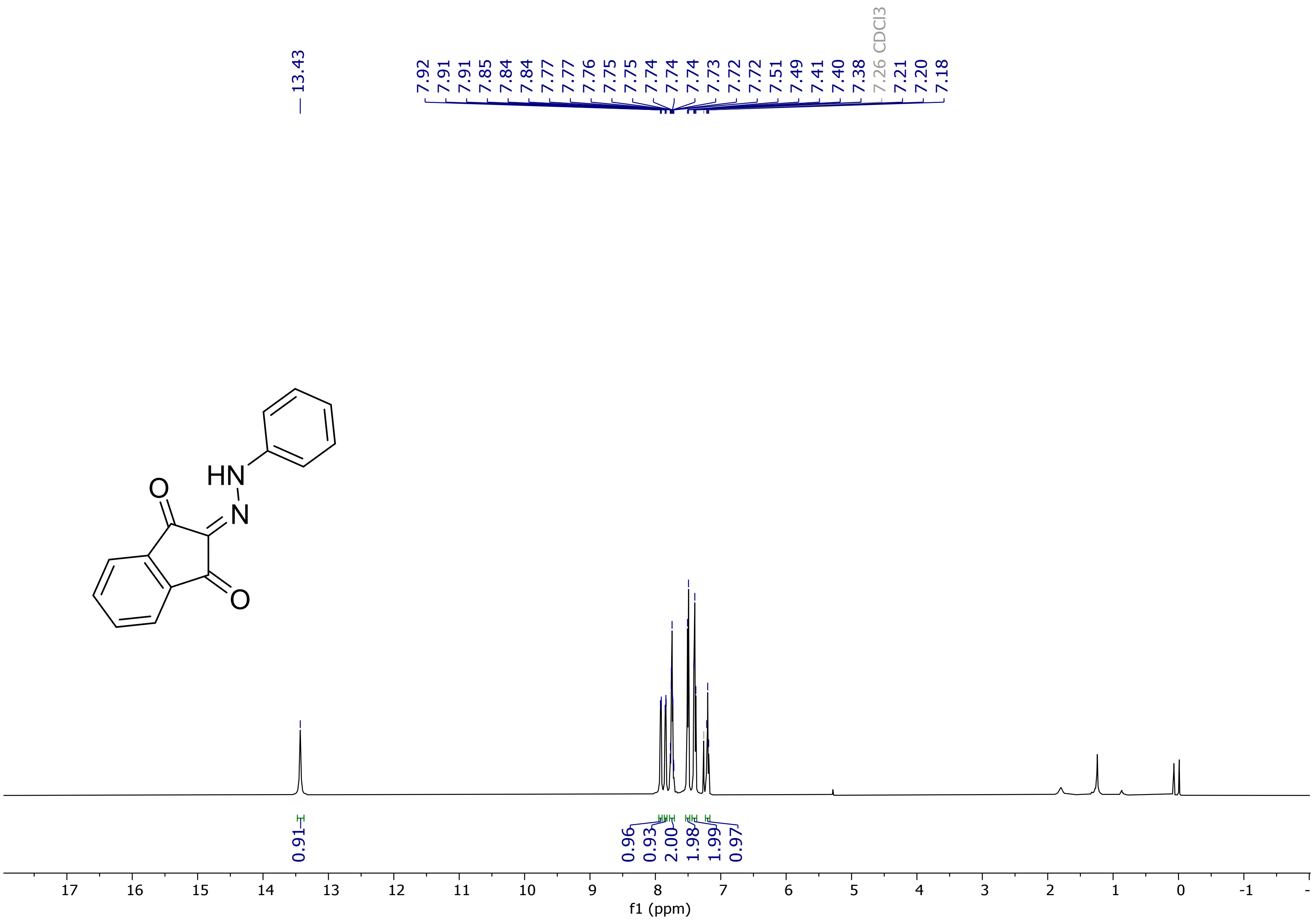

Figure S81. ^{1}H NMR spectrum of **7r** (500 MHz, $CDCl_3$)

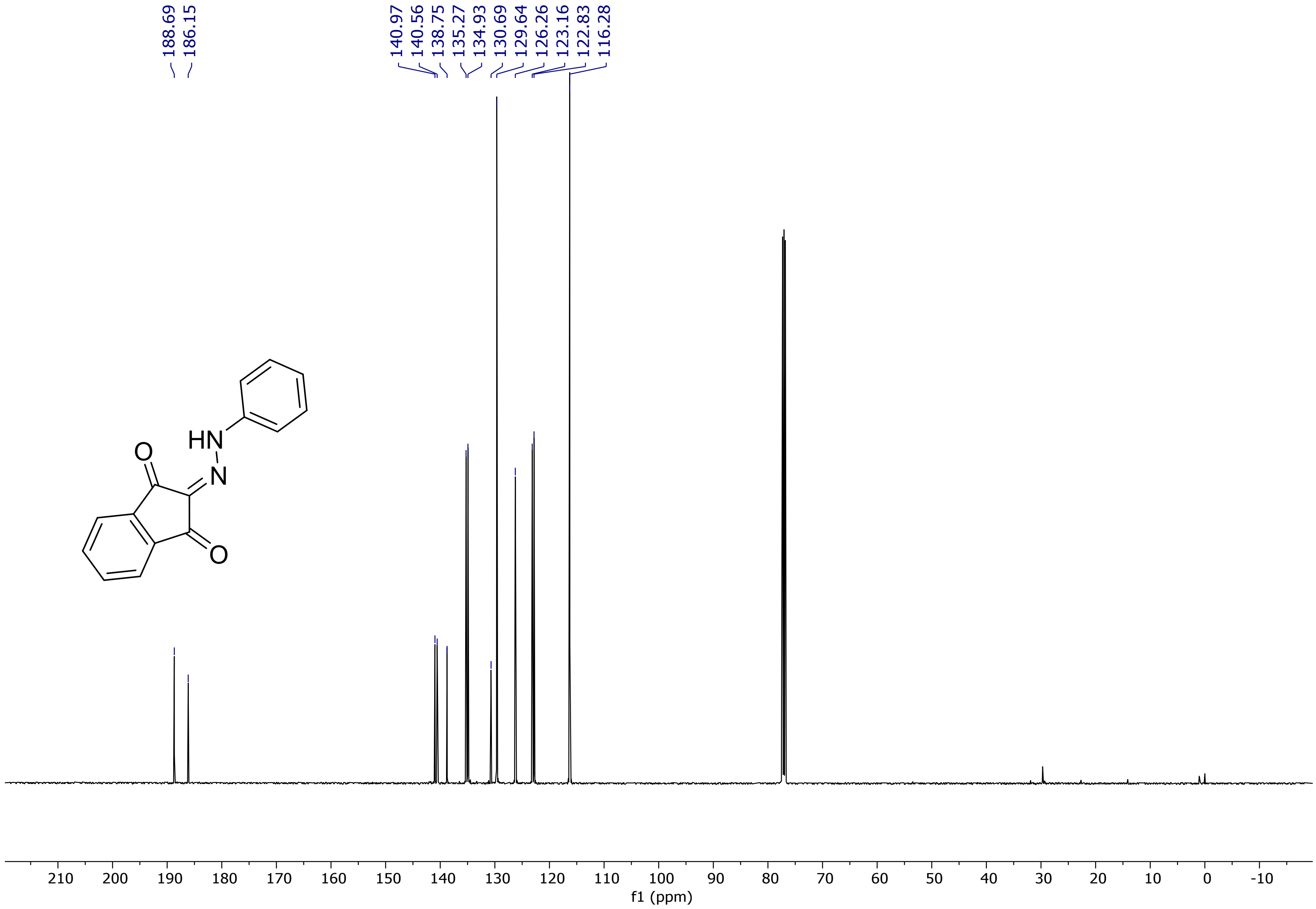

Figure S82. ^{13}C NMR spectrum of **7r** (125 MHz, $CDCl_3$)

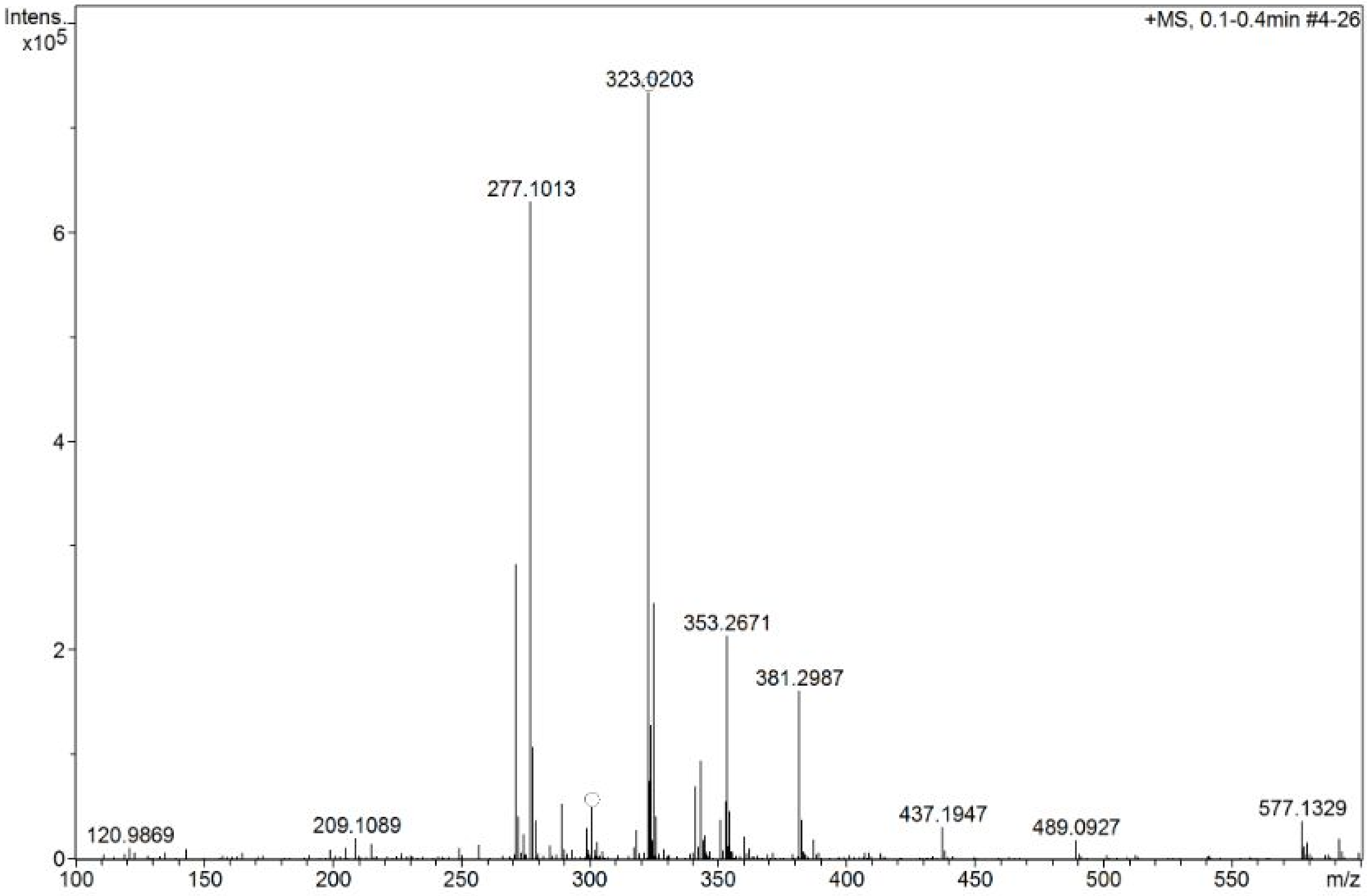

Figure S83. HRMS spectrum of **3d**

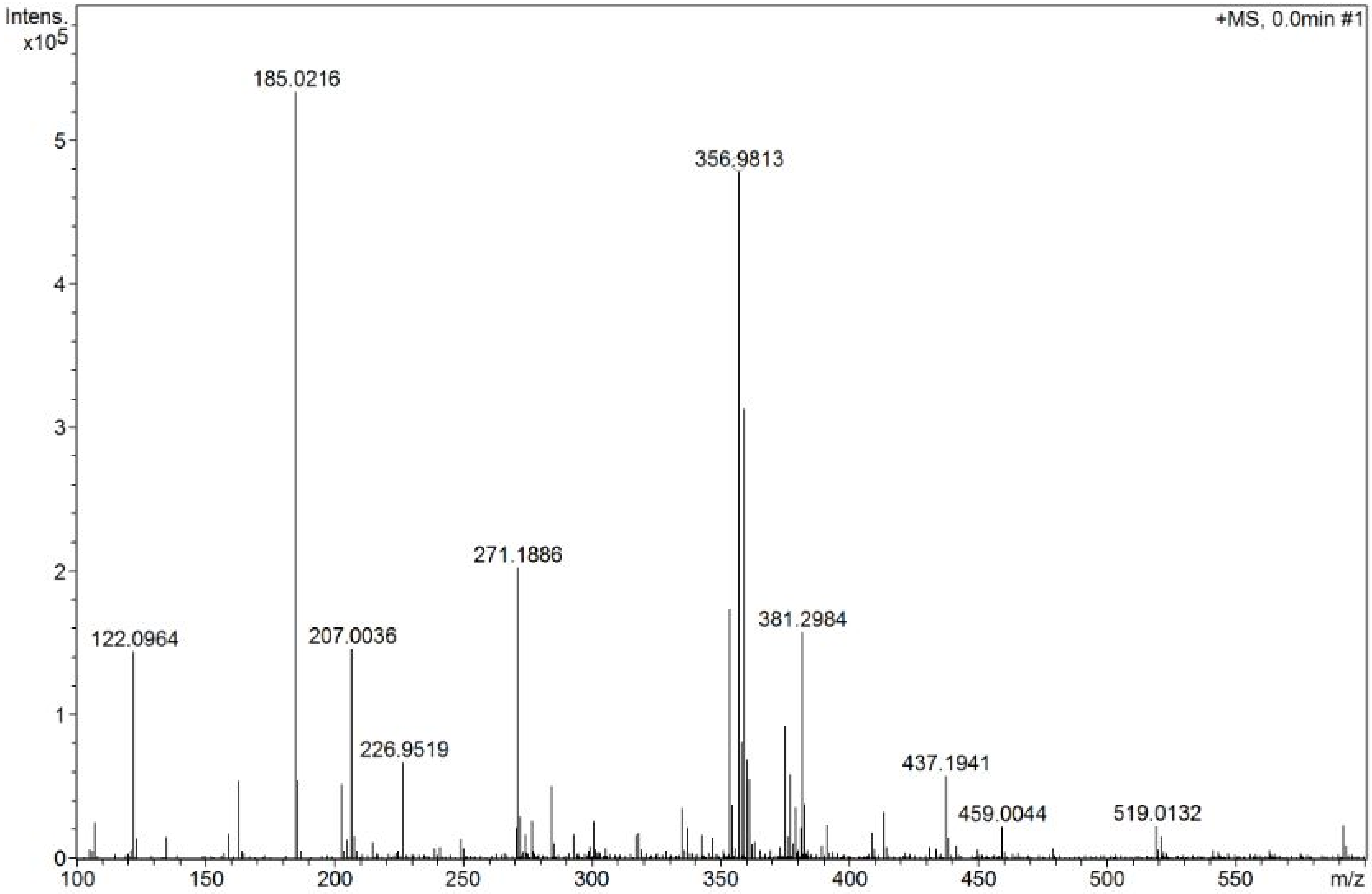

Figure S84. HRMS spectrum of **3g**

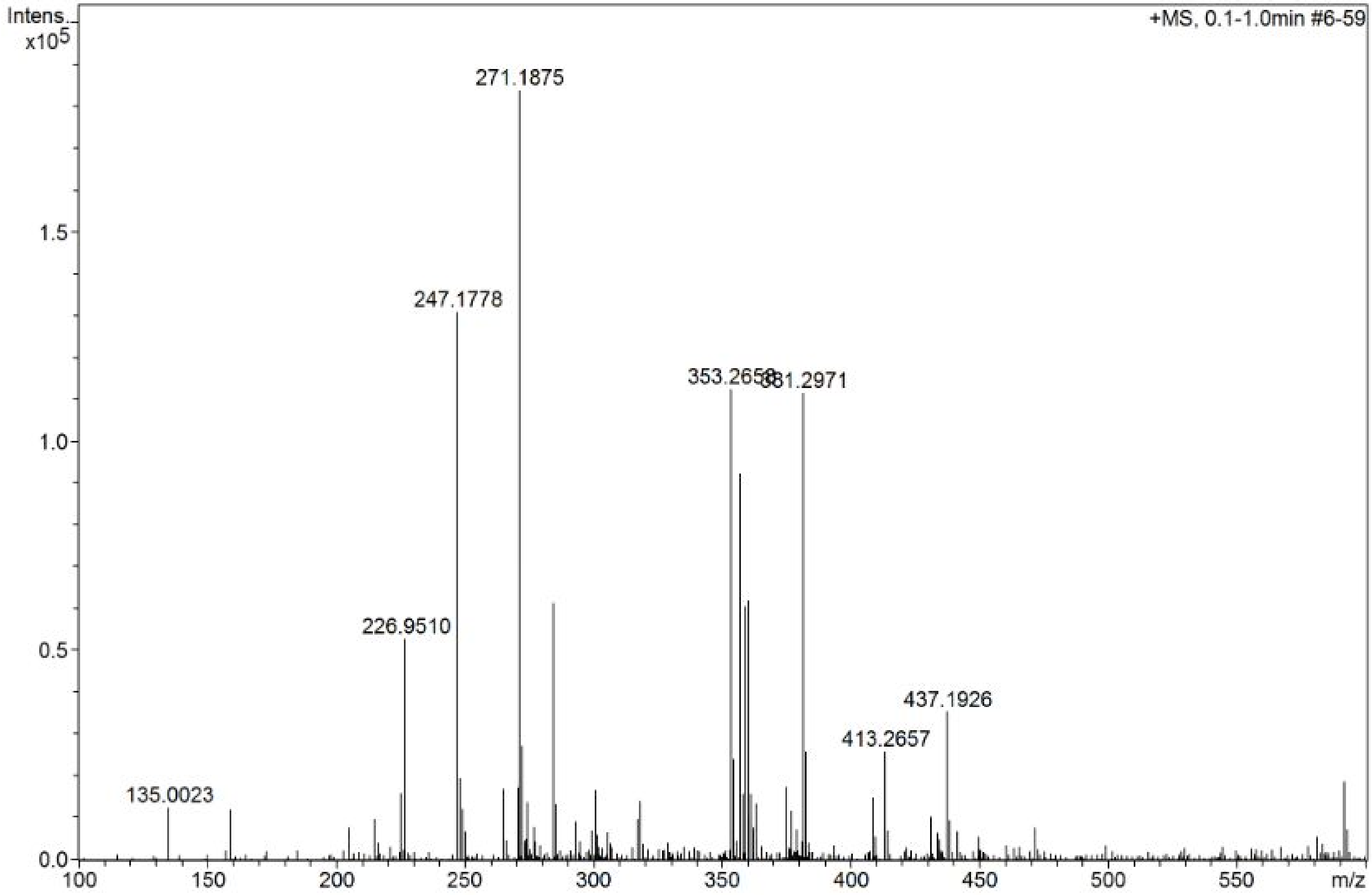

Figure S85. HRMS spectrum of **3h**

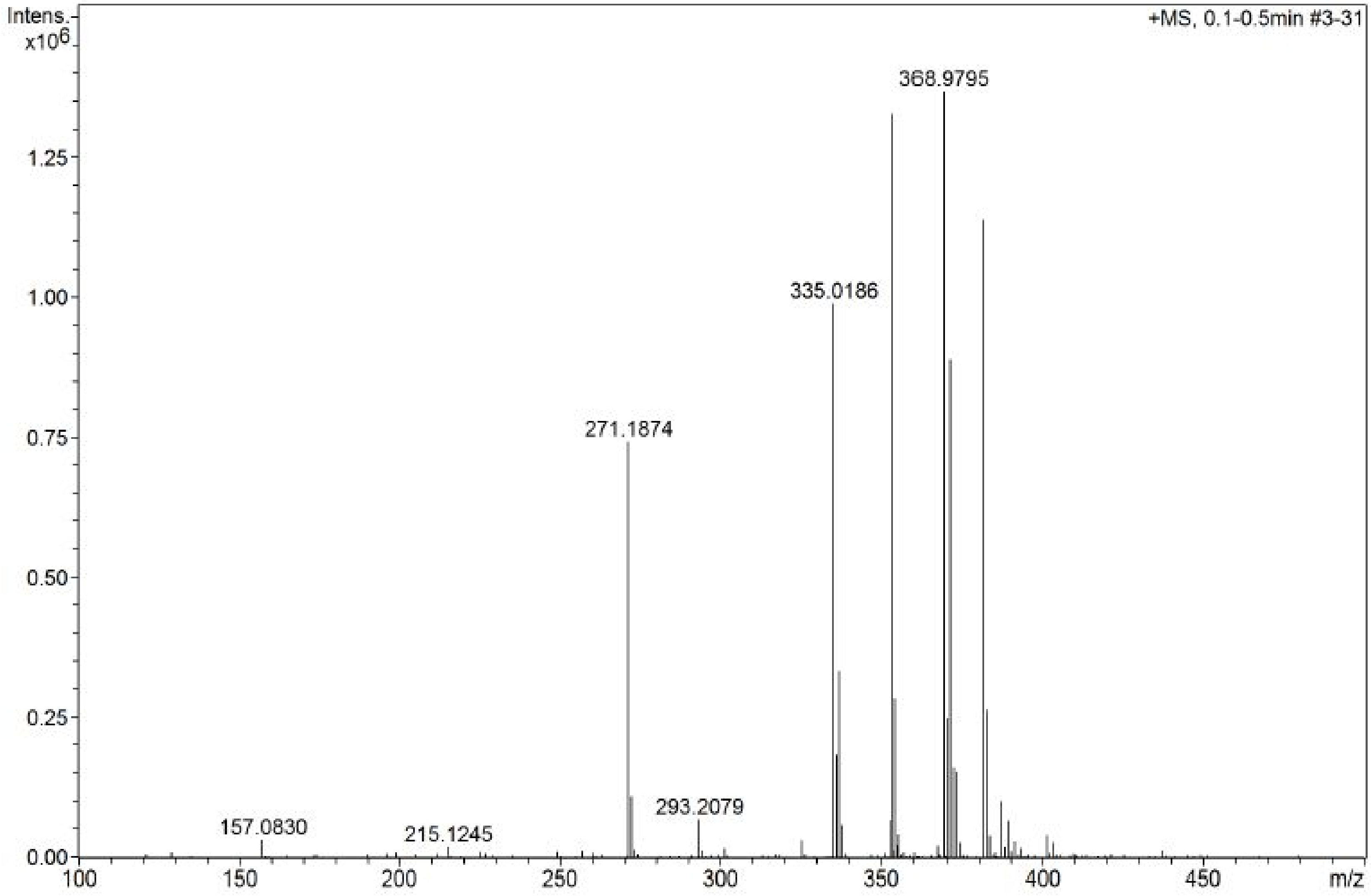

Figure S86. HRMS spectrum of **3p**

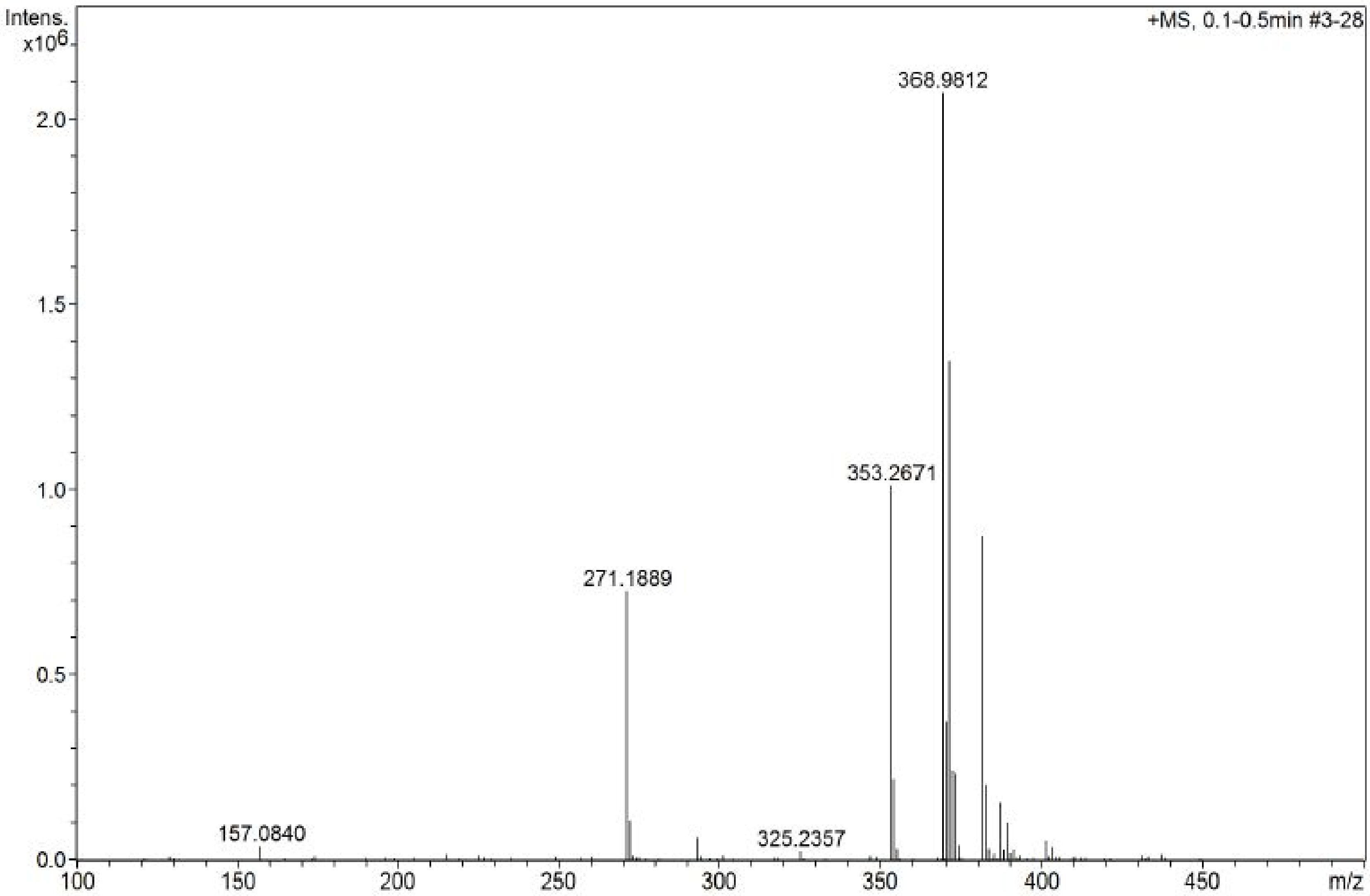

Figure S87. HRMS spectrum of **3q**

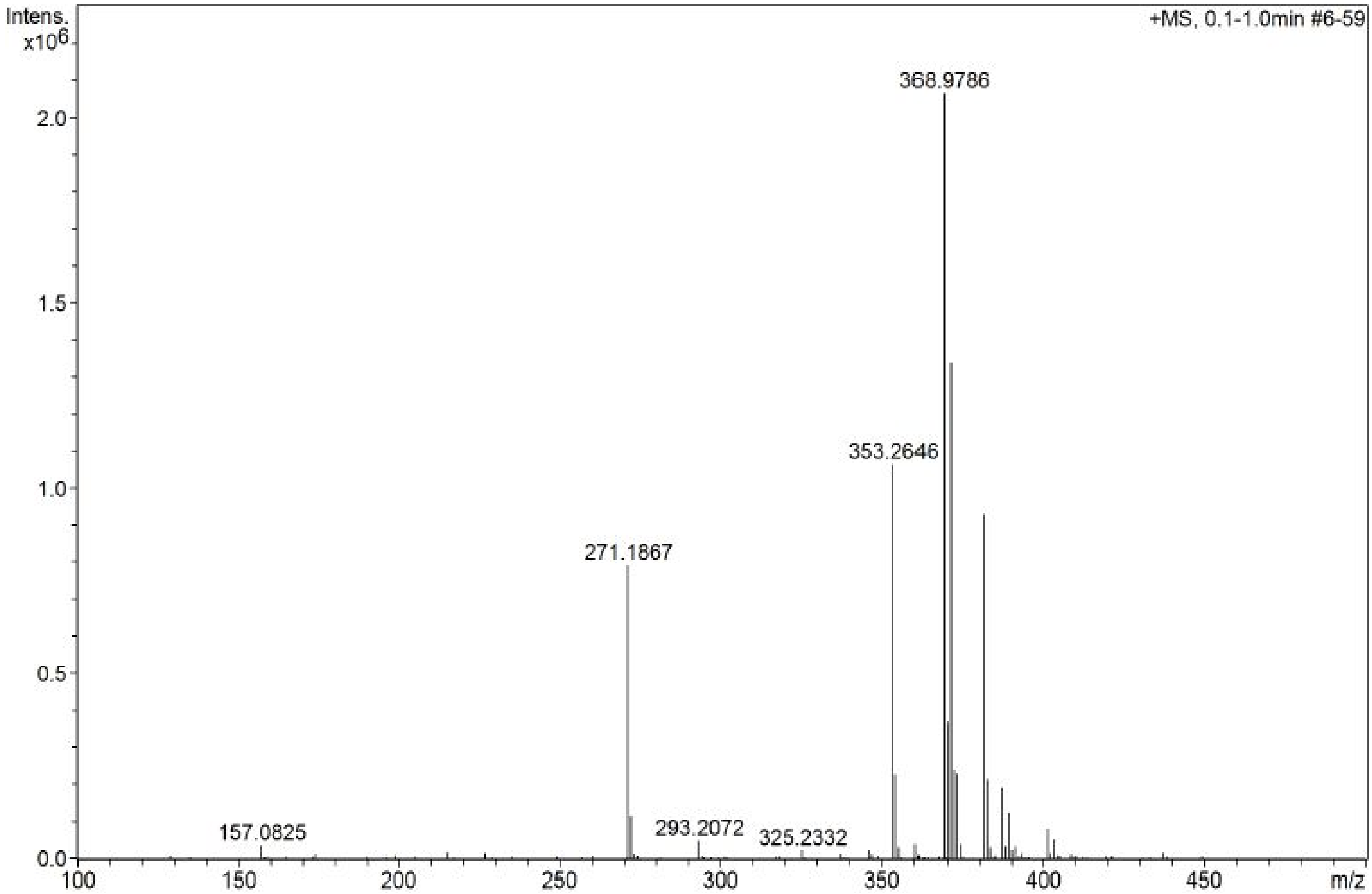

Figure S88. HMRS spectrum of **3r**

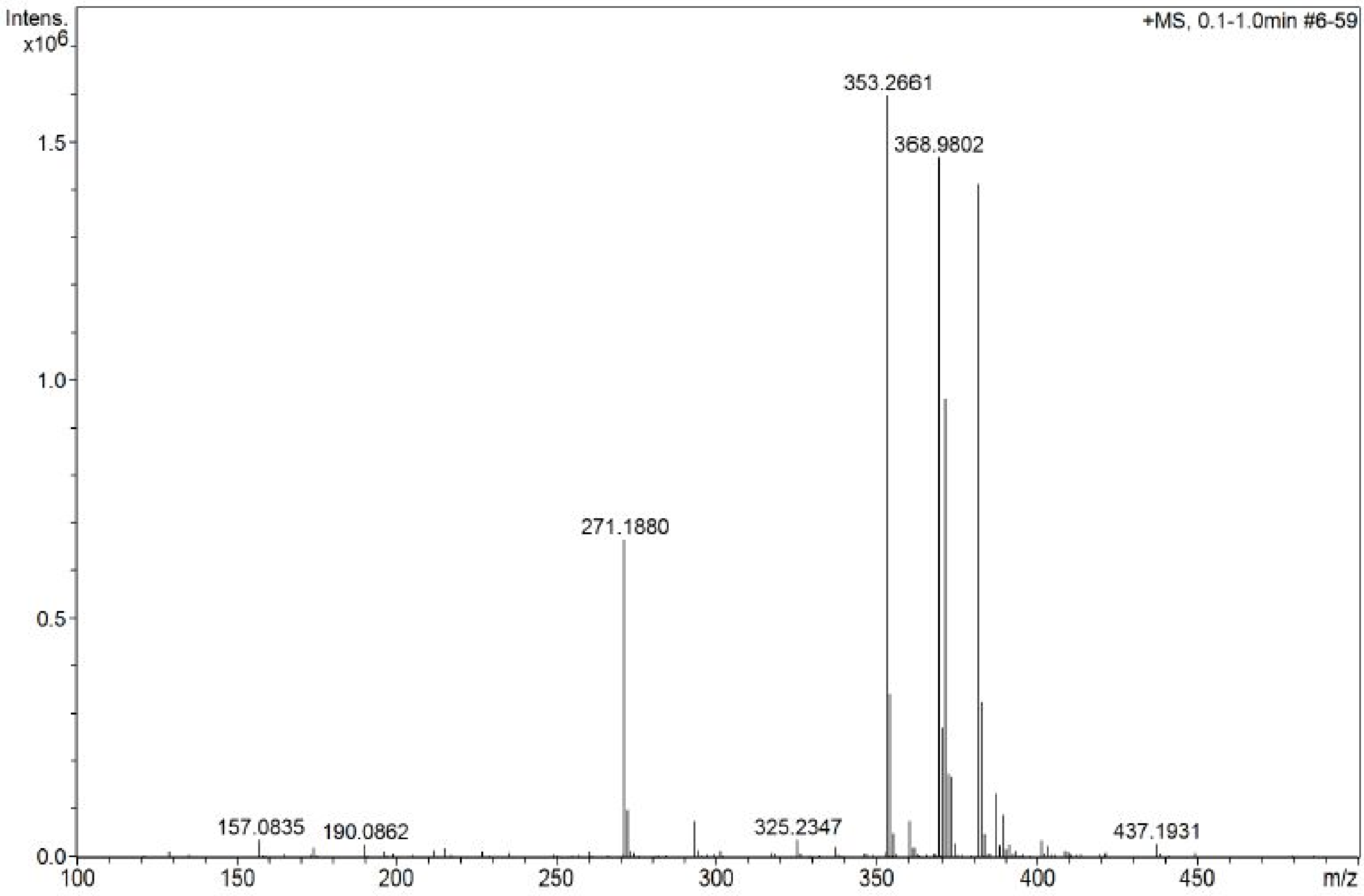

Figure S89. HRMS spectrum of **3s**

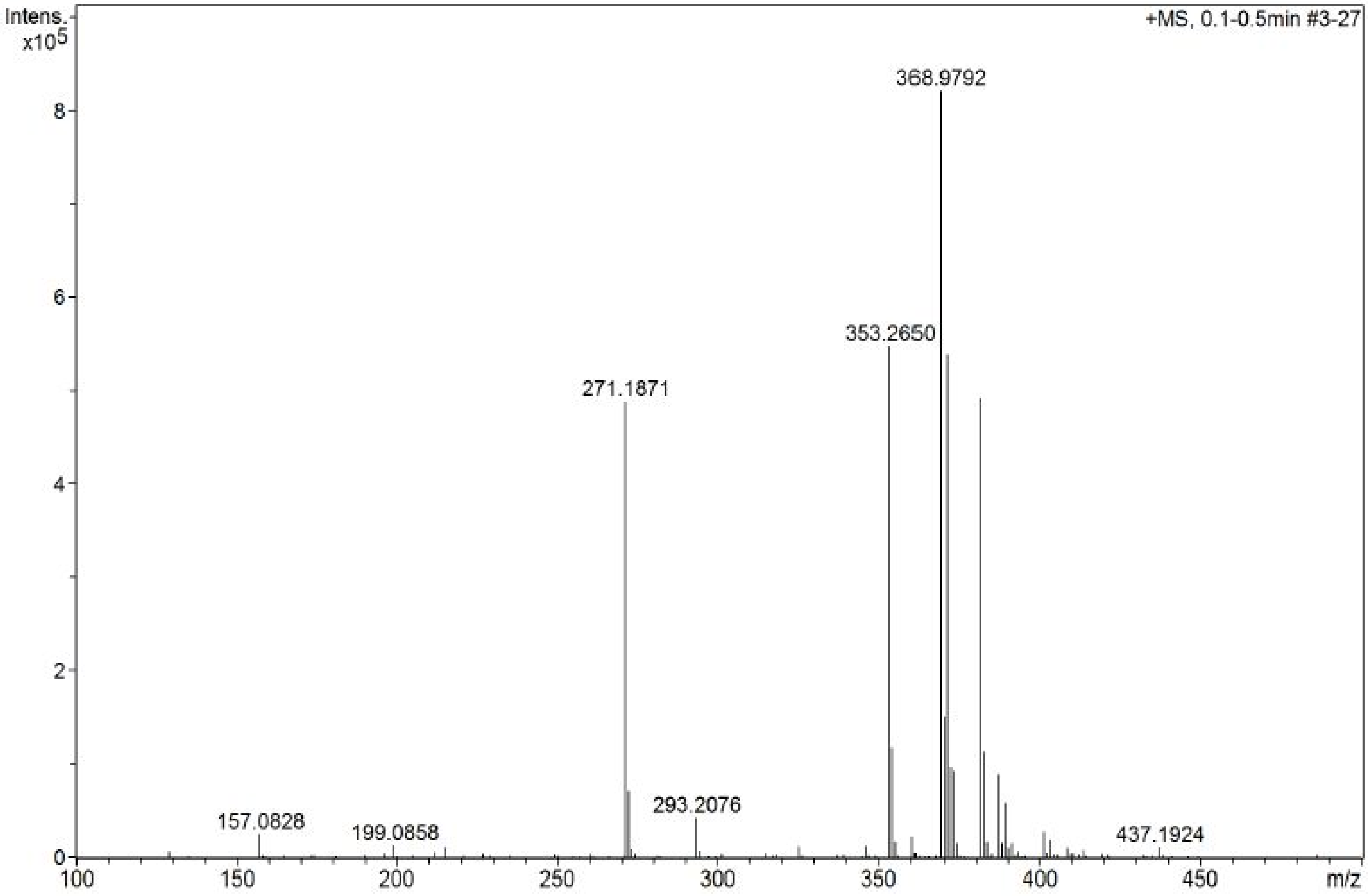

Figure S90. HRMS spectrum of **3t**

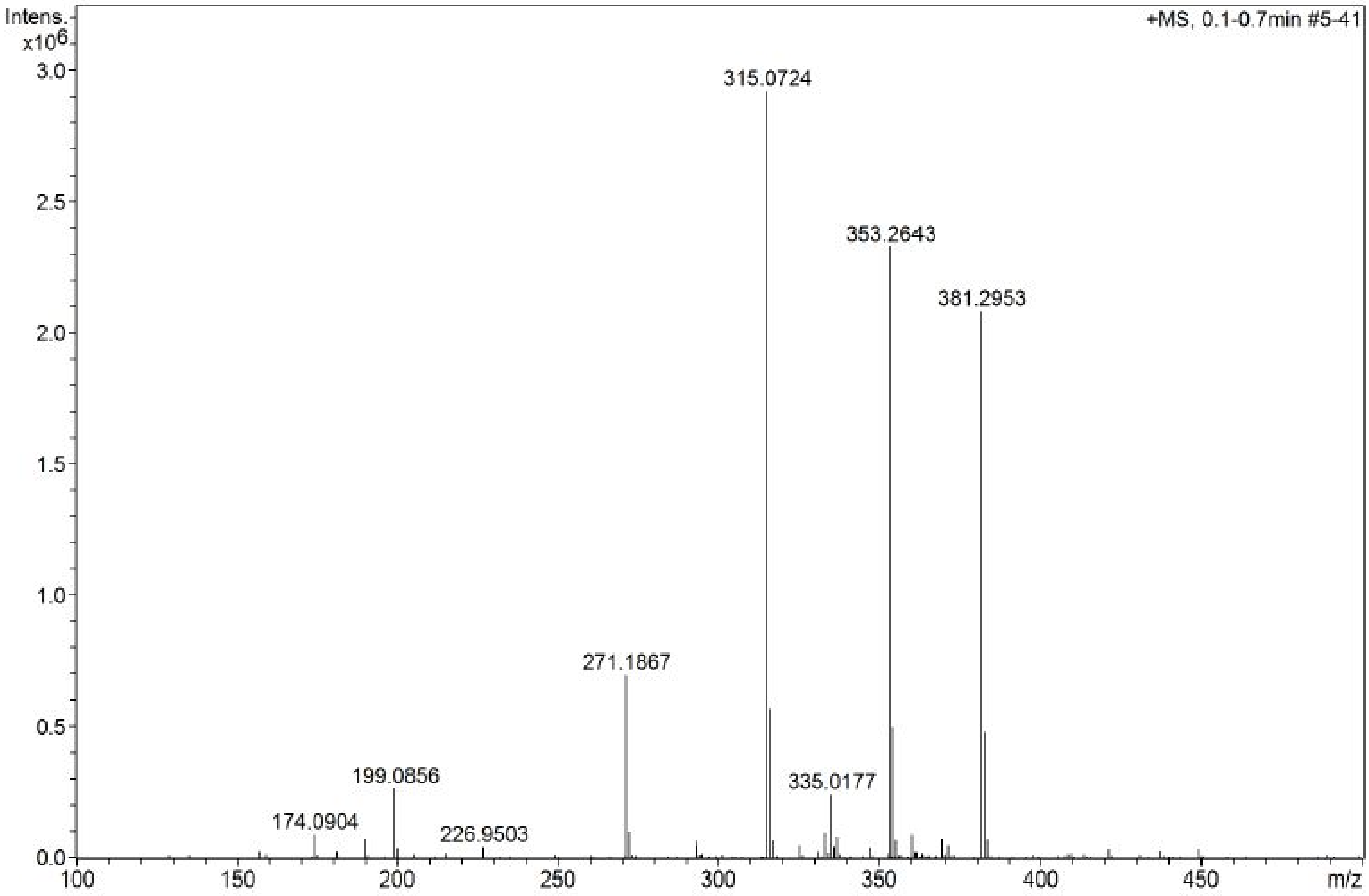

Figure S91. HRMS spectrum of **3u**

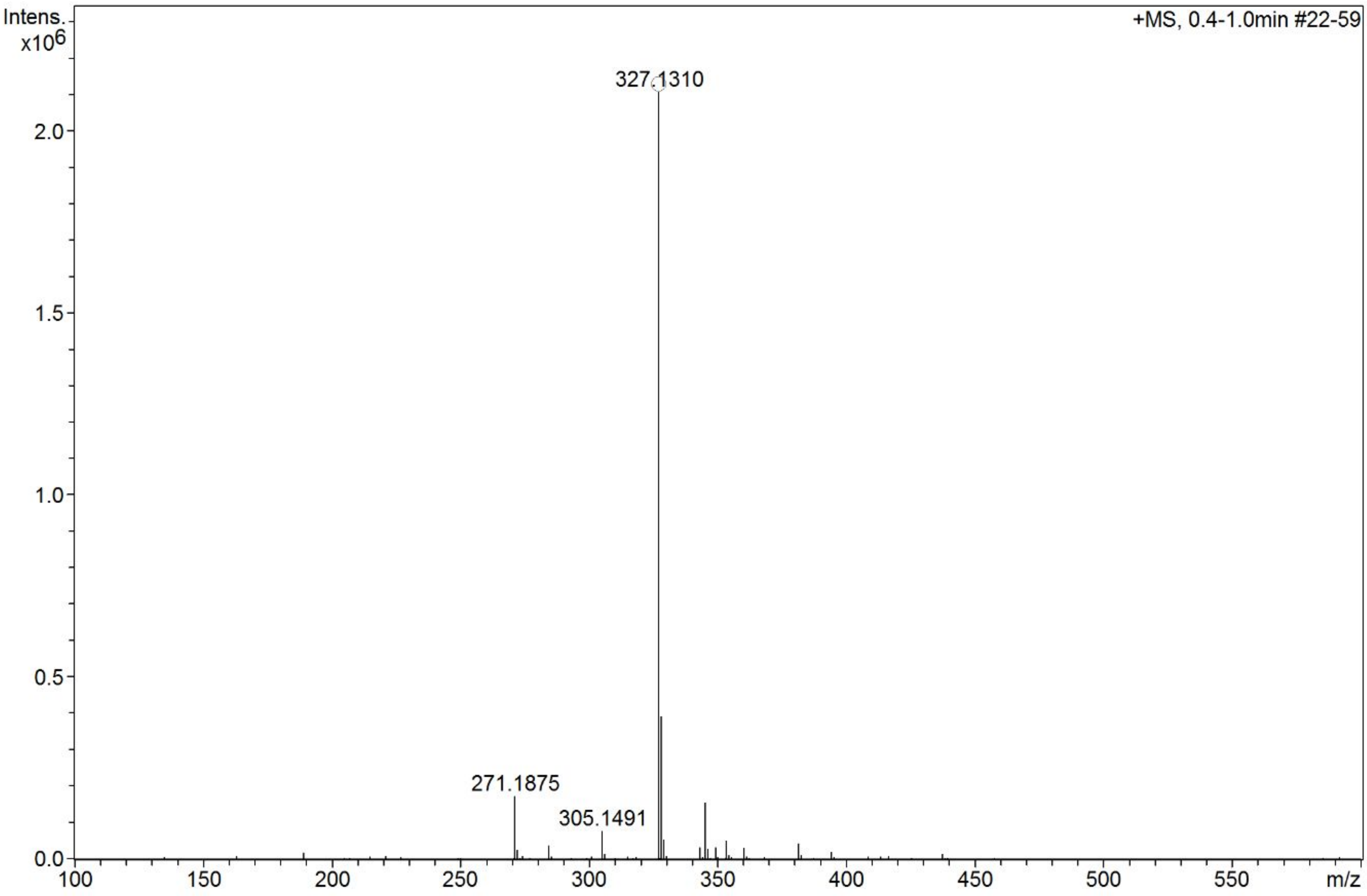

Figure S92. HRMS spectrum of **7a**

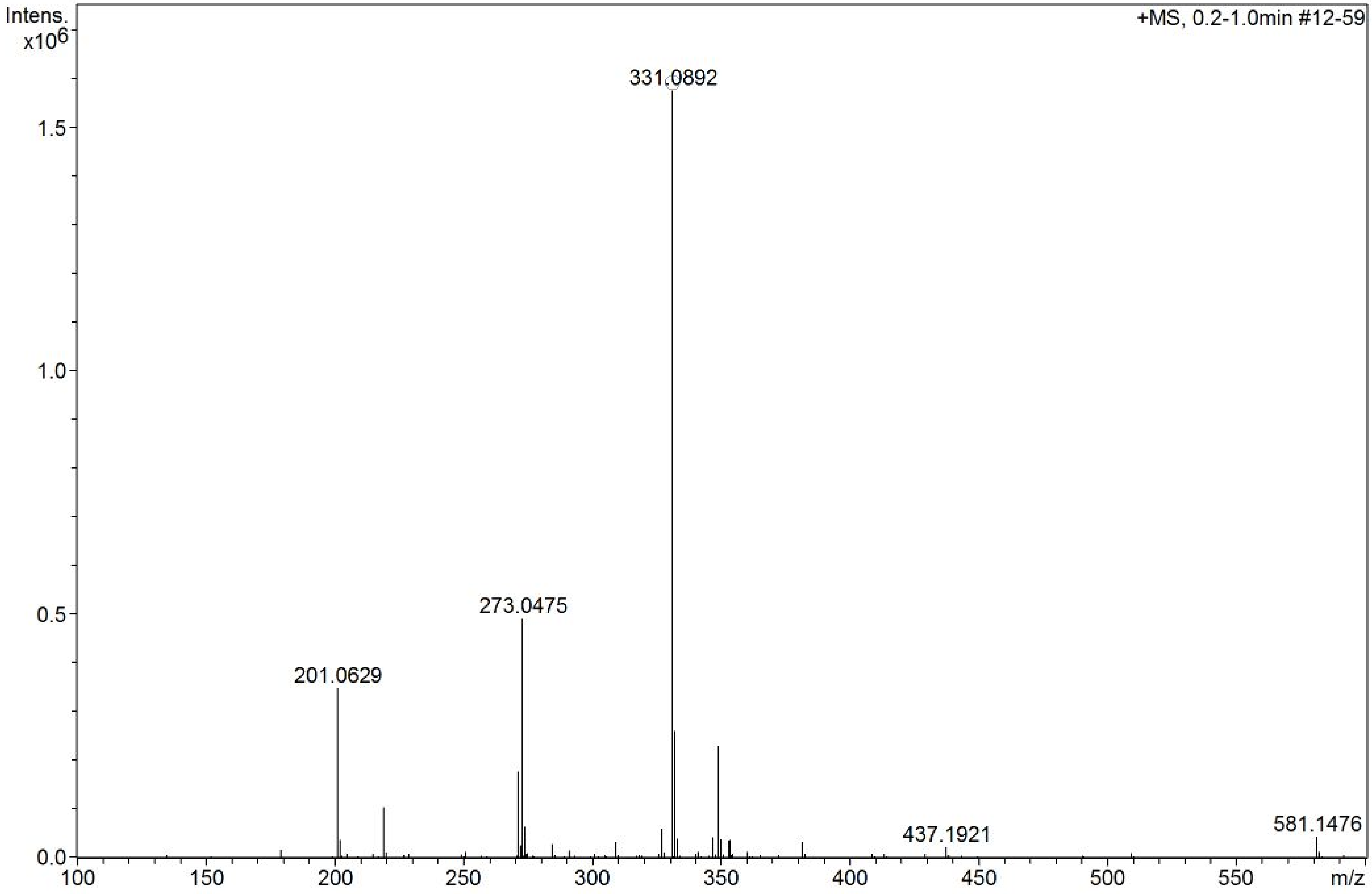

Figure S93. HRMS spectrum of **7g**

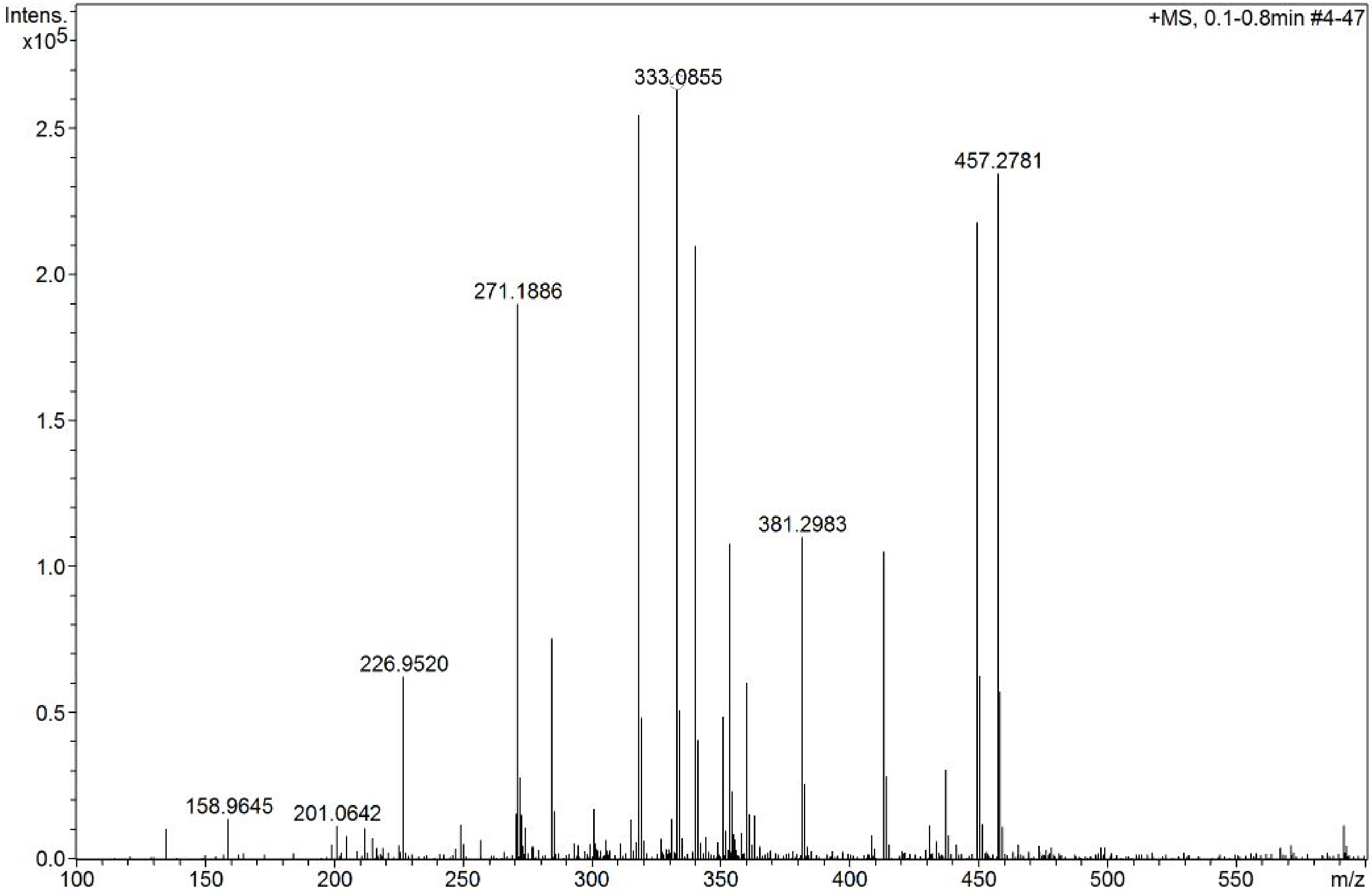

Figure S94. HRMS spectrum of **7m**

S5. Computational details

All calculations were performed at the Gaussian 09 software.[1] Geometries were fully optimized using the density functionals ωB97X-D[2] and M06-2X[3], combined with the def2-TZVP basis set.[4] We chose the hybrid M06-2X and the range-separated ωB97X-D functionals based on their established ability to accurately describe non-bonded and dispersive interactions, such as hydrogen bonds and con-jugated systems,[2,3] which are relevant to the systems under investigation. Furthermore, these functionals have demonstrated superior performance in benchmarking studies for characterizing thermodynamic and kinetic properties.[5] Additionally, they have been previously employed in DFT evaluations of tautomerism and conformational analyses.[6,7] The calculations were conducted in both gas phase and solvent media (water and ethanol), with the solvent effects simulated using the implicit SMD[8] solvation model based on the molecular electron density. The minimum energy points were characterized by computing their second-order Hessian matrix, showing only positive eigenvalues. Thermodynamic parameters were determined at standard conditions (298 K and 1 atm) using the default statistical mechanics equations provided by the Gaussian software after frequency calculations**.**

S5.1. Tautomerism analysis

Table S1 presents the calculated values for both ωB97X-D and M06-2X relative to the most stable form, *E*-NH, showing the same range of energy. Notably, due to the significant energy difference in both -OH forms (ΔG > 6 kcal mol^{-1}), this tautomer is sparsely populated. The M06-2X functional showed a reduction in the magnitude of the energy difference between the isomers compared to ωB97X-D. This reduction could be attributed to M06-2X's ability to accurately describe dispersive effects (high HF percentage), thereby contributing to the stabilization of the intrinsic structure through intramolecular interactions.

Table S1. Relative energies (ΔG, in kcal mol^{-1}) of the evaluated systems obtained at ωB97X-D/ and M06-2X/def2-TZVP@SMD level, calculated relative to the most stable form (*E*-NH).

		Z-NH	A-OH	B-OH	C-OH	D-OH
ωB97X-D	gas phase	1.3	6.6	14.4	20.7	
	SMD = water	1.2	7.7	11.8	16.0	[*a*]
	SMD = ethanol	1.1	7.9	12.8	22.0	
M06-2X	gas phase	0.8	4.8	10.4	18.1	9.4
	SMD = water	1.4	6.0	9.9	16.9	10.3

SMD = ethanol	1.9	5.7	10.8	17.0	10.1

[*a*] proton migration along the optimization: converges to the *Z*-NH form.

S5.2. References

1. G. M. J. Frisch, W. Trucks, H. B. Schlegel, G. E. Scuseria, M. A. Robb, J. R. Cheeseman, G. Scalmani, V. Barone, B. Mennucci, G. A. Petersson, H. Nakatsuji, M. Caricato, X. Li, H. P. Hratchian, A. F. Izmaylov, J. Bloino, G. Zheng, J. L. Sonnenberg, M. J. Frisch, G. W. Trucks, H. B. Schlegel, G. E. Scuseria, M. A. Robb, G. Cheeseman, J. R. Scalmani, V. Barone, B. Mennucci, H. Petersson, G. A. Nakatsuji, M. Caricato, X. Li, H. P. Hratchian, A. F. Izmaylov, J. Bloino, G. Zheng, J. L. Sonnenberg, K. Hada, M. Ehara, M. Toyota, R. Fukuda, J. Hasegawa, M. Ishida, T. Nakajima, Y. Honda, O. Kitao, H. Nakai, T. Vreven, J. E. Montgomery, J. A. Peralta Jr, F. Ogliaro, M. Bearpark, J. J. Heyd, E. Brothers, K. N. Kudin, R. Staroverov, V. N. Kobayashi, K. Normand, J. Raghavachari, A. Rendell, J. C. Burant, S. S. Iyengar, J. Tomasi, M. Cossi, N. Rega, J. M. Millam, M. Klene, J. E. Knox, J. B. Cross, V. Bakken, C. Adamo, J. Jaramillo, R. Gomperts, R. E. Stratmann, O. Yazyev, A. J. Austin, R. Cammi, C. Pomelli, J. W. Ochterski, R. L. Martin, K. Morokuma, V. G. Zakrzewski, G. A. Voth, P. Salvador, S. Dannenberg, J. J. Dapprich, A. D. Daniels, ¨O. Farkas, J. B. Foresman, J. V. Ortiz, J. Cioslowski and D. J. Fox, Gaussian 09, Revision E.01, Gaussian, 2009.

2. Chai, J.-D.; Head-Gordon, M. Long-Range Corrected Hybrid Density Functionals with Damped Atom–Atom Dispersion Corrections. Physical Chemistry Chemical Physics 2008, 10 (44), 6615. https://doi.org/10.1039/b810189b.

3. Zhao, Y.; Truhlar, D. G. The M06 Suite of Density Functionals for Main Group Thermochemistry, Thermochemical Kinetics, Noncovalent Interactions, Excited States, and Transition Elements: Two New Functionals and Systematic Testing of Four M06-Class Functionals and 12 Other Functionals. Theor Chem Acc 2008, 120 (1–3), 215–241. https://doi.org/10.1007/s00214-007-0310-x.

4. Weigend, F.; Ahlrichs, R. Balanced Basis Sets of Split Valence, Triple Zeta Valence and Quadruple Zeta Valence Quality for H to Rn: Design and Assessment of Accuracy. Physical Chemistry Chemical Physics 2005, 7 (18), 3297. https://doi.org/10.1039/b508541a.

5. Goerigk, L.; Grimme, S. A Thorough Benchmark of Density Functional Methods for General Main Group Thermochemistry, Kinetics, and Noncovalent Interactions. Physical Chemistry Chemical Physics 2011, 13 (14), 6670. https://doi.org/10.1039/c0cp02984j.

6. Lage, M. R.; Morbec, J. M.; Santos, M. H.; de M. Carneiro, J. W.; Costa, L. T. Natural Polyprenylated Benzophenone: Keto-Enol Tautomerism from Density Functional Calculations and the AIM Theory. J Mol Model 2017, 23 (4), 140. https://doi.org/10.1007/s00894-017-3251-x.

7. El-Demerdash, S. H.; Halim, S. A.; El-Nahas, A. M.; El-Meligy, A. B. A Density Functional Theory Study of the Molecular Structure, Reactivity, and Spectroscopic Properties of 2-(2-Mercaptophenyl)-1-Azaazulene Tautomers and Rotamers. Sci Rep 2023, 13 (1). https://doi.org/10.1038/s41598-023-42450-1.

8. Marenich, A. V.; Cramer, C. J.; Truhlar, D. G. Univer-sal Solvation Model Based on Solute Electron Density and on a Continuum Model of the Solvent De-fined by the Bulk Dielectric Constant and Atomic Surface Tensions. J Phys Chem B 2009, 113 (18), 6378–6396. https://doi.org/10.1021/jp810292n.

S5.3. Optimized cartesian coordinate matrices

Due to the similarity in the geometric structures of the systems evaluated across different media and levels of theory, we provide only the matrices optimized in the gas phase at the M06-2X/def2-TZVP level below.

E-azo-keto **-NH**

C	4.728283000	-1.687833000	-0.000329000
C	5.317217000	-0.422374000	0.000018000
C	4.542543000	0.721593000	0.000310000
C	3.157653000	0.602671000	0.000251000
C	2.555441000	-0.650951000	-0.000092000
C	3.354296000	-1.796218000	-0.000383000
O	2.452835000	1.767352000	0.000571000
C	1.079602000	1.818485000	0.000230000
C	0.374443000	0.518870000	0.000044000
C	1.091329000	-0.749991000	-0.000145000
O	0.563413000	2.890637000	0.000220000
O	0.508470000	-1.831979000	-0.000326000
N	-0.934541000	0.625602000	0.000038000
N	-1.683781000	-0.413151000	-0.000098000
C	-3.085870000	-0.291787000	-0.000071000
C	-3.835128000	-1.462575000	-0.000302000
C	-5.218589000	-1.386204000	-0.000276000
C	-5.849030000	-0.150389000	-0.000028000
C	-5.086887000	1.012348000	0.000199000
C	-3.703962000	0.953772000	0.000184000
H	5.346783000	-2.574780000	-0.000548000
H	6.395709000	-0.330188000	0.000064000
H	4.980280000	1.710311000	0.000593000
H	2.857865000	-2.758019000	-0.000653000
H	-1.237335000	-1.335312000	-0.000198000
H	-3.334077000	-2.423287000	-0.000509000
H	-5.803329000	-2.296521000	-0.000449000
H	-6.929095000	-0.091677000	-0.000009000
H	-5.575137000	1.978108000	0.000402000
H	-3.099202000	1.849631000	0.000353000

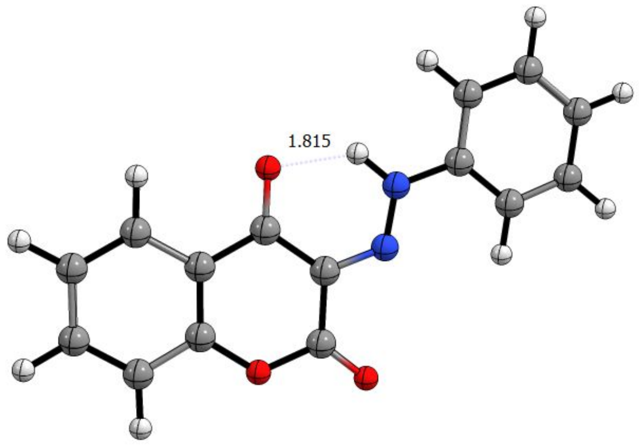

E = – 912.7680682 a. u.
H = – 912.526931 a. u.
G = – 912.586289 a. u.

Z-azo-keto **-NH**

C	-5.105367000	1.191050000	0.000179000
C	-5.409014000	-0.170213000	0.000179000
C	-4.403423000	-1.119085000	0.000085000
C	-3.081334000	-0.696942000	-0.000012000
C	-2.757400000	0.653224000	-0.000016000
C	-3.785880000	1.594614000	0.000082000
O	-2.133482000	-1.690170000	-0.000116000
C	-0.806352000	-1.425504000	-0.000071000
C	-0.372047000	-0.029524000	-0.000068000
C	-1.342100000	1.085642000	-0.000104000
O	-0.049900000	-2.369597000	-0.000020000
O	-1.019678000	2.250411000	-0.000136000
N	0.894111000	0.308954000	-0.000047000
N	1.841350000	-0.552169000	-0.000019000
C	3.188382000	-0.142875000	-0.000001000
C	4.164070000	-1.132349000	-0.000045000
C	5.501820000	-0.770867000	-0.000034000
C	5.862487000	0.568993000	0.000021000
C	4.875705000	1.548012000	0.000066000
C	3.534655000	1.204145000	0.000057000
H	-5.900632000	1.923829000	0.000258000
H	-6.441900000	-0.493762000	0.000254000
H	-4.615462000	-2.179439000	0.000081000
H	-3.507200000	2.640679000	0.000078000
H	1.603593000	-1.546512000	-0.000016000
H	3.873878000	-2.176454000	-0.000091000
H	6.262326000	-1.540516000	-0.000071000
H	6.907042000	0.850429000	0.000029000
H	5.152589000	2.594262000	0.000109000
H	2.756813000	1.954904000	0.000091000

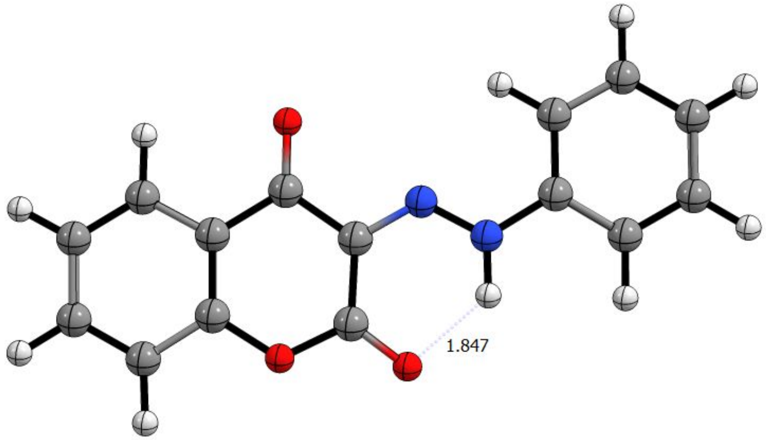

E = – 912.7668158 a. u.
H = – 912.525691 a. u.
G = – 912.585003 a.u.

Azo-enol **A-OH** (*E* form)

C	4.643901000	-1.743787000	0.000029000
C	5.276436000	-0.498531000	0.000169000
C	4.543327000	0.671816000	0.000223000
C	3.155127000	0.599994000	0.000132000
C	2.510325000	-0.635135000	-0.000006000
C	3.267833000	-1.810793000	-0.000055000
O	2.475037000	1.769242000	0.000180000
C	1.095667000	1.843653000	0.000047000
C	0.383021000	0.564561000	-0.000010000
C	1.067344000	-0.634825000	-0.000067000
O	0.599287000	2.926334000	0.000279000
O	0.453410000	-1.790860000	-0.000157000
N	-0.995932000	0.674110000	-0.000017000
N	-1.639967000	-0.397552000	-0.000102000
C	-3.052562000	-0.271120000	-0.000106000
C	-3.776067000	-1.457637000	-0.000199000
C	-5.162084000	-1.420695000	-0.000204000
C	-5.819857000	-0.199624000	-0.000117000
C	-5.089630000	0.985608000	-0.000025000
C	-3.707144000	0.959515000	-0.000019000
H	5.232911000	-2.650476000	-0.000010000
H	6.357418000	-0.445491000	0.000236000
H	5.014818000	1.644833000	0.000329000
H	2.748882000	-2.759820000	-0.000157000
H	-0.538942000	-1.576934000	-0.000171000
H	-3.241262000	-2.399204000	-0.000264000
H	-5.726586000	-2.343678000	-0.000276000
H	-6.901534000	-0.167420000	-0.000121000
H	-5.606114000	1.936673000	0.000041000
H	-3.123989000	1.869894000	0.000053000

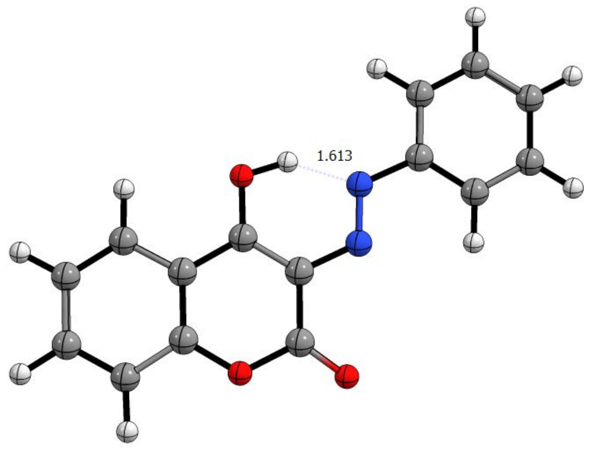

E = – 912.7599643 a. u.
H = – 912.519851 a. u.
G = – 912.578699 a. u.

Azo-enol **B-OH** (*Z* form)

C	-4.983298000	-1.341570000	-0.059638000
C	-5.385944000	-0.003988000	-0.020523000
C	-4.458125000	1.017467000	0.017676000
C	-3.103853000	0.700833000	0.017619000
C	-2.688205000	-0.628651000	-0.022034000
C	-3.641206000	-1.652109000	-0.060831000
O	-2.225438000	1.724465000	0.056344000
C	-0.845531000	1.562613000	0.061837000
C	-0.378010000	0.176070000	0.013820000
C	-1.270940000	-0.863797000	-0.024633000
O	-0.184950000	2.550840000	0.105540000
O	-0.865635000	-2.126028000	-0.069331000
N	0.956587000	-0.230497000	0.007390000
N	1.838810000	0.640301000	0.011094000
C	3.171305000	0.148813000	0.010277000
C	4.150245000	1.121994000	-0.158496000
C	5.489083000	0.763115000	-0.183149000
C	5.850124000	-0.567196000	-0.027344000
C	4.869887000	-1.538755000	0.155862000
C	3.532309000	-1.187888000	0.175230000
H	-5.723319000	-2.129275000	-0.090413000
H	-6.440764000	0.238648000	-0.020536000
H	-4.750086000	2.058182000	0.047588000
H	-3.300887000	-2.678298000	-0.091746000
H	0.108957000	-2.103152000	-0.072239000
H	3.832079000	2.150309000	-0.271160000
H	6.249231000	1.520963000	-0.319995000
H	6.894617000	-0.851022000	-0.040877000
H	5.155767000	-2.574333000	0.287934000
H	2.767775000	-1.936576000	0.327604000

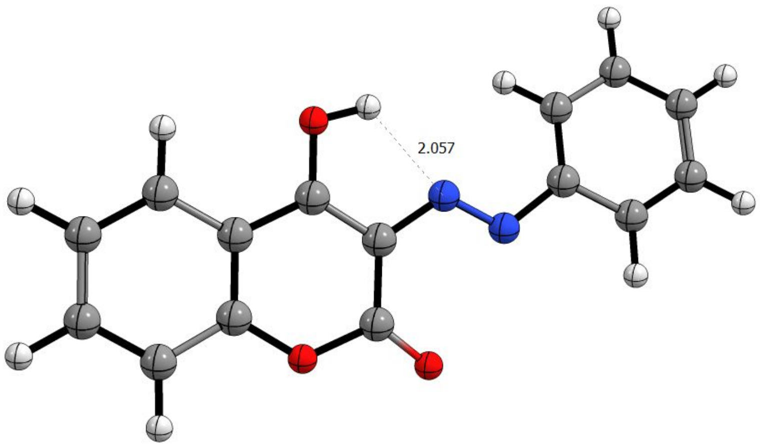

E = – 912.750383 a. u.
H = – 912.509520 a. u.
G = – 912.569782 a. u.

Azo-enol **C-OH** (*E* form)

C	-4.884039000	-1.490294000	-0.095695000
C	-5.370114000	-0.185881000	0.005354000
C	-4.500008000	0.884432000	0.078410000
C	-3.136856000	0.630025000	0.049676000
C	-2.625665000	-0.654086000	-0.049131000
C	-3.523585000	-1.718829000	-0.122627000
O	-2.309010000	1.727505000	0.124315000
C	-1.004635000	1.543956000	0.105630000
C	-0.369540000	0.327201000	0.016962000
C	-1.156519000	-0.900956000	-0.076746000
O	-0.362055000	2.686586000	0.187412000
O	-0.706832000	-2.019984000	-0.173650000
N	1.018676000	0.472404000	0.014097000
N	1.710555000	-0.555557000	0.006006000
C	3.113187000	-0.324523000	-0.015286000
C	3.899618000	-1.420426000	0.321520000
C	5.280939000	-1.303365000	0.337456000
C	5.877689000	-0.098068000	-0.004034000
C	5.089686000	0.990490000	-0.364880000
C	3.710133000	0.883565000	-0.371059000
H	-5.575556000	-2.319975000	-0.152987000
H	-6.436831000	-0.005814000	0.026437000
H	-4.847964000	1.905298000	0.156352000
H	-3.110160000	-2.716093000	-0.201077000
H	0.586787000	2.452457000	0.171831000
H	3.402958000	-2.349235000	0.570387000
H	5.891162000	-2.154703000	0.609351000
H	6.956082000	-0.006472000	-0.001910000
H	5.557661000	1.924348000	-0.649022000
H	3.092665000	1.719955000	-0.668001000

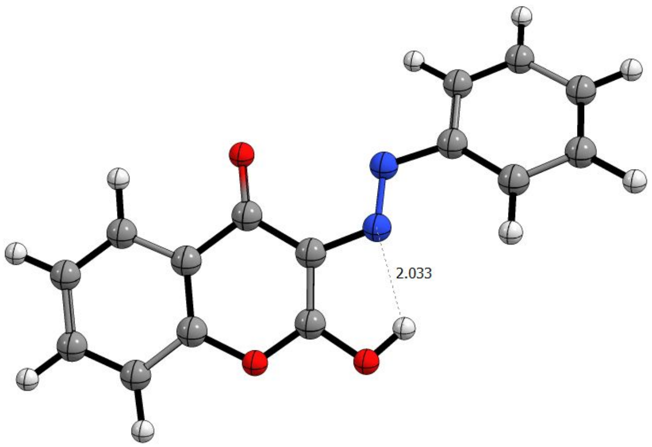

E = – 912.7387486 a. u.
H = – 912.497755 a. u.
G = – 912.557512 a. u.

Azo-enol **D-OH** (*Z* form)

C	-5.146045000	1.059078000	0.000036000
C	-5.385742000	-0.315324000	-0.000005000
C	-4.335532000	-1.213714000	-0.000026000
C	-3.040324000	-0.718518000	-0.000006000
C	-2.774288000	0.642834000	0.000034000
C	-3.849108000	1.530654000	0.000055000
O	-2.030934000	-1.656989000	-0.000028000
C	-0.766637000	-1.261447000	-0.000010000
C	-0.379141000	0.078020000	0.000044000
C	-1.375774000	1.144190000	0.000049000
O	0.075554000	-2.227924000	0.000028000
O	-1.115697000	2.326793000	0.000114000
N	0.948096000	0.431616000	0.000092000
N	1.791005000	-0.496039000	0.000099000
C	3.156370000	-0.119600000	0.000004000
C	4.086717000	-1.151597000	0.000012000
C	5.441860000	-0.857363000	-0.000072000
C	5.862931000	0.464229000	-0.000165000
C	4.925263000	1.492471000	-0.000172000
C	3.571180000	1.210592000	-0.000087000
H	-5.976839000	1.751485000	0.000052000
H	-6.401957000	-0.687068000	-0.000020000
H	-4.493823000	-2.283352000	-0.000057000
H	-3.622491000	2.589147000	0.000086000
H	1.029769000	-1.740324000	0.000097000
H	3.738031000	-2.176902000	0.000084000
H	6.166675000	-1.660695000	-0.000067000
H	6.920013000	0.695914000	-0.000231000
H	5.255475000	2.523179000	-0.000243000
H	2.829690000	1.997500000	-0.000089000

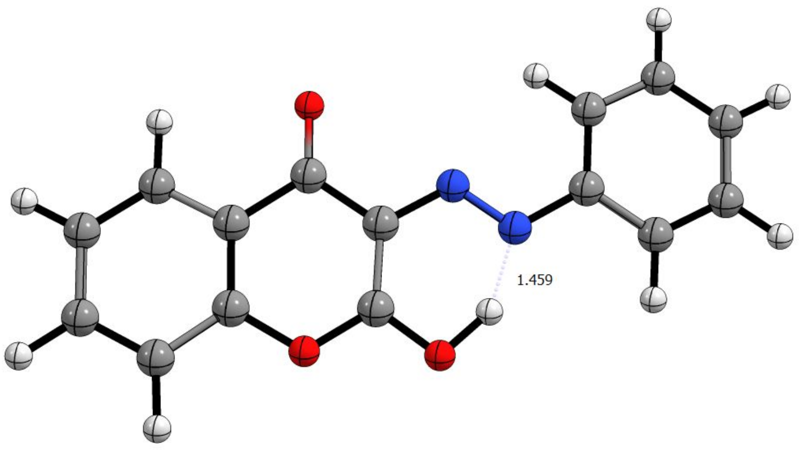

E = – 912.7505537 a. u.
H = – 912.511913 a. u.
G = – 912.571386 a. u.
